# Diretriz de Avaliação Cardiovascular Perioperatória da Sociedade Brasileira de Cardiologia – 2024

**DOI:** 10.36660/abc.20240590

**Published:** 2024-10-09

**Authors:** Danielle Menosi Gualandro, Luciana Savoy Fornari, Bruno Caramelli, Alexandre Antonio Cunha Abizaid, Brenno Rizerio Gomes, Caio de Assis Moura Tavares, Caio Julio Cesar dos Santos Fernandes, Carisi Anne Polanczyk, Carlos Jardim, Carolina Leticia Zilli Vieira, Claudio Pinho, Daniela Calderaro, Dirk Schreen, Fabiana Goulart Marcondes-Braga, Fábio de Souza, Francisco Akira Malta Cardozo, Flavio Tarasoutchi, Gabriel Assis Lopes Carmo, Gabriel Kanhouche, José Jayme Galvão de Lima, Luciana Dornfeld Bichuette, Luciana Sacilotto, Luciano Ferreira Drager, Luciano Janussi Vacanti, Luis Henrique Wolff Gowdak, Marcelo Luiz Campos Vieira, Marcelo Luiz Floriano Melo Martins, Márcio Silva Miguel Lima, Marcos Pita Lottenberg, Márlon Juliano Romero Aliberti, Mauricio Felippi de Sá Marchi, Milena Ribeiro Paixão, Mucio Tavares de Oliveira, Pai Ching Yu, Patricia Ramos Cury, Pedro Silvio Farsky, Ranna Santos Pessoa, Rinaldo Focaccia Siciliano, Tarso Augusto Duenhas Accorsi, Vinícius Machado Correia, Wilson Mathias

**Affiliations:** 1 Universidade de São Paulo Instituto do Coração Hospital das Clínicas da Faculdade de Medicina São Paulo SP Brasil Instituto do Coração (InCor) do Hospital das Clínicas da Faculdade de Medicina da Universidade de São Paulo(HCFMUSP), São Paulo, SP – Brasil; 2 University Hospital Basel Basel Switzerland University Hospital Basel, Basel – Suíça; 3 Fundação Zerbini São Paulo SP Brasil Fundação Zerbini, São Paulo, SP – Brasil; 4 Hospital Rio Grande Natal RN Brasil Hospital Rio Grande, Natal, RN – Brasil; 5 Hospital Israelita Albert Einstein São Paulo SP Brasil Hospital Israelita Albert Einstein, São Paulo, SP – Brasil; 6 Universidade Federal do Rio Grande do Sul Hospital de Clínicas Porto Alegre RS Brasil Hospital de Clínicas da Universidade Federal do Rio Grande do Sul (UFRS), Porto Alegre, RS – Brasil; 7 Universidade de São Paulo Hospital das Clínicas São Paulo SP Brasil Hospital das Clínicas da Faculdade de Medicina da Universidade de São Paulo (HCFMUSP), São Paulo, SP – Brasil; 8 Harvard University Massachusetts EUA Harvard University, Massachusetts – EUA; 9 Pontifícia Universidade Católica de Campinas Campinas SP Brasil Pontifícia Universidade Católica de Campinas (PUC-Campinas), Campinas, SP – Brasil; 10 Clinica Pinho Campinas SP Brasil Clinica Pinho, Campinas, SP – Brasil; 11 Hospital São Carlos Fortaleza CE Brasil Hospital São Carlos, Rede D’Or, Fortaleza, CE – Brasil; 12 Hospital Universitário Walter Cantidio Fortaleza CE Brasil Hospital Universitário Walter Cantidio da Universidade Federal do Ceará (UFC), Fortaleza, CE – Brasil; 13 Instituto de Medicina Nuclear Fortaleza CE Brasil Instituto de Medicina Nuclear, Fortaleza, CE – Brasil; 14 Universidade Federal do Estado do Rio de Janeiro Rio de Janeiro RJ Brasil Escola de Medicina e Cirurgia da Universidade Federal do Estado do Rio de Janeiro (UNIRIO), Rio de Janeiro, RJ – Brasil; 15 Universidade Federal de Minas Gerais Belo Horizonte MG Brasil Universidade Federal de Minas Gerais, Belo Horizonte, MG – Brasil; 16 Hospital Evangélico de Belo Horizonte Belo Horizonte MG Brasil Hospital Evangélico de Belo Horizonte, Belo Horizonte, MG – Brasil; 17 Hospital Orizonti Belo Horizonte MG Brasil Hospital Orizonti, Belo Horizonte, MG – Brasil; 18 Hospital de Base do Distrito Federal Brasília DF Brasil Hospital de Base do Distrito Federal, Brasília, DF – Brasil; 19 Centro Universitário Euroamericano Brasília DF Brasil Centro Universitário Euroamericano, Brasília, DF – Brasil; 20 Hospital São Marcos Teresina PI Brasil Hospital São Marcos, Teresina, PI – Brasil; 21 Universidade Federal da Bahia Salvador BA Brasil Universidade Federal da Bahia, Salvador, BA – Brasil; 22 Instituto Dante Pazzanese de Cardiologia São Paulo SP Brasil Instituto Dante Pazzanese de Cardiologia, São Paulo, SP – Brasil

**Table:** 

Diretriz de Avaliação Cardiovascular Perioperatória da Sociedade Brasileira de Cardiologia – 2024	
O relatório abaixo lista as declarações de interesse conforme relatadas à SBC pelos especialistas durante o período de desenvolvimento deste posicionamento, 2023-2024.	
Especialista	Tipo de relacionamento com a indústria
Alexandre Antonio Cunha Abizaid	Nada a ser declarado
Brenno Rizerio Gomes	Declaração financeira A - Pagamento de qualquer espécie e desde que economicamente apreciáveis, feitos a (i) você, (ii) ao seu cônjuge/companheiro ou a qualquer outro membro que resida com você, (iii) a qualquer pessoa jurídica em que qualquer destes seja controlador, sócio, acionista ou participante, de forma direta ou indireta, recebimento por palestras, aulas, atuação como proctor de treinamentos, remunerações, honorários pagos por participações em conselhos consultivos, de investigadores, ou outros comitês, etc. Provenientes da indústria farmacêutica, de órteses, próteses, equipamentos e implantes, brasileiras ou estrangeiras: – Astrazeneca: Forxiga; Novartis: Entresto; Bayer: Firialta. B - Financiamento de pesquisas sob sua responsabilidade direta/pessoal (direcionado ao departamento ou instituição) provenientes da indústria farmacêutica, de órteses, próteses, equipamentos e implantes, brasileiras ou estrangeiras: – B. Braun: Occlutech.
Bruno Caramelli	Nada a ser declarado
Caio de Assis Moura Tavares	Declaração financeira A - Pagamento de qualquer espécie e desde que economicamente apreciáveis, feitos a (i) você, (ii) ao seu cônjuge/companheiro ou a qualquer outro membro que resida com você, (iii) a qualquer pessoa jurídica em que qualquer destes seja controlador, sócio, acionista ou participante, de forma direta ou indireta, recebimento por palestras, aulas, atuação como proctor de treinamentos, remunerações, honorários pagos por participações em conselhos consultivos, de investigadores, ou outros comitês, etc. Provenientes da indústria farmacêutica, de órteses, próteses, equipamentos e implantes, brasileiras ou estrangeiras: – Novo Nordisk.
Caio Julio Cesar dos Santos Fernandes	Declaração financeira A - Pagamento de qualquer espécie e desde que economicamente apreciáveis, feitos a (i) você, (ii) ao seu cônjuge/companheiro ou a qualquer outro membro que resida com você, (iii) a qualquer pessoa jurídica em que qualquer destes seja controlador, sócio, acionista ou participante, de forma direta ou indireta, recebimento por palestras, aulas, atuação como proctor de treinamentos, remunerações, honorários pagos por participações em conselhos consultivos, de investigadores, ou outros comitês, etc. Provenientes da indústria farmacêutica, de órteses, próteses, equipamentos e implantes, brasileiras ou estrangeiras: – Janssen: Uptravi; Bayer: Riociguate; MSD: Sotatercept. B - Financiamento de pesquisas sob sua responsabilidade direta/pessoal (direcionado ao departamento ou instituição) provenientes da indústria farmacêutica, de órteses, próteses, equipamentos e implantes, brasileiras ou estrangeiras: – Janssen: Uptravii. Outros relacionamentos Financiamento de atividades de educação médica continuada, incluindo viagens, hospedagens e inscrições para congressos e cursos, provenientes da indústria farmacêutica, de órteses, próteses, equipamentos e implantes, brasileiras ou estrangeiras: – Janssen: Uptravii.
Carisi Anne Polanczyk	Nada a ser declarado
Carlos Jardim	Nada a ser declarado
Carolina Leticia Zilli Vieira	Nada a ser declarado
Claudio Pinho	Nada a ser declarado
Daniela Calderaro	Declaração financeira A - Pagamento de qualquer espécie e desde que economicamente apreciáveis, feitos a (i) você, (ii) ao seu cônjuge/ companheiro ou a qualquer outro membro que resida com você, (iii) a qualquer pessoa jurídica em que qualquer destes seja controlador, sócio, acionista ou participante, de forma direta ou indireta, recebimento por palestras, aulas, atuação como proctor de treinamentos, remunerações, honorários pagos por participações em conselhos consultivos, de investigadores, ou outros comitês, etc. Provenientes da indústria farmacêutica, de órteses, próteses, equipamentos e implantes, brasileiras ou estrangeiras: – Bayer: fibrilação atrial, TEV, nefropatia diabética. Outros relacionamentos Financiamento de atividades de educação médica continuada, incluindo viagens, hospedagens e inscrições para congressos e cursos, provenientes da indústria farmacêutica, de órteses, próteses, equipamentos e implantes, brasileiras ou estrangeiras: – Bayer: nefropatia diabética.
Danielle Menosi Gualandro	Declaração financeira A - Pagamento de qualquer espécie e desde que economicamente apreciáveis, feitos a (i) você, (ii) ao seu cônjuge/ companheiro ou a qualquer outro membro que resida com você, (iii) a qualquer pessoa jurídica em que qualquer destes seja controlador, sócio, acionista ou participante, de forma direta ou indireta, recebimento por palestras, aulas, atuação como proctor de treinamentos, remunerações, honorários pagos por participações em conselhos consultivos, de investigadores, ou outros comitês, etc. Provenientes da indústria farmacêutica, de órteses, próteses, equipamentos e implantes, brasileiras ou estrangeiras: – Roche.
Dirk Schreen	Nada a ser declarado
Fabiana Goulart Marcondes-Braga	Declaração financeira A - Pagamento de qualquer espécie e desde que economicamente apreciáveis, feitos a (i) você, (ii) ao seu cônjuge/ companheiro ou a qualquer outro membro que resida com você, (iii) a qualquer pessoa jurídica em que qualquer destes seja controlador, sócio, acionista ou participante, de forma direta ou indireta, recebimento por palestras, aulas, atuação como proctor de treinamentos, remunerações, honorários pagos por participações em conselhos consultivos, de investigadores, ou outros comitês, etc. Provenientes da indústria farmacêutica, de órteses, próteses, equipamentos e implantes, brasileiras ou estrangeiras: – Palestras e Comitê Consultivo: AstraZeneca; Boehringher-Lilly; Novartis; Bayer. B - Financiamento de pesquisas sob sua responsabilidade direta/pessoal (direcionado ao departamento ou instituição) provenientes da indústria farmacêutica, de órteses, próteses, equipamentos e implantes, brasileiras ou estrangeiras: – MSD: Victor Trial.
Fábio de Souza	Declaração financeira A - Pagamento de qualquer espécie e desde que economicamente apreciáveis, feitos a (i) você, (ii) ao seu cônjuge/ companheiro ou a qualquer outro membro que resida com você, (iii) a qualquer pessoa jurídica em que qualquer destes seja controlador, sócio, acionista ou participante, de forma direta ou indireta, recebimento por palestras, aulas, atuação como proctor de treinamentos, remunerações, honorários pagos por participações em conselhos consultivos, de investigadores, ou outros comitês, etc. Provenientes da indústria farmacêutica, de órteses, próteses, equipamentos e implantes, brasileiras ou estrangeiras: – Pfizer: Amiloidose. Outros relacionamentos Financiamento de atividades de educação médica continuada, incluindo viagens, hospedagens e inscrições para congressos e cursos, provenientes da indústria farmacêutica, de órteses, próteses, equipamentos e implantes, brasileiras ou estrangeiras: – Pfizer: amiloidose; AstraZeneca: amiloidose.
Flávio Tarasoutchi	Outros relacionamentos Financiamento de atividades de educação médica continuada, incluindo viagens, hospedagens e inscrições para congressos e cursos, provenientes da indústria farmacêutica, de órteses, próteses, equipamentos e implantes, brasileiras ou estrangeiras: – Edwads.
Francisco Akira Malta Cardozo	Declaração financeira A - Pagamento de qualquer espécie e desde que economicamente apreciáveis, feitos a (i) você, (ii) ao seu cônjuge/ companheiro ou a qualquer outro membro que resida com você, (iii) a qualquer pessoa jurídica em que qualquer destes seja controlador, sócio, acionista ou participante, de forma direta ou indireta, recebimento por palestras, aulas, atuação como proctor de treinamentos, remunerações, honorários pagos por participações em conselhos consultivos, de investigadores, ou outros comitês, etc. Provenientes da indústria farmacêutica, de órteses, próteses, equipamentos e implantes, brasileiras ou estrangeiras: – Bayer: Xarelto; Daiichi Sankyo: Lixiana; Novo Nordisk: Rybelsius. Outros relacionamentos Financiamento de atividades de educação médica continuada, incluindo viagens, hospedagens e inscrições para congressos e cursos, provenientes da indústria farmacêutica, de órteses, próteses, equipamentos e implantes, brasileiras ou estrangeiras: – Daiichi Sankyo.
Gabriel Assis Lopes do Carmo	Nada a ser declarado
Gabriel Kanhouche	Nada a ser declarado
José Jayme Galvão de Lima	Nada a ser declarado
Luciana Dornfeld Bichuette	Nada a ser declarado
Luciana Sacilotto	Nada a ser declarado
Luciana Savoy Fornari	Nada a ser declarado
Luciano Ferreira Drager	Nada a ser declarado
Luciano Janussi Vacanti	Nada a ser declarado
Luis Henrique Wolff Gowdak	Declaração financeira A - Pagamento de qualquer espécie e desde que economicamente apreciáveis, feitos a (i) você, (ii) ao seu cônjuge/ companheiro ou a qualquer outro membro que resida com você, (iii) a qualquer pessoa jurídica em que qualquer destes seja controlador, sócio, acionista ou participante, de forma direta ou indireta, recebimento por palestras, aulas, atuação como proctor de treinamentos, remunerações, honorários pagos por participações em conselhos consultivos, de investigadores, ou outros comitês, etc. Provenientes da indústria farmacêutica, de órteses, próteses, equipamentos e implantes, brasileiras ou estrangeiras: – Servier: síndrome coronariana crônica; Novartis: hipercolesterolemia. B - Financiamento de pesquisas sob sua responsabilidade direta/pessoal (direcionado ao departamento ou instituição) provenientes da indústria farmacêutica, de órteses, próteses, equipamentos e implantes, brasileiras ou estrangeiras: – Servier: síndrome coronariana crônica. Outros relacionamentos Financiamento de atividades de educação médica continuada, incluindo viagens, hospedagens e inscrições para congressos e cursos, provenientes da indústria farmacêutica, de órteses, próteses, equipamentos e implantes, brasileiras ou estrangeiras: – Servier: síndrome coronariana crônica.
Marcelo Luiz Campos Vieira	Nada a ser declarado
Marcelo Luiz Floriano Melo Martins	Nada a ser declarado
Márcio Silva Miguel Lima	Nada a ser declarado
Marcos Pita Lottenberg	Nada a ser declarado
Márlon Juliano Romero Aliberti	Nada a ser declarado
Mauricio Felippi de Sá Marchi	Nada a ser declarado
Milena Ribeiro Paixão	Nada a ser declarado
Mucio Tavares de Oliveira Junior	Declaração financeira B - Financiamento de pesquisas sob sua responsabilidade direta/pessoal (direcionado ao departamento ou instituição) provenientes da indústria farmacêutica, de órteses, próteses, equipamentos e implantes, brasileiras ou estrangeiras: – Sanofi; Pasteur.
Pai Ching Yu	Nada a ser declarado
Patricia Ramos Cury	Nada a ser declarado
Pedro Silvio Farsky	Declaração financeira A - Pagamento de qualquer espécie e desde que economicamente apreciáveis, feitos a (i) você, (ii) ao seu cônjuge/ companheiro ou a qualquer outro membro que resida com você, (iii) a qualquer pessoa jurídica em que qualquer destes seja controlador, sócio, acionista ou participante, de forma direta ou indireta, recebimento por palestras, aulas, atuação como proctor de treinamentos, remunerações, honorários pagos por participações em conselhos consultivos, de investigadores, ou outros comitês, etc. Provenientes da indústria farmacêutica, de órteses, próteses, equipamentos e implantes, brasileiras ou estrangeiras: – Novo Nordisk: semaglutida; Lilly: empagliflozina e tirzepatida; AstraZeneca: dapagliflozina; GSK: vacina shingrix. Outros relacionamentos Financiamento de atividades de educação médica continuada, incluindo viagens, hospedagens e inscrições para congressos e cursos, provenientes da indústria farmacêutica, de órteses, próteses, equipamentos e implantes, brasileiras ou estrangeiras: – Novo Nordisk: semaglutida.
Ranna Santos Pessoa	Nada a ser declarado
Rinaldo Focaccia Siciliano	Nada a ser declarado
Tarso Augusto Duenhas Accorsi	Declaração financeira A - Pagamento de qualquer espécie e desde que economicamente apreciáveis, feitos a (i) você, (ii) ao seu cônjuge/ companheiro ou a qualquer outro membro que resida com você, (iii) a qualquer pessoa jurídica em que qualquer destes seja controlador, sócio, acionista ou participante, de forma direta ou indireta, recebimento por palestras, aulas, atuação como proctor de treinamentos, remunerações, honorários pagos por participações em conselhos consultivos, de investigadores, ou outros comitês, etc. Provenientes da indústria farmacêutica, de órteses, próteses, equipamentos e implantes, brasileiras ou estrangeiras: – Abbott: MitraClip; Boston: Watchman; Edwards: cirurgia e TAVI; Novartis: Sybrava. Outros relacionamentos Financiamento de atividades de educação médica continuada, incluindo viagens, hospedagens e inscrições para congressos e cursos, provenientes da indústria farmacêutica, de órteses, próteses, equipamentos e implantes, brasileiras ou estrangeiras: – Novo Nordisk: Congresso Brasileiro da SBC; EMS: Congresso de Departamentos da SBC.
Vinícius Machado Correia	Declaração financeira A - Pagamento de qualquer espécie e desde que economicamente apreciáveis, feitos a (i) você, (ii) ao seu cônjuge/ companheiro ou a qualquer outro membro que resida com você, (iii) a qualquer pessoa jurídica em que qualquer destes seja controlador, sócio, acionista ou participante, de forma direta ou indireta, recebimento por palestras, aulas, atuação como proctor de treinamentos, remunerações, honorários pagos por participações em conselhos consultivos, de investigadores, ou outros comitês, etc. Provenientes da indústria farmacêutica, de órteses, próteses, equipamentos e implantes, brasileiras ou estrangeiras: – Novo Nordisk: palestra sobre tratamento farmacológico da obesidade.
Wilson Mathias Junior	Nada a ser declarado

**Sumário**

**1. Definição do Problema** 08

1.1. Objetivo da Diretriz 08

1.2. Metodologia e Evidências 08

1.3. Particularidades do Perioperatório 08

1.4. Criação do Perioperative Risk Team 08

**2. Avaliação Pré-operatória Geral e Suplementar** 11

2.1. Estratificação do Risco Pré-operatório 11

**2.1.1. Condições Cardiovasculares Graves no Perioperatório** 11

**2.1.2. Estimativa do Risco Intrínseco Relacionado ao Tipo de**

**Operação** 11

**2.1.3. Avaliação da Capacidade Funcional** 12

**2.1.4. Ferramentas para Estimar o Risco Cardíaco Perioperatório** 12

**2.1.5. Avaliação da Fragilidade** 14

***2.1.5.1. Como Avaliar Fragilidade Antes de Cirurgia Não Cardíaca*** 14

***2.5.1.2. Impacto da Avaliação de Fragilidade em Cirurgia Não***

***Cardíac a*** 15

2.2. Eletrocardiograma 16

2.3. Ecocardiograma 17

2.4. Testes Não Invasivos para Detecção de Isquemia Miocárdica 17

**2.4.1. Eletrocardiograma de Esforço** 17

**2.4.2. Cintilografia de Perfusão Miocárdica com Estresse** 18

**2.4.3. Ecocardiograma de Estresse com Dobutamina** 18

**2.4.4. Sumário de Recomendações para Realização dos Testes Não**

**Invasivos** 19

2.5. Cineangiocoronariografia 19

2.6. Angiotomografia de Coronárias 19

**3. Doenças e Procedimentos com Aspectos Específicos no**

**Perioperatório** 20

3.1. Insuficiência Cardíaca 20

3.2. Hipertensão Arterial 21

**3.2.1. Manejo Pressórico no Pré-operatório** 21

**3.2.2. Manejo Pressórico no Intraoperatório** 22

**3.2.3. Manejo Pressórico no Pós-operatório** 22

**3.2.4. Anti-hipertensivos no Perioperatório** 22

***3.2.4.1. Antagonistas do Sistema Renina-Angiotensina-Aldosterona*** 22

***3.2.4.2. Bloqueadores do Canal de Cálcio*** 23

***3.2.4.3. Diuréticos*** 23

***3.2.4.4. Simpatolíticos de Ação Central*** 23

**3.2.5. Considerações Finais** 23

3.3. Conduta em Procedimentos de Baixo Risco 23

**3.3.1. Odontológicos** 24

***3.3.1.1. Agentes Antitrombóticos*** 24

***3.3.1.2. Dispositivos Cardíacos Eletrônicos Implantáveis*** 24

***3.3.1.3. Anestésicos Locais*** 24

**3.3.2. Dermatológicos** 24

**3.3.3. Endoscópicos** 25

***3.3.3.1. Manejo de Antiplaquetários em Procedimentos Endoscópicos*** 25

***3.3.3.2. Manejo de Anticoagulantes em Procedimentos Endoscópicos*** 26

**3.3.4. Oftalmológicos** 27

3.4. Doença Valvar 27

**3.4.1. Estenose Aórtica** 28

**3.4.2. Estenose Mitral** 29

**3.4.3. Insuficiência Aórtica e InsuficiêNcia Mitral** 29

**3.4.4. Prótese Valvar** 29

3.5. Transplante de Órgãos Sólidos 29

**3.5.1. Fígado** 29

***3.5.1.1. Cardiomiopatia Associada à Cirrose*** 30

***3.5.1.2. Cardiopatia Alcoólica*** 30

***3.5.1.3. Hipertensão Portopulmonar*** 30

***3.5.1.4. Síndrome Hepatopulmonar*** 31

***3.5.1.5. Doença Arterial Coronariana*** 32

***3.5.1.5.1. Quando Investigar DAC?*** 32

***3.5.1.5.2. Qual Exame Solicitar para Investigar DAC?*** 32

***3.5.1.5.3. Quando e como Intervir na DAC?*** 32

**3.5.2. Rim** 33

***3.5.2.1. Estratificação do Risco da Presença de DAC*** 34

**4. Medidas para Redução do Risco Cirúrgico do Ponto de**

**Vista CV** 35

4.1. Terapia Medicamentosa Perioperatória 35

**4.1.1. Betabloqueadores** 35

**4.1.2. Estatinas** 35

**4.1.3. Antiagregantes Plaquetários** 37

***4.1.3.1. Monoterapia com Ácido Acetilsalicílico*** 37

***4.1.3.2. Monoterapia Antiagregante com Outros Fármacos que Não o***

***Ácido Acetilsalicílico*** 37

***4.1.3.3. Dupla Antiagregação Plaquetária*** 38

4.2. Revascularização Miocárdica 38

4.3. Manejo da Anticoagulação no Perioperatório 39

**4.3.1. Varfarina** 40

***4.3.1.1. Pacientes com Risco Moderado para Tromboembolismo*** 41

***4.3.1.2. Procedimentos de Urgência ou Emergência*** 41

**4.3.2. Anticoagulantes Diretos** 41

***4.3.2.1. Dabigatrana*** 42

***4.3.2.2. Rivaroxabana*** 43

***4.3.2.3. Apixabana*** 43

***4.3.2.4. Edoxabana*** 43

***4.3.2.5. Avaliação do Efeito Anticoagulante dos DOACs*** 43

***4.3.2.6. Tempo Ideal para Suspensão dos DOACs Antes de Cirurgias***

***Eletivas*** 43

4.4. Profilaxia de Endocardite Infecciosa 44

**4.4.1. Procedimentos Odontológicos** 45

**4.4.2. Procedimentos no Trato Respiratório** 46

**4.4.3. Procedimentos no Trato Geniturinário e Gastrointestinal** 46

**4.4.4. Procedimentos Dermatológicos e Musculoesqueléticos** 47

**4.4.5. Piercing e Tatuagem** 47

**5. Biomarcadores no Perioperatório** 47

5.1. Peptídeos Natriuréticos 47

5.2. Troponinas Cardíacas e Monitorização de Complicações

Cardiovasculares 48

**6. Diagnóstico e Tratamento das Complicações**

**Cardiovasculares no Perioperatório** 49

6.1. Injúria/Infarto Agudo do Miocárdio Perioperatório 49

6.2. Fibrilação/Flutter Atrial Agudos 52

6.3. Insuficiência Cardíaca Aguda 53

6.4. Tromboembolismo Venoso 54

**Referências** 55

**Figure:**
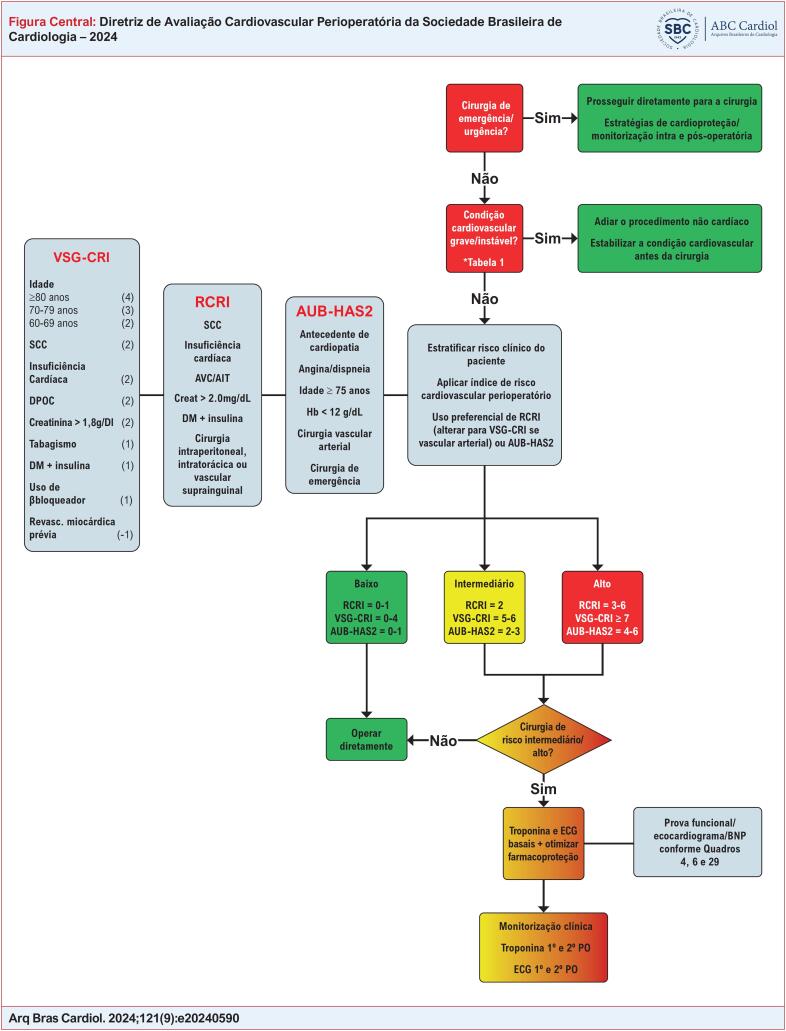


## 1. Definição do Problema

### 1.1. Objetivo da Diretriz

A evolução do conhecimento na medicina tem acontecido de forma vertiginosa nos últimos anos, e as diretrizes têm como função filtrar as melhores evidências e organizá-las para que possam ser postas em prática no exercício diário da medicina. A necessidade de atualização varia temporalmente em cada área e reflete a quantidade e qualidade das pesquisas médicas no assunto em questão. O interesse pelo tema de perioperatório em cirurgias não cardíacas é muito recente, tendo se iniciado em 1996, após a publicação do trabalho de Mangano et al., que observou redução de complicações cardiovasculares (CV) em pacientes que receberam atenolol por via venosa no pré-operatório imediato.^1^ No Brasil, surgiram publicações a partir de 2003, sendo que a I Diretriz de Avaliação Perioperatória foi publicada em 2007 e atualizada em 2011 e 2017.^2^ Esta é a quarta atualização no período de 15 anos, demonstrando, assim, a importância do tema tanto no âmbito de pesquisa como na prática profissional cotidiana.

Apesar de a porcentagem de complicações perioperatórias ser pequena, a sua ocorrência no Brasil ainda é significativamente maior do que em outros países. Além disso, a maioria dos procedimentos é de baixo risco, mas, considerando o volume anual de mais de 8 milhões de intervenções cirúrgicas no país, há quantidade expressiva, em números absolutos, de pacientes com risco elevado de complicações que requerem maior cuidado.

Os objetivos principais desta Diretriz de Avaliação Cardiovascular Perioperatória da Sociedade Brasileira de Cardiologia – 2024 são:

Oferecer e uniformizar o conhecimento atualizado sobre a estratificação do risco de complicações;

Estabelecer métodos adequados para a estratificação do risco e para o diagnóstico de complicações não só para melhorar a eficiência, como também para reduzir o custo com exames desnecessários;

Oferecer conhecimento atualizado sobre a relação entre as diversas comorbidades e as doenças CV que podem interagir no contexto perioperatório, como interação e suspensão adequada de medicações ou tempo seguro para realizar o procedimento cirúrgico após intervenção CV;

Oferecer recomendações sobre o local ideal para a realização da intervenção para pacientes de maior risco e até mesmo contraindicar a operação quando o risco de complicações supera o potencial benefício dela.

### 1.2. Metodologia e Evidências

A metodologia e os níveis de evidência para esta Diretriz são aqueles adotados pela SBC e abaixo referidos:

Classes (Graus) de recomendação:

✓ Classe I – Condições para as quais há evidências conclusivas ou, na sua falta, consenso de que o procedimento é seguro e útil/eficaz.
✓ Classe II – Condições para as quais há evidências conflitantes e/ou divergência de opinião sobre segurança e utilidade/eficácia do procedimento.
✓ Classe IIA – Peso ou evidência/opinião a favor do procedimento. A maioria aprova.
✓ Classe IIB – Segurança e utilidade/eficácia menos bem estabelecida, não havendo predomínio de opiniões a favor.
✓ Classe III – Condições para as quais há evidências e/ou consenso de que o procedimento não é útil/eficaz e, em alguns casos, pode ser prejudicial.

Níveis de evidência:

✓ Nível A – Dados obtidos a partir de múltiplos estudos randomizados de bom porte, concordantes e/ou de metanálise robusta de estudos clínicos randomizados.
✓ Nível B – Dados obtidos a partir de metanálise menos robusta, de um único estudo randomizado ou de estudos não randomizados (observacionais).
✓ Nível C – Dados obtidos de opiniões consensuais de especialistas.

Todas as evidências científicas usadas nesta Diretriz foram retiradas de periódicos indexados, com conselho editorial e revisores gabaritados.

### 1.3. Particularidades do Perioperatório

O período perioperatório no paciente com cardiopatia apresenta muitas peculiaridades que divergem da prática clínica habitual como o uso de medicações anticoagulantes e antitrombóticas em pacientes submetidos a intervenções cirúrgicas, uma vez que tais medicamentos nem sempre podem ser descontinuados, gerando um maior risco de sangramento intraoperatório. Além disto, é muito importante salientar que ferramentas de estratificação de risco de complicações CV usadas pelos cardiologistas, como o Escores ATP-3, Framingham ou CHADS-VASc, estabelecem perfis de risco em anos e não são adequadas para prever o risco no curto período perioperatório de uma intervenção cirúrgica não cardíaca, na maioria das vezes inferior a 30 dias. Por essas razões, a Diretriz de Avaliação Cardiovascular Perioperatória da Sociedade Brasileira de Cardiologia – 2024 traz informações relevantes especificamente para avaliação do risco de eventos CV, formas de prevenção e recomendações para tornar o período perioperatório de cirurgias não cardíacas mais seguro para o paciente com cardiopatia. O sumário das novidades incluídas na Diretriz de Avaliação Cardiovascular Perioperatória da Sociedade Brasileira de Cardiologia – 2024 pode ser consultado na Tabela 1 .

**Tabela 1:** Novas recomendações incluídas na Diretriz de Avaliação Cardiovascular Perioperatória da Sociedade Brasileira de Cardiologia – 2024

Recomendação		Classe
Avaliação pré-operatória geral e suplementar: Estratificação de risco pré-operatório		
Avaliação da capacidade funcional		
Determinar a capacidade funcional de pacientes em programação de operações de risco intermediário ou alto durante a anamnese (baseado na habilidade de subir dois lances de escada).		**I**
Avaliação da fragilidade		
A fragilidade deve ser avaliada rotineiramente em idosos submetidos a cirurgias de risco intermediário ou alto.		**IIa**
A fragilidade deve ser mensurada objetivamente através de instrumento específico.		**IIa**
Eletrocardiograma		
Pacientes submetidos a operações de risco intrínseco intermediário ou alto de complicações cardiovasculares.		**I**
Pacientes com risco intermediário ou alto de eventos cardiovasculares perioperatórios estimado pelos algoritmos.		**I**
Testes não invasivos para detecção de isquemia miocárdica		
Eletrocardiograma de esforço		
	Solicitação de teste ergométrico em pacientes com baixa capacidade funcional, de risco intermediário ou alto, em programação de cirurgia não cardíaca eletiva de risco intermediário ou alto em que o teste poderá potencialmente alterar a conduta.	**IIb**
Cintilografia de perfusão miocárdica com estresse/ecocardiograma com estresse		
	Solicitação de provas funcionais com imagem em pacientes com baixa capacidade funcional, de risco intermediário ou alto, em programação de cirurgia não cardíaca eletiva de risco intermediário ou alto, em que a prova funcional poderá potencialmente alterar a conduta.	**IIa**
	Solicitação de provas funcionais em pacientes com baixa capacidade funcional, assintomáticos, com diagnóstico prévio ou alta probabilidade de doença arterial coronariana.	**IIb**
**Doenças e procedimentos com aspectos específicos no perioperatório**		
**Hipertensão arterial**		
Deve-se evitar, durante todo o período perioperatório, episódios de hipotensão.		**I**
Pacientes com suspeita de hipertensão arterial secundária devem ser investigados antes da cirurgia, salvo em casos de urgência/emergência.		**I**
Pacientes em uso crônico de antagonistas do sistema renina-angiotensina-aldosterona podem ter a medicação mantida no perioperatório, sendo a suspensão permitida em casos selecionados.		**I**
Pacientes em uso crônico de bloqueadores de canal de cálcio podem ter a medicação mantida no perioperatório, sendo a suspensão permitida em casos selecionados.		**I**
Pacientes em uso crônico de diuréticos podem ter a medicação mantida no perioperatório, sendo a suspensão permitida em casos selecionados.		**I**
Pacientes em uso crônico de clonidina devem ter a medicação mantida no perioperatório.		**I**
**Conduta em procedimentos de baixo risco**		
**Odontológicos**		
	Avaliação e tratamento odontológico são importantes no perioperatório.	**IIa**
	Uso de solução antimicrobiana oral antes e após os procedimentos odontológicos e cirurgias não cardíacas de risco intermediário e alto.	**IIa**
	O uso de anestésicos com 1-4 ampolas de vasoconstritor é seguro para tratamento dentário de pacientes cardiopatas.	**IIa**
	Manter tratamento antiplaquetário e anticoagulante na maioria dos tratamentos odontológicos, incluindo extração de até dois dentes, restaurações, próteses, endodontia, limpezas e implantes.	**IIa**
**Doença valvar**		
Pacientes com estenose mitral importante, assintomática, com complicadores (pressão arterial pulmonar sistólica [PSAP] ≥ 50 mmHg no repouso ou PSAP ≥ 60 mmHg ao esforço) deverão ser submetidos ao tratamento da valvopatia antes da cirurgia não cardíaca.		**I**
**Transplante de órgãos sólidos**		
**Fígado**		
	Para pacientes com ecocardiograma mostrando PSAP acima de 50 mmHg, deve ser solicitado cateterismo cardíaco direito com medida da pressão em artéria pulmonar.	**I**
	Para pacientes com ecocardiograma mostrando PSAP acima de 40 mmHg, principalmente se outros sinais de hipertensão pulmonar estiverem presentes, deve-se considerar o cateterismo cardíaco direito com medida da pressão em artéria pulmonar.	**IIa**
	Para pacientes assintomáticos para doença arterial coronariana (DAC) sem disfunção segmentar ao ecocardiograma e com dois ou mais fatores de risco para DAC, solicitar preferencialmente um ecocardiograma transtorácico com dobutamina.	**IIa**
	Para pacientes sintomáticos para DAC, calcular a probabilidade pré-teste de DAC e solicitar os exames adicionais conforme as diretrizes específicas.	**IIa**
	A cineangiocoronariografia deve ser realizada em pacientes com alta probabilidade pré-teste de DAC, angina importante e refratária ao tratamento clínico, disfunção nova de ventrículo esquerdo ou achados de alto risco nos testes não invasivos, apesar de as complicações hemorrágicas serem mais comuns e alterações como elevação da creatinina poderem contribuir para o aumento na morbidade dos pacientes com cirrose.	**IIa**
	A cirurgia de revascularização miocárdica antes do transplante deve ser reservada apenas para pacientes em que o risco de morte pela DAC supere o risco de morte pela doença hepática, e a decisão deve ser compartilhada com uma equipe multidisciplinar.	**IIa**
	Pacientes com pressão arterial pulmonar média maior ou igual a 35 mmHg no cateterismo direito devem ser encaminhados ao especialista em hipertensão pulmonar.	**IIb**
**Rim**		
	Todos os pacientes candidatos a transplante renal devem ser avaliados quanto à presença e à gravidade da doença cardiovascular com base na história clínica, exame físico e exames rotineiros.	**I**
	Recomenda-se a dosagem de troponina ultrassensível antes, 24 h e 48 h após o transplante renal para detecção de injúria/infarto perioperatório.	**I**
	Recomenda-se que a tomada de decisão diagnóstica e a definição de estratégia terapêutica sejam discutidas e decididas por um "Heart-Kidney Team" incluindo cardiologista clínico, cardiologista intervencionista, cirurgião cardiovascular, nefrologista e/ou especialista em transplante renal.	**I**
	Pacientes estáveis com DAC obstrutiva devem ser reavaliados clinicamente quanto à progressão da doença a cada 12 meses; pacientes sem DAC obstrutiva significativa devem ser reavaliados a cada 36 meses para detecção de DAC *de novo* .	**IIa**
**Medidas para redução de risco cirúrgico do ponto de vista cardiovascular**		
**Terapia médica perioperatória**		
**Betabloqueadores**		
	Pacientes em uso crônico de betabloqueadores devem ter a medicação mantida no perioperatório.	**I**
**Estatinas**		
	Pacientes submetidos a operações não vasculares com indicação clínica do uso de estatinas devido a doenças associadas (DAC, doença cerebrovascular, doença arterial periférica, diabetes) independentemente do contexto do perioperatório.	**IIa**
**Dupla antiagregação plaquetária**		
	Em caso de interrupção de dupla antiagregação plaquetária (DAP) antes do tempo mínimo ideal, realizar cirurgias não cardíacas em centros com suporte multidisciplinar e retaguarda hemodinâmica.	**I**
	Utilizar teste de agregabilidade plaquetária para abreviar tempo de suspensão de inibidor de P2Y12 antes de cirurgias não cardíacas.	**IIb**
	Para casos de risco trombótico muito elevado (menos de 1 mês de intervenção coronária percutânea) e interrupção de DAPT, utilizar tirofibana como terapia de ponte.	**IIb**
	Utilizar teste de agregabilidade plaquetária como rotina para avaliar suspensão de ácido acetilsalicílico ou de inibidor de P2Y12 antes de cirurgias não cardíacas.	**III**
**Revascularização miocárdica**		
Recomendações para o intervalo entre a revascularização miocárdica e a operação não cardíaca em pacientes submetidos a intervenções coronárias percutâneas eletivas:		
	– ≥ 6 meses	**I**
	– Entre 3 e 6 meses	**IIa**
	– Entre 30 dias e 3 meses	**IIb**
	– < 30 dias	**III**
Recomendações para o intervalo entre a revascularização miocárdica e a operação não cardíaca em pacientes submetidos a intervenções coronárias percutâneas devido a síndromes coronarianas agudas:		
	– ≥ 12 meses	**I**
	– Entre 6 e 12 meses	**IIa**
	– Entre 30 dias e 6 meses	**IIb**
	– < 30 dias	**III**
**Biomarcadores no perioperatório**		
**Peptídeos natriuréticos**		
	Pacientes com idade maior que 65 anos ou pacientes com idade entre 45-64 anos com doença cardiovascular estabelecida ou fator de risco * para doença cardiovascular submetidos a operações não cardíacas.	**I**
**Troponinas cardíacas e monitorização de complicações cardiovasculares**		
Pós-operatório em unidade de terapia intensiva (UTI) por 48 horas em pacientes de risco alto de complicações segundo algoritmos, submetidos a cirurgias não cardíacas de risco intermediário ou alto.		**I**
Pós-operatório em UTI por 48 horas em pacientes de risco intermediário de complicações segundo algoritmos, submetidos a cirurgias não cardíacas de risco intermediário ou alto.		**IIa**
**Diagnóstico e tratamento das complicações cardiovasculares no perioperatório**		
Injúria/infarto agudo do miocárdio perioperatório (PMI)		
O diagnóstico de PMI deve ser feito na presença de um delta absoluto ≥ ao percentil 99 do valor superior de referência do kit de troponina utilizado entre os valores de troponina pré-operatório e do primeiro ou segundo pós-operatório. Na ausência do valor basal, o delta pode ser considerado entre dois valores pós-operatórios.		**I**
O diagnóstico do infarto agudo do miocárdio (IAM) após o segundo dia pós-operatório deve ser feito baseado na definição universal do IAM, e seu tratamento deve ser baseado nas diretrizes atuais.		**I**
Em pacientes com IAM perioperatório ou PMI devido a causas isquêmicas, a otimização das causas secundárias de isquemia (anemia, taquicardia, hipotensão, hipertensão) deve ser realizada, assim como a avaliação de risco de sangramento e discussão multidisciplinar com o cirurgião.		**I**
**Fibrilação/ *flutter* atrial agudos**		
A manutenção da anticoagulação em longo prazo deve ser considerada em pacientes com fibrilação atrial (FA) detectada após cirurgia não cardíaca, quando analisado risco de acidente vascular cerebral, conforme CHA_2_DS_2_VASc e risco de sangramento, de acordo com a cirurgia realizada.		**IIa**
**Tromboembolismo venoso (TEV)**		
Em pacientes com instabilidade hemodinâmica, deve-se optar por anticoagulação parenteral com heparina não fracionada (HNF) em detrimento de heparina de baixo peso molecular (HBPM) ou fondaparinux.		**I**
Em pacientes com TEV e indicação de anticoagulação parenteral, deve-se preferir HBPM ou fondaparinux à HNF.		**I**

* Fatores de risco: Diabetes, hipertensão, doença arterial coronária, obesidade, fibrilação atrial.

### 1.4. Criação do *Perioperative Risk Team*

Esta Diretriz tem foco principal, mas não exclusivo, no cardiologista, em função do fato de que as complicações mais graves são as que envolvem o sistema CV, como a cardiopatia isquêmica, o tromboembolismo pulmonar e a insuficiência cardíaca (IC) descompensada. São essas complicações que estão mais relacionadas com mortalidade, aumento do período de internação hospitalar e maior custo, não só pela realização procedimentos adicionais, como pela solicitação de exames diagnósticos. Os pacientes que entram em nossas salas cirúrgicas atualmente são mais idosos e consequentemente têm mais comorbidades, envolvendo, além do cirurgião, anestesista e cardiologista, outros especialistas no seu tratamento. O acompanhamento dos pacientes de maior risco deve ser interdisciplinar, desde o planejamento adequado para determinação do melhor momento para a intervenção, a relação risco/benefício até a otimização do tratamento das doenças de base e suas complicações.

Os cardiologistas já estão familiarizados com o *Heart Team* , geralmente formado pelo cirurgião cardíaco, o hemodinamicista e o próprio cardiologista clínico, que costumam atuar no pré-operatório decidindo qual a melhor abordagem para aquele caso. No ambiente perioperatório de intervenções cirúrgicas não cardíacas, o número de especialistas de diferentes áreas de atuação é maior, além de incluir todo o período intra e pós-operatório. Esta Diretriz de Avaliação Cardiovascular Perioperatória da Sociedade Brasileira de Cardiologia – 2024 inova ao propor a criação de um *perioperative risk team* (PRT). O objetivo do PRT, indicado para casos mais graves, é reunir todas as informações relativas ao paciente, como doenças e tratamentos prévios, prognóstico, tipo de cirurgia proposta e condição cardiológica atual. Com harmonia e o trabalho realizado em equipe pelos diversos especialistas, será possível oferecer a melhor decisão conjunta para o paciente e sua família. Mais uma vez pelo fato de que as complicações mais graves são as que envolvem o sistema CV, o cardiologista deve indicar, quando julgar necessário, para casos graves e específicos, a formação do PRT e estabelecer a sua dinâmica. Ferramentas como plataformas de vídeo, através de gravação de reuniões interdisciplinares devidamente armazenadas, podem ser de grande utilidade para melhorar o resultado e dar garantias para os profissionais envolvidos e para o paciente.

## 2. Avaliação Pré-operatória Geral e Suplementar

### 2.1. Estratificação do Risco Pré-operatório

#### 2.1.1. Condições Cardiovasculares Graves/Instáveis no Perioperatório

Em casos de operações eletivas, o primeiro passo é a verificação das condições clínicas basais do paciente. Existem circunstâncias clínicas em que o risco espontâneo de complicações é muito alto, independentemente do procedimento cirúrgico a ser adotado. A identificação desse cenário é fundamental, pois a estabilização de tais condições deve ter prioridade em relação à operação eletiva, que deve, sempre que possível, ser adiada, sendo reconsiderada somente após a compensação clínica ( Tabela 2 ).

**Tabela 2:** Condições cardiovasculares graves/instáveis no perioperatório

Síndrome coronariana aguda
Doenças instáveis da aorta torácica
Edema agudo dos pulmões
Choque cardiogênico
Insuficiência cardíaca classe funcional III/IV da NYHA*
Angina classe funcional III/IV da CCS *
Estenose aórtica/mitral importante sintomática
Bradiarritmias ou taquiarritmias graves (BAVT, TV)
Fibrilação atrial de alta resposta ventricular (FC > 120 bpm)
Hipertensão arterial sistêmica não controlada (PA > 180 x 110 mmHg)
Hipertensão arterial pulmonar sintomática

* Pacientes com estas condições que se encontram estáveis e com tratamento já otimizado devem ter a relação risco versus benefício da intervenção cirúrgica analisada em virtude do risco de complicações. NYHA: New York Heart Association; CCS: Canadian Cardiovascular Society; BAVT: bloqueio atrioventricular total; TV: taquicardia ventricular; PA: pressão arterial; FC: frequência cardíaca.

#### 2.1.2. Estimativa do Risco Intrínseco Relacionado ao Tipo de Operação

O risco intrínseco da operação é determinado pelo tipo e pela sua duração, sem levar em conta as características clínicas do paciente. É definido como a probabilidade de ocorrência de eventos CV perioperatórios, independentemente das variáveis clínicas dos pacientes. Ele está relacionado à duração, ao estresse hemodinâmico e à perda de sangue e fluidos que ocorrem durante a intervenção. Os pacientes com condições clínicas estáveis, que não apresentam condições cardíacas de alto risco, podem ser encaminhados para a realização de procedimentos com baixo risco intrínseco sem necessidade de avaliação adicional. Apesar da dificuldade de determinar um risco específico para procedimentos cirúrgicos, já que ocorrem em diferentes circunstâncias, a Sociedade Europeia de Cardiologia propôs uma classificação que leva em consideração o risco da ocorrência de morte CV, infarto agudo do miocárdio (IAM) e acidente vascular cerebral (AVC) em 30 dias ( Tabela 3 ).

**Tabela 3:** Classificação do risco cardiovascular de acordo com o tipo de operação

Risco baixo (<1%)	Risco intermediário (1-5%)	Risco alto (>5%)
Mama	Carótida assintomática	Aorta e vascular *major*
Procedimentos dentários	Endarterectomia carótida (sintomática)	Revascularização periférica aberta devido à isquemia aguda ou amputação
Tireoide	Angioplastia arterial periférica	Angioplastia carótida (sintomática)
Ocular	Aneurisma de aorta endovascular	Ressecção adrenal
Ginecológica *minor*	Cabeça e pescoço	Pancreática
Ortopédica *minor* (menisco)	Intraperitoneal: colecistectomia, hérnia hiatal, esplenectomia	Fígado e vias biliares
Reconstrutiva	Intratorácica *non-major*	Esofagectomia
Superficia	Neurológica ou ortopédica *major* (espinal, quadril)	Pneumectomia (VATS ou aberta)
Urológica *minor* (RTU)	Transplante renal	Transplante pulmonar
VATS *minor*	Urológica e ginecológica *major*	Transplante hepático
		Cistectomia total
		Reparo de perfuração intestinal

Adaptada de Halvorsen et al.^3^ RTU: ressecção transuretral de próstata; VATS: cirurgia torácica por videotoracoscopia.

Além disso, o grau de urgência da intervenção cirúrgica deve ser sempre levado em consideração. As operações de urgência e emergência estão associadas a maior ocorrência de complicações CV. Em situações em que o prognóstico da doença de base que levou à indicação cirúrgica demande a intervenção de **emergência** , o papel do cardiologista deve-se restringir a sugerir medidas de monitorização (incluindo o local para o pós-operatório) e intervenções para redução do risco no intra e pós-operatório, não sendo recomendado indicar nenhum exame complementar que atrase a cirurgia proposta. No caso de cirurgias de **urgência** , existe tempo hábil para a otimização da terapêutica CV ou a realização de exames complementares como ecocardiograma transtorácico, quando indicado. Por outro lado, a solicitação de provas funcionais para avaliação de isquemia miocárdica não deve ser feita, uma vez que seu resultado não mudará a conduta porque a cirurgia proposta não poderá ser adiada para o tratamento coronariano. Além disso, existem as cirurgias **tempo-dependentes** , isto é, não são urgências, porém o atraso na realização da operação pode causar uma piora no prognóstico da doença de base. Um exemplo usual são as cirurgias oncológicas, que, se adiadas, podem comprometer o prognóstico oncológico. Nesses casos, recomendamos a discussão multidisciplinar e a escolha da melhor estratégia individualizada.

#### 2.1.3. Avaliação da Capacidade Funcional

Pacientes com baixa capacidade funcional (menos de quatro equivalentes metabólicos [METs] ou incapacidade de subir dois lances de escadas) apresentam maior chance de complicações perioperatórias.^4^ A capacidade funcional pode ser aferida de maneira objetiva, pelo teste ergométrico (o que nem sempre é possível ou desejável), ou por meio da história clínica. Um estudo recente demonstrou que pacientes que referem ser capazes de subir dois lances de escadas na história clínica durante a consulta de avaliação pré-operatória apresentaram uma taxa menor de eventos CV pós-operatórios.^5^

Além disso, a estimativa da capacidade funcional associada ao Índice de Risco Cardíaco Revisado (RCRI, de *Revised Cardiac Risk Index* ) apresentou maior acurácia na predição de eventos pós-operatórios quando comparada à utilização do RCRI isoladamente.^5^ Além da maior probabilidade de má evolução perioperatória, pacientes com baixa capacidade funcional podem ter seus sintomas subestimados em virtude da limitação física. Esse aspecto, portanto, pode ser considerado na decisão de solicitação de exames complementares, como provas funcionais para detecção de isquemia.

O Quadro 1 apresenta a recomendação para avaliação da capacidade funcional no pré-operatório.

**Quadro 1:** Recomendação para avaliação da capacidade funcional no pré-operatório

Recomendação	Classe de recomendação	Nível de evidência
Determinar a capacidade funcional de pacientes em programação de operações de risco intermediário ou alto durante a anamnese (baseada na habilidade de subir dois lances de escada).	I	B

#### 2.1.4. Ferramentas para Estimar o Risco Cardíaco Perioperatório

A impressão subjetiva sobre o risco cardíaco perioperatório deve ser valorizada, mas estimá-lo objetivamente permite a utilização racional de recursos complementares de estratificação de risco e de assistência CV perioperatória. Além disso, o cálculo do risco cardíaco fundamenta a discussão multidisciplinar para definição de propostas que tenham por objetivo oferecer o menor risco global para o paciente.

Esta Diretriz não preconiza a adoção de um algoritmo específico de estratificação do risco cardíaco perioperatório, mas recomenda que, após excluir condições cardíacas graves/instáveis ( Tabela 2 ), seja feito o cálculo do risco conforme um dos índices disponíveis na literatura, conforme a preferência do avaliador.

Entre os índices para estimativa do risco cardíaco perioperatório, destacam-se, pela praticidade e acurácia na discriminação de diferentes classes de risco, o RCRI, ou escore de Lee,^6^ que é um dos mais utilizados na prática clínica e na literatura de medicina perioperatória, e o mais recentemente publicado índice AUB (American University of Beirut)-HAS2.^7^ As características clínicas e o tipo de cirurgia proposta integram a avaliação em ambos os modelos, e não há diferente ponderação entre as variáveis para cada um dos índices ( Tabelas 4 e 5 ). De acordo com o número de variáveis, foram observadas taxas crescentes de complicações CV nas coortes pivotais do RCRI e do AUB-HAS2.

**Tabela 4:** Variáveis dos índices de risco: RCRI^6^ e AUB-HAS2^7^

RCRI	AUB-HAS2
História de doença coronária *	Histórico de doença cardíaca **
História de insuficiência cardíaca	Sintomas da doença cardíaca: angina ou dispneia
História de doença cerebrovascular	Idade ≥ 75 anos
Creatinina > 2,0 mg/dL	Hemoglobina < 12 g/dL
Cirurgia intraperitoneal, intratorácica ou vascular suprainguinal	Cirurgia vascular arterial
Diabetes com insulinoterapia	Cirurgia de emergência

* No RCRI, os critérios para doença coronária são história de infarto agudo do miocárdio, prova funcional positiva, presença de angina, uso de nitrato ou onda Q no eletrocardiograma.

** No AUB-HAS2, os critérios para histórico de doença cardíaca são antecedente de infarto do miocárdio, revascularização miocárdica, insuficiência cardíaca, fibrilação atrial ou valvopatia moderada a severa confirmada por ecocardiograma. RCRI: Índice de Risco Cardíaco Revisado; AUB: American University of Beirut.

**Tabela 5:** Classificação do risco de acordo com o número de variáveis de risco presentes nas coortes de derivação e validação do RCRI6 e do AUB-HAS2^7^

RCRI	AUB-HAS2	
Nenhuma	Nenhuma	
Uma	Uma	
Duas	Duas	
Três a seis	Três	
	Quatro a seis	
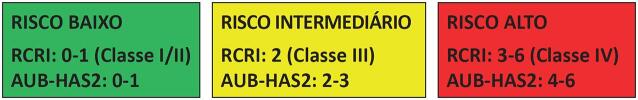	

6 RCRI: Índice de Risco Cardíaco Revisado; AUB: American University of Beirut.

Ainda não há evidência na população brasileira de superioridade de um algoritmo em relação aos demais, e cabe ressaltar que, embora os dois índices estimem desfechos cardiológicos graves, a definição de desfecho é diferente entre eles. O RCRI, já validado em nossa população,^8^ estima o risco da ocorrência de IAM, edema agudo dos pulmões, bloqueio atrioventricular (BAV) total e parada cardiorrespiratória nos primeiros 30 dias após o procedimento cirúrgico.^6^ Já o AUB-HAS2, ainda não validado na nossa população, estima o risco de IAM, acidente vascular encefálico ou óbito também nos primeiros 30 dias após a operação.^7^

Com relação ao algoritmo de risco CV pré-operatório RCRI, na sua versão antiga presente na última edição desta Diretriz,^2^ os valores de risco de eventos CV no pós-operatório eram de 0,4% para a Classe I (nenhuma variável de risco), 0,9% para a Classe II (uma variável), 7% para Classe III (duas variáveis) e 11% para a Classe IV (três ou mais variáveis). Outros algoritmos que estimam o risco de complicações no período perioperatório também incluíram valores numéricos percentuais que representam as taxas observadas nos estudos de referência. A partir de estudos mais recentes, entretanto, encontrou-se que essas estimativas de risco estariam desatualizadas e seriam mais altas, em virtude provavelmente da maior gravidade dos pacientes operados. De fato, como foi publicado na última diretriz europeia de avaliação perioperatória,^3^ os números foram 4% para Classe I, 6% para Classe II, 10% para Classe III e 15% para Classe IV.

Por outro lado, como esses números variam muito conforme o país e a população, os autores da nova edição desta Diretriz optaram por uma classificação semi-quantitativa, estabelecendo como risco baixo (Classes I e II), intermediário (Classes III) e alto (Classe IV) nos relatórios de avaliação de risco pré-operatório, não especificando valores absolutos.

Entre as limitações dos índices, destaca-se a possível perda de acurácia em pacientes com baixa capacidade funcional. Esses pacientes têm pior prognóstico perioperatório^9^ e podem ser assintomáticos simplesmente por não atingirem limiar para deflagrar sintomas. Por esse motivo, mesmo que o paciente com baixa capacidade funcional receba estimativa de risco cardíaco baixo ou intermediário pelos índices, a avaliação pode ser complementada com testes para doença coronária e IC antes de cirurgias de alto risco intrínseco, notadamente quando o paciente tem fatores de risco para doença CV (vide Item 2.4). Outra questão é a perda de acurácia dos índices para cirurgias específicas, como é o caso do RCRI, que perde a habilidade de discriminar classes de risco e subestima eventos em pacientes submetidos a operações vasculares, notadamente na correção de aneurisma de aorta abdominal.^6 , 10 - 12^ Especificamente para operações vasculares arteriais, esta Diretriz recomenda a utilização do índice proposto pelo *Vascular Study Group of New England* (VSG): VSG *Cardiac Risk Index* (VSG-CRI) ( Tabelas 6 e 7 ).^11^ Quando comparado ao RCRI, o VSG-CRI mostrou melhor acurácia na predição de IAM, arritmia clinicamente relevante ou IC no pós-operatório vascular arterial.^11^ O índice AUB-HAS2 mantém boa acurácia em discriminar quatro classes de risco progressivo também no perioperatório vascular arterial, com taxas absolutas de complicação perioperatória mais elevadas que aquelas observadas para o conjunto de todas as operações.^13 , 14^

**Tabela 6:** Variáveis clínicas e respectivas pontuações no índice *Vascular Study Group of New England Cardiac Risk Index* (VSG-CRI)^11^

Fatores de risco VSG-CRI		PONTOS
Idade		
	≥ 80 anos	4
	70-79 anos	3
	60-69 anos	2
Doença arterial coronariana		2
Insuficiência cardíaca		2
Doença pulmonar obstrutiva crônica		2
Creatinina > 1,8 mg/dL		2
Tabagismo		1
Diabetes com uso de insulina		1
Uso crônico de betabloqueador		1
Revascularização miocárdica prévia		-1

**Tabela 7:** Classes de risco conforme o índice *Vascular Study Group of New England Cardiac Risk Index* (VSG-CRI)^11^

Pontuação VSG-CRI	Classe de risco
0-3	Baixo
4	Baixo
5	Intermediário
6	Intermediário
7	Alto
≥ 8	Alto

Outros índices de risco validados para avaliação cardíaca perioperatória são o índice de risco do American College of Physicians^15 , 16^ e o índice do Estudo Multicêntrico de Avaliação Perioperatória (EMAPO),^8^ este último desenvolvido e validado no Brasil. Quando o objetivo for a análise da estimativa de risco global, não relacionado apenas a desfechos de morbimortalidade CV, pode-se utilizar a ferramenta desenvolvida pelo American College of Surgeons (ACS), conhecida como ACS NSQIP® *Surgical Risk Calculator* (www.riskcalculator.facs.org).^17^ Essa ferramenta foi desenvolvida a partir de base de dados de mais de 1 milhão de operações realizadas nos Estados Unidos e contempla, além do tipo específico de procedimento cirúrgico a ser adotado, 21 variáveis clínicas, fornecendo estimativa de risco de oito desfechos diferentes. A maior limitação dessa ferramenta é que ela não é rápida ou facilmente aplicável, requer a utilização de calculadora e considera algumas variáveis de determinação subjetiva.

A escolha do índice de risco CV perioperatório deve levar em conta a experiência do avaliador e a natureza vascular, ou não vascular, do procedimento cirúrgico proposto. Cabe ressaltar que o risco de tromboembolismo venoso (TEV), altamente prevalente e prevenível no perioperatório, não está contemplado nos índices discutidos nessa seção e merece abordagem dedicada.^2^

O fluxograma para a avaliação perioperatória pode ser visualizado na Figura Central.

#### 2.1.5. Avalição da Fragilidade

A síndrome de fragilidade é uma entidade clínica multidimensional relacionada à idade, conceitualmente definida como a redução da reserva fisiológica em diversos sistemas do organismo, levando ao estado de maior vulnerabilidade a agentes estressores, como doenças agudas e procedimentos cirúrgicos. A fragilidade se associa com risco aumentado de mortalidade e diversos outros desfechos adversos, como internação hospitalar, menor capacidade funcional e pior qualidade de vida.^18^ Nas últimas décadas, houve aumento da atenção global sobre a avaliação de fragilidade em diferentes cenários clínicos, refletindo o fenômeno crescente de envelhecimento populacional em todo o mundo.^19 , 20^ No Brasil, a prevalência de fragilidade é de 9% entre pessoas com 50 anos ou mais que vivem na comunidade e 16% quando considerada a faixa etária de 65 anos ou mais. Essas prevalências assemelham-se às de países desenvolvidos e apresentam valores mais altos quando a população estudada se encontra em ambientes de saúde, como clínicas e hospitais.^21^ Apesar da falta de consenso sobre um método padrão-ouro que defina fragilidade, o Fenótipo de Fragilidade Física e o Índice de Fragilidade são consideradas as medidas de maior validade para identificar essa síndrome.^22^ Embora esteja associada com multimorbidade, envelhecimento e limitações para atividades básicas e instrumentais de vida diária, a síndrome de fragilidade é considerada uma entidade distinta e separada desses outros fatores.^22 - 24^ O espectro de gravidade da síndrome de fragilidade engloba desde o paciente robusto e pré-frágil até o propriamente frágil.

Embora seja evidente que a doença CV pode causar fragilidade pela hospitalização por eventos agudos, por causar imobilidade e limitar a capacidade física de indivíduos com doença estabelecida (por exemplo, hospitalização por síndrome coronariana aguda [SCA], limitação física por sintomas de IC),^25^ há plausibilidade biológica para que existam processos fisiopatológicos subjacentes comuns nas duas condições, indicando uma relação bidirecional.^26^ A fragilidade se associa a uma série de alterações fisiológicas e funcionais, incluindo inflamação crônica, disfunção endotelial, disautonomia, ativação do sistema renina-angiotensina-aldosterona e estresse oxidativo, que são fatores de risco para doenças CV.^27^ De fato, em uma coorte de pacientes sem doença CV,^28^ a presença de fragilidade foi um fator de risco para a incidência de eventos CV em um seguimento de 6 anos. Outros estudos também observaram uma associação independente entre fragilidade e desfechos CV adversos em uma variedade de cenários de condições CV.^29^

##### 2.1.5.1. Como Avaliar Fragilidade Antes de Cirurgia Não Cardíaca

Apesar da falta de consenso sobre um único instrumento para rastreio de fragilidade em idosos tanto em nível populacional quanto em situações específicas, como na avaliação perioperatória de cirurgia não cardíaca, recomenda-se que a fragilidade seja avaliada de forma objetiva pelo uso de instrumentos previamente validados. A Tabela 8 apresenta alguns exemplos de instrumentos que podem ser utilizados para avaliação de fragilidade. Ressalta-se que existem outros instrumentos que podem ser utilizados além dos ilustrados na Tabela 8 . A recomendação do uso de instrumentos baseia-se no fato de que a impressão subjetiva dos médicos, principalmente os profissionais não especializados em saúde do idoso, pode ser influenciada por fatores de confusão, como idade avançada, presença de múltiplas doenças, baixo peso e uso de dispositivos de marcha, os quais podem levar à suposição errônea da presença de fragilidade quando esta não está presente de fato.^30 - 32^ A avaliação da fragilidade com o uso de instrumentos validados também permite a obtenção de informação mais detalhada sobre o *status* do paciente, em vez da simples dicotomização nas categorias frágil e não frágil, o que pode ser simplista e superficial.

**Tabela 8:** Exemplos de instrumentos disponíveis para avaliação de fragilidade

Instrumento	Tipo de avaliação	Componentes	Definição de fragilidade	Link ou aplicativo para utilização do instrumento
Fenótipo de Fragilidade Física^31^	Informações autorrelatadas e testes de performance física para avaliar cinco critérios fenotípicos	Inatividade física Lentificação da marcha Perda de peso Fraqueza muscular Exaustão	Frágil: ≥ 3 Pré-frágil: 1-2 Robusto: 0	https://www.johnshopkitnssolutions.com/solution/frailty/
*Clinical Frailty Scale* (CFS)^32^	Saúde global e capacidade funcional	Escala com nove níveis, de 1 ( *very fit* ) a 9 ( *terminally ill* )	Frágil: ≥ 5 Pré-frágil: 4 Robusto: 1-3	https://www.acutefrailtynetwork.org.uk/Clinical-Frailty-Scale/Clinical-Frailty-Scale-App
*Essential Frailty Toolset* (EFT)^33^	Combinação de desempenho físico e cognitivo com exames complementares	Teste de sentar-se e levantar-se da cadeira Teste cognitivo Albumina sérica Hemoglobina	Frágil: ≥ 3 Pré-frágil: 1-2 Robusto: 0	*Frailty Tool* : aplicativo disponível para iOS e Android
Índice de Fragilidade por acúmulo de déficits^34^	Multidimensional pelo modelo de acúmulo de déficits	Avaliação de pelo menos 40 itens que devem: – Estar associados com saúde – Englobar vários sistemas – Ter prevalência > 1% na população de interesse – Aumentar a prevalência com o avançar da idade	Escore contínuo entre 0 (ausência de déficits) a 1 (déficit total), que indica a proporção de alterações encontradas entre os itens avaliados Em geral, um escore > 0,25 é utilizado para definir a presença de fragilidade	https://www.bidmc.org/research/research-by-department/medicine/gerontology/calculator
Escala FRAIL^35^	Questionário conciso e autorrelatado sobre cinco itens de saúde	Fadiga Resistência Deambulação Número de condições crônicas Perda de peso	Frágil: ≥ 3 Pré-frágil: 1-2 Robusto: 0	https://www.sciencedirect.com/science/article/pii/S1279770723014987?via=ihub-cesec100 https://www.ncbi.nlm.nih.gov/pmc/articles/PMC4515112/#APP1

A fragilidade também pode ser avaliada e classificada a partir de diferentes domínios de saúde: físico, cognitivo, psicossocial e nutricional. Dependendo da ferramenta utilizada para definir a fragilidade, um ou mais domínios são avaliados, o que permite caracterizar os pacientes em fenótipos clínicos distintos.^22^

##### 2.5.1.2. Impacto da Avaliação de Fragilidade em Cirurgia Não Cardíaca

A fragilidade é um preditor de vários desfechos perioperatórios adversos em várias especialidades cirúrgicas, tanto em procedimentos eletivos como de urgência/emergência. As evidências indicam que pacientes frágeis apresentam maior risco de complicações clínicas no período pós-operatório, como infecções, *delirium* , insuficiência renal aguda, arritmias cardíacas e IAM, bem como problemas relacionados à cicatrização da ferida cirúrgica, como deiscência e hérnias. Como consequência, os pacientes frágeis permanecem por período mais prolongado no hospital. Essas complicações pós-operatórias têm impacto negativo na recuperação funcional e na qualidade de vida dos pacientes frágeis que foram submetidos à cirurgia, e isso pode levar à maior necessidade de transferência para instituições de longa permanência e à maior mortalidade tanto em curto quanto em longo prazo em comparação com os pacientes não frágeis.^33 - 43^ Apesar disso, o impacto da inclusão da avaliação de fragilidade na performance prognóstica dos escores de risco tradicionais (RCRI, VSG-CRI, AUB-HAS2, entre outros) ainda é incerto, devido ao número limitado de estudos nessa área.^44 , 45^ A combinação através de outros métodos estatísticos ainda precisa ser explorada para determinar seu impacto e utilidade clínica.^46^

Apesar das limitações existentes, entende-se que a avaliação perioperatória de fragilidade pode: (1) auxiliar no processo de tomada de decisão por prover um dimensionamento mais completo dos riscos envolvidos com o procedimento cirúrgico, o que melhora o diálogo entre o paciente, a família e os diferentes profissionais envolvidos nos cuidados perioperatórios (anestesista, cirurgião, clínico, cardiologista, geriatra); (2) identificar um subgrupo de pacientes com maior risco de complicações nos quais intervenções precoces, incluindo no período pré-operatório possam ser implementadas (por exemplo, reabilitação física e nutricional, correção de anemia e medidas para prevenção e identificação precoce de *delirium* ).

O Quadro 2 apresenta as recomendações para avaliação de fragilidade no perioperatório de cirurgia não cardíaca.

**Quadro 2:** Recomendações para avaliação de fragilidade no perioperatório de cirurgia não cardíaca

Recomendação	Classe de recomendação	Nível de evidência
A fragilidade deve ser avaliada rotineiramente em idosos submetidos a cirurgias de risco intermediário ou alto.	IIa	B
A fragilidade deve ser mensurada objetivamente através de instrumento específico.	IIa	C

### 2.2. Eletrocardiograma

O eletrocardiograma (ECG) pode detectar arritmias, distúrbios de condução, isquemia miocárdica ou IAM prévio, sobrecargas ventriculares e alterações decorrentes de distúrbios eletrolíticos ou de efeitos de medicamentos. Além disso, um traçado eletrocardiográfico basal é importante para a avaliação comparativa no perioperatório em pacientes com alto risco de ocorrência de eventos CV.

Entretanto, a aplicação de rotina de um teste com especificidade limitada pode levar à ocorrência de resultados falso-positivos em pacientes assintomáticos, uma vez que alterações eletrocardiográficas costumam ser causa de preocupação da equipe cirúrgica e anestésica e, muitas vezes, podem levar ao cancelamento desnecessário da operação.^47^ Estima-se que cerca de 50% dos indivíduos acima de 40 anos possuem alguma alteração eletrocardiográfica.^48^ As anormalidades encontradas no ECG tendem a aumentar com a idade e a presença de comorbidades, mas apresentam baixo poder preditivo de complicações.^49 - 51^

Em um estudo retrospectivo com mais de 23.000 pacientes, a presença de alterações eletrocardiográficas pré-operatórias foi associada à maior incidência de mortes de causa cardíaca em 30 dias.^52^ Esse resultado foi corroborado em dois estudos prospectivos posteriores, que encontraram resultados semelhantes, de que anormalidades no ECG pré-operatório foram preditoras de eventos CV perioperatórios.^53 , 54^ Em um estudo retrospectivo, a presença de intervalo QT corrigido entre 480 ms e 519 ms foi um preditor independente de mortalidade após operações não cardíacas.^55^ No grupo de pacientes submetidos à cirurgia de risco baixo a moderado, entretanto, o ECG pré-operatório apresentou informação prognóstica limitada. Portanto, a interpretação do ECG como fator prognóstico depende da história clínica do paciente, assim como do seu risco CV.

**Figura 1:**
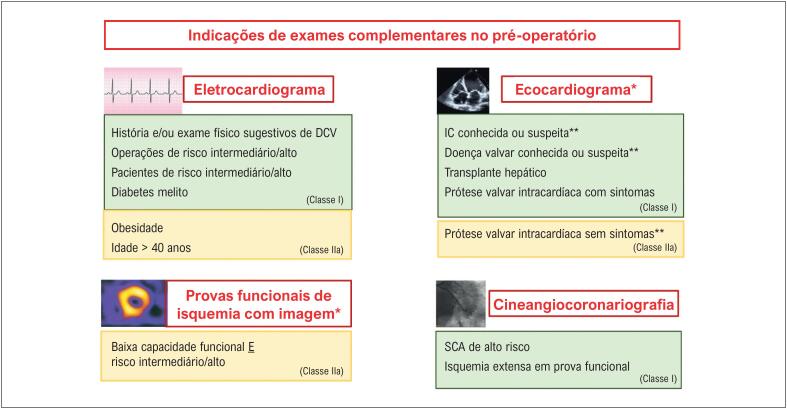
Indicações de exames cardíacos complementares no pré-operatório. *Somente indicado em pacientes em programação de operações de risco intermediário ou alto. **Sem avaliação no último ano (ou 6 meses em casos de doença valvar). DCV: doença cardiovascular; IC: insuficiência cardíaca; SCA: síndromes coronarianas agudas.

Dessa forma, o principal papel do ECG pré-operatório é fornecer um traçado basal para comparação no caso da suspeita de um evento CV pós-operatório. A indicação de ECG pré-operatório deve ser criteriosa conforme história clínica, tipo de cirurgia e doenças apresentadas ( Figura 1 ).

O Quadro 3 apresenta as recomendações para a solicitação de ECG.

**Quadro 3:** Recomendações para solicitação de ECG

Recomendação	Classe de recomendação	Nível de evidência
História e/ou anormalidades ao exame físico sugestivas de doença cardiovascular	I	C
Pacientes submetidos a operações de risco intrínseco de complicações cardiovasculares intermediário ou alto	I	C
Pacientes de risco intermediário ou alto para eventos cardiovasculares perioperatórios estimados pelos algoritmos	I	B
Presença de diabetes melito	I	C
Obesos	IIa	C
Idade superior a 40 anos	IIa	C

### 2.3. Ecocardiograma

O ecocardiograma é um método diagnóstico não invasivo de baixo custo com extensa aplicação em vários campos da Cardiologia. Oferece uma ampla avaliação morfofuncional CV, o que é fundamental nas decisões diagnósticas e terapêuticas pré-operatórias. Com relação à morfologia e à anatomia, o tamanho das cavidades, a massa ventricular e a avaliação estrutural das valvas e grandes vasos são dados básicos avaliados em uma rotina ecocardiográfica, além das funções sistólica e diastólica. A avaliação hemodinâmica advém de cálculos matemáticos que usam dados obtidos pela ferramenta Doppler, e o mapeamento de fluxo em cores avalia de forma acurada a dinâmica de fluxos intracardíacos e transvalvares. Alterações cardíacas morfofuncionais podem estar intimamente correlacionadas ao aumento do risco perioperatório.

Além da técnica ecocardiográfica transtorácica básica, existem múltiplas outras modalidades que podem ser realizadas. Entre elas, há a ecocardiografia transesofágica, técnica semi-invasiva que possibilita minucioso detalhamento anatômico, pesquisa de trombos intracavitários, avaliação da aorta, entre outros. Outras técnicas são a mensuração da deformação ( *strain* ) miocárdica para uma avaliação mais pormenorizada da função contrátil ventricular, o uso de agentes de realce (microbolhas) para avaliação de perfusão miocárdica e a ecocardiografia tridimensional, que possibilita uma avaliação volumétrica mais acurada, com determinação da fração de ejeção do ventrículo esquerdo (FEVE).

Contudo, vale ressaltar que o ecocardiograma de repouso no pré-operatório de cirurgia não cardíaca não é um exame a ser realizado rotineiramente, devendo ser solicitado somente quando, após uma avaliação clínica básica inicial, houver suspeita de cardiopatia. Essa avaliação inicial envolve anamnese e exame físico minuciosos e eventualmente exames clínicos básicos como os laboratoriais, ECG e radiografia de tórax.^56 , 57^

A ecocardiografia transtorácica é o principal método diagnóstico em pacientes com IC suspeita ou conhecida, e suas múltiplas modalidades ajudam a estimar o risco cirúrgico.^58 - 60^ No entanto, o seu emprego deve ser criterioso, pois ainda não há evidências de que o seu uso esteja associado ao aumento da sobrevida ou a menor permanência hospitalar. Pelo contrário, vários estudos sugerem que seu uso mais liberal aumente o tempo de hospitalização, sem benefício clínico.^61^ Por fim, nos pacientes com valvopatia conhecida ou suspeita, próteses e dispositivos intracardíacos, a ecocardiografia transtorácica ou transesofágica deve ser utilizada para auxiliar a determinar o risco perioperatório, como também para orientar a profilaxia para endocardite infecciosa (EI) ( Figura 1 ).

O Quadro 4 apresenta as recomendações para a realização do ecocardiograma pré-operatório.

**Quadro 4:** Recomendações para realização do ecocardiograma pré-operatório

Recomendação	Classe de recomendação	Nível de evidência
Pacientes com insuficiência cardíaca ou sintomas sugestivos e que serão submetidos à cirurgia de risco intermediário ou alto, sem avaliação no último ano, ou que apresentaram piora clínica	I	B
Pacientes portadores ou com suspeita de alteração anatômica valvar moderada/importante e que serão submetidos à cirurgia de risco intermediário ou alto, sem avaliação nos últimos 6 a 12 meses, ou que apresentaram piora clínica	I	C
Pacientes que serão submetidos a transplante hepático	I	B
Pacientes portadores de prótese valvar intracardíaca, que serão submetidos à cirurgia de risco intermediário ou alto e sintomáticos	I	C
Pacientes portadores de prótese valvar intracardíaca, que serão submetidos à cirurgia de risco intermediário ou alto sem avaliação no último ano	IIa	C
Pacientes assintomáticos que serão submetidos à cirurgia de alto risco (vide Tabela 3 )	IIb	C
Rotina em indivíduos assintomáticos, sem suspeita clínica de insuficiência cardíaca ou doença valvar moderada a grave submetidos à cirurgia de risco intermediário ou baixo	III	C

### 2.4. Testes Não Invasivos para Detecção de Isquemia Miocárdica

#### 2.4.1. Eletrocardiograma de Esforço

O teste ergométrico (TE) é uma ferramenta segura, útil e eficaz para a detecção de isquemia miocárdica,^62^ tendo justamente como mecanismo produzir uma disparidade entre oferta e demanda. Assim, é razoável pensar que a detecção de anormalidades durante a sua execução possa ser reprodutível durante o perioperatório e seus variados níveis de estresse. Porém, não há evidências robustas de que usar essa estratégia resulte na redução da mortalidade perioperatória.^63^

Considerando que o objetivo da estratificação do risco é reduzir o risco perioperatório, não seria lógica a realização do exame em indivíduos já estratificados como de baixo risco pelos algoritmos recomendados. Nesse cenário, com baixa prevalência da doença arterial coronariana (DAC), o TE não agregaria valor à estratificação clínica perioperatória, tendo em vista que quanto menor a prevalência da DAC em determinada população, menor será também o valor preditivo positivo do TE. Este poderia, inclusive, retardar a realização da cirurgia em razão da sua execução e devido à necessidade da realização de outros exames mais específicos para a diferenciação dos resultados verdadeiros dos falsos-positivos.^64 , 65^

Mesmo em indivíduos de alto risco, como no pré-operatório de cirurgia vascular, são baixos o valor preditivo positivo, a sensibilidade e a especificidade do TE (10, 74 e 69%, respectivamente), ainda que o valor preditivo negativo seja elevado (98%).^66^ Por outro lado, em um estudo de coorte, a realização de testes provocativos de isquemia no pré-operatório em pacientes de alto risco e com três ou mais fatores de risco clínicos esteve associada a menor tempo de internação hospitalar e a menor mortalidade hospitalar em 1 ano.^64^ Dessa forma, entre os indivíduos assintomáticos com maior prevalência da doença, o TE poderia ser solicitado somente quando o resultado fosse influenciar no prognóstico e, consequentemente, na conduta pré-operatória, quer para guiar uma terapêutica clínica mais intensiva ou mesmo para um procedimento de revascularização miocárdica. Nesse cenário, a baixa tolerância ao exercício (inferior a 4 METs) e o início da resposta isquêmica em baixa carga estão associados a maior número de eventos cardíacos perioperatórios.^9^

O Quadro 5 apresenta as recomendações para a realização do teste ergométrico no pré-operatório.

**Quadro 5:** Recomendações para a realização do teste ergométrico no pré-operatório

Recomendação	Classe de recomendação	Nível de evidência
Solicitação de teste ergométrico em pacientes com baixa capacidade funcional, de risco intermediário ou alto, em programação de cirurgia não cardíaca eletiva de risco intermediário ou alto em que o teste poderá potencialmente alterar a conduta	IIb	C
Pacientes que serão submetidos a cirurgia de baixo risco	III	C
Pacientes com baixo risco de complicações e que serão submetidos a cirurgia de risco baixo ou intermediário	III	C

#### 2.4.2. Cintilografia de Perfusão Miocárdica com Estresse

A avaliação complementar com TE, apesar da maior disponibilidade e menor custo, apresenta limitações principalmente em pacientes com alta probabilidade pré-teste de DAC, naqueles com alterações basais no traçado do ECG ou que possuam limitações para realizar atividade física em esteira.^62^

Dessa forma, o uso de prova funcional com método de imagem associada a estresse físico ou farmacológico apresenta maior acurácia na avaliação de risco de eventos CV perioperatórios, podendo ser útil na estratificação de risco adicional de pacientes com baixa capacidade funcional, DAC estabelecida ou alta probabilidade pré-teste sem diagnóstico prévio de síndrome coronariana crônica.

No cenário perioperatório, existe uma boa correlação entre o grau de lesão coronariana e a presença de defeitos de perfusão miocárdica, com maior capacidade preditiva da prova funcional nos pacientes com doença mais extensa.^67^ Uma metanálise avaliando 1.179 pacientes submetidos a cirurgias vasculares arteriais revelou maior taxa de eventos perioperatórios com grandes defeitos de perfusão miocárdica (> 20%) com área sob a curva (AUC) de 0,78 (intervalo de confiança de 95% [IC95%], 0,65-0,89) para o método. Vale ressaltar que, mesmo no estudo com população de alto risco (doença vascular estabelecida), somente 23% dos indivíduos apresentaram isquemia de grande extensão.^68^

Em pacientes com boa capacidade funcional (≥ 4 METs), o uso de provas funcionais com imagem acrescentou pouco quando adicionado ao RCRI (AUC 0,77 vs. AUC 0,70).^69^ Outro fato que deve ser ponderado ao se considerar a solicitação de uma prova funcional são estudos mais recentes (fora do contexto perioperatório) demostrando benefício do tratamento clinico frente à estratégia invasiva mesmo em pacientes com grandes áreas isquêmicas.^70^

Por fim, apesar da utilidade no diagnóstico de DAC e na estratificação de risco de eventos, a solicitação de provas funcionais exige cautela no contexto perioperatório, uma vez que pode desencadear uma propensão a revascularização coronariana antes da cirurgia não cardíaca, estratégia que não demostrou superioridade em ensaios clínicos randomizados e ainda pode atrasar a cirurgia não cardíaca durante o tempo mínimo de dupla antiagregação plaquetária (DAP).^71^ Mesmo em pacientes com alta carga isquêmica em prova funcional listados para transplante renal, uma estratégia invasiva não demonstrou superioridade frente ao tratamento clínico otimizado.^72^

#### 2.4.3. Ecocardiograma de Estresse com Dobutamina

A ecocardiografia sob estresse é precisa e segura em identificar pacientes com DAC, com importante papel como preditor de eventos cardíacos.^73 , 74^ O estresse pode ser realizado através de esforço físico, na esteira ergométrica ou cicloergômetro (bicicleta), ou por uso de fármacos, principalmente dobutamina e dipiridamol associados à atropina caso não haja contraindicações. As modalidades de estresse pela dobutamina ou pelo exercício apresentam acurácia diagnóstica semelhantes, superiores ao dipiridamol.^75^

A ecocardiografia sob estresse farmacológico pela dobutamina ou dipiridamol tem reconhecidamente um grande valor para avaliação diagnóstica e prognóstica. A alteração de contração segmentar envolvendo uma grande extensão do VE, surgindo em estágios precoces de infusão do fármaco, além de firmar o diagnóstico de insuficiência coronariana, confere ao paciente um alto risco de complicações isquêmicas em curto prazo, aumentando significantemente o risco CV perioperatório. Por sua vez, a ausência de alteração de contração segmentar no estresse tem uma boa correlação com a ausência de obstruções coronarianas críticas. Ainda, o aparecimento dessa alteração em uma pequena extensão miocárdica, no estágio tardio de pico do estresse, também se traduz em melhor prognóstico e menor risco de eventos CV. Caso um ecocardiograma sob estresse pela dobutamina não demonstre isquemia residual no paciente com infarto prévio, o prognóstico é bom, e a probabilidade de reinfarto, morte e edema agudo pulmonar é baixa no período transoperatório de uma cirurgia não cardíaca.^66^ O uso da ecocardiografia sob estresse pela dobutamina na avaliação do risco perioperatório já está bem documentado na literatura, apresentando valor preditivo positivo variando de 25 a 55% e valor preditivo negativo de 93 a 100% para eventos cardíacos em pacientes submetidos a cirurgia não cardíaca.^66 , 76^ Os resultados geralmente são utilizados para influenciar a conduta clínica pré-operatória, especialmente a decisão de realização de cineangiocoronariografia com angioplastia ou cirurgia de revascularização miocárdica antes ou após a cirurgia eletiva. Em uma metanálise de 15 estudos que compararam a cintilografia de perfusão miocárdica com a ecocardiografia sob estresse pela dobutamina na estratificação de risco CV pré-operatório, foi demonstrado que o valor prognóstico das anormalidades em ambas as modalidades de imagens para eventos isquêmicos perioperatórios é semelhante.^77^

Por fim, o uso de agentes de realce com microbolhas melhora a visibilidade dos bordos endocárdicos do VE, aumentando a sensibilidade do exame para a detecção de alterações na contração segmentar. Adicionalmente, é possível agregar a avaliação de perfusão do miocárdio em tempo real. Déficits de perfusão detectados em uma parede com isquemia se correlacionam com pior prognóstico, tendo maior chance de eventos a curto prazo, o que aumenta o risco CV perioperatório.

#### 2.4.4. Sumário de Recomendações para a Realização dos Testes Não Invasivos ( Figura 1 )

O Quadro 6 apresenta as recomendações para a solicitação de cintilografia com estresse/ecocardiograma com estresse no pré-operatório.

**Quadro 6:** Recomendações para a solicitação de cintilografia com estresse/ecocardiograma com estresse no pré-operatório

Recomendação	Classe de Recomendação	Nível de Evidência
Solicitação de provas funcionais com imagem em pacientes com baixa capacidade funcional, de risco intermediário ou alto, em programação de cirurgia não cardíaca eletiva de risco intermediário ou alto em que a prova funcional poderá potencialmente alterar a conduta	IIa	B
Solicitação de provas funcionais em pacientes com baixa capacidade funcional, assintomáticos, com diagnóstico prévio ou alta probabilidade de doença arterial coronariana	IIb	B
Solicitação de provas funcionais de rotina em pacientes de baixo risco e/ou que serão submetidos a procedimentos de baixo risco intrínseco	III	C

### 2.5. Cineangiocoronariografia

A avaliação coronariana invasiva através da cineangiocoronariografia não é recomendada rotineiramente antes de cirurgias não cardíacas e não deve substituir os testes não invasivos para avaliação de isquemia miocárdica, quando indicados. A realização desnecessária da cineangiocoronariografia, além de potencialmente atrasar o planejamento cirúrgico, não apresenta evidências de aumento na sobrevida dos pacientes ou de diminuição do risco de infarto periprocedimento, mesmo naqueles submetidos a cirurgias com alto risco de complicações CV.^71 , 78^

As indicações para realizar avaliação coronariana invasiva são similares àquelas fora do contexto de avaliação pré-operatória, como pacientes com SCA ou com isquemia extensa em prova funcional ( Figura 1 ).^79^ Em um pequeno estudo randomizado, os autores avaliaram a realização rotineira de cineangiocoronariografia no pré-operatório de endarterectomia de carótidas. Apesar do menor número de eventos isquêmicos nos pacientes submetidos à angioplastia pré-operatória, não houve diferença na mortalidade. Além disso, a cirurgia foi realizada em média 4 dias após o procedimento, o que não é recomendável nem seguro.^80 , 81^

Especialmente procedimentos tempo-dependentes, como cirurgias oncológicas, não devem ser postergados para avaliação invasiva em pacientes assintomáticos. Essa recomendação é baseada em estudos que falharam em mostrar qualquer redução de complicações cardíacas pós-operatórias em pacientes assintomáticos submetidos à revascularização coronariana pré-operatória.^82^

O Quadro 7 apresenta as recomendações para a realização de cineangiocoronariografia no pré-operatório.

**Quadro 7:** Recomendações para a realização de cineangiocoronariografia no pré-operatório

Recomendação	Classe de Recomendação	Nível de Evidência
Pacientes com síndromes coronarianas agudas de alto risco	I	A
Pacientes com isquemia extensa em prova funcional	I	B
Pacientes estáveis submetidos a cirurgias de baixo risco	III	C

### 2.6. Angiotomografia de Coronárias

A angiotomografia (angio-TC) de coronárias apresenta alta sensibilidade para a detecção anatômica de estenoses coronarianas, incluindo doença multiarterial e lesão em tronco de coronária esquerda.^83^ Entretanto, os benefícios da angio-TC antes de cirurgias não cardíacas ainda são incertos.

O benefício do estudo da anatomia coronária pela angio-TC foi avaliado por Li et al., que estudaram 841 idosos com indicação de cirurgia não cardíaca de alto risco sem DAC conhecida ou suspeita. A identificação de DAC significativa de um vaso foi de 12,2%, de dois vasos, 5,4% e de três vasos, de 1,9%. Um escore Agatston acima de 195 foi independentemente associado com maior risco de DAC. DAC significativa foi encontrada em 19,5% dos pacientes. A análise multivariada identificou o grau de estenose como fator independente para o cancelamento de cirurgia agendada. Os autores consideraram a angio-TC coronária útil para descartar ou confirmar DAC significativa.^84^

O uso da angio-TC como exame adicional ao escore de risco RCRI foi estudado por Ahn et al.^85^, que avaliaram estudos retrospectivos em pacientes com indicação de procedimentos de porte intermediário. A presença de lesões maiores que 50% aumentavam a incidência de eventos cardiovasculares adversos maiores (MACE), alcançando até 29,7% na DAC multiarterial *versus* 4,3% na sua ausência. Os autores concluem que esse exame poderia ser vantajoso na reclassificação do risco. Em um estudo prospectivo com 955 pacientes, Sheth et al. demonstraram que o uso de angio-TC no pré-operatório pode melhorar a estimativa de risco de eventos nos pacientes que tiverem eventos (morte CV ou infarto). Entretanto, a reclassificação de risco utilizando angio-TC comparada com o escore de risco RCRI ocorreu em 22% dos pacientes estudados e pode superestimar inapropriadamente em até 5 vezes o risco de complicações.^86^

A extensão e a gravidade da DAC encontrada pela angio-TC em relação a incidência de MACE foram avaliadas em uma metanálise de 11 estudos.^87^ Observou-se que a gravidade e a extensão da DAC estiveram associadas ao aumento do risco de MACE (ausência de DAC 2%, DAC não obstrutiva 4,1%, DAC obstrutiva um vaso 7,1%, DAC multiarterial 23,1%). A DAC multiarterial apresentou o maior risco (razão de chances [OR] 8,9). O aumento do escore de cálcio também foi associado ao maior risco perioperatório (escore cálcio ≥ 100 OR 5,1, ≥ 1000 OR 10,4 ambos p < 0,001).

A angio-TC foi diretamente comparada com a ecocardiografia de estresse pela dobutamina em relação a sua acurácia prognóstica no ensaio PANDA.^88^ Foram incluídos 215 pacientes com mais de um fator de risco submetidos às duas metodologias. O ecocardiograma com estresse apresentou OR de 6,1 para eventos, a presença de DAC significativa pela angio-TC apresentou OR de 18,8, e a presença de escore de cálcio elevado apresentou OR de 4,2. Os autores concluem que a angio-TC pode apresentar um melhor valor prognóstico em relação ao ecocardiograma com estresse antes de cirurgia não cardíaca, mas o tamanho amostral desse estudo era muito pequeno para uma conclusão definitiva.

Na ausência de mais estudos sem evidências que comprovem redução de eventos com uma estratégia guiada pela angio-TC e, principalmente, pelo receio da geração excessiva de angioplastias "preventivas", a angio-TC não é recomendada rotineiramente para a estratificação de risco antes de cirurgia não cardíaca.

## 3. Doenças e Procedimentos com Aspectos Específicos no Perioperatório

### 3.1. Insuficiência Cardíaca

A IC afeta em torno de 1 a 2% da população geral nos países desenvolvidos, cerca de 5,7 milhões de pacientes nos Estados Unidos e > 10% da população maior que 70 anos.^89 , 90^ As doenças do aparelho circulatório são as principais causas de morte no Brasil, sendo responsáveis por aproximadamente 29% das mortes ocorridas no país. As doenças isquêmicas do coração e a IC são responsáveis por aproximadamente 39% dessas mortes por doenças do aparelho circulatório.^91^

A IC é fator de risco bem conhecido para eventos cardíacos perioperatórios. Dados de um grande registro de cirurgias não cardíacas que incluiu mais de 150.000 procedimentos revelou que a presença de IC esteve associada com aumento de 63% no risco de mortalidade perioperatória e de 51% no risco de reinternação em 30 dias quando comparado ao grupo com DAC sem IC.^92^

Recentemente, uma avaliação da base de dados dos Estados Unidos envolvendo 21.560.996 cirurgias não cardíacas entre 2012 e 2014 revelou a presença de IC perioperatória em 4,9% dos casos e esteve associada a maior taxa de mortalidade intra-hospitalar (4,8% *versus* 0,8%), conferindo aumento de risco de 2,2 vezes. O risco foi maior entre os pacientes com IC aguda do que entre os pacientes com IC crônica; o risco foi maior entre os pacientes com IC crônica descompensada em comparação com IC crônica isolada (7,8% *versus* 3,8%, p < 0,001).^93^

Em outro estudo de coorte retrospectiva envolvendo 609.735 pacientes submetidos à cirurgia não cardíaca, a mortalidade em 90 dias foi maior entre os pacientes com IC sintomática (10,1%), seguido por pacientes com IC assintomática (4,9%), enquanto, em pacientes sem IC, a mortalidade foi de 1,2%. Assim, a presença de IC, sintomática ou não, aumenta o risco de morte precoce.^94^

A FEVE reduzida é considerada forte preditor de eventos em pacientes submetidos à cirurgia vascular. No entanto, a maioria dos estudos analisou a FEVE dicotomizando em maior ou menor que 40%. Um estudo envolvendo 174 pacientes com IC revelou que apenas a FEVE gravemente reduzida (< 30%) foi preditor independente de mortalidade. A presença de redução da FEVE moderada (30-40%) ou leve (40-50%) ou, ainda, a IC de FEVE preservada (> 50%) não foram preditores independentes de morte em 30 dias.^95^ Apesar do poder preditor de eventos da FEVE, a realização rotineira de ecocardiograma para todos os pacientes a serem submetidos a cirurgia cardíaca não é indicada. Um estudo de coorte canadense envolvendo mais de 250.000 pacientes (15% com ecocardiograma pré-operatório, n = 40.084) revelou que esse exame não está associado à melhora da sobrevida ou à redução do tempo de hospitalização após cirurgia não cardíaca de grande porte.^61^

A elevação de peptídeos natriuréticos no pré-operatório está relacionada a pior prognóstico no período perioperatório, uma vez que está associada à piora da função ventricular e a maior taxa de eventos CV.^96 , 97^ A dosagem desses biomarcadores pode auxiliar na estratificação de risco de pacientes com IC.

O manejo clínico de pacientes com IC no período perioperatório exige alguns cuidados especiais relacionados à volemia do paciente. Deve-se evitar tanto a hipovolemia, que pode intensificar hipotensão, quanto a hipervolemia, que pode gerar congestão pulmonar e sistêmica. O estudo austríaco IMPROVE pretende determinar se o uso de um inodilatador, levosimendana, pode ter algum benefício na evolução pós-operatória, avaliando o impacto do uso da medicação nos níveis de peptídeos natriuréticos. Seus resultados podem trazer uma nova forma de abordagem perioperatória para pacientes com IC submetidos a cirurgias não cardíacas.^98^

**Figura 2:**
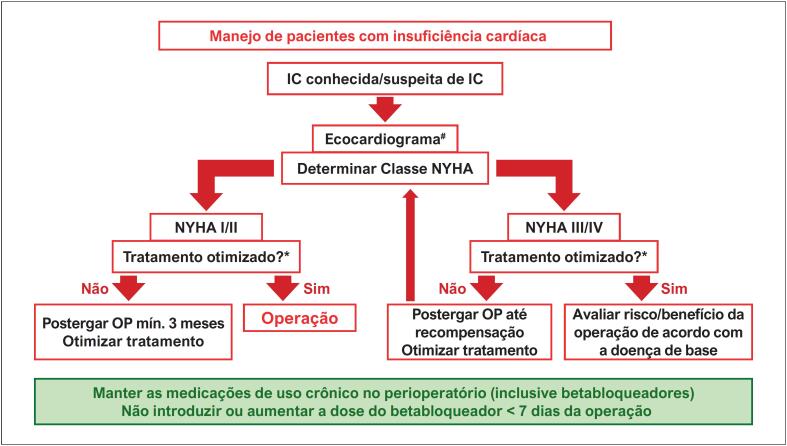
Abordagem pré-operatória de pacientes com insuficiência cardíaca ou suspeita de insuficiência cardíaca. IC: insuficiência cardíaca; NYHA: New York Heart Association; OP: operação; mín.: mínimo. ^#^Em casos de pacientes com ecocardiograma disponível no último ano e sem novos sintomas, não é necessário repetir o ecocardiograma. *Tratamento otimizado da IC segundo diretrizes atuais (máxima dose tolerada de inibidores da enzima de conversão da angiotensina/ bloqueadores dos receptores da angiotensina/sacubitril/valsartan, betabloqueadores, espironolactona e inibidores do cotransportador de sódio-glicose tipo 2 [SGLT2] para IC com fração de ejeção reduzida e adequado controle da volemia e pressão arterial, espironolactona e inibidores SGLT2 para pacientes com IC e fração de ejeção preservada), avaliação e correção de isquemia miocárdica, em caso de doença valvar importante, correção da doença valvar.

O fluxograma para avaliação pré-operatória de pacientes com IC conhecida ou suspeita pode ser consultado na Figura 2 .

O Quadro 8 apresenta as recomendações para o manejo perioperatório de pacientes com IC.

**Quadro 8:** Recomendações para o manejo perioperatório de pacientes com insuficiência cardíaca (IC)

Recomendação	Classe de recomendação	Nível de evidência
Cirurgias eletivas em pacientes com IC descompensada (classe funcional III/IV segundo NYHA) devem ser postergadas até a compensação clínica do paciente	I	C
Cirurgias eletivas em pacientes com IC de início recente, cujo tratamento ainda não foi otimizado, devem ser postergadas no mínimo 3 meses para permitir o uso das medicações em doses adequadas	I	C
Todas as medicações de uso crônico devem ser mantidas no período perioperatório e reintroduzidas o mais precocemente possível no pós-operatório. Na impossibilidade de administração de medicação por via oral, deve-se considerar a administração por sonda nasoenteral ou via venosa	I	C
O uso de betabloqueador deve ser mantido no perioperatório, porém a introdução de altas doses em pacientes que não faziam uso previamente não é recomendada, a menos que haja tempo suficiente para ajuste de dose antes da realização da cirurgia	I	C

NYHA: New York Heart Association.

### 3.2. Hipertensão Arterial

A hipertensão arterial sistêmica é uma condição clínica de elevada prevalência, tanto na população geral quanto em pacientes que serão submetidos a cirurgias, sendo responsável por cerca de 50% das mortes por DAC e AVC.^99^ Apesar de bem estabelecida a importância de seu controle rigoroso na prevenção de eventos CV a longo prazo, seu manejo no período perioperatório é fonte de ampla discussão, uma vez que ainda possui aspectos controversos.

#### 3.2.1. Manejo Pressórico no Pré-operatório

O diagnóstico prévio de hipertensão é preditor independente de mortalidade em pacientes submetidos a cirurgias não cardíacas.^100^ Por outro lado, não há evidência concreta da existência de relação entre a medida de pressão arterial no momento da admissão hospitalar e complicações cardíacas perioperatórias,^101^ sugerindo, portanto, que o risco perioperatório atribuído à hipertensão é, na verdade, decorrente majoritariamente de suas lesões de órgão-alvo.^102^

Em relação à hipotensão, a literatura traz dados mais concretos. Uma coorte prospectiva de 251.567 pacientes demonstrou que níveis de pressão arterial sistólica (PAS) menores que 119 mmHg e diastólica (PAD) menores que 63 mmHg associam-se a aumento de mortalidade no período pós-operatório de até 30 dias.^103^ É necessário, portanto, que o manejo da pressão arterial considere esse provável efeito em "J", em que níveis pressóricos baixos também representam risco.

Com relação à hipertensão, recomenda-se que cirurgias eletivas de alto risco sejam adiadas apenas se a PAS for maior ou igual a 180 mmHg e/ou a PAD maior ou igual a 110 mmHg. É importante reforçar que esse limite pressórico foi estipulado com base em dados observacionais e antigos^104^, definido em um contexto em que ainda não se sabia dos agora conhecidos riscos da hipotensão no período perioperatório. Assim sendo, é adequado que se mantenha o alvo pressórico definido, mas com o cuidado de evitar ao máximo a hipotensão.

Por fim, pacientes com suspeita de hipertensão arterial secundária devem ser investigados antes da cirurgia. Embora não existam evidências robustas sobre o aumento do risco perioperatório em portadores de hipertensão secundária, sabe-se que aqueles com feocromocitoma não diagnosticado apresentam mortalidade cirúrgica muito elevada.^105^

#### 3.2.2. Manejo Pressórico no Intraoperatório

No intraoperatório, nas raras situações em que é necessária a introdução do anti-hipertensivo, o anti-hipertensivo ideal deve ser facilmente titulado e ter rápido início de ação, poucos efeitos colaterais e baixo custo. Diversas classes estão disponíveis para uso, incluindo os betabloqueadores (esmolol e labetalol), bloqueadores dos canais de cálcio (nicardipina) e nitratos (nitroprussiato de sódio e nitroglicerina).

Pacientes com hipertensão arterial também são mais suscetíveis à hipotensão, na maior parte das vezes associada à depleção do volume intravascular.^106^ A preocupação excessiva frente ao controle pressórico rigoroso no período pré-operatório pode ser causa de hipotensão tanto durante quanto após a cirurgia, sendo particularmente associada a injúria cardíaca, renal e cerebral e maior mortalidade.^107^

Nesse cenário, o estudo INPRESS ( *Effect of Individualized vs Standard Blood Pressure Management Strategies on Postoperative Organ Dysfunction Among High-Risk Patients Undergoing Major Surgery* ) comparou uma estratégia individualizada que visava manter a PAS dentro de 10% do valor em repouso *versus* uma estratégia padrão, em que os pacientes recebiam vasoconstritor apenas se a PAS fosse inferior a 80 mmHg ou a 40% do repouso. Observou-se, no primeiro grupo, redução de síndrome da resposta inflamatória sistêmica (SIRS) e de disfunção orgânica 7 dias após a cirurgia.^108^ Uma análise retrospectiva envolvendo pacientes submetidos a cirurgias não cardíacas eletivas reforçou o efeito deletério da hipotensão no perioperatório.^109^

##### 3.2.3. Manejo Pressórico no Pós-operatório

O diagnóstico prévio de hipertensão arterial é o principal fator de risco para a ocorrência de hipertensão no período pós-operatório, mas outros elementos como dor, hipercapnia e excitação ao despertar da anestesia também contribuem para tanto. A terapia medicamentosa com anti-hipertensivos por via venosa deve ser considerada para pacientes com PAS sustentada maior ou igual a 180 mmHg e/ou PAD maior ou igual a 110 mmHg, por aumentar o risco de sangramento, em especial em cirurgias cardíacas, vasculares e endoscópicas, como ressecção transuretral de próstata. A hipotensão também deve ser devidamente tratada e prevenida, mantendo o paciente com volemia preservada.

O Quadro 9 apresenta as recomendações para o manejo pressórico no perioperatório.

**Quadro 9:** Recomendações para o manejo pressórico no perioperatório

Recomendação	Grau de recomendação	Nível de evidência
Cirurgias eletivas de alto risco devem ser adiadas se a pressão arterial sistólica for maior ou igual a 180 mmHg e/ou a diastólica for maior ou igual a 110 mmHg	I	C
A otimização da volemia (evitar desidratação) deve ser realizada durante todo o perioperatório	I	C
Deve-se evitar, durante todo o período perioperatório, episódios de hipotensão	I	B
Pacientes com suspeita de hipertensão arterial secundária devem ser investigados antes da cirurgia, salvo em casos de urgência/emergência	I	C
O reinício da terapêutica anti-hipertensiva no pós-operatório, de preferência a que o paciente utilizava antes da cirurgia, deve ser realizado o mais rapidamente possível	I	C

##### 3.2.4. Anti-hipertensivos no Perioperatório

###### 3.2.4.1. Antagonistas do Sistema Renina-Angiotensina-Aldosterona

O bloqueio do sistema renina-angiotensina-aldosterona é bastante controverso no contexto perioperatório, uma vez que estudos demonstraram resultados conflitantes. Grandes análises retrospectivas que avaliaram a suspensão *versus* a manutenção de inibidores da enzima conversora de angiotensina (IECA) e bloqueadores do receptor de angiotensina II (BRA) no perioperatório não evidenciaram diferença entre os grupos na incidência de hipotensão, na necessidade de ressuscitação volêmica e na mortalidade em 30 dias.^110^ Além disso, sua descontinuação no dia da cirurgia não se associou a aumento significativo da incidência de hipertensão imediatamente antes da intervenção, bem como não foi responsável por uma maior taxa de cancelamento de cirurgias.^111^

Entretanto, Roshanov et al. observaram decréscimo no risco de hipotensão intraoperatória e redução de desfecho composto por morte, IAM e acidente vascular encefálico com a suspensão da medicação.^112^ Apesar da possível maior incidência de hipotensão com a manutenção do IECA/BRA, não foi evidenciado aumento de lesão renal aguda.^113^ Vale ressaltar ainda que, em casos em que o BRA foi suspenso e não reintroduzido dentro de 2 dias após a cirurgia, houve aumento de mortalidade.^114^

Dessa forma, as evidências atuais não fornecem uma resposta definitiva quanto à manutenção ou suspensão do IECA/BRA no período perioperatório, devendo a decisão ser individualizada. O estudo randomizado em andamento STOP-or-NOT poderá trazer evidências adicionais para esse tópico.^115^ Para tanto, deve-se levar em conta o tipo de cirurgia, a variabilidade pressórica do paciente e a previsão de sangramento. É importante frisar que, caso a medicação seja suspensa, ela deve, idealmente, ser reintroduzida o quanto antes, assim que o paciente tenha condições clínicas para tal.

###### 3.2.4.2. Bloqueadores do Canal de Cálcio

Em pacientes submetidos a cirurgias não cardíacas, não foi demonstrada diferença significativa de valores pressóricos com o uso de bloqueadores de canal de cálcio quando comparado com outras classes anti-hipertensivas, e seu uso não esteve associado ao aumento de eventos adversos.^116^ Diante disso, ainda que a recomendação formal seja de manter os bloqueadores de canal de cálcio durante o perioperatório naqueles pacientes que já façam uso crônico, tal decisão deve ser individualizada, tendo em vista os riscos já descritos em relação à hipotensão e à ausência de efeitos deletérios de sua suspensão.

###### 3.2.4.3. Diuréticos

Não há evidências robustas a respeito do uso de diuréticos no período perioperatório. Sua manutenção, incluindo a administração no dia do procedimento cirúrgico, mostrou-se segura e não se associou a aumento da incidência de hipotensão ou necessidade de reposição de fluidos e drogas vasopressoras, bem como a aumento de eventos CV no pós-operatório.^117^

###### 3.2.4.4. Simpatolíticos de Ação Central

Os primeiros estudos que avaliaram o uso de clonidina no perioperatório de cirurgias não cardíacas demonstraram redução de isquemia miocárdica;^118^ porém, estudos maiores e mais recentes falharam em mostrar tal associação, sendo seu uso relacionado a um aumento da incidência de parada cardíaca não fatal, principalmente em assistolia ou atividade elétrica sem pulso. Seu uso está associado também a um aumento significativo de bradicardia e hipotensão, sendo a hipotensão preditora independente de IAM.^119^ Dessa forma, a clonidina pode ser utilizada em caso de pressão arterial descontrolada sem tempo hábil para seu controle efetivo, mas não deve ser introduzida com o objetivo de redução de eventos CV. Sua suspensão em usuários crônicos não é recomendada pela possibilidade de efeito rebote.

##### 3.2.5. Considerações Finais

O manejo da hipertensão arterial durante o perioperatório é bastante desafiador. Nesse contexto, ainda que seja importante evitar níveis pressóricos extremamente elevados (PAS > 180 mmHg e PAD > 110 mmHg), é fundamental que se evite a hipotensão, que se apresenta nos estudos mais recentes e robustos como fator de mau prognóstico e confere aumento de risco de mortalidade. Diante disso, excetuando-se os betabloqueadores e os simpatolíticos de ação central, cuja suspensão pode ser deletéria, o manejo dos anti-hipertensivos no perioperatório deve ser individualizado para que se evite picos pressóricos muito elevados, labilidade pressórica e, principalmente, hipotensão. No período perioperatório, a avaliação clínica individualizada deve incluir o monitoramento dos valores de pressão arterial e, principalmente, o surgimento de manifestações relacionadas à hipotensão como tontura, desmaio, sonolência, sinais de baixo débito, deterioração da função renal, redução do débito urinário ou *delirium* .

O Quadro 10 apresenta as recomendações para o manejo dos anti-hipertensivos no perioperatório.

**Quadro 10:** Recomendações para o manejo dos anti-hipertensivos no perioperatório

Recomendação	Grau de recomendação	Nível de evidência
Pacientes em uso crônico de antagonistas do sistema renina-angiotensina-aldosterona podem ter a medicação mantida no perioperatório, sendo a suspensão permitida em casos selecionados	I	C
Pacientes em uso crônico de bloqueadores de canal de cálcio podem ter a medicação mantida no perioperatório, sendo a suspensão permitida em casos selecionados	I	C
Pacientes em uso crônico de diuréticos podem ter a medicação mantida no perioperatório, sendo a suspensão permitida em casos selecionados	I	C
Pacientes em uso crônico de clonidina devem ter a medicação mantida no perioperatório	I	B

### 3.3. Conduta em Procedimentos de Baixo Risco

A denominação "procedimentos de baixo risco" utilizada neste texto se refere exclusivamente ao baixo risco de complicações CV e de sangramento. Os autores desta Diretriz entendem que há procedimentos de baixo risco cirúrgico em todas as especialidades médicas, mas as considerações específicas do ponto de vista cirúrgico fogem aos objetivos deste texto.

#### 3.3.1. Odontológicos

Manter a saúde bucal adequada é fundamental para redução de risco de complicações sistêmicas especialmente em pacientes com doenças CV, resultando, por exemplo, em melhor controle glicêmico, em redução de riscos de bacteremia e em possível melhor controle da pressão arterial.^120 - 122^ Doenças bucais, como doenças periodontais e infecções endodônticas, podem trazer riscos de bacteremia e sepse no pós-operatório com intubação e em receptores de transplantes,^123^ devendo ser tratadas antes dos procedimentos cirúrgicos.^124^

O uso de 15 mL de solução de clorexidina 0,2% (mais efetiva) ou iodopovidona (5%) para bochecho de 30 a 60 segundos é recomendado antes do procedimento odontológico, assim como no pré-operatório de operações de risco intermediário ou alto, visando à redução da ocorrência de sepse.^125 , 126^

##### 3.3.1.1. Agentes Antitrombóticos

Quando os pacientes que recebem agentes antitrombóticos, incluindo antiplaquetários e anticoagulantes, são submetidos a tratamento odontológico cirúrgico, a decisão deve ser tomada sobre continuar a terapia de anticoagulação e o risco de complicações hemorrágicas ou interromper brevemente a anticoagulação e aumentar o risco de complicações embólicas. Resultados de décadas de estudos de milhares de pacientes odontológicos recebendo terapia de anticoagulação revelam que as complicações hemorrágicas que requerem mais do que medidas locais para hemostasia têm sido raras e nunca fatais. No entanto, algumas complicações embólicas foram fatais, e outras complicações debilitantes ocorreram em pacientes cuja anticoagulação foi interrompida para procedimentos odontológicos.^127^

A maioria dos procedimentos odontológicos tem baixo risco de sangramento, apresentando taxa de hemorragia de até 3,63%.^128^ Além disso, há fortes evidências, no caso dos medicamentos mais antigos (ou seja, varfarina, agentes antiplaquetários) e evidências limitadas para medicamentos anticoagulantes orais mais recentes de que, para a maioria dos pacientes, a ocorrência de sangramento grande e não controlável é muito baixa. Por essas razões, esta Diretriz não recomenda alterar ou interromper a anticoagulação ou a terapia antiplaquetária antes da intervenção odontológica.^129 , 130^

Em geral, procedimentos odontológicos podem ser realizados com segurança em pacientes anticoagulados com varfarina e razão normalizada internacional (RNI) < 3,5, desde que medidas locais para redução do sangramento sejam aplicadas. Para pacientes com RNI acima dessa faixa ou para aqueles em que se antecipa maior risco de sangramento, deve-se discutir a melhor estratégia a ser adotada entre o dentista e o responsável pela prescrição do anticoagulante.^131^

O manejo do sangramento na prática odontológica pode ser feito através de análogos de lisina (ácido tranexâmico 250 mg, 15 a 25 mg/kg, isto é, dois comprimidos de 250 mg, duas a três vezes ao dia), esponja de fibrina e cianoacrilatos utilizados localmente, entre outros.

##### 3.3.1.2. Dispositivos Cardíacos Eletrônicos Implantáveis

Durante os procedimentos dentários em pacientes portadores de dispositivos cardíacos eletrônicos implantáveis (DCEI), além do risco de infecção nos dispositivos eletrônicos, há a possibilidade de interferência do equipamento odontológico no DCEI, particularmente do fotopolimerizador a bateria, do raspador dentário e de limpadores dentários ultrassônicos, quando a distância entre eles é menor que 23 cm.^132^ Nessas situações, outros equipamentos odontológicos devem ser empregados.

Porém, outros equipamentos eletrônicos odontológicos como escovas elétricas de dentes, bisturi elétrico, testador elétrico de polpa, canetas de alta e baixa rotação, localizadores de ápice e amalgamadores não mostraram interferência.^132 , 133^

##### 3.3.1.3. Anestésicos Locais

O uso excessivo de anestésicos locais com vasoconstritores em doses excessivas pode aumentar a frequência cardíaca, a pressão arterial e a subsequente demanda miocárdica de oxigênio. Entretanto, estudos recentes confirmam que o uso de 1 a 4 ampolas de anestésicos com vasoconstritor lidocaína com epinefrina 1:80000, 1:100000 e 1:200000 é relativamente seguro para pacientes com doenças CV e hipertensão controladas.^134 , 135^

O Quadro 11 apresenta o sumário das recomendações odontológicas.

**Quadro 11:** Sumário das recomendações odontológicas

Recomendação	Grau de recomendação	Nível de evidência
Avaliação e tratamento odontológico são importantes no perioperatório	IIa	B
Uso de solução antimicrobiana oral antes e após os procedimentos odontológicos e cirurgias não cardíacas de risco intermediário e alto	IIa	B
Uso de anestésicos com 1-4 ampolas de vasoconstritor é seguro para tratamento dentário de pacientes cardiopatas	IIa	C
Manter tratamento antiplaquetário e anticoagulante na maioria dos tratamentos odontológicos, incluindo extração de até dois dentes, restaurações, próteses, endodontia, limpezas e implantes	IIa	B

#### 3.3.2. Dermatológicos

Os procedimentos cirúrgicos dermatológicos apresentam baixo risco, tanto para eventos CV quanto para sangramento. Dados da literatura sugerem que aproximadamente 50% dos pacientes que se apresentam para procedimentos dermatológicos estão em uso de terapia antiagregante ou anticoagulante.^136 , 137^ Nesses casos, a equipe cirúrgica e o anestesiologista devem ser informados sobre os medicamentos em uso e sobre os cuidados necessários, incluindo a hemostasia mais demorada e cuidadosa, já que, na maioria das vezes, o risco associado à suspensão da terapia antitrombótica supera o de sangramento inerente ao procedimento.

Em pacientes que fazem uso de ácido acetilsalicílico (AAS) para prevenção secundária de eventos CV, não se indica a suspensão antes da realização de qualquer intervenção cirúrgica dermatológica.^138 , 139^

Em pacientes que fazem uso de monoterapia com clopidogrel, o risco de sangramento durante cirurgias é aumentado, e algumas evidências sugerem, inclusive, suspender a medicação pelo menos 6 dias antes.^140 , 141^ No entanto, não há estudos que demonstrem a segurança da suspensão em termos de evento CV. Embora a tendência seja extrapolar as evidências da monoterapia com AAS para o clopidogrel, não há evidências suficientes que suportem a manutenção ou a suspensão. Nos casos de cirurgia dermatológica, como o sangramento costuma ser pequeno e controlado, recomendamos não suspender.

Em pacientes que fazem uso de DAP indicada pelo uso de *stent* coronariano e que estão fora do período de maior risco trombótico, a recomendação é suspender o segundo antiplaquetário,^142 , 143^ respeitando-se os intervalos já descritos nesta Diretriz (consultar seção de antiagregantes plaquetários, no Item 4.1.3 desta Diretriz).^144^

Para indivíduos em uso de varfarina, a recomendação é que ela não seja descontinuada e que a RNI seja ajustada para valores ≤ 3,5 para minimizar o risco de sangramento. Apesar disso, alguns trabalhos não demonstraram correlação entre o nível da RNI e o risco de sangramento aumentado nos pacientes em uso de varfarina.^142 , 145 - 148^

Para pacientes em uso de anticoagulantes orais diretos (DOACs), ainda que as evidências sejam escassas, recomenda-se a sua manutenção para a maioria dos procedimentos dermatológicos,^142 , 149 , 150^ tomando-se o cuidado para que a intervenção cirúrgica seja marcada, quando possível, algumas poucas horas antes da próxima dose, evitando, assim, o pico do nível sérico da droga.

O Quadro 12 apresenta as recomendações para pacientes em programação de procedimentos dermatológicos.

**Quadro 12:** Recomendações para pacientes em programação de procedimentos dermatológicos

Recomendação	Classe de recomendação	Nível de evidência
O ácido acetilsalicílico deve ser mantido em pacientes em prevenção secundária de eventos cardiovasculares submetidos a qualquer intervenção cirúrgica dermatológica	I	B
Para pacientes que fazem uso de dupla antiagregação plaquetária por stent e apresentam-se fora do período de maior risco trombótico, manter o ácido acetilsalicílico e suspender o segundo antiplaquetário	I	C
O clopidogrel (em monoterapia) pode ser mantido em pacientes em prevenção secundária de eventos cardiovasculares submetidos a intervenções dermatológicas	IIa	C
Para pacientes que fazem uso de varfarina e que serão submetidos a procedimentos dermatológicos, manter a medicação com ajuste de valores da razão normalizada internacional ≤ 3,5	IIa	C
Para pacientes que fazem uso de anticoagulantes orais diretos e que serão submetidos a procedimentos dermatológicos, manter a medicação, tomando-se o cuidado para que a intervenção cirúrgica seja marcada algumas poucas horas antes da próxima dose	IIa	C

#### 3.3.3. Endoscópicos

Do ponto de vista da análise de risco de eventos CV, os procedimentos endoscópicos são considerados de baixo risco,^151^ não sendo habitualmente necessária a suspensão do procedimento para intervenção CV, exceto nas condições CV graves, já apontadas na seção de algoritmos de avaliação perioperatória desta Diretriz (Item 2.1.1). Além disso, a maior parte dos medicamentos que pertencem à terapêutica CV não necessita ser interrompida e pode ser ingerida com o mínimo de água. Na verdade, a questão mais importante a ser levantada é se o paciente faz uso de medicações antitrombóticas, devido ao potencial risco de sangramento da intervenção endoscópica com a sua manutenção e pelo risco de eventos tromboembólicos em virtude da interrupção dessas medicações. Os procedimentos endoscópicos possuem diferentes potenciais para sangramento, sendo esse aspecto muito importante para a definição da estratégia a ser utilizada. O risco varia conforme o tipo do procedimento, principalmente relacionado à presença de intervenções terapêuticas e biópsias. A Tabela 9 apresenta os riscos de sangramento atribuídos a procedimentos endoscópicos comuns na prática clínica.^152^ O risco de eventos tromboembólicos com a interrupção da terapêutica antitrombótica varia de acordo com a indicação da terapêutica e as condições individuais dos pacientes.

**Tabela 9:** Classificação do risco de sangramento em procedimentos endoscópicos

Procedimentos de alto risco	Procedimentos de baixo risco
Polipectomia	Diagnósticos (EDA, colonoscopia e sigmoidoscopia flexível), incluindo biópsia em mucosa
Esfincterotomia biliar ou pancreática	CPRE com colocação de *stent* ou dilatação com balão sem esfincterotomia
Enteroscopia terapêutica assistida por balão	Enteroscopia *push* e enteroscopia diagnóstica assistida por balão
Gastrostomia ou jejunostomia percutânea endoscópica	Cápsula endoscópica
Ultrassonografia endoscópica **com** biópsia por agulha fina	Ultrassonografia endoscópica **sem** biópsia por agulha fina
Cistogastrostomia	Colocação de *stent* intestinal
Dilatação esofágica	Ablação de esôfago de Barrett
Mucosectomia e dissecção submucosa	Coagulação com plasma de argônio
Ablação de tumores	

Adaptada de Acosta et al.^152^ EDA: endoscopia digestiva alta; CPRE: colangiopancreatografia retrógrada endoscópica.

##### 3.3.3.1. Manejo de Antiplaquetários em Procedimentos Endoscópicos

Para procedimentos endoscópicos classificados como de baixo risco de sangramento, a terapêutica antiplaquetária pode ser mantida, seja na forma de monoterapia (independente do agente) ou DAP.^152 - 157^ Para procedimentos considerados de alto risco de sangramento, algumas considerações devem ser feitas. Pacientes em uso de dupla antiagregação plaquetária (DAP) por *stent* recente ou SCA (vide Itens 4.1 e 4.2) são aqueles com maior risco de eventos com a interrupção da terapêutica antiplaquetária.^144^ Assim, procedimentos endoscópicos eletivos de alto risco de sangramento, sempre que possível, devem ser adiados até que o período de maior risco seja finalizado. No entanto, para procedimentos que precisam ser realizados nesse período, as estratégias mais aceitas são a manutenção do AAS e a suspensão do segundo antiplaquetário,^155 , 158^ apesar das evidências para essa estratégia serem limitadas. Pacientes em uso de monoterapia com AAS para prevenção secundária de eventos CV podem manter seu uso no perioperatório de procedimentos endoscópicos, mesmo naqueles considerados de alto risco de sangramento, visto que a maioria das evidências na literatura demonstra baixo risco de sangramento significativo nessas circunstâncias.^156 , 157 , 159 - 169^ Alguns estudos têm demonstrado aumento de sangramento em procedimentos como dissecção submucosa em pacientes com neoplasia gástrica^170^ e mucosectomia em tumores colônicos maiores de 20 mm,^171^ situações que devem ser analisadas de maneira individual e de acordo com o risco de eventos trombóticos com a suspensão do AAS.^155^ Há alguma evidência da segurança do uso de clopidogrel em monoterapia durante a realização de gastrostomia percutânea endoscópica, podendo-se considerar sua manutenção nessa circunstância.^160^ Já as evidências do uso de prasugrel e ticagrelor em procedimentos endoscópicos de alto risco de sangramento são escassas. No caso da opção de suspensão da terapêutica antiagregante plaquetária, os intervalos entre a suspensão e o procedimento deverão seguir as recomendações desta Diretriz, que se encontram na seção de manejo de antiagregantes plaquetários.^144^ O antiplaquetário pode ser retomado após o procedimento, assim que a hemostasia esteja garantida, podendo-se considerar a realização de dose de ataque em pacientes de alto risco de eventos CV.^158^

##### 3.3.3.2. Manejo de Anticoagulantes em Procedimentos Endoscópicos

Para procedimentos endoscópicos com baixo risco de sangramento, a terapêutica anticoagulante com varfarina pode ser mantida,^152 , 153 , 155 - 157 , 161 , 169^ devendo ser suspensa naqueles com alto risco de sangramento.^156 , 162 , 168^ Até o momento, ainda não existem evidências em relação ao uso dos DOACs nessas circunstâncias, sugerindo-se sua manutenção em procedimentos com baixo risco de sangramento, bem como sua suspensão naqueles com alto risco de sangramento.^152 , 156 , 157 , 169^ Os intervalos para a suspensão e para a retomada dos DOACs e da varfarina (incluindo a consideração da terapêutica de ponte nos pacientes considerados de maior risco de eventos tromboembólicos) devem seguir as orientações da seção de manejo da anticoagulação no perioperatório desta Diretriz (Item 4.3). Com relação ao uso dos DOACs, tentar realizar o procedimento antes da próxima dose, procurando-se evitar o pico de ação da droga que se dá principalmente nas primeiras 2 horas após a sua administração.^157 , 169^

O Quadro 13 apresenta as recomendações para pacientes na programação de procedimentos endoscópicos.

**Quadro 13:** Recomendações para pacientes em programação de procedimentos endoscópicos

Recomendação	Grau de Recomendação	Nível de evidência
Para procedimentos endoscópicos com baixo risco de sangramento, deve ser mantida a terapêutica antiplaquetária (monoterapia ou dupla antiagregação plaquetária) ou anticoagulante com varfarina	I	B
Pacientes em uso de monoterapia com ácido acetilsalicílico para prevenção secundária de eventos cardiovasculares devem manter seu uso no perioperatório de procedimentos endoscópicos, inclusive na maioria dos procedimentos considerados de alto risco de sangramento	I	B
Para procedimentos endoscópicos de alto risco de sangramento, a terapêutica anticoagulante com varfarina ou com anticoagulantes orais diretos deve ser suspensa	I	B
Pacientes com dupla antiagregação plaquetária após angioplastia coronária não devem ser submetidos, se possível, a procedimentos endoscópicos de alto risco de sangramento, no período de duração ideal do tratamento	I	B
Pacientes que precisam ser submetidos a procedimentos endoscópicos de alto risco de sangramento antes do término previsto da dupla antiagregação plaquetária após angioplastia devem manter o ácido acetilsalicílico e suspender o segundo antiplaquetário	IIa	C
Para procedimentos endoscópicos de baixo risco de sangramento, a terapêutica anticoagulante com anticoagulantes orais diretos pode ser mantida	IIa	C

#### 3.3.4. Oftalmológicos

As intervenções cirúrgicas oftalmológicas são procedimentos relativamente frequentes na população de idade mais avançada. Comorbidades CV que demandam a utilização de medicamentos antitrombóticos e o que fazer com eles no período perioperatório são assunto de intenso debate entre os cirurgiões oftalmológicos e os cardiologistas. No Brasil, o temor de complicações hemorrágicas, incluindo hematomas na região periorbital, é responsável pela interrupção indiscriminada de AAS e varfarina em 82,7% dos pacientes que realizam operações de glaucoma.^172^ Por outro lado, as evidências relativas à ocorrência de complicações, ainda que escassas, demonstram que esse receio não é justificável. A taxa de complicações hemorrágicas descrita em estudos observacionais é muito baixa e sem maiores consequências, principalmente nas cirurgias de catarata que utilizam técnicas anestésicas convencionais.^173 - 177^ Alguns tipos de intervenção cirúrgica oftalmológica, entretanto, apresentam risco hemorrágico maior, como a trabeculectomia, usada no tratamento do glaucoma,^178 , 179^ e a vitrectomia, técnica para as doenças da retina.^180 , 181^ No entanto, as evidências não demonstram risco aumentado de complicações hemorrágicas significativas nessas cirurgias com o uso do AAS.^179 , 182 - 184^ Nesses casos, a conduta deve ser individualizada, mas, em geral, recomenda-se a manutenção desse agente no perioperatório.^184 , 185^ Pacientes em uso de DAP por *stent* recente ou SCA (vide Itens 4.1. e 4.2) são aqueles com maior risco de eventos com a interrupção da terapêutica antiplaquetária. Dessa maneira, os procedimentos oftalmológicos, sempre que possível, devem ser adiados até que seja finalizado esse período de maior risco. Para procedimentos que precisam ser realizados nesse período, a estratégia depende do risco hemorrágico da intervenção. Nas intervenções de menor risco hemorrágico (injeções intravítreas, catarata e anestesia peribulbar), o AAS e os inibidores dos receptores P2Y12 devem ser mantidos. No entanto, no caso de intervenções de maior risco hemorrágico, como vitrectomia e trabeculectomia, a recomendação mais aceita é de manter o AAS e suspender o segundo antiplaquetário, respeitando-se os intervalos já descritos em seção específica desta Diretriz (Item 4.2),^144^ apesar de as evidências para essa estratégia serem limitadas. De forma semelhante aos pacientes que fazem uso de monoterapia com AAS, as evidências na literatura são favoráveis à manutenção da monoterapia com clopidogrel no perioperatório de cirurgias de catarata.^176 , 177^ As evidências são mais escassas nas cirurgias de glaucoma e de retina, motivo pelo qual recomenda-se suspender o clopidogrel no perioperatório dessas intervenções, respeitando-se o período de 5 dias entre a suspensão e o procedimento.

Em relação aos pacientes anticoagulados com varfarina, as evidências na literatura são favoráveis à sua manutenção em operações de menor risco hemorrágico, como as de catarata, certificando-se que a anticoagulação avaliada pelo resultado da RNI esteja na faixa terapêutica.^173 , 174 , 184 , 186 , 187^ Uma metanálise de estudos observacionais, acompanhando pacientes submetidos à cirurgia de catarata na vigência do uso de varfarina, constatou incidência de sangramento da ordem de 10%; esses sangramentos foram, em sua maioria, autolimitados e subconjuntivais, e nenhum paciente cursou com perda visual relacionada a tal ocorrência.^175^ Já nas operações de glaucoma e doenças da retina, a varfarina deve ser suspensa e o manejo perioperatório deve seguir a estratégia descrita nesta Diretriz na seção de manejo de anticoagulação no perioperatório (consultar Item 4.3), de acordo com o risco individual de eventos trombóticos dos pacientes. Até o momento, as evidências dos riscos de hemorragia em pacientes utilizando os DOACs em cirurgias oftalmológicas só são bem estabelecidas no caso das cirurgias de catarata. O risco de sangramento com varfarina nas cirurgias de catarata foi bem estudado, e a maioria das diretrizes recomenda a sua manutenção desde que a RNI se encontre na faixa terapêutica. Os DOACs conferem um menor risco de sangramento do que a varfarina.^188^ Baseado em uma revisão sistemática e metanálise, sabe-se que os pacientes em uso de DOAC têm 22% de redução de risco relativo de sangramento intraocular espontâneo comparado com a varfarina.^189^ Dois pequenos estudos compararam a suspensão ou a manutenção dos DOACs durante a cirurgia de catarata e não acharam aumento de complicações relacionadas à manutenção da terapia com DOACs.^186 , 190^ Baseados nessas evidências, a tendência da maioria dos grandes centros tem sido manter o uso dos DOACs na cirurgia de catarata, principalmente pela ausência de evidências em contrário.^184 , 187^ Com relação às cirurgias com maior risco de sangramento, não há evidências com relação à segurança do uso dos DOACs, e a tendência tem sido a sua suspensão em geral 24 h antes da cirurgia.

As recomendações relativas ao que deve ser feito, principalmente para pacientes portadores de *stents* coronarianos e próteses valvares de natureza mecânica, devem ser individualizadas, considerando a relação entre os riscos trombótico e hemorrágico. Para os pacientes cuja recomendação for manter o anticoagulante e/ou antiagregante, o cirurgião deve ser informado da necessidade de garantir hemostasia adequada. Uma sugestão que pode ser considerada e discutida com o anestesiologista − a quem cabe a decisão final − é utilizar um tipo de anestesia específica, menos associada a complicações hemorrágicas.^177^ No caso dos antiagregantes, se a decisão for pela interrupção, eles devem ser reiniciados no pós-operatório o mais rapidamente possível. Recomenda-se também que o procedimento, nesses casos associados a maior risco CV, seja realizado em hospital com capacidade para intervenção hemodinâmica (angioplastia) de urgência, caso necessário.

O Quadro 14 apresenta as recomendações para pacientes em programação de operações oftalmológicas.

**Quadro 14:** Recomendações para pacientes em programação de operações oftalmológicas

Recomendação	Grau de recomendação	Nível de evidência
Para os pacientes que tiverem a recomendação de manter o anticoagulante e/ou antiagregante, o oftalmologista deve ser informado da necessidade de garantir hemostasia adequada	I	B
Pacientes que serão submetidos a operações oftalmológicas e estão em uso de ácido acetilsalicílico para prevenção cardiovascular secundária devem manter seu uso no perioperatório	I	B
Pacientes que serão submetidos a operações oftalmológicas para glaucoma ou vitrectomia e que estão em uso de clopidogrel em monoterapia devem suspender seu uso no perioperatório	I	C
Pacientes que serão submetidos à vitrectomia ou à trabeculectomia e estão em terapia anticoagulante com varfarina devem suspender seu uso no perioperatório	I	B
Pacientes que serão submetidos a cirurgia de catarata devem manter o uso de varfarina, desde que a razão normalizada internacional esteja dentro da faixa terapêutica	I	B
Pacientes que serão submetidos a cirurgia de catarata e estão em uso de anticoagulantes orais diretos podem manter o seu uso durante a cirurgia	I	B
Pacientes em uso de clopidogrel em monoterapia para prevenção cardiovascular secundária, que serão submetidos a operações de catarata, devem manter seu uso no perioperatório	IIa	B
Pacientes em uso de dupla antiagregação plaquetária por stent recente (vide Item 4.2) ou síndrome coronária aguda há menos de 1 ano e que necessitam realizar intervenções de menor risco hemorrágico (injeções intravitreais, catarata e anestesia peribulbar) devem manter o uso da dupla antiagregação plaquetária no perioperatório	IIa	B
Pacientes em uso de dupla antiagregação plaquetária por stent recente (vide Item 4.2) ou síndrome coronária aguda há menos de 1 ano e que necessitam realizar intervenções de maior risco hemorrágico (vitrectomia ou trabeculectomia) devem manter o uso do ácido acetilsalicílico e suspender os inibidores dos receptores P2Y12 no perioperatório	IIa	C

### 3.4. Doença Valvar

Pacientes portadores de valvopatia candidatos a cirurgias não cardíacas têm um maior risco de complicações CV durante o período perioperatório.^191^ Esse risco está relacionado ao tipo e à gravidade anatômica da valvopatia, à presença de sintomas e ao tipo de cirurgia a ser realizada.^6^ As principais complicações CV que podem ocorrer nesse contexto incluem congestão pulmonar, edema agudo de pulmão, choque cardiogênico, IAM, taquiarritmias, eventos embólicos, sangramentos e EI.^192^

Diante da suspeita de valvopatia após história clínica e exame físico detalhados, recomenda-se a realização de um ecocardiograma transtorácico. Esse exame tem como objetivo avaliar a gravidade anatômica da doença valvar, a função ventricular e o remodelamento das câmaras cardíacas e estimar a pressão sistólica da artéria pulmonar. Caso persistam dúvidas, outros métodos diagnósticos podem ser utilizados, como o ecocardiograma transesofágico, a tomografia computadorizada, a ressonância magnética e o cateterismo cardíaco.

Pacientes portadores de valvopatia anatomicamente importante sintomáticos apresentam alta morbimortalidade e devem ser submetidos a tratamento intervencionista valvar.^193 , 194^ Nos cenários eletivos, o tratamento da valvopatia deve ser priorizado antes da realização de cirurgias não cardíacas. No caso de cirurgia não cardíaca emergencial, o procedimento deverá ser realizado com cuidados específicos para cada valvopatia a fim de minimizar as chances de descompensação cardiovascular.

De uma maneira geral, as valvopatias estenóticas acarretam um maior risco perioperatório em comparação com valvopatias regurgitantes. Por esta razão, é necessário dispensar um cuidado adicional aos pacientes com estenose aórtica e/ou estenose mitral.^3 , 195^ Se houver lesão de mais de uma valva cardíaca e/ou combinação de estenose e IC, os cuidados devem ser baseados na lesão de gravidade anatômica mais significativa.

#### 3.4.1. Estenose Aórtica

A estenose aórtica é uma valvopatia comum em pacientes idosos, afetando aproximadamente 2 a 4% dos adultos com mais de 75 anos,^196^ com expectativa de aumento desse número nos próximos anos. Quando a estenose aórtica é anatomicamente importante, representa um fator de risco bem estabelecido para mortalidade, IC e IAM no período perioperatório de cirurgias não cardíacas.^193^

Nos pacientes com estenose aórtica anatomicamente importante, assintomáticos, é seguro realizar cirurgias não cardíacas de baixo risco, desde que se evite sobrecarga de volume.^191 , 193^ Nesses casos, se houver dúvidas sobre a condição de sintomas do paciente, é prudente realizar teste funcional. O *Heart Team* , incluindo cirurgião e anestesista, tem um papel importante na tomada de decisão.

Nos pacientes com estenose aórtica importante sintomática ou em programação de cirurgia eletiva de risco moderado a alto, a correção valvar deve ser priorizada antes da cirurgia não cardíaca.^197 - 199^ A substituição valvar associa-se à menor mortalidade intra-hospitalar e em 30 dias em indivíduos submetidos a cirurgias de risco intermediário e alto.^198 , 199^ Além disso, um estudo realizado por Mizuno et al.,^197^ com 218 pacientes, demonstrou que portadores de estenose aórtica submetidos a cirurgia não cardíacas de alto risco apresentaram progressão mais rápida da valvopatia em comparação com aqueles que não passaram por intervenção cirúrgica. A escolha entre cirurgia ou implante transcateter da valva aórtica (TAVI) deve ser baseada nas diretrizes mais recentes de valvopatia. Quando a cirurgia não cardíaca precisar ser realizada com brevidade, é importante considerar a possibilidade de TAVI devido à recuperação mais rápida associada a esse procedimento.^5^ Nos casos em que a TAVI e a cirurgia valvar forem inviáveis, a valvoplastia por cateter-balão pode ser uma alternativa, embora seja necessário ponderar os riscos desse procedimento e estar ciente de que ocorrerá reestenose posteriormente.^193 , 197 , 200^

Se a cirurgia não cardíaca for urgente ou tempo-dependente, como no caso de fratura de fêmur, é necessário realizar monitorização hemodinâmica invasiva durante anestesia, evitando mudança do *status* volêmico, especialmente devido ao elevado risco de hipervolemia. Nesses casos, é recomendado pós-operatório em unidade de tratamento intensivo (UTI), com monitorização hemodinâmica e eletrocardiográfica contínuas, além da realização de dosagens seriadas de troponina. Nas cirurgias tempo-dependente, podem ser consideradas a realização de valvoplastia por cateter-balão e TAVI a depender da gravidade anatômica da lesão e da disponibilidade desses recursos.

#### 3.4.2. Estenose Mitral

Pacientes com estenose mitral discreta a moderada podem ser submetidos a cirurgias não cardíacas com segurança, desde que sejam tomadas medidas para prevenir taquicardia e sobrecarga volêmica antes e após o procedimento.

Pacientes com estenose mitral importante, mesmo na ausência de sintomas, apresentam maior risco de complicações CV. Nos casos em que há indicação de correção cirúrgica ou percutânea da valvopatia, os pacientes devem ser submetidos ao procedimento valvar antes de qualquer cirurgia não cardíaca eletiva.^201^ Em situações de estenose mitral importante assintomática e sem indicação de tratamento intervencionista, deve-se considerar o tratamento valvar antes de cirurgias de alto risco.

Em situações de cirurgia não cardíaca emergencial, é recomendada a monitorização hemodinâmica invasiva e prevenção de taquicardia e hipervolemia. O aumento da frequência cardíaca, especialmente se houver o desenvolvimento de fibrilação atrial (FA), pode levar à congestão e ao edema pulmonar. Dessa forma, o emprego de betabloqueador e/ou diurético pode ser feito no período perioperatório.

#### 3.4.3. Insuficiência Aórtica e InsuficiêNcia Mitral

Valvopatias regurgitantes primárias estão associadas a um aumento do risco cardíaco durante cirurgias não cardíacas, porém são mais bem toleradas do que as lesõs valvares estenóticas.^202 , 203^ Insuficiência aórtica e insuficiência mitral de graus leve ou moderado não acarretam aumento do risco de complicaçõs CV durante cirurgia não cardíaca. No caso de disfunção valvar importante, desde que não gerem sintomas e com fração de ejeção preservada, também é possível realizar cirurgias não cardíacas com cautela para evitar sobrecarga volêmica.

Por outro lado, no caso de insuficiência aórtica ou insuficiência mitral primárias importantes com indicação de troca valvar, há elevado risco de complicaçõs CV, e a correção da valvopatia deve ser priorizada.^191^ Nos casos de insuficiência mitral secundária sintomática de graus moderado a importante, é importante avaliar, em conjunto com o *Heart Team* , se o paciente é candidato à cirurgia ou à correção transcateter com dispositivos *edge-to-edge* , antes da cirurgia não cardíaca eletiva.^193^

Caso o procedimento não cardíaco seja de urgência ou emergência, deve ser realizado após otimização do tratamento farmacológico e estabilização hemodinâmica, empregando-se preferencialmente vasodilatadores e diuréticos, além do pós-operatório em UTI.

#### 3.4.4. Prótese Valvar

Pacientes portadores de prótese valvar normofuncionante e sem disfunção ventricular esquerda podem ser submetidos a cirurgia não cardíaca sem risco adicional.

O Quadro 15 apresenta as recomendações para pacientes com doenças valvares.

**Quadro 15:** Recomendações para pacientes com doenças valvares

Recomendação	Classe de recomendação	Nível de evidência
Realizar ecocardiograma em pacientes sabidamente portadores ou com suspeita de alteração anatômica valvar moderada/importante e que serão submetidos à cirurgia de risco intermediário ou alto, sem avaliação nos últimos 6 a 12 meses ou que apresentaram piora clínica	I	C
Pacientes portadores de valvopatia com indicação de tratamento intervencionista valvar devem, prioritariamente, ser submetidos ao tratamento cardíaco e, posteriormente, à cirurgia não cardíaca proposta	I	B
Pacientes com estenose aórtica importante, assintomática, em programação de operações não cardíacas eletivas de risco intermediário e alto deverão ser submetidos ao tratamento intervencionista da valvopatia antes da operação não cardíaca	I	B
Pacientes com estenose mitral importante, assintomática e com complicadores (pressão sistólica na artéria pulmonar [PSAP] ≥ 50 mmHg no repouso ou PSAP ≥ 60 mmHg ao esforço) deverão ser submetidos ao tratamento da valvopatia antes da cirurgia não cardíaca	I	C
Pacientes com valvopatia regurgitante importante assintomática podem ser submetidos à cirurgia não cardíaca eletiva	I	C

### 3.5. Transplante de Órgãos Sólidos

#### 3.5.1. Fígado

Considerado o tratamento de escolha para boa parte das doenças hepáticas terminais, o transplante de fígado vem se tornando um procedimento rotineiro em vários centros. Novas abordagens anestésicas, o aumento da experiência dos cirurgiões e a melhoria nos cuidados de pós-operatórios têm possibilitado condutas que, até pouco mais de uma década, seriam exceção. Dessa forma, muitos grupos têm estendido as indicações de transplante a populações cada vez mais idosas e para casos mais complexos que, anteriormente, seriam contraindicados.^204^ Adicionalmente, indicações por esteato-hepatite não alcoólica, habitualmente associada a obesidade, diabetes, hipertensão e doenças CV, têm se tornado cada vez mais frequentes, passando a liderar as indicações de transplante hepático em alguns centros.^205^

Estudos mostram que, nos pacientes submetidos a transplante de fígado, até 26% têm alguma lesão coronariana crítica.^206^ No período de 1 ano após o transplante, 15,2% dos pacientes têm algum tipo de doença CV e 2,8% morrem em função delas.^207^ Dessa forma, a mortalidade por causas cardíacas se mantém como a terceira causa mais frequente após o transplante de fígado, atrás apenas de infecções e rejeição ou disfunção de enxerto. Os eventos cardíacos mais comuns são arritmias, edema pulmonar e disfunção ventricular, além de morte súbita e IAM.^206^ Por essas razões, a avaliação cardiológica no pré-transplante é crucial para definir o manejo correto desses pacientes, tanto no pré como no pós-operatório.

Além dos eventos relacionados à DAC, outras comorbidades características do paciente hepatopata podem aumentar a morbimortalidade no pós-transplante de fígado. Entre elas, as principais são a cardiomiopatia relacionada ao álcool e a própria cirrose, a hipertensão pulmonar (HP) e a síndrome hepatopulmonar (SHP).

A Tabela 10 ^208^ resume as principais comorbidades cardiopulmonares dos pacientes candidatos ao transplante de fígado, os achados clínicos, o diagnóstico e a evolução esperada após o transplante.

**Tabela 10:** Prevalência de comorbidades cardiopulmonares em pacientes candidatos a transplante de fígado

	Prevalência	Expectativa de evolução após o transplante de fígado
Doença arterial coronariana	2,5-27%	Progressão, expectativa de piora dos fatores de risco
Cardiomiopatia cirrótica	Desconhecida, mas provavelmente elevada	Reversão após o transplante de fígado
Doença cardíaca valvar	Desconhecida, mas provavelmente baixa	Progressão independente do transplante de fígado
Doença pulmonar obstrutiva crônica	18%	Progressão independente do transplante de fígado
Hidrotórax hepático	5-12%	Resolução após o transplante de fígado
Síndrome hepatopulmonar	20%	Resolução após o transplante de fígado na maior parte dos casos
Hipertensão pulmonar	4%	Inalterada ou lentamente progressiva

Adaptado de Martinez-Palli et al.^208^

##### 3.5.1.1. Cardiomiopatia Associada à Cirrose (CAC)

A cardiomiopatia associada à cirrose (CAC) caracteriza-se pela seguinte tríade no cirrótico:^209 , 210^ disfunção sistólica (decorrente principalmente de um déficit na resposta contrátil induzida pelo estresse e de FEVE em repouso abaixo de 55%), disfunção diastólica e alterações eletrofisiológicas (especialmente aumento do intervalo QT, déficit cronotrópico e bradicardia, alterações de repolarização ventricular). Aumento de átrio esquerdo, da massa miocárdica, elevação do peptídeo natriurético do tipo B (BNP), do BNP N-terminal (NT-pró-BNP) e da troponina I também são comumente encontrados.

Apesar de esses achados aumentarem a morbimortalidade nos candidatos ao transplante de fígado, não se demonstrou nenhum benefício no tratamento específico dessas alterações.

##### 3.5.1.2. Cardiopatia Alcóolica

A cardiopatia alcóolica chega a acometer 21 a 32% dos pacientes com cardiomiopatia dilatada em alguns relatos.^211 , 212^ Considerando que a cirrose alcoólica está entre as maiores causas de doença hepática, compreende-se que a ocorrência concomitante da cirrose com cardiomiopatia dilatada é relativamente comum e seu diagnóstico deve ser considerado na avaliação pré-operatória, durante a qual, não raramente, são diagnosticadas doenças até então desconhecidas do paciente.

##### 3.5.1.3. Hipertensão Portopulmonar (HPP)

O estado hiperdinâmico do paciente com hipertensão portal pode provocar a vasoconstricção e remodelamento dos vasos pulmonares, levando à HP. Tais alterações acometem de 5 a 10% dos candidatos ao transplante e não estão relacionadas à etiologia ou à gravidade da hipertensão portal, mesmo na ausência de cirrose hepática.^213 , 214^ Pela classificação de HP, a hipertensão portopulmonar (HPP) pertence ao grupo 1, devido às suas semelhanças patológicas e hemodinâmicas com as demais causas de HP pré-capilar. Pode ser estratificada, conforme a pressão média da artéria pulmonar, em leve (> 25 e < 35 mmHg), moderada (> 35 e < 45 mmHg) ou severa (> 45 mmHg).^213^ O quadro clínico e os exames apresentam características clínicas únicas.^215 , 216^

**Figura 3:**
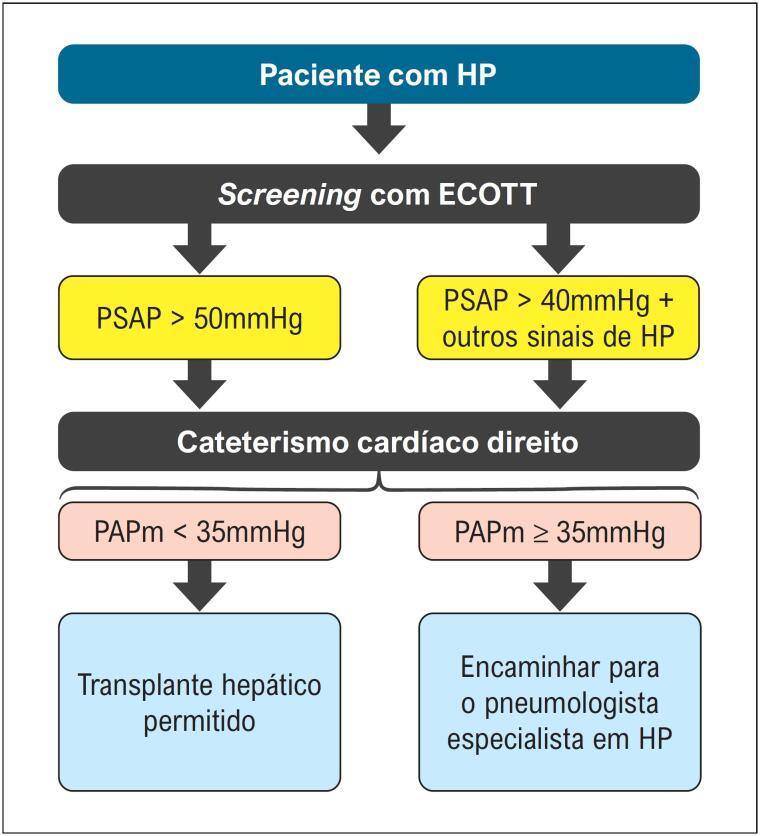
Avaliação da pressão arterial pulmonar em candidatos a transplante hepático. HP: hipertensão pulmonar; ECOTT: ecocardiograma transtorácico; PSAP: pressão sistólica de artéria pulmonar; PAPm: pressão arterial pulmonar média. Adaptada de Cartin-Ceba e Krowka.^214^

Como os sintomas são inespecíficos e o diagnóstico de HPP impacta diretamente na elegibilidade para o transplante de fígado, a triagem com ecocardiograma transtorácico é recomendada para todos os candidatos. Uma pressão sistólica da artéria pulmonar (PSAP) acima de 50 mmHg possui uma elevada acurácia diagnóstica de HPP em candidatos a transplante hepático (sensibilidade de 97% e especificidade de 77% para HPP moderada a importante).^217^ Além disso, o ecocardiograma é útil para avaliar a função ventricular direita e descartar disfunção do VE e doenças valvares, que podem gerar um componente de HP pós-capilar.^216^ As diretrizes da International Liver Transplantation Society sugerem o cateterismo direito para obtenção do diagnóstico definitivo de HPP se a PSAP estimada no ecocardiograma transtorácico for superior a 50 mmHg. No entanto, novas evidências demonstram um aumento na sensibilidade do diagnóstico se for utilizado ponto de corte de PSAP estimada maior que 40 mmHg. Assim, de acordo com as condições dos serviços, a PSAP pelo ecocardiograma entre 40 e 45 mmHg pode ser utilizada como indicação para o cateterismo cardíaco, especialmente se houver outros indicativos de HP ( Figura 3 ).^216 , 217^

O diagnóstico de HPP envolve a exclusão de outras causas de HP e a presença de parâmetros hemodinâmicos confirmatórios no cateterismo direito:^216^

- Pressão arterial pulmonar média (PAPm) > 25 mmHg
- Resistência vascular pulmonar (RVP) > 3 Wood (ou 240 dyn.s.cm-5)
- Pressão capilar pulmonar (PCP) < 15 mmHg. Se PCP > 15 mmHg, provavelmente por hipervolemia (comum nos cirróticos), um gradiente transpulmonar (PAPm-PCP) > 12 mmHg sugere HPP.

Vale ressaltar que aproximadamente 20% dos pacientes cirróticos possuem aumento moderado da pressão pulmonar no ecocardiograma, mas apenas uma fração pequena possuí realmente HPP.^218^

O manejo da HPP depende dos parâmetros hemodinâmicos avaliados pelo cateterismo direito ( Figura 3 ).^214^ Em pacientes com PAPm entre 35 mmHg e 50 mmHg com aumento da RVP (> 240), o transplante hepático não pode ser realizado, a menos que haja queda da PAPm e da RVP após o tratamento medicamentoso da HP. Essas medicações possuem efeitos vasodilatador, antiagregante plaquetário e antiproliferativo, podendo atuar na redução da pressão arterial pulmonar.^214^ As principais classes disponíveis são:

- Inibidores de fosfodiesterase (ex.: sildenafil)
- Antagonistas dos receptores de endotelina (ex.: bosentan, ambrisentan, macitentan)
- Estimulador solúvel da guanilato ciclase (riociguat)
- Prostanoides (epoprostenol, iloprost, trepostinil)

No entanto, a equiparação da morbimortalidade de pacientes tratados com tais medicações com os pacientes sem diagnóstico de HP ainda não foi demonstrada. Mais estudos são necessários nesta área.

A mortalidade perioperatória em HPP grave é próxima de 100%, motivo pelo qual se constitui como contraindicação ao transplante isolado de fígado, podendo, em centros seletos, ser indicado o transplante combinado pulmão-fígado.^219^

##### 3.5.1.4. Síndrome Hepatopulmonar

Apesar de ser por vezes confundida com a HPP, a SHP apresenta características diversas da primeira, sendo definida como hipóxia em presença de doença hepática que piora com a postura ereta, com evidência de vasodilatação intrapulmonar. A hipóxia se dá pelo acúmulo de vasodilatadores pulmonares, em especial o óxido nítrico, levando a *shunt* arteriovenoso intrapulmonar.^220^

A SHP é definida pela seguinte tríade:^214^

- Presença de hipertensão portal ou doença hepática crônica ou shunts portossistêmicos congênitos;
- Hipoxemia (gradiente alvéolo-arterial > 15 mmHg ou 20 mmHg se idade > 64 anos); e
- Presença de vasodilatação intrapulmonar, levando a shunt (aparecimento de bolhas em câmaras esquerdas após três ciclos cardíacos no ecocardiograma com microbolhas).

A SHP acomete de 5 a 32% dos candidatos ao transplante e não está relacionada à etiologia ou à gravidade da doença hepática.^221 , 222^

O quadro clínico típico consiste em um paciente com estigmas de hepatopatia crônica associados a cianose, baqueteamento digital, dispneia na posição ereta (platipneia) e hipóxia também nesta posição (ortodeoxia). A hipoxemia é um achado marcante nesta síndrome.^214^

A triagem é realizada com a oximetria de pulso. Uma saturação de oxigênio inferior a 96% possui uma sensibilidade de 100% e especificidade de 88% para detecção de pressão parcial de oxigênio arterial (PAO2) < 60 mmHg.^214^ Portanto, recomenda-se a coleta de gasometria arterial nesses pacientes. Se a gasometria evidenciar um gradiente alvéolo-arterial elevado (≥ 15 mmHg ou 20 mmHg se idade > 64 anos) e uma PAO2 < 80 mmHg, procedemos ao ecocardiograma transtorácico com microbolhas.^214^ Se houver um *shunt* intracardíaco (por exemplo, comunicação interatrial, forame oval patente), as bolhas passam para as câmaras cardíacas esquerdas após um a dois ciclos cardíacos, a contar da visualização das bolhas no átrio direito. Se essas bolhas aparecerem no átrio esquerdo após três ciclos cardíacos, está confirmada a existência do *shunt* pulmonar (as bolhas conseguem passar pelos vasos pulmonares dilatados na SHP). Vale salientar que o ecocardiograma com microbolhas é sensível, porém não é específico para SHP, pois diversos pacientes cirróticos possuem o exame positivo ( *shunts* intrapulmonares) sem fechar critério para SHP por não apresentarem hipoxemia. Um exame mais específico para SHP é a cintilografia pulmonar com macroagregado de albumina-99mm Tc.^214^

O manejo da SHP consiste em suporte com oxigênio, apesar de não evidenciar benefício sustentado na melhora da dispneia e na qualidade de vida desses pacientes. Diversas terapias medicamentosas já foram testadas, porém sem benefício comprovado.^214^

Diferentemente da HP, o tratamento de escolha para a SHP é o transplante de fígado, embora dados não definitivos correlacionem o grau de hipóxia com a mortalidade perioperatória.^223^

##### 3.5.1.5. Doença Arterial Coronariana

Os fatores de risco para a DAC estão tão ou mais presentes nos pacientes cirróticos do que na população geral. Em pacientes com doença hepática avançada, a prevalência de diabetes e de DAC é igual ou maior que na população geral. Em especial nos pacientes com diabetes melito, a prevalência de DAC varia entre 2,5 a 27%.^224 , 225^ Por outro lado, o grau de comprometimento da doença (grau de estenose) não parece se correlacionar com um pior prognóstico.^226^ Comparando os candidatos a transplante hepático com os candidatos a transplante renal, os últimos possuem maior frequência de diabetes melito, hipertensão arterial e DAC.^227^

###### 3.5.1.5.1. Quando Investigar DAC?

As indicações de testes não invasivos para DAC em pacientes com doença hepática avançada variam entre as sociedades: algumas indicam a todos os candidatos a transplante hepático, ao passo que outras sugerem a estratificação de risco cardiovascular antes de solicitar o exame.^228^ Uma estratégia interessante para pacientes assintomáticos para DAC e candidatos a transplante hepático é avaliar a presença de fatores de risco cardiovasculares (sexo masculino, hipertensão arterial, diabetes melito, dislipidemia, tabagismo prévio ou atual e história familiar de DAC precoce).^219^ Se zero ou um fatores, o valor preditivo negativo para DAC obstrutiva é elevado, portanto, não há necessidade de solicitar teste não invasivo. Já se houver dois ou mais fatores, há indicação de prosseguir investigação com teste não invasivo.^225^

Para pacientes sintomáticos, devemos seguir a avaliação habitual para DAC, como preconizada nas diretrizes específicas, estratificando o paciente de acordo com sintomas (característica da dor, dispneia) e com os fatores de risco (sexo, idade).^228 , 226^ Após a estratificação, determina-se a probabilidade pré-teste de DAC. Se for baixa/intermediária (< 15%), pode-se optar por testes não invasivos (anatômicos ou funcionais) ou por não prosseguir investigação (se probabilidade muito baixa de DAC). Se for elevada (> 15%), deve-se dar preferência ao cateterismo cardíaco. Em geral, a preferência para abordagem invasiva é dada em pacientes com angina importante e refratária ao tratamento clínico, disfunção de VE ou achados de alto risco nos testes não invasivos.^228^

###### 3.5.1.5.2. Qual Exame Solicitar para Investigar DAC?

Uma vez classificado o risco do paciente, passa-se à escolha da melhor forma de investigação complementar. Pela própria característica dos candidatos ao transplante de fígado, essa investigação não tem uma padronização formal. Boa parte dos pacientes tem limitações à locomoção, ascites volumosas, neuropatia pela própria cronicidade da doença etc., não permitindo, por exemplo, teste ergométrico. Ademais, cerca de 50% dos pacientes com cirrose apresentam prolongamento do intervalo QT, o que pode prejudicar a avaliação do teste.^216^ Além disso, a doença hepática terminal se caracteriza por vasodilatação e taquicardia, prejudicando a resposta induzida por vasodilatadores (dipiridamol e adenosina) na cintilografia miocárdica. Os receptores beta-adrenérgicos respondem pouco aos estímulos simpáticos, levando a respostas duvidosas nos estudos com dobutamina.^215 , 217 , 218^ Já foi comparado o uso de dobutamina *versus* vasodilatadores como estresse farmacológico nesse cenário de pré-operatório de transplante hepático, e as evidências favorecem a dobutamina, sobretudo o ecocardiograma com essa modalidade de estresse farmacológico.^218 , 219^ Também nesse caso, a indicação de qual teste utilizar deve respeitar as características regionais de cada centro.

A angiotomografia de coronárias também já foi avaliada nesse contexto e, quando normal ou com lesões coronarianas não obstrutivas, alcançou um valor preditivo negativo de 95% para eventos cardiovasculares maiores e de 100% para eventos clínicos coronarianos. Um escore de cálcio acima de 400 tem alto poder preditivo para eventos cardiovasculares precoces nesses pacientes.^226^ Um cuidado que devemos ter com este exame e também com o cateterismo cardíaco é o uso de contraste. A doença hepática terminal normalmente vem acompanhada por disfunção renal, que deve ser levada em conta antes de indicarmos esses exames.

###### 3.5.1.5.3. Quando e Como Intervir na DAC?

A opção pelo tratamento desses pacientes deve obedecer ao critério de que a não intervenção pode levar a um risco excessivo durante e após a cirurgia. No entanto, a melhor opção de tratamento não está estabelecida e deve ser individualizada para cada paciente.

A indicação de procedimentos percutâneos, com colocação de *stents* , que, pelo consenso atual para esse tipo de intervenção, devem ser farmacológicos, deve levar em conta a característica tempo-sensível da cirurgia do transplante de fígado, decidindo-se pela opção que menor impacte o tempo de espera em lista.

A opção de revascularização cirúrgica deve, quando possível, ser postergada para depois do transplante, pela grande chance de ocorrerem eventos hemorrágicos ou agravamento da situação hepática com a cirurgia. A cirurgia de revascularização miocárdica antes do transplante deve ser reservada apenas para pacientes em que o risco de morte pela DAC supere o risco de morte de doença hepática. A cirurgia de revascularização miocárdica pode ser realizada após o transplante de fígado com razoável segurança e baixas taxas de complicação.^229 , 230^

O Quadro 16 apresenta as recomendações para pacientes em programação de transplante hepático.

**Quadro 16:** Recomendações para pacientes em programação de transplante hepático

Recomendação	Classe de recomendação	Nível de evidência
Eletrocardiograma e radiografia de tórax devem ser solicitados para todos os pacientes	I	C
Ecocardiograma deve ser solicitado para todos os pacientes	I	B
Para pacientes com ecocardiograma mostrando pressão sistólica da artéria pulmonar (PSAP) acima de 50 mmHg, deve ser solicitado o cateterismo cardíaco direito com medida da pressão em artéria pulmonar	I	C
Para pacientes com ecocardiograma mostrando PSAP acima de 40 mmHg, principalmente se outros sinais de hipertensão pulmonar estiverem presentes, deve-se considerar o cateterismo cardíaco direito com medida da pressão em artéria pulmonar	IIa	C
Para pacientes assintomáticos para doença arterial coronariana (DAC), sem disfunção segmentar ao ecocardiograma e com dois ou mais fatores de risco para DAC * , solicitar preferencialmente um ecocardiograma transtorácico com dobutamina	IIa	B
Para pacientes sintomáticos para DAC, calcular a probabilidade pré-teste de DAC e solicitar os exames adicionais conforme as diretrizes específicas	IIa	C
A cineangiocoronariografia deve ser realizada em pacientes com alta probabilidade pré-teste de DAC, angina importante e refratária ao tratamento clínico, disfunção nova de ventrículo esquerdo ou achados de alto risco nos testes não invasivos, apesar das complicações hemorrágicas serem mais comuns e alterações como elevação da creatinina poderem contribuir para o aumento na morbidade dos cirróticos	IIa	C
A intervenção coronariana percutânea com colocação de *stents* (quando for necessário realizá-la antes do transplante em função da gravidade) deve levar em conta a possibilidade de o paciente falecer em função da doença hepática enquanto aguarda o período de antiagregação e o benefício real da intervenção em minimizar riscos perioperatórios	IIa	C
A cirurgia de revascularização miocárdica antes do transplante deve ser reservada apenas para pacientes em que o risco de morte pela DAC supere o risco de morte pela doença hepática, e a decisão deve ser compartilhada com uma equipe multidisciplinar	IIa	C
Os vasodilatadores pulmonares podem ser usados para tentar reduzir a hipertensão pulmonar em pacientes com pressão arterial pulmonar (PAP) média entre 35 e 45 mmHg e aumento da resistência vascular sistêmica (> 240 dynes)	IIb	B
Pacientes com PAP média maior ou igual a 35 mmHg no cateterismo direito devem ser encaminhados ao especialista em hipertensão pulmonar^213^	IIb	C
Realizar transplante hepático em pacientes com hipertensão pulmonar severa em centros que não ofereçam terapias agressivas para redução da PAP ou, ainda, a possibilidade de transplante concomitante de pulmão	III	B

* Sexo masculino, hipertensão arterial, diabetes melito, dislipidemia, tabagismo prévio ou atual e história familiar de DAC precoce.

#### 3.5.2. Rim

Pacientes com doença renal crônica estágio 5 (taxa de filtração glomerular < 15 mL/min/1,73m^2^) em tratamento dialítico ou não constituem um dos grupos de maior risco cardiovascular, com taxas de mortalidade cardiovascular cinco a cem vezes maiores do que aquelas encontradas na população geral para a mesma faixa etária. Embora o transplante renal se associe à redução da taxa de complicações cardiovasculares em comparação a pacientes mantidos em diálise, sua incidência permanece mais elevada em transplantados de rim do que na população geral. De fato, a doença cardiovascular é a principal causa de óbito pós-transplante, especialmente devido à DAC.^231^ O período pós-transplante apresenta alto risco de IAM, principalmente durante o primeiro ano.^232^ Gill et al. relataram, em uma coorte de 600 pacientes, um aumento significativo na incidência de eventos cardiovasculares (CV) no primeiro ano após o transplante (39,6/100 pacientes-ano, intervalo de confiança de 95% [IC95%] 20,6-76,1) em comparação ao período pré-transplante.^233^ Nos primeiros 30 dias após o transplante renal, aproximadamente metade dos óbitos é consequência de IAM.^234^ Da mesma maneira, no seguimento tardio, a doença isquêmica crônica do coração é responsável por mais de um terço dos óbitos em pacientes com enxerto funcionante.^234^

Desse modo, a avaliação pré-operatória de candidatos a transplante renal visa não apenas à redução do risco cardiovascular no curto prazo, relacionado ao procedimento cirúrgico, mas também à redução de eventos CV tardios. Durante a avaliação de candidatos ao transplante renal, a identificação da presença e da extensão da DAC se reveste de importância fundamental por permitir que a equipe médica estabeleça mais precisamente o risco/benefício do transplante, a eventual necessidade de intervenção coronária no pré-operatório, o uso de medidas cardioprotetoras no perioperatório e o controle de fatores de risco no pós-operatório.^235 , 236^

O objetivo desta seção é fornecer ao cardiologista os meios mais adequados de estabelecer o risco cardiovascular em uma população muito especial de pacientes, quase sempre excluída dos estudos de estratificação de risco operatório. A meta principal consiste em identificar, entre candidatos ao transplante renal, aqueles com maior probabilidade de diagnóstico de DAC. Dessa maneira, as recomendações aqui incluídas devem ser aplicadas apenas aos pacientes assintomáticos ou com sintomas atípicos; para aqueles indivíduos com evidências clínicas e/ou achados de exames de investigação diagnóstica sugestivos de DAC, a investigação complementar e o tratamento devem seguir as regras propostas para a população geral contidas em seções específicas desta Diretriz.

A identificação de DAC é um imenso desafio em candidatos ao transplante renal. Doença coronária obstrutiva significativa, definida como estenose ≥ 70% em vasos epicárdicos maiores (ou ≥ 50% no tronco da artéria coronária esquerda), é descrita em candidatos a transplante renal, sendo observada em até 50% dos indivíduos dependendo dos critérios de inclusão para estudo angiográfico.^237 , 238^ No entanto, esses pacientes frequentemente se apresentam assintomáticos ou com sintomas atípicos na presença de DAC avançada. Os métodos não invasivos de detecção de isquemia miocárdica, como teste ergométrico, cintilografia de perfusão miocárdica ou ecocardiograma sob estresse farmacológico − todos rotineiramente utilizados na população geral −, apresentam sensibilidade e especificidade menores do que em indivíduos com função renal normal, propiciando grande número de resultados falso-negativos.^239 - 241^ Mais recentemente, o estudo ISCHEMIA-CKD12 mostrou, em uma população de pacientes com doença renal crônica estágios 4 ou 5, que a revascularização miocárdica baseada na quantificação da área isquêmica não foi superior ao tratamento clínico otimizado na ocorrência de desfechos CV compostos. Cabe salientar que, nesse estudo, pouco mais da metade dos pacientes incluídos estava em terapia de substituição renal (53,4%), não se tratando, portanto, de um estudo específico para a população de pacientes dialíticos. De qualquer forma, não houve interação significativa entre o efeito do tratamento (intervenção *versus* tratamento conservador) para o desfecho primário no subgrupo diálise *versus* não diálise.^242^

O uso indiscriminado da cineangiocoronariografia não se justifica, por se tratar de método invasivo, não isento de riscos de complicações e com custo elevado, além de resultar em taxas superiores a 50% de exames normais ou sem lesões obstrutivas significativas. Por outro lado, estudos observacionais sugerem que pacientes com coronariopatia importante apresentam risco elevado de eventos e de morte CV.^243^ Avanços na área de imagem CV podem levar à substituição da avaliação invasiva por testes não invasivos para documentação não apenas da presença e extensão de aterosclerose coronariana, mas também do seu significado funcional.^244 , 245^

Até o momento, as evidências não suportam a indicação de intervenção baseada pelo achado de doença coronária obstrutiva durante a avaliação pré-transplante renal.^72 , 246 - 248^ Em um estudo retrospectivo envolvendo 406 pacientes submetidos à coronariografia invasiva antes do transplante renal, a ocorrência de eventos CV (morte CV, síndrome coronariana aguda ou necessidade de revascularização pós-transplante) foi similar entre pacientes com DAC obstrutiva e não revascularizados (23%) e revascularizados (26%) no seguimento de 5 anos.^249^

Desse modo, a investigação da presença e da extensão de DAC obstrutiva em candidatos a transplante renal deve ser apenas mais uma etapa na avaliação geral do risco dessa população, a fim de identificar aqueles indivíduos com maior chance de se beneficiar de estratégias de revascularização e que, portanto, devem ser encaminhados para estudo angiográfico. Tal estratégia deve reduzir tanto o número de testes invasivos desnecessários, otimizando os recursos econômicos disponíveis,^250^ quanto levar a uma relação mais favorável entre o risco inerente da intervenção (maior nesses pacientes do que na população geral) e o benefício prognóstico no longo prazo.

##### 3.5.2.1. Estratificação do Risco da Presença de DAC

Não existe consenso quanto à melhor estratégia para investigar e tratar a DAC nesse grupo de pacientes.^251^ O assunto é controverso, e existem até mesmo propostas para eliminar de uma vez por todas a pesquisa de DAC oculta em indivíduos assintomáticos.^252^ Isso se deve, sobretudo, à carência de estudos prospectivos nessa população.^253^ Sabe-se, no entanto, que os parâmetros clínicos mais fortemente associados à doença cardíaca isquêmica pós-transplante renal são idade > 50 anos, diabetes melito e evidência prévia de doença CV (história clínica e/ou achados de exames). A prevalência de DAC significativa (estenose ≥ 70%) aumenta de acordo com o número de fatores de risco presentes. Esses três fatores de risco têm servido de base na formulação de algoritmos de investigação de DAC em pacientes com doença renal crônica.^254^ Outros fatores considerados preditores de eventos cardiovasculares nesta população são: hipertensão arterial sistêmica, disfunção ventricular, hipertrofia ventricular esquerda, tabagismo, dislipidemia e tempo em diálise acima de 1 ano. Em geral, os fatores de risco tradicionais têm menor impacto em pacientes renais, e os escores de risco, como o Framingham, subestimam o risco real de eventos nesses pacientes.^255^

**Figura 4:**
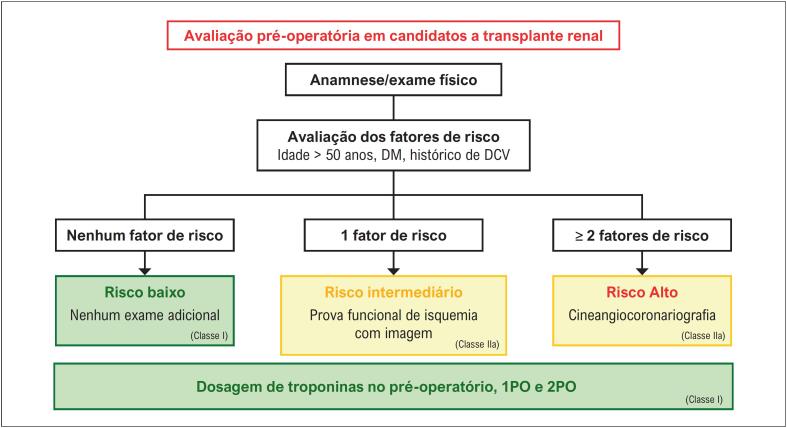
Fluxograma de avaliação perioperatória em pacientes candidatos a transplante renal. DM: diabetes mellitus; DCV: doença cardiovascular; PO: pós-operatório.

Com base nos resultados de vários estudos, a maioria observacionais, propusemos um modelo de estratificação de risco dos pacientes renais crônicos assintomáticos do ponto de vista CV, em avaliação para transplante renal, conforme a presença ou ausência dos três fatores de risco citados ( Figura 4 ).^256^ Caso haja alguma latência entre a estratificação inicial e a realização do transplante, sugerimos um período de 3 anos para a necessidade de nova estratificação, caso o paciente se mantenha estável e sem novos sintomas ou eventos CV.

Como elemento adicional da estratificação de risco de eventos CV no período perioperatório de transplante renal, recomendamos a dosagem de troponina (hs-Tn) em todos os pacientes antes do procedimento e seriados em 24 h e 48 h após o transplante.^257^ Um aumento absoluto da concentração de hs-Tn no 1º ou 2º dia pós-transplante comparado aos valores pré-operatórios caracteriza a ocorrência de injúria/infarto perioperatório. Na indisponibilidade de valores basais, um aumento significativo da concentração de hs-Tn no 1º dia pós-transplante (e.g., > 5 vezes o limite superior da normalidade) ou uma mudança significativa nessas concentrações entre o 1º e o 2º dia (aumento ou diminuição absoluta acima do limite superior da normalidade *versus* 1º dia) também definem a ocorrência de injúria/infarto perioperatório.

Finalmente, recomendamos a integração multidisciplinar na tomada de decisão sobre a investigação diagnóstica em pacientes assintomáticos candidatos a transplante renal e para a tomada de decisão frente ao achado de eventual DAC obstrutiva ("Heart-Kidney Team").^258^

O Quadro 17 apresenta as recomendações para avaliação do risco perioperatório em candidatos a transplante renal.

**Quadro 17:** Recomendações para avaliação do risco perioperatório em candidatos a transplante renal

Recomendação	Classe de recomendação	Nível de evidência
Todos os pacientes candidatos a transplante renal devem ser avaliados quanto à presença e gravidade da doença cardiovascular baseado na história clínica, exame físico e exames rotineiros	I	A
Pacientes sem fatores de risco principais * são considerados de baixo risco cardiovascular e podem ser liberados para o transplante renal sem a necessidade de investigação complementar	I	C
Recomenda-se a dosagem de troponina ultrassensível antes e 24 e 48 h após o transplante renal para detecção de injúria/infarto perioperatório	I	B
Recomenda-se que a tomada de decisão diagnóstica e a definição de estratégia terapêutica sejam discutidas por um "Heart-Kidney Team", incluindo cardiologista clínico, cardiologista intervencionista, cirurgião cardiovascular, nefrologista e/ou especialista em transplante renal	I	C
Pacientes com apenas um dos fatores de risco principais * são considerados de risco cardiovascular intermediário e devem ser submetidos à estratificação não invasiva. Se houver evidência de isquemia miocárdica induzida por estresse, prosseguir na investigação invasiva com cineangiocoronariografia; se não houver evidência de isquemia induzida por estresse ou outras alterações sugestivas da presença de doença arterial coronariana (DAC) (por exemplo, queda da fração de ejeção do ventrículo esquerdo), liberar para o transplante renal	IIa	C
Pacientes que apresentem pelo menos dois dos fatores de risco principais* são considerados de alto risco cardiovascular e deverão ser encaminhados diretamente para estudo invasivo antes do transplante renal	IIa	C
Pacientes estáveis com DAC obstrutiva devem ser reavaliados clinicamente quanto à progressão da doença a cada 12 meses; pacientes sem DAC obstrutiva significativa devem ser reavaliados a cada 36 meses para detecção de DAC de novo	IIa	C
Pacientes assintomáticos com doença coronária obstrutiva não devem ser encaminhados para revascularização miocárdica de rotina pela possibilidade de serem submetidos ao transplante ("intervenção profilática"), a menos que haja inequívoco impacto prognóstico da intervenção	III	A

* Idade > 50 anos, diabetes melito e evidência prévia de doença cardiovascular.

## 4. Medidas para Redução do Risco Cirúrgico do Ponto de Vista CV

### 4.1. Terapia Medicamentosa Perioperatória

#### 4.1.1. Betabloqueadores

Embora os estudos pioneiros sugerissem benefício dos betabloqueadores no que diz respeito à redução de eventos CV no perioperatório, este tema ainda permanece um tanto controverso, e a melhor conduta parece ser a individualização do tratamento.^259^ De fato, demonstrou-se que o benefício dos betabloqueadores estava diretamente relacionado ao risco cardíaco individual, sendo evidenciado benefício nos pacientes de alto risco, enquanto ele não foi visto naqueles de baixo risco.^260^ Nesse contexto, o estudo POISE ( *Effects of extended-release metoprolol succinate in patients undergoing non-cardiac surgery* ) avaliou o uso de betabloqueadores em pacientes de alto risco ou com doença aterosclerótica estabelecida e demonstrou redução de IAM, porém com aumento da incidência de acidente vascular encefálico e mortalidade total, provavelmente associados ao aumento das taxas de bradicardia e hipotensão.^261^ A maior crítica ao estudo se refere ao fato de o metoprolol ter sido introduzido 2 a 4 horas antes do procedimento cirúrgico, podendo chegar à dose de 400 mg por dia, o que é pouco utilizado na prática clínica e muito próximo da dose máxima recomendada pelos fabricantes. Dessa forma, não houve tempo hábil para a titulação mais adequada da medicação. Já quando iniciados com antecedência de pelo menos 1 semana, os betabloqueadores demonstraram redução de mortalidade a longo prazo em pacientes submetidos à cirurgia vascular.^262^

Dessa forma, de maneira geral, sugerimos que os betabloqueadores devem ser mantidos em paciente que façam uso crônico de alguma medicação dessa classe. Além disso, em pacientes que não fazem uso prévio de algum betabloqueador, ele não deve ser introduzido durante o período de 7 dias que antecedem o procedimento.

O Quadro 18 apresenta as recomendações para o manejo dos betabloqueadores no perioperatório.

**Quadro 18:** Recomendações para o manejo dos betabloqueadores no perioperatório

Recomendações	Classe de recomendação	Nível de evidência
Pacientes em uso crônico de betabloqueadores devem ter a medicação mantida no perioperatório	I	B
A introdução de betabloqueadores em pacientes sem uso prévio é contraindicada no intervalo de até 7 dias da data do procedimento cirúrgico	III	B

#### 4.1.2. Estatinas

As estatinas, além de reduzirem os níveis de colesterol, apresentam efeito pleiotrópico, reduzindo a inflamação e estabilizando as placas de aterosclerose. A utilização de estatinas para prevenção de eventos CV após operações vasculares está bem estabelecida, sendo baseada em estudos prospectivos, randomizados e controlados por placebo. Em 2004, foi publicado o primeiro estudo randomizado com 100 pacientes, no qual os autores demonstraram que o uso de 20 mg de atorvastatina estava associado a grande diminuição dos eventos CV maiores (morte, IAM, acidente vascular encefálico, angina instável) no perioperatório e ao final de 6 meses de seguimento. Tal efeito ocorreu independentemente dos níveis basais de colesterol.^263^ Em 2009, foi demonstrado que o uso de 80 mg de fluvastatina de liberação lenta em 250 pacientes submetidos a operações vasculares reduziu a ocorrência de isquemia miocárdica pós-operatória e o desfecho combinado de IAM e morte cardíaca em 30 dias, comparado ao grupo placebo (247 pacientes).^264^ Esses dados foram confirmados em uma metanálise envolvendo 23.536 pacientes, na qual o uso de estatina no perioperatório vascular reduziu a mortalidade geral e as taxas de IAM e acidente vascular encefálico.^265^ A introdução de atorvastatina 20 mg em pacientes que serão submetidos a operações vasculares deve ser feita, de preferência, 2 semanas antes do procedimento e mantida durante 30 dias. Após esse tempo, a dose deve ser ajustada para a meta de lipoproteína de baixa densidade individual de cada paciente.

Por outro lado, as evidências sobre o uso de estatinas para a prevenção de complicações CV em operações não vasculares são mais heterogêneas. Lindenauer et al.^266^ avaliaram 780.591 pacientes submetidos a operações não cardíacas (92% operações não vasculares) em um estudo de coorte retrospectiva, dos quais 77.082 (9,9%) receberam estatinas. Nesse estudo, os pacientes que receberam estatinas apresentaram menor mortalidade intra-hospitalar. Outro estudo retrospectivo, de caso-controle, somente com operações não vasculares, incluindo 989 casos de pacientes que morreram em até 30 dias do pós-operatório e 1.879 controles, demonstrou que o uso de estatinas também foi associado à redução de mortalidade (OR = 0,4; IC95% 0,24-0,68).^267^ Em uma coorte retrospectiva incluindo 752 pacientes submetidos a operações não vasculares, os autores demonstraram uma redução do desfecho composto de IAM não fatal, fibrilação atrial (FA) e mortalidade em 30 dias em pacientes que utilizaram estatina.^268^ Em uma análise dos pacientes incluídos no estudo VISION, Berwanger et al. avaliaram 2.842 pacientes recebendo estatina e 4.492 pacientes sem estatina e compararam a ocorrência de mortalidade, elevação isolada de troponina (definida como elevação de troponina, sem critérios de IAM e sem outra causa) ou acidente vascular encefálico em 30 dias utilizando o *propensity score matching* . Os pacientes que receberam estatinas apresentaram redução do risco do desfecho composto (risco relativo [RR] 0,83; IC95% 0,73-0,95; p = 0,007). O uso de estatinas reduziu a mortalidade geral (RR 0,58; IC95% 0,40-0,83; p = 0,003), a mortalidade CV (RR 0,42; IC95% 0,23-0,76; p = 0,004) e a ocorrência de elevação isolada de troponina (RR 0,86; IC95% 0,73-0,98; p = 0,02). Não houve redução na mortalidade não CV, na taxa de IAM ou acidente vascular encefálico. Cabe ressaltar que, apesar da análise com uso de *propensity score matching* , os pacientes do grupo estatina apresentavam mais frequentemente DAC, diabetes, doença vascular periférica, uso de AAS e de IECA/BRA em relação aos pacientes sem estatina. Assim, apesar de apresentarem mais fatores de risco, os pacientes do grupo estatina tiveram menos eventos CV.^269^ Em um estudo recente retrospectivo envolvendo 180.000 pacientes, o uso de estatina no dia da cirurgia ou no primeiro dia após a operação foi associado a uma redução na mortalidade em 30 dias^270^ Entretanto, o estudo randomizado LOAD, no qual 648 pacientes foram randomizados para receber atorvastatina 80mg antes da operação e 40 mg por 7 dias, não mostrou diferença no desfecho combinado de mortalidade geral, IAM não fatal e AVC em 30 dias ( *hazard ratio* [HR] 0,87, IC95% 0,60-1,26, p = 0,46).^271^ Cabe ressaltar que o estudo não tinha poder suficiente para determinar conclusões definitivas. Por outro lado, em uma metanálise de estudos randomizados, Putzu et al. demonstraram que o uso de estatinas no perioperatório estava associado a uma menor ocorrência de infarto perioperatório, sem redução na mortalidade.^272^ Assim, devido a resultados controversos, a prescrição de estatina visando à redução de complicações em pacientes submetidos a cirurgias não vasculares não é recomendada de rotina. Entretanto, os pacientes com indicação do uso de estatina pelas comorbidades (DAC, diabetes, doença vascular periférica) independentemente do contexto perioperatório, podem se beneficiar da introdução de estatina no perioperatório de operações não vasculares.

Frequentemente, as estatinas são suspensas no pós-operatório. As principais razões para a suspensão das estatinas são íleo pós-operatório e impossibilidade de administrar medicações por via oral, suspensão devido à instabilidade hemodinâmica, preocupação com a ocorrência de efeitos colaterais e falta de reconhecimento da importância de manter a estatina. A suspensão de estatina no perioperatório em pacientes que fazem uso crônico dessa medicação é um preditor independente de eventos CV após operações vasculares.^273 , 274^ O uso de estatinas no perioperatório é seguro. Embora pacientes que usam estatina apresentem um nível de creatinofosfoquinase (CPK) basal mais elevado, a ocorrência de elevação maior que cinco vezes o seu valor de referência ou de rabdomiólise é rara.^273^ Portanto, em pacientes que já utilizam, a estatina deve ser mantida durante todo o perioperatório.

O Quadro 19 apresenta as recomendações para o uso perioperatório das estatinas.

**Quadro 19:** Recomendações para o uso perioperatório das estatinas

Recomendação	Classe de recomendação	Nível de evidência
Pacientes que serão submetidos a operações vasculares arteriais	I	A
Manter em pacientes que já usam	I	B
Pacientes submetidos a operações não vasculares com indicação clínica do uso de estatinas devido a doenças associadas (doença arterial coronariana, doença cerebrovascular, doença arterial periférica, diabetes), independentemente do contexto do perioperatório	IIa	C

#### 4.1.3. Antiagregantes Plaquetários

Procedimentos cirúrgicos realizados na vigência de antiplaquetários estão relacionados a um aumento do risco de sangramento.^139 , 275^ Por outro lado, a sua suspensão está relacionada ao efeito rebote^276^ e ao aumento de eventos clínicos aterotrombóticos.^139 , 277^

##### 4.1.3.1. Monoterapia com Ácido Acetilsalicílico

Com relação ao uso do AAS, diversas evidências como o estudo POISE-2^275^ (que avaliou 10.010 pacientes, sendo 70% em prevenção primária) corroboram a suspensão do uso do AAS na prevenção primária 7 dias antes do procedimento cirúrgico e a sua não reintrodução no pós-operatório.

Já os pacientes que fazem uso de AAS para a prevenção secundária devem manter o seu uso durante todo o período perioperatório, pelo risco de efeito rebote e aumento do risco de eventos aterotrombóticos.^139 , 278 , 279^ As únicas exceções são as neurocirurgias, em que qualquer mínimo sangramento pode ser catastrófico, e as ressecções transuretrais de próstata,^139^ que, por não terem acesso à hemostasia direta, estão associados a maior sangramento. Nesses casos, o AAS deve ser suspenso 7 dias antes do procedimento. Entretanto, em pacientes em programação de ressecções transuretrais de próstata realizadas pela técnica *green light laser* , o AAS pode ser mantido.^280 , 281^ Além disso, não há recomendação para suspensão rotineira de AAS para a biópsia transretal da próstata, um procedimento urológico extremamente comum.^280^

Não há recomendação para iniciar o AAS antes de operações não cardíacas. Se avaliamos pacientes com doença vascular estabelecida, mas que erroneamente não vinham em uso de antiagregante, é opinião desta Diretriz, por consenso de especialistas, que, por ocasião da alta hospitalar, essa terapia seja planejada, mas, até o momento, não há estudo que embase o início antes da operação.

##### 4.1.3.2. Monoterapia Antiagregante com Outros Fármacos que Não o Ácido Acetilsalicílico

Embora o AAS ainda seja o antiagregante mais prescrito como monoterapia, muitos pacientes utilizam um substituto por apresentarem alergia ou intolerância gastrointestinal a esse medicamento, por terem AVC como antecedente ou seguindo as novas diretrizes de síndrome coronariana.^282^ Para esses pacientes, caso o antigregante plaquetário em monoterapia seja o clopidogrel, o risco de sangramento durante cirurgias é aumentado.^140^ Por outro lado, não há estudos que demonstrem a segurança da suspensão em termos de evento CV. Portanto, as evidências relativas ao AAS, com relação à manutenção ou suspensão do medicamento, não podem ser extrapoladas para o clopidogrel.

Os especialistas envolvidos na elaboração desta Diretriz optaram por recomendar que o clopidogrel seja mantido no período perioperatório de procedimentos cirúrgicos não cardíacos associados a baixo risco de sangramento, como no caso das intervenções dermatológicas. Por outro lado, no caso de cirurgias de risco moderado ou alto de sangramento, o clopidogrel deve ser suspenso 5 dias antes da operação.

Se a monoterapia antigregante estiver sendo realizada com o uso de ticagrelor, esse medicamento deve ser suspenso 5 dias antes da operação. No caso do prasugrel, a suspensão deve ocorrer 7 dias antes da operação. Para pacientes em uso de ticagrelor e prasugrel que serão submetidos a procedimentos cirúrgicos não cardíacos associados a baixo risco de sangramento, como no caso das intervenções dermatológicas, não existem evidências científicas suficientes. Sugerimos que seja analisado cada caso individualmente de maneira multidisciplinar.

O Quadro 20 apresenta as recomendações para o manejo dos antiagregantes plaquetários no perioperatório em pacientes em uso de monoterapia.

**Quadro 20:** Recomendações para o manejo dos antiagregantes plaquetários no perioperatório em pacientes em uso de monoterapia

Recomendação	Classe de recomendação	Nível de evidência
Para pacientes em uso de ácido acetilsalicílico, no contexto de prevenção primária, ele deve ser suspenso 7 dias antes de operação não cardíaca	I	A
Para pacientes em uso de ácido acetilsalicílico, no contexto de prevenção secundária, ele deve ser mantido na dose de 100 mg ao dia durante todo o perioperatório, exceto para neurocirurgias ou procedimentos de risco hemorrágico proibitivo	I	A
Para pacientes em uso de monoterapia com clopidogrel, ele pode ser mantido em procedimentos de baixo risco de sangramento e deve ser suspenso 5 dias antes em procedimentos de risco moderado ou alto de sangramento	IIb	C
Para pacientes em uso de monoterapia com ticagrelor, ele deve ser suspenso 5 dias antes da operação	IIb	C
Para pacientes em uso de monoterapia com prasugrel, ele deve ser suspenso 7 dias antes da operação	IIb	C

##### 4.1.3.3. Dupla Antiagregação Plaquetária (DAP)

A atualização do manejo dos antiagregantes plaquetários em pacientes com DAP deve ser consultada na diretriz específica "Foco em Manejo dos Pacientes com Intervenção Coronária Percutânea – 2022". Abaixo, é apresentado o sumário das recomendações.^144^

O Quadro 21 apresenta as recomendações para o manejo dos antiagregantes plaquetários no perioperatório em pacientes em uso de DAP.

**Quadro 21:** Recomendações para o manejo dos antiagregantes plaquetários no perioperatório em pacientes em uso de DAP

Recomendação	Classe de recomendação	Nível de evidência
O ácido acetilsalicílico deve ser mantido na dose de 100 mg ao dia durante todo o perioperatório, exceto para neurocirurgias ou procedimentos de risco hemorrágico proibitivo	I	A
Suspender clopidogrel e ticagrelor 5 dias antes de cirurgias não cardíacas	I	B
Suspender prasugrel 7 dias antes de cirurgias não cardíacas	I	B
Em caso de interrupção de dupla antiagregação plaquetária (DAP) antes do tempo mínimo ideal, realizar cirurgias não cardíacas em centros com suporte multidisciplinar e retaguarda hemodinâmica	I	C
Realizar cirurgias de baixo risco de sangramento na vigência de DAP se o intervalo decorrido desde a angioplastia for menor do que 3 meses	IIa	C
Utilizar teste de agregabilidade plaquetária para abreviar tempo de suspensão de inibidor de P2Y12 antes de cirurgias não cardíacas	IIb	B
Para casos de risco trombótico muito elevado (menos de 1 mês de intervenção coronária percutânea e interrupção de DAP), utilizar tirofiban como terapia de ponte	IIb	B
Realizar terapia de ponte com heparina de baixo peso molecular	III	B
Utilizar teste de agregabilidade plaquetária como rotina para avaliar suspensão de ácido acetilsalicílico ou de inibidor de P2Y12 antes de cirurgias não cardíacas	III	C

#### 4.2. Revascularização Miocárdica

Evidências mais recentes na literatura não conseguiram demonstrar o papel benéfico da revascularização miocárdica profilática em pacientes com DAC estável no pré-operatório de cirurgias não cardíacas.^71 , 283^ Somam-se a tal fato o desenvolvimento da terapêutica medicamentosa e a consequente farmacoproteção perioperatória, que tornam as potenciais situações de benefício da revascularização miocárdica profilática cada vez mais restritas. Assim, as indicações de revascularização miocárdica no pré-operatório de cirurgias não cardíacas são idênticas àquelas fora do contexto perioperatório e visam à melhora do prognóstico a longo prazo, não objetivando apenas a redução de eventos isquêmicos perioperatórios.

Nos casos com indicação inequívoca de revascularização miocárdica em pacientes que se encontram no pré-operatório de cirurgias não cardíacas, dados como a estabilidade clínica do paciente e o prognóstico da doença de base que levou à indicação do procedimento cirúrgico, além do potencial risco de sangramento do procedimento, devem ser levados em consideração na tomada de decisão. Em relação ao tempo entre a revascularização miocárdica cirúrgica e a operação não cardíaca, não há estudos específicos. Portanto, o tempo ideal refere-se ao tempo necessário para a recuperação física do paciente após uma operação cardíaca (cerca de 30 dias), sendo que não há um tempo mínimo estabelecido, sendo necessária uma avaliação individualizada considerando o estado clínico geral do paciente e a urgência da operação não cardíaca.

Por outro lado, o intervalo entre a revascularização miocárdica e a operação não cardíaca é um fator importante em situações de angioplastia.

A atualização sobre as novas evidências em relação ao intervalo de segurança entre a revascularização miocárdica e a operação não cardíaca pode ser consultada na diretriz específica "Foco em Manejo dos Pacientes com Intervenção Coronária Percutânea – 2022". Abaixo, é apresentado um resumo das recomendações. As Classes de recomendação indicadas por esta Diretriz refletem as evidências disponíveis, o bom senso e a experiência clínica, em especial no que diz respeito à urgência da operação não cardíaca. Por essa razão, intervalos menores receberam Classes de recomendação menos fortes e são justificáveis na presença de justificativas individualizadas como urgência da operação não cardíaca.^144^

O Quadro 22 apresenta as recomendações para o intervalo entre a revascularização miocárdica e a operação não cardíaca em pacientes submetidos a intervenções coronárias percutâneas eletivas.

**Quadro 22:** Recomendações para o intervalo entre a revascularização miocárdica e a operação não cardíaca em pacientes submetidos a intervenções coronárias percutâneas eletivas

Recomendação	Classe de recomendação	Nível de evidência
≥ 6 meses	I	A
Entre 3 e 6 meses	IIa	B
Entre 30 dias e 3 meses	IIb	B
< 30 dias	III	B

O Quadro 23 apresenta as recomendações para o intervalo entre a revascularização miocárdica e a operação não cardíaca em pacientes submetidos a intervenções coronárias percutâneas devido a síndromes coronarianas agudas.

**Quadro 23:** Recomendações para o intervalo entre a revascularização miocárdica e a operação não cardíaca em pacientes submetidos a intervenções coronárias percutâneas devido a síndromes coronarianas agudas

Recomendação	Classe de recomendação	Nível de evidência
≥ 12 meses	I	A
Entre 6 e 12 meses	IIa	B
Entre 30 dias e 6 meses	IIb	B
< 30 dias	III	B

### 4.3. Manejo da Anticoagulação no Perioperatório

A anticoagulação de pacientes é uma situação cada vez mais frequente na prática clínica, seja para pacientes com FA, prevenção ou tratamento do tromboembolismo venoso (TEV) ou para pacientes portadores de próteses mecânicas cardíacas. Estima-se que um quarto desses pacientes precisarão de uma interrupção temporária da anticoagulação para uma intervenção cirúrgica programada dentro dos próximos 2 anos após o início da terapia.^284^ O grande desafio do manejo de anticoagulantes no perioperatório está no fato de que a interrupção da anticoagulação aumenta temporariamente o risco tromboembólico, assim como a sua manutenção, na vigência de procedimentos invasivos, pode aumentar o risco de complicações hemorrágicas, e ambas aumentam o risco de morte.^285 - 288^ Ao avaliar o risco tromboembólico dentro do espectro da avaliação perioperatória, é necessário reconhecer as diferentes situações de risco de tromboembolismo. Uma delas é a do paciente recebendo anticoagulação para a prevenção de TEV; a outra é a dos pacientes que recebem anticoagulação na vigência de próteses mecânicas cardíacas e/ou FA para a prevenção do tromboembolismo arterial. A Tabela 11 representa uma proposta para a estratificação de risco desses pacientes, sendo considerados de risco alto aqueles com > 10% de risco anual de tromboembolismo, risco moderado aqueles com 5 a 10% e baixo risco aqueles com < 5%.^289 - 291^ Os pacientes portadores de próteses mecânicas valvares podem ser considerados de alto risco de tromboembolismo.

**Tabela 11:** Estratificação de risco para o tromboembolismo

Categoria de risco	Fibrilação atrial	TEV
Alto *	Escore CHADS_2_ de 5 ou 6 AVC ou AIT recente (< 3 meses) Doença valvar reumática	TEV recente (< 3 meses) Trombofilia grave †
Moderado	Escore CHADS_2_ de 3 ou 4	TEV há 3-12 meses Trombofilia leve ‡ Novo TEV Neoplasia ativa
Baixo	Escore CHADS_2_ de 0 a 2 (sem AVC ou AIT prévio)	TEV > 12 meses sem outros fatores de risco

Escore CHADS2: Insuficiência cardíaca = 1 ponto; HAS = 1 ponto; idade > 75 anos = 1 ponto; DM = 1 ponto; AVC/AIT = 2 pontos.

* Pacientes de alto risco também podem incluir aqueles com AVC ou AIT > 3 meses antes da cirurgia planejada e escore CHADS2 < 5, aqueles que cursaram com tromboembolismo durante a interrupção temporária da anticoagulação ou aqueles submetidos a certos tipos de cirurgia associadas a um alto risco de AVC ou outro tipo de tromboembolismo (cirurgia de troca de valva cardíaca, endarterectomia de carótida e grandes cirurgias vasculares).

† Trombofilia grave: deficiência de proteína C, S, antitrombina ou presença de anticorpos antifosfolípide.

‡ Trombofilia leve: mutação heterozigótica do fator V de Leiden ou do gene da protrombina. TEV: tromboembolismo venoso; AVC: acidente vascular

Além de avaliar o risco tromboembólico, temos que considerar o risco de sangramento que certos procedimentos cirúrgicos apresentam na vigência do uso de medicações antitrombóticas. Na Tabela 12 , apresentamos o risco de sangramento associado a cada tipo de procedimento cirúrgico.^286^ De forma geral, dividimos os procedimentos naqueles com alto risco de sangramento grave no período de 2 a 4 dias (2 a 4%) e aqueles com baixo risco (0 a 2%). Sangramento grave é geralmente definido como um sangramento que leve à morte, intracraniano, que necessite de reoperação para ser controlado, que leve à queda na hemoglobina ≥ 2 g/dL ou que necessite de transfusão de ≥ 2 unidades de hemácias.^292^

**Tabela 12:** Risco de sangramento conforme o procedimento cirúrgico

Alto risco (risco de sangramento maior em 2 dias entre 2 e 4%)	Cirurgia de aneurisma de aorta abdominal Qualquer grande cirurgia (duração > 45 minutos) Cirurgia de prótese de joelho bilateral Procedimentos de aspiração por agulha fina guiados endoscopicamente Biópsia renal Laminectomia Urológica, de cabeça e pescoço, abdominal, neurocirurgia, câncer de mama Polipectomia, varizes de esôfago, esfincterectomia biliar, dilatação pneumática Ressecção transuretral de próstata
Baixo risco (risco de sangramento maior em 2 dias entre 0-2%)	Hernioplastia abdominal Histerectomia abdominal Dissecção de nódulo axilar Broncoscopia com ou sem biópsia Cirurgia do túnel do carpo Cirurgia oftalmológica Remoção de cateter venoso central Colecistectomia Biópsias cutâneas, de bexiga, próstata, mama, tireoide e de linfonodos Dilatação e curetagem Endoscopia gastrointestinal com ou sem biópsia, enteroscopia, stent biliar ou pancreático sem esfincterectomia Cirurgia de hemorroida Cirurgia de hidrocele Cirurgia de prótese de joelho ou quadril, mão, ombro, pé e artroscopia Angiografia não coronariana Extrações e outras cirurgias dentárias

Além de avaliar o risco de sangramento baseado no tipo de procedimento cirúrgico que será realizado, existem condições clínicas inerentes a cada paciente que podem conferir um risco ainda maior de sangramento. Existem escores que podem quantificar o risco de sangramento baseados nas características clínicas dos pacientes submetidos à terapia anticoagulante, como é o caso do HAS-BLED escore.^293^ Um escore HAS-BLED ≥ 3 está associado a maior risco de sangramento (HR 11,8, IC95% 5,6-24,9).

Alguns procedimentos são associados a um mínimo risco de sangramento e podem ser realizados sem a interrupção da terapia anticoagulante, como é o caso de implante de marca-passo/desfibrilador implantável, angiografia coronária, procedimentos dentários menores (extração de até dois dentes, restaurações, próteses, endodontia, limpezas e implantes), cirurgia de catarata, procedimentos dermatológicos menores, artrocentese ou injeção intra-articular.^294^

#### 4.3.1. Varfarina^289 - 291 , 295^

**Figura 5:**
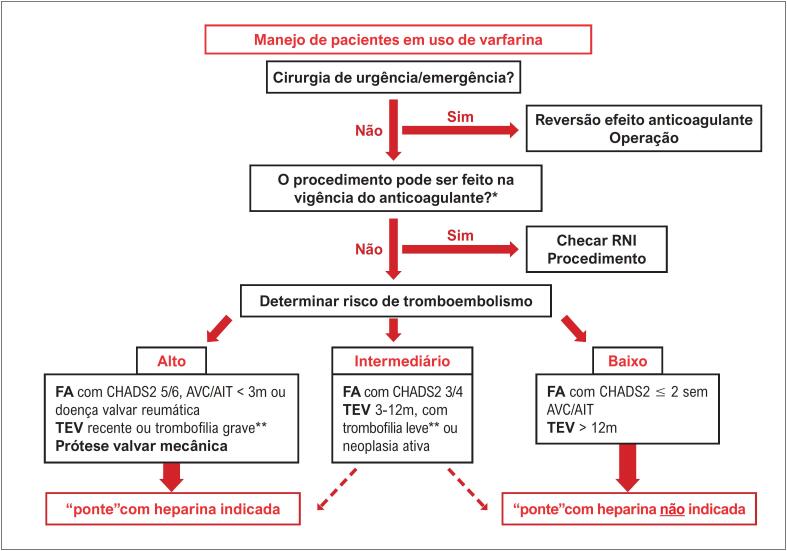
Manejo perioperatório de pacientes anticoagulados com varfarina. *Vide item 3.3. *Trombofilia grave: deficiência de proteína C, S, antitrombina ou presença de anticorpos antifosfolípide.**Trombofilia leve: mutação heterozigótica do fator V de Leiden ou do gene da protrombina. RNI: razão normalizada internacional.

A varfarina é um antagonista da vitamina K, cujo efeito anticoagulante demora dias para desaparecer (meia-vida de 36 a 42 horas), assim como pode exigir tempo semelhante para ser novamente atingido depois da cirurgia. O fluxograma para o manejo de pacientes em uso de varfarina no perioperatório pode ser consultado na Figura 5 .

Pacientes com alto risco para tromboembolismo podem necessitar da terapia de ponte com agentes anticoagulantes parenterais, como a heparina não fracionada, ou por via subcutânea, como as heparinas de baixo peso molecular. Esses dois agentes apresentam início de ação mais rápido, assim como meia-vida mais curta, o que proporcionaria a possibilidade de suspender o anticoagulante o mais próximo possível do procedimento cirúrgico, minimizando ao máximo o risco tromboembólico. Como o metabolismo da varfarina pode sofrer a influência de vários fatores como idade do paciente, função renal e interações medicamentosas, sugere-se medir a RNI no dia anterior à cirurgia para se assegurar que esteja < 1,5 e, caso contrário, ter tempo hábil para a sua reversão com a administração de vitamina K por via oral (1 a 2 mg) e reavaliar a RNI no dia seguinte.

A opção de realizar ou não a terapia de "ponte" em pacientes em uso de varfarina depende de análise conjunta do risco tromboembólico ( Tabela 11 ), do risco de hemorragia ( Tabela 12 ) e do do próprio paciente ( Figura 5 ). O objetivo da "ponte" com heparina é minimizar o tempo que o paciente não recebe anticoagulação, visando diminuir o risco de tromboembolismo. Por outro lado, estudos observacionais e metanálises demonstraram que a realização da "ponte" com heparina pode estar relacionada com aumento de sangramento.^296 , 297^ No estudo BRIDGE, 1.884 pacientes foram randomizados para realizar ou não ponte com heparina, e o uso da "ponte" não reduziu eventos trombóticos significativamente, mas aumentou os sangramentos maiores. Vale ressaltar que o CHA_2_DS_2_ médio dos pacientes incluídos neste estudo foi 2,3 (uma população de menor risco trombótico), o que poderia indicar que, para pacientes mais graves, com risco trombótico mais elevado (CHA_2_DS_2_ maior que 4), os resultados poderiam ser diferentes.^298^ Podemos concluir que a "ponte" com heparina não deve ser realizada indiscriminadamente para todos os pacientes anticoagulados.

##### 4.3.1.1. Pacientes com Risco Moderado para Tromboembolismo

Existem poucas evidências sobre qual a melhor conduta nos pacientes com risco moderado de tromboembolismo no que se refere a usar ou não a terapia de ponte. Assim, a escolha deve se embasar em características individuais de cada paciente e da cirurgia proposta, indicando a terapia de ponte somente em casos específicos, a critério do médico assistente.

##### 4.3.1.2. Procedimentos de Urgência ou Emergência

As medidas terapêuticas empregadas para a reversão da anticoagulação oral com varfarina dependem da rapidez com que se necessite a normalização do tempo de protrombina (TP) medido pela RNI. No caso de cirurgias que possam aguardar de 18 a 24 horas, a suspensão da varfarina associada ao uso da vitamina K, na dose de 2,5 a 5 mg por via venosa, geralmente produz a normalização da RNI, quando se encontra dentro da faixa terapêutica.^289 - 291^

No caso da necessidade da rápida normalização da RNI, é necessário repor os fatores de coagulação deficientes, estando o plasma fresco congelado (PFC) e o concentrado de complexo protrombínico disponíveis para isso. A resolução 10, de 23 de janeiro de 2004, da Agência de Vigilância Sanitária (ANVISA) determina que, "para a correção de hemorragia por uso de anticoagulantes cumarínicos ou reversão rápida dos efeitos dos cumarínicos (…)", o produto de escolha é o complexo protrombínico. Como a disponibilidade desse tipo de concentrado ainda não é suficientemente ampla nos hospitais brasileiros, o uso do PFC é uma alternativa aceitável.^299^ No caso do PFC, a dose preconizada é de 15 mL por quilo de peso, devendo-se evitar a sobrecarga de volume.^300^ Para o complexo protrombínico, ainda não existe padronização a ser empregada. Na Tabela 13 , está esquematizado o que é utilizado em alguns serviços ingleses, porém, independentemente do que for utilizado para repor os fatores dependentes da vitamina K, é necessário o uso associado da vitamina K1 (2,5-5,0 mg por via oral ou venosa lenta), para a manutenção dos valores normais de protrombina durante o período perioperatório.^289 - 291^

**Tabela 13:** Dose de concentrado de complexo protrombínico a ser administrado para a reversão da anticoagulação, conforme o valor da razão normalizada internacional (RNI)

RNI	Dose tomando-se como base o fator IX
2,0-3,9	25 U/kg
4,0-5,9	35 U/kg
≥ 6,0	50 U/kg

**Quadro 24:** Recomendações para pacientes anticoagulados com varfarina submetidos a cirurgias de urgência/emergência

Recomendação	Classe de recomendação	Nível de evidência
Suspensão do medicamento anticoagulante, administração de vitamina K por via venosa e reposição dos fatores deficientes com complexo protrombínico ou plasma fresco congelado, de acordo com a disponibilidade desses produtos	I	C

**Quadro 25:** Recomendações para pacientes anticoagulados com varfarina

Recomendações para pacientes com alto risco para tromboembolismo e pacientes portadores de próteses valvares mecânicas	Classe de recomendação	Nível de evidência
Interromper a varfarina 5 dias antes da cirurgia e aguardar razão normalizada internacional < 1,5	I	C
Realizar terapia de ponte com heparina não fracionada ou de baixo peso molecular em dose plena quando razão normalizada internacional < 2	IIa	C
Suspender a heparina não fracionada 4 a 6 horas antes do procedimento e a heparina de baixo peso molecular 24 horas antes	IIa	C
No pós-operatório, reiniciar heparina não fracionada ou de baixo peso molecular em dose plena e a varfarina pelo menos 24 horas após o procedimento cirúrgico e suspender a heparina somente quando a razão normalizada internacional estiver dentro da faixa terapêutica	IIa	C
Em pacientes submetidos a cirurgias com alto risco de hemorragia, reiniciar a heparina de baixo peso molecular 48 a 72 horas após a cirurgia	IIa	C
**Recomendações para pacientes com baixo risco de tromboembolismo**	**Grau de recomendação**	**Nível de evidência**
Não utilizar a terapia de ponte (suspender varfarina 5 dias antes da cirurgia e aguardar razão normalizada internacional < 1,5 para a realização do procedimento)	IIa	C
No pré-operatório, podem ser usadas a heparina não fracionada ou a de baixo peso molecular profilática, se indicado	IIa	C
No pós-operatório, se indicado, usar heparina não fracionada ou de baixo peso molecular profilática e reiniciar a varfarina de 12 a 24 horas após o procedimento	IIa	C

**Figura 6:**
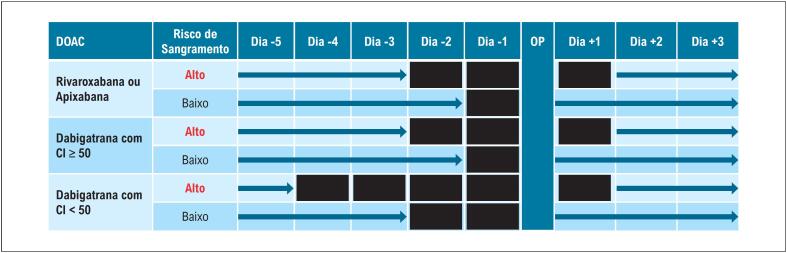
Esquema para manejo dos DOACs no perioperatório. Cl: clearence de creatinina; OP: operação; DOAC: anticoagulante oral direto. Os quadrados pretos indicam os dias nos quais o paciente não deve receber os DOACs. Alto risco de sangramento: qualquer operação requerendo anestesia neuroaxial ou epidural, neurocirurgia, cirurgia torácica major (lobectomia, pneumectomia, esofagectomia), cirurgia vascular major (aorta, revascularização membros inferiores, endarterectomia de carótida), cirurgia abdominal e pélvica major (ressecção de neoplasia hepatobiliar, câncer de pâncreas ou pseudocisto, neoplasia gástrica e colorretal, ressecção de doença inflamatória intestinal, neoplasia renal, de bexiga, endométrio ou ovário, prostatectomia radical), cirurgia ortopédica major (artroplastia de quadril ou fratura de quadril, artroplastia de joelho, osteotomia metatarsal), outras cirurgias major para câncer ou reconstrutivas (câncer de cabeça e pescoço).

**Quadro 26:** Recomendações para manejo dos anticoagulantes orais diretos (DOACs)

Recomendações para pacientes em uso crônico de dabigatrana	Classe de recomendação	Nível de evidência
Pacientes com função renal normal e em cirurgias com baixo risco de sangramento podem ter a medicação suspensa 24 horas antes da cirurgia e reintroduzida 24 horas após a cirurgia	I	B
Pacientes com função renal normal e em cirurgias com alto risco de sangramento podem ter a medicação suspensa 48 horas antes da cirurgia e reintroduzida 48 horas após a cirurgia	I	B
Pacientes com disfunção renal ( *clearance* < 50 mL/min) e em cirurgias com baixo risco de sangramento podem ter a medicação suspensa 48 horas antes da cirurgia e reintroduzida 24 horas após a cirurgia	I	B
Pacientes com disfunção renal ( *clearance* < 50 mL/min) e em cirurgias com alto risco de sangramento podem ter a medicação suspensa 96 horas antes da cirurgia e reintroduzida 48 horas após a cirurgia	I	B
Nos casos de anestesia regional com cateter epidural, esperar ao menos 6 horas após a retirada do cateter para iniciar a primeira dose de dabigatrana	I	B
**Recomendações para pacientes em uso crônico de rivaroxabana**	**Classe de recomendação**	**Nível de evidência**
Pacientes com função renal normal podem ter a medicação suspensa 24 horas antes da cirurgia e reintroduzida 24 horas após a cirurgia	I	B
Nos casos de disfunção renal grave ( *clearance* de creatinina 15 a 30 mL/minuto) ou em cirurgias com alto risco de sangramento, a rivaroxabana deve ser suspensa pelo menos 48 horas antes da intervenção e reintroduzida 48 horas após a cirurgia	I	B
Nos casos de anestesia regional com cateter epidural, aguardar pelo menos 6 horas após a retirada do cateter para a próxima dose de rivaroxabana	I	B
**Recomendações para pacientes em uso crônico de apixabana**	**Classe de recomendação**	**Nível de evidência**
Pacientes com função renal normal podem ter a medicação suspensa 24 horas antes da cirurgia e reintroduzida 24 horas após a cirurgia	I	B
Nos casos de disfunção renal moderada ( *clearance* de creatinina 15 a 50 mL/minutos) ou cirurgias com alto risco de sangramento, a apixabana deve ser suspensa pelo menos 48 horas antes da intervenção e reintroduzida 48 horas após a cirurgia	I	B
Nos casos de anestesia regional com cateter epidural, aguardar pelo menos 6 horas após a retirada do cateter para a próxima dose de apixabana	I	B

O Quadro 24 apresenta as recomendações para pacientes anticoagulados com varfarina submetidos a cirurgias de urgência/emergência.

O Quadro 25 apresenta as recomendações para pacientes anticoagulados com varfarina.

#### 4.3.2. Anticoagulantes Diretos

A partir de 2010, o desenvolvimento de anticoagulantes com ação direta na cascata da coagulação, chamados anticoagulantes orais diretos (DOACs), trouxe uma alternativa promissora para até então os únicos anticoagulantes disponíveis, que eram os antagonistas da vitamina K.^301^ Eles têm uma maior facilidade posológica, doses preestabelecidas, maior estabilidade farmacocinética e menores interações medicamentosas e com a alimentação, sem necessidade de controles seriados de TP/RNI. São medicamentos que foram aprovados para uso na prevenção de AVC em pacientes com FA não valvar, no tratamento do TEV (trombose venosa profunda/tromboembolismo pulmonar), na prevenção do TEV recorrente e do TEV nas grandes cirurgias ortopédicas.

O termo FA não valvar gerou muita confusão, mas basicamente os ensaios excluíam portadores de próteses mecânicas e estenose mitral moderada e grave, deixando muitas dúvidas com relação aos outros tipos de valvopatias. Atualmente, com os novos ensaios disponíveis, os DOACs estão autorizados na indicação de anticoagulação em pacientes portadores das seguintes valvopatias: insuficiência aórtica, insuficiência mitral, estenose aórtica e estenose mitral discreta, sendo que pacientes portadores de biopróteses também podem usar DOACs no lugar da varfarina. Em pacientes submetidos a TAVI, o uso de DOAC está autorizado de forma isolada, quando indicada a anticoagulação. Se não houver indicação de anticoagulação, não é recomendado substituir o antiagregante pelo DOAC.

Para pacientes portadores de estenose mitral moderada a importante e para aqueles com próteses mecânicas, até o momento com as publicações disponíveis, o uso dos DOACs está contraindicado. Com relação a estenose mitral moderada e importante, o estudo INVICTUS demonstrou que a varfarina é superior a rivaroxabana em pacientes com doença reumática (redução de eventos CV e mortalidade), permanecendo a medicação de escolha nessa população.^302^ Com relação ao uso dos DOACs no cenário de prótese mecânica em posição aórtica, o ensaio PROACT-Xa foi finalizado precocemente devido ao aumento de eventos tromboembólicos com o uso de apixabana.

##### 4.3.2.1. Dabigatrana^295 , 303 - 306^

A dabigatrana é um medicamento anticoagulante que age como inibidor direto da trombina, bloqueando, de maneira reversível, a conversão do fibrinogênio em fibrina (fator Iia). É um medicamento com rápido início de ação, pico de concentração entre 30 e 120 minutos, meia-vida de 12-17 horas e excreção predominantemente renal (80%). Devido ao seu rápido início de ação e meia-vida mais curta, não há a necessidade da terapia de ponte associada a tal medicamento. Uma das preocupações associadas ao uso da dabigatrana está relacionada à ausência de antídotos específicos até bem pouco tempo atrás, quando as possibilidades disponíveis se limitavam ao uso do complexo protrombínico e hemodiálise, que apresentavam sucesso limitado. O primeiro antídoto contra os inibidores diretos da trombina (dabigatrana), o idarucizumab, foi aprovado pela Food and Drug Administration (FDA) dos Estado Unidos em outubro de 2015. O idarucizumab é um fragmento de anticorpo monoclonal que se liga tanto com a dabigatrana livre quanto à ligada a trombina com uma afinidade mais alta do que a afinidade da dabigatrana pelo fator II, tendo revertido completamente o efeito anticoagulante da dabigatrana. A dose é de 5 g via endovenosa em duas aplicações de 2,5/50 mL *bolus* dados com intervalo de 15 minutos.^307 , 3^

##### 4.3.2.2. Rivaroxabana^286 , 295 , 303 , 304 , 306^

A rivaroxabana é um medicamento que age como um agente inibidor do fator Xa, bloqueando, dessa maneira, a conversão da protrombina em trombina. É também uma substância com rápido início de ação, com pico de concentração entre 2 e 4 horas, meia-vida curta (5 e 9 horas em jovens e 11 e 13 horas em idosos), metabolização hepática e excreção renal (66%). Também, devido ao seu rápido início de ação e meia-vida mais curta, não é necessária a terapia de ponte associada a esse medicamento. Antigamente, só se dispunha do complexo protrombínico para a tentativa de reversão do efeito da rivaroxabana, uma vez que não havia antídotos específicos disponíveis. Atualmente, o andexanet alfa é um antídoto específico contra os inibidores do fator Xa, se ligando ao sítio ativo dos inibidores do fator Xa, com rápida reversão do efeito anticoagulante da apixabana e da rivaroxabana em minutos. O andexanet alfa pode ser administrado em uma alta dose de 800 mg via endovenosa seguido de 8 mg/min por 120 minutos ou em uma baixa dose de 400 mg via endovenosa seguido de 4 mg/min por 120 minutos.^308^

##### 4.3.2.3. Apixabana^286 , 295 , 304 , 306^

A apixabana é um medicamento que também é um inibidor do fator Xa, bloqueando a conversão da protrombina em trombina. Tem rápido início de ação, pico de concentração em 3 horas, meia-vida curta (8 a 15 horas), metabolização hepática e excreção renal (27%) e fecal. Também, por conta de seu rápido início de ação e meia-vida mais curta, não há necessidade da terapia de ponte associada a ela. Atualmente, o Andexanet alfa é o antídoto específico contra os inibidores do fator Xa, mostrando rápida reversão do efeito anticoagulante da apixabana e da rivaroxabana em minutos.^308^

##### 4.3.2.4. Edoxabana^309^

A edoxabana também é um agente inibidor do fator Xa. Tem um rápido início de ação, pico de concentração em 1 a 2 horas, meia-vida curta (10 a 14 horas) e excreção renal (50%) e por via biliar e intestinal (50%). Também, por conta de seu rápido início de ação e meia-vida mais curta, não há a necessidade da terapia de ponte associada a ela. Apesar de ter sido o mais recente DOAC utilizado, o uso do Andexanet alfa também é uma possibilidade de antídoto para essa droga.^310^

##### 4.3.2.5. Avaliação do Efeito Anticoagulante dos DOACs

Embora não exista um exame preciso universal para determinar o efeito anticoagulante dos DOACs, os testes de coagulação convencionais podem ajudar a determinar se existe alguma ação do medicamento, sendo testes qualitativos e não quantitativos. Em uma situação de urgência, eles podem ser utilizados. Para a dabigatrana, utiliza-se o tempo de trombina e o tempo de tromboplastina parcial ativada, sendo que, se estiverem aumentados, significa que a droga ainda está ativa. Para os inibidores do fator Xa, utiliza-se a atividade da protrombina ou o TP, sendo que a RNI não deve ser utilizada.

##### 4.3.2.6. Tempo Ideal para Suspensão dos DOACs Antes de Cirurgias Eletivas

As diretrizes passadas se baseavam na farmacocinética dos DOACs para estimar o tempo ideal para a sua suspensão antes de cirurgias eletivas, avaliando dados de meia-vida, grau de eliminação renal e função renal do paciente. Contudo, em 2019, foi publicado o estudo PAUSE^311^, em que foram incluídos 3.007 pacientes com FA em uso de dabigatrana, rivaroxabana ou apixabana, e foi realizado um protocolo fixo de suspensão e reintrodução do DOAC baseado na farmacocinética das medicações, função renal do paciente e risco de sangramento associado ao procedimento cirúrgico, sem utilização de testes de coagulação de rotina ou de "ponte" com heparina. Nesse estudo, essa estratégia esteve associada a baixos níveis de eventos tromboembólicos e sangramentos, podendo ser extrapolada para a prática clínica ( Figura 6 ). Várias diretrizes incorporaram o protocolo do estudo PAUSE nas suas orientações para a suspensão de DOACs antes de procedimentos cirúrgicos.^312 , 313^ Nos pacientes que vão ser submetidos a procedimentos cirúrgicos com baixo risco de sangramento, o DOAC pode ser suspenso 1 dia antes da cirurgia, e, naqueles que serão submetidos a procedimentos com alto risco de sangramento, o DOAC deve ser suspenso 2 dias antes da cirurgia. São considerados procedimentos com alto risco de sangramento: qualquer operação requerendo anestesia neuroaxial ou epidural, neurocirurgia, cirurgia torácica *major* (lobectomia, pneumectomia, esofagectomia), cirurgia vascular *major* (aorta, revascularização membros inferiores, endarterectomia de carótida), cirurgia abdominal e pélvica *major* (ressecção de neoplasia hepatobiliar, câncer de pâncreas ou pseudocisto, neoplasia gástrica e colorretal, ressecção de doença inflamatória intestinal, neoplasia renal, de bexiga, endométrio ou ovário, prostatectomia radical), cirurgia ortopédica *major* (artroplastia de quadril ou fratura de quadril, artroplastia de joelho, osteotomia metatarsal), outras cirurgias *major* para câncer ou reconstrutivas (câncer de cabeça e pescoço).

Com relação especificamente à dabigatrana, devido à sua alta taxa de excreção renal, se o *clearance* de creatinina for menor que 50 mL/min, ela deve ser suspensa 2 dias antes das cirurgias com baixo risco de sangramento e 4 dias antes das cirurgias com alto risco de sangramento. Nos casos de anestesia regional com cateter peridural, deve-se aguardar pelo menos 6 horas após a retirada do cateter para a próxima dose do DOAC.

Com relação à edoxabana, por ter sido o DOAC mais recente a ter sido lançado, os estudos avaliando seu manejo no contexto perioperatório são muito limitados. A princípio, a conduta mais aceita é a interrupção da edoxabana 24 horas antes das cirurgias com baixo risco de sangramento e a interrupção 48 a 72 horas antes das cirurgias associadas a um alto risco de sangramento.

É aconselhável, baseado em várias diretrizes,^312^ iniciar dose profilática de heparina de baixo peso molecular 12 h após o procedimento cirúrgico (se adequada hemostasia) em pacientes/procedimentos com alto risco de TEV. Recomendamos parar a dose profilática de heparina de baixo peso molecular concomitantemente à reintrodução da anticoagulação oral.

O Quadro 26 apresenta as recomendações para manejo dos DOACs.

#### 4.4. Profilaxia de Endocardite Infecciosa

A endocardite infecciosa (EI) é uma doença de elevadas morbidade e mortalidade.^314 , 315^ As estratégias para reduzir sua incidência se baseiam predominantemente em modelos experimentais, em estudos observacionais e na opinião de especialistas, havendo atualmente algumas divergências entre as principais diretrizes.^316 - 318^

O principal determinante da EI é a doença estrutural cardíaca, especialmente as valvopatias.^315^ No Brasil, a principal causa de doença valvar crônica é a febre reumática, cuja prevalência e mortalidade têm aumentado anualmente, acometendo predominantemente adultos jovens.^319^ A lesão do endotélio cardíaco em decorrência de lesões estruturais promove exposição subendotelial, gerando ativação plaquetária, seguida de deposição de fibrina. Nesses locais de endotélio lesionado ou inflamado, pode ocorrer a adesão de microrganismos circulantes e sua proliferação, culminando na EI. No caso de microrganismos de alta virulência, como o *Staphylococcus aureus* , e uso de drogas intravenosas e dispositivos vasculares, a EI pode ocorrer em indivíduos com coração estruturalmente normal.^320^

Uma vez que bactérias são as principais etiologias da EI, diversos estudos avaliaram o risco de bacteremia relacionada a atividades rotineiras e a procedimentos invasivos. Nos países de baixa e média renda, como o Brasil, a principal etiologia da EI é *Streptococcus spp* ., especialmente do grupo *viridans* , encontrados na cavidade oral. Já nos países de alta renda, a principal etiologia é *Staphylococcus aureus* , relacionado majoritariamente à assistência à saúde.^315^ Descrevemos a seguir as recomendações de profilaxia para cada tipo de procedimento baseadas nas evidências científicas mais recentes, com enfoque nas particularidades da população brasileira.

##### 4.4.1. Procedimentos Odontológicos

Desde a década de 30, diversos estudos correlacionaram a extração dentária e a manipulação endodôntica e/ou periodontal à presença de bacteremia transitória.^321 - 324^ Seguindo esse preceito, modelos experimentais animais confirmaram a redução da bacteremia após manipulação dentária com o uso de antibioticoterapia profilática.^325 , 326^ Desde então, foi estabelecida a profilaxia antes de procedimentos odontológicos para os indivíduos com doença estrutural cardíaca.

O impacto positivo da profilaxia e o risco no emprego de antibióticos têm sido avaliados nos últimos anos. Estudos observacionais estimaram a prevalência de 2,7 a 13% de EI relacionada a procedimentos dentários.^327 - 329^ As atividades cotidianas, como mastigação, escovação dentária e utilização de fio dental, também se correlacionaram à bacteremia transitória.^325 , 330 - 333^ Além disso, o emprego de antibióticos tem o potencial risco de eventos adversos, como anafilaxia, e o uso frequente aumenta a possibilidade de indução de resistência bacteriana.^333 , 334^

Frente a essa lacuna de evidências científicas e ao potencial risco na prescrição de antibióticos *versus* a elevada morbidade e mortalidade da EI, houve uma readequação nas recomendações de profilaxia antes de procedimentos dentários. Atualmente, é recomendada a profilaxia para indivíduos com alto risco de EI, havendo algumas diferenças nas diretrizes.

Em 2008, o National Institute for Health and Clinical Excellence (NICE) no Reino Unido passou a não recomendar a profilaxia contra EI para indivíduos submetidos a procedimentos dentários.^318^ Um estudo observacional naqueles países mostrou aumento na incidência de EI após a restrição da profilaxia a partir de 2008; entretanto, não é possível estabelecer correlação direta com as recomendações do NICE.^335 , 336^ A partir de 2016, a recomendação foi atualizada para "a profilaxia não é recomendada rotineiramente", passando a admitir o uso de antibióticos de forma individualizada e detalhando os indivíduos com elevado risco de desenvolver EI.^337^ Em 2020, outro estudo observacional indicou aumento dos casos de EI na Inglaterra; entretanto, sem tendência de crescimento logo após ajuste das recomendações em 2008 e sem aumento no caso de EI causada por estreptococos orais.^338^

A American Heart Association (AHA) recomenda a profilaxia para indivíduos com alto risco de EI desde 2007^339^, e a European Society of Cardiology (ESC), para o mesmo grupo desde 2009.^317^ A população considerada de alto risco de desenvolver complicações relacionadas à EI está descrita na Tabela 14 .^317 , 339^ Um estudo mostrou aumento nos casos de EI estreptocócica nos Estados Unidos em relação às demais etiologias a partir da atualização das recomendações de profilaxia em 2007; entretanto, sem aumento nos casos totais de hospitalização ou cirurgia.^340^ Outros estudos observacionais americanos não mostraram aumento no número de casos totais de EI ou de EI por microrganismos relacionados à cavidade oral após a atualização nas recomendações de profilaxia.^335 , 341 , 342^

**Tabela 14:** Pacientes com indicação de profilaxia para endocardite infecciosa^316 - 318^

Sociedade Brasileira de Cardiologia
Indivíduos com maior risco de desenvolver endocardite infecciosa: Doença cardíaca valvar adquirida com estenose ou regurgitação Prótese cardíaca valvar Valvopatia adquirida corrigida com material protético Endocardite prévia Miocardiopatia hipertrófica Cardiopatia congênita cianogênica não corrigida Cardiopatia congênita cianogênica corrigida com material protético (primeiros 6 meses) Cardiopatia congênita cianogênica corrigida com lesão residual Valvopatia em paciente transplantado cardíaco
**Não é indicada profilaxia:** dispositivos eletrônicos implantáveis (marca-passo, cardiodesfibrilador), defeito septal corrigido, stents vasculares, filtro de cava, shunts no sistema nervoso central, fístulas, cateteres venosos
American Heart Association (AHA)
**Particularidades:** indicada profilaxia para indivíduos com maior risco de desfecho desfavorável se desenvolver endocardite infecciosa. Não contempla doença cardíaca valvar adquirida com estenose ou regurgitação.
European Society of Cardiology (ESC)
**Particularidades:** não contempla doença cardíaca valvar adquirida com estenose ou regurgitação, valvopatia em paciente transplantado cardíaco, cardiopatias congênitas e valvopatia em paciente transplantado cardíaco.
National Institute for Health and Clinical Excellence (NICE)
**Particularidades:** não contempla valvopatia adquirida corrigida com material protético e valvopatia em paciente transplantado cardíaco; quanto às cardiopatias congênitas, é recomendada profilaxia, exceto defeito do septo atrial, defeito septal ventricular e ducto arterioso patente completamente corrigidos

Tendo em vista o baixo acesso à assistência odontológica no Brasil, potencialmente determinando elevadas taxas de má higiene dental e, portanto, maior risco de bacteremia relacionada a procedimentos dentários invasivos,^343^ associado à raridade de eventos adversos graves com antibiótico e à inexistência de estudos de profilaxia em nosso meio, incluímos nas nossas orientações a profilaxia para pacientes com doença de valva nativa.

Dessa forma, recomenda-se a utilização de profilaxia para endocardite antes de procedimentos dentários que envolvam a manipulação de tecido gengival, região periodontal ou perfuração da mucosa oral ( Tabela 15 ) para todos os indivíduos com doença valvar anatomicamente significativa ( Tabela 14 ). O antibiótico deve ser administrado em dose única 30 a 60 minutos antes do procedimento ( Tabela 16 ). No caso de não utilização do antibiótico antes do procedimento, ele pode ser utilizado em até 2 horas após o procedimento.

**Tabela 15:** Procedimentos dentários e indicação de profilaxia para endocardite infecciosa

Profilaxia indicada	Pacientes que serão submetidos a procedimentos com manipulação do tecido gengival, região periodontal e/ou perfuração da mucosa oral
Profilaxia não indicada	Anestesia local em tecido não infectado Radiografia odontológica Colocação, ajustes ou remoção de aparelhos ortodônticos Queda natural de dente de leite Sangramento oriundo de trauma da mucosa oral ou lábios

**Tabela 16:** Esquemas de profilaxia a serem utilizados 1 hora antes de procedimentos dentários

Via de administração		Antibiótico	Dose adulto	Dose criança
Oral		Amoxicilina	2 g	50 mg/kg
	Alergia à penicilina	Azitromicina ou Claritromicina	500 mg	15 mg/kg
		Cefalexina *	2 g	50 mg/kg
		Doxiciclina	100 mg	2,2 mg/kg < 45 kg, 100 mg > 45 kg
		Clindamicina	600 mg	20 mg/kg
Parenteral (IV ou IM)		Ampicilina	2 g	50 mg/kg
	Alergia à penicilina	Cefazolina ou Ceftriaxona *	1 g	50 mg/kg
		Clindamicina	600 mg	20 mg/kg

* Evitar cefalosporinas nos casos de anafilaxia, angioedema ou urticária com penicilina devido ao risco de sensibilidade cruzada. IV: intravenoso; IM: intramuscular.

A EI é mais frequente como resultado de bacteremia por atividades diárias do que após procedimentos dentários. Não há dúvidas de que manter uma boa saúde bucal é a melhor estratégia de prevenção de endocardite. Nos indivíduos com doenças periodontais e endodônticas, a incidência e a magnitude da bacteremia nos cuidados cotidianos e durante procedimentos são maiores em relação a indivíduos com dentes saudáveis.^343^ Dessa forma, recomendamos incentivar cuidados dentários diários e avaliação bianual por dentista.

O Quadro 27 apresenta as recomendações para profilaxia antes de procedimentos dentários.

**Quadro 27:** Recomendação para profilaxia antes de procedimentos dentários

Recomendação	Classe de recomendação	Nível de evidência
Pacientes com condições de risco para endocardite infecciosa de acordo com a Sociedade Brasileira de Cardiologia ( Tabela 14 ) e submetidos a procedimentos com manipulação do tecido gengival, região periodontal e/ou perfuração da mucosa oral	I	B

##### 4.4.2. Procedimentos no Trato Respiratório

Pacientes submetidos à incisão ou à biópsia da mucosa do trato respiratório, como cirurgias otorrinolaringológicas, devem receber esquema antimicrobiano igual ao utilizado antes de tratamento odontológico com elevado risco de bacteremia. Não há recomendação de profilaxia para broncoscopia, laringoscopia e intubação orotraqueal. No caso de procedimento para tratamento de infecção, como drenagem de abscesso, deverá ser administrada profilaxia com antibiótico com ação antiestafilocócica.^317^

##### 4.4.3. Procedimentos no Trato Geniturinário e Gastrointestinal

Apesar das poucas evidências, recomendamos a profilaxia antes de procedimentos geniturinários ou gastrointestinais com incisão ou biópsia de mucosa para os pacientes com alto risco de EI. Entre estes, incluem-se o parto vaginal e a cesárea, situações potencialmente relacionadas à bacteremia, embora igualmente não haja estudos específicos quanto à eficácia dessa conduta.^344^

Nos exames de endoscopia digestiva alta e colonoscopia, recomendamos a prescrição de profilaxia sempre que houver elevada possibilidade de manipulação da mucosa (por exemplo, biópsias, ressecções). Na ocorrência de manipulação inesperada de mucosa sem a prévia administração de profilaxia, os antibióticos poderão ser administrados até 2 horas após o procedimento. Nos demais exames endoscópicos, como ecocardiograma transesofágico e colangiopancreatografia (CPRE), não está indicada profilaxia.

O esquema antibiótico recomendado para os procedimentos geniturinários e gastrointestinais está descrito na Tabela 17 .

**Tabela 17:** Esquemas de profilaxia a serem utilizados 30 minutos antes de procedimentos geniturinários e gastrointestinais

Antibiótico		Dose adulto	Dose criança
	Ampicilina venosa * + Gentamicina venosa	2 g 1,5 mg/kg	50 mg/kg 1,5 mg/kg
Alergia à penicilina:			
	Vancomicina venosa + Gentamicina venosa	1 g 1,5 mg/kg	20 mg/kg 1,5 mg/kg

* Fazer reforço com ampicilina venosa 1 g 6 horas após o procedimento; como alternativa administrar amoxicilina 1 g.

O Quadro 28 apresenta as recomendações para profilaxia antes de procedimentos geniturinários e gastrointestinais.

**Quadro 28:** Recomendação para profilaxia antes de procedimentos geniturinários e gastrointestinais

Recomendação	Classe de recomendação	Nível de evidência
Pacientes com condições de risco para endocardite infecciosa de acordo com a Sociedade Brasileira de Cardiologia ( Tabela 14 )	IIa	C

##### 4.4.4. Procedimentos Dermatológicos e Musculoesqueléticos

No caso de procedimento para tratamento de infecção, como drenagem de abscesso, deverá ser administrada profilaxia com antibiótico com ação antiestafilocócica e antiestreptocócica.^317^

##### 4.4.5. *Piercing* e Tatuagem

Há relatos de casos de EI relacionada a *piercing* e tatuagem, especialmente *piercing* colocado na língua.^345 , 346^ Dessa forma, os pacientes devem ser alertados sobre esse risco e desencorajados de realizar esses procedimentos.^317^

## 5. Biomarcadores no Perioperatório

### 5.1. Peptídeos Natriuréticos

Os peptídeos natriuréticos são liberados na circulação sanguínea pelo miocárdio em resposta a múltiplos estímulos fisiológicos, como estresse miocárdico, isquemia e outros. Múltiplos estudos demonstraram que níveis pré-operatórios elevados de BNP são potentes preditores de complicações CV perioperatórias.^347 , 348^

Em 2012, Biccard et al. Conduziram um estudo prospectivo observacional envolvendo 788 pacientes submetidos a cirurgia não cardíaca visando avaliar a utilidade clínica da dosagem pré-operatória de BNP em comparação com outros biomarcadores pré-operatórios como as troponinas. Tanto a elevação de troponina quanto de BNP pré-operatórios foram preditores independentes de eventos cardíacos.^349^

Em uma metanálise realizada com 15 estudos observacionais prospectivos e 4.856 pacientes, os autores encontraram que a elevação pré-operatória de níveis de BNP ou NT-pró-BNP foi associada a um risco elevado (20 vezes maior) de eventos CV maiores, mortalidade cardíaca e mortalidade por todas as causas (10 vezes) no período perioperatório (< 43 dias da cirurgia).^350^ No entanto, dados desses estudos não permitiram definir um ponto de corte para o BNP, além de não definir se acrescentam informação prognóstica sobre os índices de risco prognóstico já existentes.^351^

Um estudo observacional multicêntrico prospectivo analisou 979 pacientes no período pré-operatório de cirurgia não cardíaca com o objetivo de avaliar o valor incremental da troponina de alta sensibilidade como preditor de risco em comparação com o escore RCRI. Nesse estudo, os autores também avaliaram o papel dos peptídeos natriuréticos como preditores de risco em cirurgia não cardíaca. Observaram que tanto os níveis de troponina de alta sensibilidade quanto de NT-pró-BNP estavam mais elevados entre os indivíduos que evoluíram a óbito quando comparados aos que sobreviveram. Os autores ainda sugerem que valores de NT-pró-BNP maiores do que 300 pg/mL conferem maior risco de mortalidade (4,8% *versus* 1,4%, p = 0,002).^352^

Em 2014, uma metanálise envolvendo 18 estudos prospectivos observacionais avaliou dados individuais de 2.179 pacientes submetidos a cirurgia não cardíaca e revelou que níveis de BNP e NT-pró-BNP pré e pós-operatório melhoram a predição de risco de morte ou infarto não fatal em 30 dias após o procedimento. Níveis pré-operatórios de BNP > 92 pg/mL ou NT-pró-BNP > 300 pg/mL conferem aumento do risco de morte ou infarto não fatal em 3,4 vezes ( *odds ratio* 3,4 com IC95% de 2,6 a 4,5). Níveis pós-operatórios BNP > 400 pg/mL e de NT-pró-BNP > 900 conferem aumento de risco em 2,7 e 1,8 vezes respectivamente. Os níveis dos peptídeos natriuréticos, tanto pré quanto pós-operatórios, foram preditores independentes de morte e infarto não fatal em 30 dias. A metanálise também indicou que níveis de BNP < 30 pg/mL têm probabilidade negativa de evento perioperatório.

A dosagem adicional de peptídeo natriurético pós-operatória pode melhorar a estratificação de risco para eventos CV em 30 dias e 180 dias após cirurgia não cardíaca em relação à dosagem pré-operatória.^353^ Mais recentemente, uma subanálise do estudo multicêntrico VISION ( *Vascular Events in Noncardiac Surgery Patients Cohort Evaluation* ) envolvendo 10.402 pacientes com idade maior do que 45 anos submetidos a cirurgias não cardíacas (vasculares e não vasculares) confirmou resultados de estudos anteriores de que peptídeos natriuréticos são bons preditores de morte vascular ou injúria miocárdica no pós-operatório de cirurgia não cardíaca.^354^ Valores de NT-pró-BNP entre 100 e 200 pg/mL aumentaram o risco de evento primário em 2,3 vezes, valores entre 200 e 1.500 pg/mL, em 3,6 vezes, e maiores do que 1.500 pg/mL, em 5,5 vezes. Os autores mostram ainda que NT-pró-BNP pré-operatório melhora a predição de risco cardíaco quando adicionado ao escore de risco RCRI.^355^

Diante das evidências aqui expostas, propõe-se discreta alteração nas recomendações conforme descrito a seguir.

O Quadro 29 apresenta as recomendações da dosagem pré-operatória de BNP/NT-pró-BNP para predizer risco de eventos CV perioperatórios.

**Quadro 29:** Recomendações da dosagem pré-operatória de BNP/NT-pró-BNP para predizer risco de eventos cardiovasculares perioperatório

Recomendação	Classe de recomendação	Nível de evidência
Pacientes com idade maior que 65 anos ou pacientes com idade entre 45 e 64 anos com doença cardiovascular estabelecida ou fator de risco * para DCV submetidos a operações não cardíacas	I	B

* Diabetes, hipertensão, doença arterial coronariana, obesidade, fibrilação arterial (fatores de risco para desenvolver insuficiência cardíaca com fração de ejeção preservada). BNP: Peptídeo natriurético do tipo B; NT-pró-BNP: peptídeo natriurético do tipo B N-terminal.

### 5.2. Troponinas Cardíacas e Monitorização de Complicações Cardiovasculares

Apesar dos diferentes algoritmos e ferramentas disponíveis para a correta estratificação de risco de pacientes em programação de cirurgia não cardíaca, a acurácia na predição de eventos desfavoráveis ainda fica aquém do ideal.^356^ Entre as diversas ferramentas que podem auxiliar na predição de eventos CV e mortalidade pós-operatória, o uso de biomarcadores vem ganhando maior espaço com as evidencias cientificas adquiridas nas últimas décadas, em especial através do uso de peptídeos natriuréticos (BNP/NT-pró-BNP) e troponina T e I de alta sensibilidade (hs-cTnT/I).

No contexto pré-operatório de cirurgia não cardíaca, diversos estudos consolidaram o papel da troponina de alta sensibilidade na otimização da estratificação de risco. Em um estudo com 979 pacientes submetidos a cirurgias não cardíacas, uma dosagem pré-operatória de hs-cTnT acima do percentil 99 demostrou risco aumentado para o desfecho combinado de mortalidade, IAM, parada cardíaca recuperada e insuficiência cardíaca aguda (RR 2,6 IC95% 1,3-5,3). A mesma coorte demostrou superioridade da hs-cTnT em relação ao algoritmo do RCRI na predição de mortalidade intra-hospitalar (AUC 0,809 *vs.* 0,658, p = 0,006).^352^ Uma metanálise com sete estudos e mais de 4.000 pacientes revelou aumento de eventos CV combinados (RR 2,9, IC95% 1,9 a 4,4), mortalidade a curto prazo (RR curto prazo 5,4, IC95% 3,21-9,06) e mortalidade a longo prazo (RR 2,9, IC95% 1,8-4,6) nos pacientes com troponina T de alta sensibilidade elevada antes de cirurgia não cardíaca.^357^

Os *kits* de troponina I de alta sensibilidade também foram avaliados no contexto perioperatório. Nos estudos BASEL-PMI e Tropovasc, 1.022 pacientes foram avaliados com uso de troponina T e I de alta sensibilidade. Ambas as troponinas apresentaram boa acurácia na predição de eventos CV combinados em 30 dias após cirurgias não cardíacas e não vasculares (AUC hs-cTnI 0,77, IC95% 0,71–0,83 e AUC hs-cTnT 0,79, IC95% 0,73–0,85). Nos pacientes submetidos a cirurgias vasculares arteriais, a troponina I de alta sensibilidade apresentou melhor desempenho quando comparada a troponina T (AUC hs-cTnI 0,67, IC95% 0,59–0,75, e AUC hs-cTnT 0,59, IC95% 0,51–0,67).^358^

Por fim, em pacientes internados com programação cirúrgica, diversas comorbidades e patologias agudas e crônicas podem influenciar nos níveis de troponina (por exemplo, anemia, sepse, insuficiência renal, IC).^359 , 360^ Diferentes coortes observaram que a troponina de alta sensibilidade (em especial hs-cTnT) pode estar elevada acima do percentil 99 do valor superior de referência em até 50% dos pacientes que irão ser submetidos a cirurgias não cardíacas.^352 , 358 , 361 - 365^

Portanto, embora a presença de elevação de troponina pré-operatória seja um fator de risco para a ocorrência de eventos pós-operatórios, ainda não há evidências ou recomendações específicas para investigações adicionais ou medidas específicas para redução do risco nessa população. Esses valores elevados devem ser considerados como uma injúria miocárdica crônica (definida pela 4ª definição universal de infarto como pelo menos um valor de troponina acima do percentil 99 do valor superior de referência sem alteração dinâmica).^360^ Valores desproporcionalmente elevados em relação às comorbidades conhecidas do paciente devem ser investigados antes de operações completamente eletivas. Nos demais casos, deve ser considerado o valor basal do paciente e ser utilizado para a correta interpretação das dosagens obtidas na monitorização subsequente de eventos, facilitando o diagnóstico correto da injúria/IAM perioperatório (PMI) e diferenciando elevações agudas de crônicas do biomarcador.^361^

Como o PMI é na maioria das vezes assintomático, a monitorização com a dosagem de troponina diária no primeiro e segundo dias de pós-operatório deve ser feita para diagnosticar o PMI (vide Item 6.1). Ainda não há um consenso definitivo sobre em quais pacientes exatamente a monitorização com troponina tem a melhor custo-efetividade. Ao realizar monitorização indiscriminadamente, não se perde nenhum PMI, mas realiza-se muitos exames normais. Por outro lado, ao monitorar somente pacientes de risco muito alto, a incidência de PMI será maior, mas muitos pacientes não são diagnosticados.

No estudo VISION, os autores realizaram monitorização com troponinas em todos os pacientes com idade acima de 45 anos submetidos a operações em anestesia regional ou geral, internados por pelo menos 1 dia.^365^ No estudo BASEL-PMI, os autores monitoraram pacientes com idade superior a 65 anos ou com idade superior a 45 anos e com doença aterosclerótica conhecida (coronária, cerebrovascular ou periférica).^361^ Em dados mais recentes, o percentual relativo de indivíduos que registraram injúria miocárdica após cirurgias não cardíacas (MINS) foi maior entre aqueles classificados como de maior risco pelo RCRI (7,9%, 14,2%, 25,3% e 38% nas classes I, II, II e IV, respectivamente); porém, em números absolutos, 30% de todas as injúrias foram em indivíduos na classe I, ou seja, escore do RCRI de 0.^366^ Por outro lado, não há evidência para realização de troponina de rotina em pacientes sem fatores de risco ou RCRI classe I que são submetidos a procedimentos de menor complexidade.^367^ Em contrapartida, já foram demonstrados custo-efetividade e posicionamento de outros autores em favor da monitorização de troponina perioperatória para os pacientes acima de 65 anos ou com histórico de doença aterosclerótica.^361 , 368 , 369^

Na diretriz da ESC recentemente publicada, a monitorização é recomendada pera pacientes com fatores de risco CV (idade ≥ 65 anos, dislipidemia, hipertensão arterial, diabetes, tabagismo, história familiar), doença CV conhecida ou sintomas de doença CV.^3^ Considerando as particularidades do nosso país e as evidências disponíveis, esta Diretriz recomenda a realização da monitorização sistemática com realização de ECG e dosagem de troponina no pré-operatório e uma vez ao dia durante 48 horas para pacientes com estimativa de risco intermediário ou alto de acordo com os algoritmos de avaliação pré-operatória. Outra informação de extrema relevância é que cerca de 80 a 90% das alterações de troponina, isoladas ou não, foram registradas até o segundo dia de pós-operatório,^361 , 365^ sendo razoável realizar a monitorização por 48 horas após a operação.

Cabe ressaltar que, após a disponibilidade das troponinas de alta sensibilidade e das novas evidências, a monitorização sistemática com ECGs ou monitorização automática do segmento ST caiu em desuso por apresentar grande variação de sensibilidade (entre 55 e 100%) e especificidade (entre 37 e 85%) para a detecção de isquemia perioperatória (intra e pós-operatória), porque sua efetividade depende da técnica empregada e também das características basais da população estudada (probabilidade pré-teste de DAC).^370 - 372^

O Quadro 30 apresenta as recomendações para a monitorização no perioperatório.

**Quadro 30:** Recomendações para a monitorização no perioperatório

Recomendação	Classe de recomendação	Nível de evidência
Dosagem de troponina no pré-operatório, primeiro e segundo dia do pós-operatório, em pacientes de risco intermediário ou alto de complicações segundo algoritmos, submetidos a cirurgias não cardíacas de risco intermediário ou alto	I	B
Realização de eletrocardiograma no pré-operatório, primeiro e segundo dia do pós-operatório, em pacientes de risco intermediário ou alto de complicações segundo algoritmos, submetidos a cirurgias não cardíacas de risco intermediário ou alto	I	C
Pós-operatório em unidade de tratamento intensivo (UTI) por 48 horas em pacientes de risco alto de complicações segundo algoritmos, submetidos a cirurgias não cardíacas de risco intermediário ou alto	I	C
Pós-operatório em UTI por 48 horas em pacientes de risco intermediário de complicações segundo algoritmos, submetidos a cirurgias não cardíacas de risco intermediário ou alto	IIa	C
Dosagem de troponinas em pacientes de baixo risco ou pacientes submetidos a operações de baixo risco	III	B

## 6. Diagnóstico e Tratamento das Complicações Cardiovasculares no Perioperatório

### 6.1. Injúria/infarto Agudo do Miocárdio Perioperatório (PMI)

O IAM é a complicação cardíaca mais temida no período perioperatório, descrito em 0,3 a 3% dos pacientes de baixo risco e sem história de DAC e chegando a 33% em pacientes de alto risco com história de DAC.^373^ Em estudos observacionais mais recentes, a incidência variou entre 0,4 a 0,9%, porém admitindo-se subdiagnóstico na ausência de monitorização adequada.^374 , 375^ Embora os índices de mortalidade mostrem um redução nas últimas décadas, readmissão hospitalar ou morte em 30 dias ainda ocorrem em um a cada três pacientes,^375 - 377^ o que está provavelmente relacionado à existência de comorbidades, à dificuldade diagnóstica e à limitação para o uso do arsenal terapêutico antitrombótico e antiplaquetário em pacientes com IAM perioperatório.

A maioria dos pacientes com IAM perioperatório não apresenta sintomas, devido à ocorrência dos eventos no intraoperatório, assim como ao uso de analgesia potente no pós-operatório.^356 , 361 , 365 , 378 , 379^ Além disso, quando presente, a dor torácica é frequentemente atribuída a outras etiologias mais óbvias, como dores incisionais ou relacionadas à posição do paciente. Outras manifestações, como dispneia e náuseas, têm explicações alternativas nesse período (atelectasias e efeito de medicamentos), fazendo com que a hipótese de IAM perioperatório seja frequentemente subvalorizada pela equipe médica. Além disso, somente 25% dos pacientes apresentam alterações de isquemia no ECG.^361 , 380^ Alterações isquêmicas devem ser diferenciadas de outras causas de alterações do ECG, como distúrbios eletrolíticos, hipotermia, efeitos de drogas ou posicionamento incorreto das derivações. Por isso, a monitorização com a dosagem de troponinas é a peça fundamental no diagnóstico.

**Figura 7:**
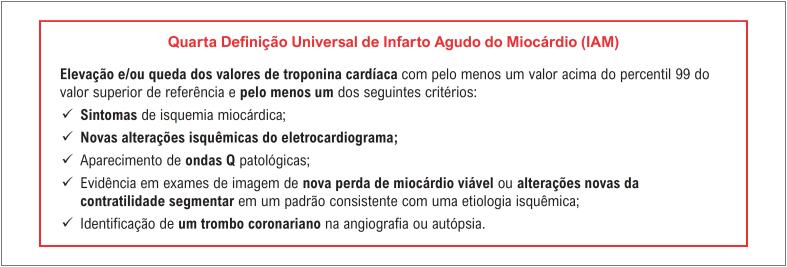
Quarta definição universal de infarto agudo do miocárdio.

Nos últimos anos, foi demonstrado que as elevações de troponina no pós-operatório estão associadas à alta mortalidade em 30 dias e 1 ano após a operação, independentemente da presença de outros critérios da definição universal de IAM ( Figura 7 ). Levando em consideração todas as particularidades do IAM perioperatório, optamos por utilizar o termo "injúria/infarto agudo do miocárdio perioperatório" (PMI) para os eventos que ocorrem nos primeiros 2 dias após a operação, durante a fase de monitorização utilizando as troponinas.^361^ Esta diretriz optou por utilizar a abreviatura PMI para representar o termo injúria/infarto agudo do miocárdio perioperatória, tendo em vista o uso consagrado e mais comum da abreviatura na língua inglesa.

**Figura 8:**
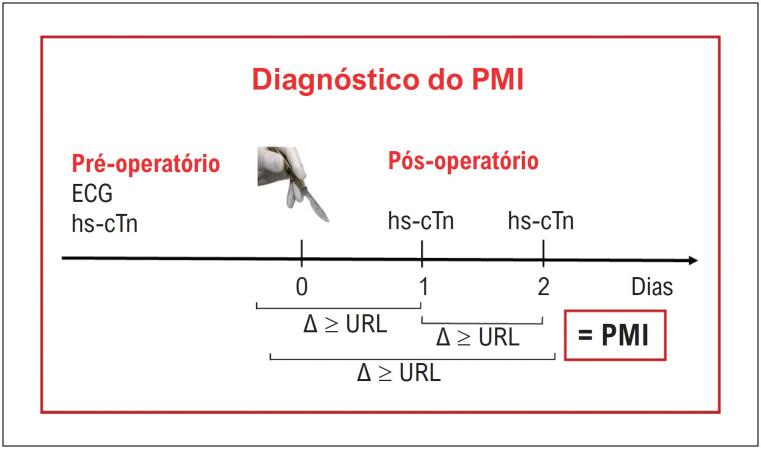
Diagnóstico do PMI. URL: valor superior de referência do percentil 99 do kit; URL hs-cTnT (Roche) 14 ng/L, URL hs-cTnI (Abbott) 26 ng/L, URL hs-cTnI (Siemens) 40 ng/L. ECG: eletrocardiograma; hs-cTn: troponina cardíaca de alta sensibilidade; PMI: injúri/infarto do miocárdico perioperatório.

O PMI é definido como a ocorrência de uma elevação de troponina aguda após a operação, definida como um delta absoluto igual ou superior ao valor do percentil 99 do limite superior de referência do *kit* de troponina utilizado entre o valor pré-operatório e o valor do primeiro ou do segundo dia pós-operatório. Na ausência de um valor pré-operatório, o delta pode ser realizado entre os dois valores de troponina pós-operatórios ( Figura 8 ).^361 , 378^ No estudo BASEL-PMI, no qual a monitorização utilizando a troponina foi realizada em pacientes com idade acima de 65 anos ou pacientes com idade acima de 45 anos e doença aterosclerótica (coronária, cerebral ou periférica) submetidos a operações não cardíacas, a incidência de PMI utilizando a troponina T de alta sensibilidade (delta ≥ 14 ng/L) foi de 16%. Confirmando os achados de estudos anteriores, apenas 18% dos pacientes apresentaram sintomas. Pacientes com PMI apresentaram uma mortalidade significativamente maior do que pacientes sem PMI em 30 dias (9% x 1%, p < 0,001, razão de risco ajustada [aHR] 2,7; IC95% 1,5–4,8) e 1 ano (23% x 9%, p < 0,001, aHR 1,6; IC95% 1,2–2,2).^361^ Esses achados foram posteriormente validados para a troponina I.^378^

A troponina I tem diversos fabricantes, sendo que cada fabricante tem um valor do percentil 99 do limite superior de referência. A definição de PMI foi validada para os *kits* de troponina I da Abbott (delta ≥ 26 ng/L) e Siemens (delta ≥ 40 ng/L), porém podemos extrapolar a definição de PMI para os demais *kits* .^378^ Embora não tenha havido diferença na mortalidade entre pacientes com PMI que preenchiam um dos critérios da definição universal de IAM ou não, os pacientes com PMI que preenchiam algum dos critérios adicionais da definição de IAM ( Figura 7 ) apresentaram uma maior incidência de eventos CV maiores (MACE, incluindo IAM, IC aguda, arritmias e morte CV) do que pacientes com PMI somente com a elevação de troponina isolada.^378^

Outro conceito específico do perioperatório é a definição de MINS (injúria miocárdica após cirurgias não-cardíacas). MINS é definido como um valor de troponina T ≥ 65 ng/L no pós-operatório ou um valor de troponina T entre 20 e 65 ng/L e um delta absoluto ≥ 5 ng/L entre os valores pré e pós-operatórios. Além disso, MINS se refere a causas isquêmicas de elevações de troponinas, isto é, outras causas como sepse, tromboembolismo pulmonar, taquiarritmias e IC foram excluídas.^365^ O estudo VISION incluiu pacientes com idade acima de 45 anos que foram submetidos a cirurgias não cardíacas e demonstrou uma incidência de MINS de 18%, sendo que 93% dos pacientes com MINS não apresentaram sintomas. A ocorrência de MINS foi relacionada com um aumento importante da mortalidade (aHR 3,20; IC95% 2,4-4,3). Além disso, os autores demonstraram que quanto maior o valor absoluto da troponina pós-operatória, assim como quanto maior o delta agudo, maior a mortalidade.^365^ Cabe ressaltar que parte da definição de MINS leva em conta apenas o valor absoluto da troponina no pós-operatório, não diferenciando a injúria miocárdica aguda da crônica.

**Figura 9:**
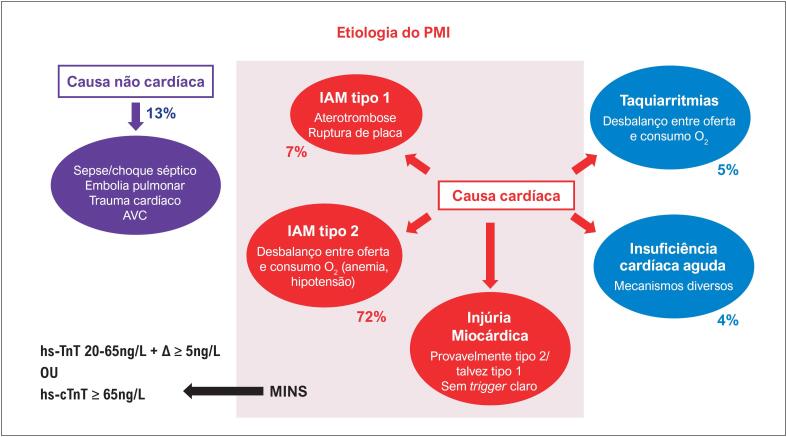
Etiologia do PMI e de MINS. PMI: injúria/infarto agudo perioperatório, IAM: infarto agudo do miocárdio, AVC: acidente vascular cerebral; MINS: injúria miocárdica após cirurgia não cardíaca; hs-cTnT: troponina T de alta sensibilidade. O trecho da figura com fundo cor de rosa representa o que é MINS.

Tradicionalmente, a etiologia do IAM perioperatório foi considerada como uma mistura entre IAM do tipo 1 (ruptura de placa devido ao aumento da agregabilidade plaquetária, resposta inflamatória, redução da fibrinólise, liberação de catecolaminas) e IAM do tipo 2 (desbalanço entre a oferta/consumo de oxigênio devido a anemia, taquicardia, hipoxemia e hipotensão).^356 , 373^ Estudos anatomopatológicos e *in vivo* demonstraram que cerca de 50% dos IAMs perioperatórios eram do tipo 1.^379 , 381 - 383^ Entretanto, após o desenvolvimento dos exames de troponina de alta sensibilidade, podemos detectar elevações de troponina em diversos outros contextos que não IAM, como sepse, IC, insuficiência renal e até mesmo após exercício extenuante.^359 , 384^ Por isso, a etiologia do PMI é muito mais abrangente e inclui o conceito de MINS, como demonstrado na Figura 9 ^385^, e o prognóstico do PMI também depende diretamente da etiologia, sendo que pacientes com PMI por causas não cardíacas (sepse), IC e taquiarritmias apresentam mortalidade mais elevada.^386^

Em pacientes com PMI, a realização do ECG é mandatória, e a realização de um ecocardiograma, se possível, deve ser considerada para a avaliação da função sistólica biventricular, presença de doença valvar e alterações na contratilidade segmentar.

**Figura 10:**
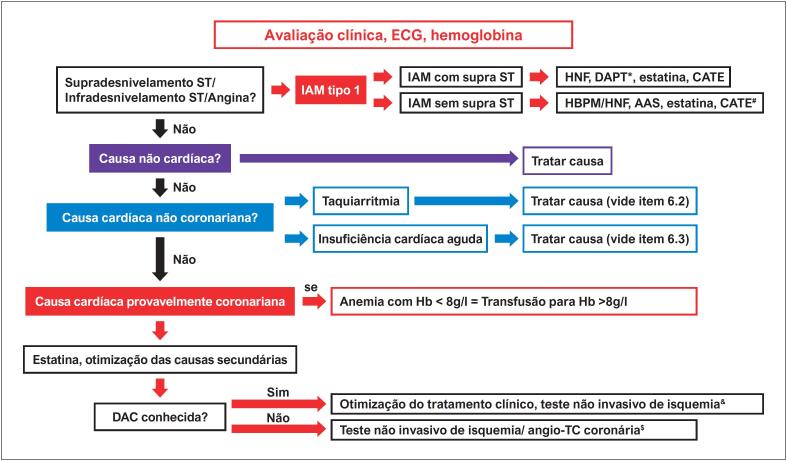
Fluxograma de conduta em pacientes com PMI. *Clopidogrel ou ticagrelor a depender do risco de sangramento; ^#^timing a depender do risco de sangramento após discussão interdisciplinar; ^&^se não realizada no pré-operatório, ambulatorial ou antes da alta conforme o caso; ^$^ambulatorial ou antes da alta conforme o caso. AAS: ácido acetilsalicílico; CATE: cineangiocoronariografia; DAP: dupla antiagregação plaquetária; ECG: eletrocardiograma; Hb: hemoglobina; HBPM: heparina de baixo peso molecular; HNF: heparina não fracionada; IAM: infarto agudo do miocárdio; PMI: injúria/infarto agudo do miocárdio perioperatório.

O tratamento do PMI depende fundamentalmente da etiologia, e a conduta diante de um paciente com PMI está demonstrada na Figura 10 . Cabe ressaltar que, na presença de um IAM tipo 1, a escolha do antiagregante a ser adicionado à aspirina depende do risco de sangramento, que deve ser discutido em conjunto com o cirurgião. Em pacientes de alto risco de sangramento, deve-se dar preferência ao clopidogrel. Nos casos de IAM sem supra de ST, a administração do segundo antiagregante deve ser feita durante ou após a cineangiocoronariografia. Além disso, a otimização das causas secundárias de isquemia (taquicardia, hipotensão, hipertensão, anemia, dor) deve sempre ser realizada.^373^ Embora as evidências para o tratamento do PMI sejam escassas, estudos retrospectivos sugerem que a otimização do tratamento clínico dos fatores de risco CV (dislipidemia, hipertensão arterial, diabetes, cessação do tabagismo) está associada a uma melhora do prognóstico e, portanto, deve ser realizada em todos os pacientes.^387^

Em um estudo recente, foi demonstrado que os fatores de risco para a ocorrência do desfecho combinado de MACE em pacientes com PMI de causa cardíaca provavelmente coronariana são presença de sintomas (angina ou dispneia), delta de troponina entre duas e quatro vezes o valor superior de referência do percentil 99, cirurgias de urgência e emergência e cirurgias de alto risco. Surpreendentemente, a presença de sangramento perioperatório foi um fator protetor, provavelmente porque, após a correção do *trigger* para a isquemia, não houve mais eventos.^386^ Quanto à realização de provas de isquemia em pacientes com PMI de causa provavelmente coronariana, ainda não há evidências para determinar o momento ideal do exame. Entretanto, considerando que o risco de eventos cardíacos e mortalidade após PMI é maior nos primeiros 120 dias após a cirurgia, é razoável realizar a prova funcional antes desse período.

Em relação ao tratamento de MINS, o estudo randomizado MANAGE avaliou o uso de dabigatrana 110 mg duas vezes ao dia em pacientes com MINS e demonstrou uma redução nos eventos combinados de mortalidade vascular, IAM não fatal, AVC isquêmico, trombose arterial periférica, amputação e trombose venosa.^388^

Embora menos frequente, o IAM perioperatório pode ocorrer após o segundo dia de pós-operatório, após o período de monitorização com troponinas. Nesses casos, o diagnóstico deve ser feito utilizando os critérios da definição universal de IAM ( Figura 7 ).^360^ O tratamento é o mesmo do IAM fora do contexto perioperatório, respeitando as particularidades já descritas acima (considerar risco de sangramento e discussão multidisciplinar com o cirurgião).^389^

O Quadro 31 apresenta as recomendações para o diagnóstico e tratamento do PMI e IAM perioperatório.

**Quadro 31:** Recomendações para o diagnóstico e tratamento do PMI

Recomendação	Classe de recomendação	Nível de evidência
O diagnóstico de PMI deve ser feito na presença de um delta absoluto ≥ ao percentil 99 do valor superior de referência do kit de troponina utilizado entre os valores de troponina pré-operatório e do primeiro ou segundo pós-operatório. Na ausência do valor basal, o delta pode ser considerado entre dois valores pós-operatórios	I	B
Realização de avaliação clínica, eletrocardiograma e hemoglobina em pacientes com PMI	I	B
Determinação da etiologia do PMI utilizando o fluxograma de conduta	I	B
Tratamento da causa do PMI segundo as diretrizes específicas	I	C
O diagnóstico do IAM após o segundo dia pós-operatório deve ser feito baseado na definição universal do IAM, e seu tratamento deve ser baseado nas diretrizes atuais	I	C
Em pacientes com IAM perioperatório ou PMI devido a causas isquêmicas, a otimização das causas secundárias de isquemia (anemia, taquicardia, hipotensão, hipertensão) deve ser realizada, assim como a avaliação de risco de sangramento e discussão multidisciplinar com o cirurgião	I	C

### 6.2. Fibrilação/ *Flutter* Atrial Agudos

O diagnóstico de FA/ *flutter* atrial requer a documentação do ritmo preferencialmente com um ECG de 12 derivações convencional para evitar artefatos e erros diagnósticos. A documentação a partir de derivação única (incluindo monitores hospitalares) requer duração da arritmia de pelo menos 30 segundos.^390^ Em cirurgias não cardíacas, uma metanálise mostrou a incidência de 10%^391^ de FA pós-operatória (FAPO), com maior incidência em cirurgias torácicas.^392^ Apesar de muitas vezes autolimitada e assintomática, está associada com maior risco recorrência tardia e de fenômenos cardioembólicos.^393^

A definição de FAPO é a ocorrência presumivelmente nova durante ou após as primeiras horas após a cirurgia, com pico de incidência entre o segundo e o quarto dias.^394^ A patogênese da FAPO resulta da interação entre inflamação, gatilhos preexistentes, doença estrutural e insultos perioperatórios.^395^ Sua ocorrência após cirurgia não cardíaca está associada ao aumento de três vezes no risco de AVC, cinco vezes no risco de IAM e três vezes em mortalidade.^396^ As medidas preventivas ao desenvolvimento de FA são o adequado controle hidroeletrolítico no pré e pós-operatório (normovolemia, monitorização e reposição de magnésio e potássio) e a manutenção das medicações de uso prévio, quando adequadas ao contexto hemodinâmico.^397^

Algumas medicações foram estudadas com o objetivo de reduzir a incidência de FA e suas consequências deletérias. A terapia antiarrítmica preventiva, com amiodarona ou magnésio venoso, pode ser discutida individualmente em pacientes em programação de cirurgias torácicas. O uso do magnésio oral (total de 3,2 g em três dias e 1,6 g no dia da cirurgia) em pacientes submetidos a revascularização do miocárdio (estudo POMAF-CS, controlado randomizado e unicêntrico) diminuiu a incidência de FAPO em comparação com placebo.^398^ Tisdale et al., em estudo retrospectivo, demonstrou que, em pacientes submetidos a esofagectomias, o uso da amiodarona endovenosa na indução anestésica (43,75 mg/hora durante 96 horas) reduziu a taxa de FA no perioperatório, porém sem impacto no tempo de internação. Ademais, a amiodarona nesse contexto resultou em hipotensão, bradicardia e prolongamento intervalo QT corrigido, sendo questionado seu uso rotineiro.^399^ Riber et al., em estudo randomizado, duplo-cego, placebo controlado, em pneumectomias, demonstraram que a administração de 300 mg de amiodarona venosa no pós-operatório, seguido de 1.200 mg oral por dia por 5 dias em pacientes estáveis hemodinamicamente reduziu a taxa de FA no perioperatório (9 vs. 32% no grupo-controle).^392^ Khalil et al., por sua vez, compararam três grupos no período pós-operatório imediato, o primeiro com a intervenção amiodarona (ataque de 5 mg/kg, seguido de 15 mg/kg por dia, por 48 horas); o segundo recebeu magnésio venoso (ataque de 80 mg/kg, seguido de 8 mg/kg/h, por 48 horas); por fim, o grupo-controle, proveniente de análise retrospectiva em pacientes submetidos à ressecção pulmonar, revelou uma taxa de FA no perioperatório, respectivamente, de 10, 12,5 e 20,5%.^400^ Outras medicações não antiarrítmicas também foram avaliadas. As estatinas mostraram papel potencial na prevenção de FAPO em cirurgia cardíaca, particularmente de revascularização miocárdica.^401 , 402^ Posteriormente, uma metanálise, apenas em pós-operatório de cirurgia não cardíaca, não observou o benefício na prevenção de FAPO.^272^ A colchicina permanece em investigação (COP-AF [ *Colchicine For The Prevention Of Perioperative Atrial Fibrillation In Patients Undergoing Thoracic Surgery* ]: NCT03310125).^403^

No manejo da FAPO, considerando a presença de fatores que dificultam o controle de ritmo, a primeira abordagem pode incluir correção de volemia, distúrbios eletrolíticos, redução de estímulos adrenérgicos (dor, inflamação pós-operatória) e controle de frequência cardíaca (< 100 a 110 bpm).^404^ Em paciente com fração de ejeção preservada, a preferência é por bloqueadores dos canais de cálcio e betabloqueadores, enquanto, em pacientes com fração de ejeção reduzida, digoxina e betabloqueadores específicos. A digoxina pode ter efeito atenuado pelo estado hiperadrenérgico cirúrgico. A introdução das medicações deve ser preferencialmente de titulação lenta, a fim de evitar hipotensão, que é sabidamente deletéria no pós-operatório. O controle do ritmo, ou seja, a reversão da FA, é a escolha preferencial em pacientes com alta resposta ventricular e difícil controle cronotrópico e pela cardioversão elétrica ou química, observando o tempo de início da arritmia e eventual necessidade de realizar ecocardiograma transesofágico, conforme diretriz brasileira de FA.^405^

**Figura 11:**
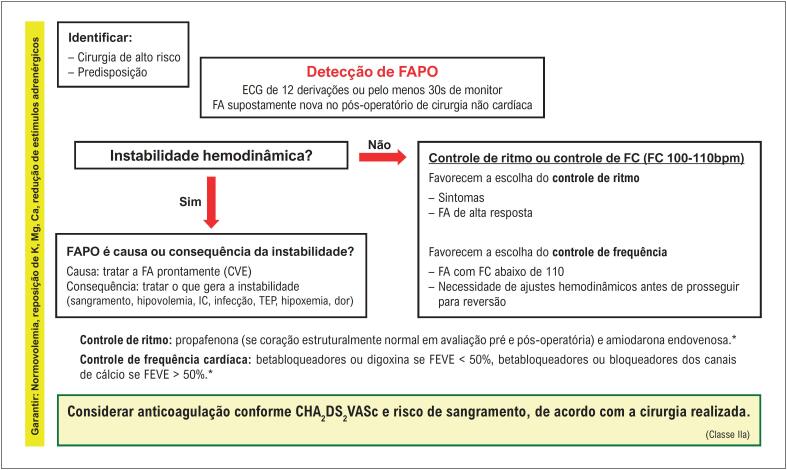
Fluxograma para o tratamento de pacientes com fibrilação atrial no pós-operatório. *Dosagens conforme diretriz de FA e julgamento clínico. FA: fibrilação atrial; FAPO: fibrilação atrial pós-operatória; FC: frequência cardíaca; CVE: cardioversão elétrica; IC: insuficiência cardíaca; TEP: tromboembolismo pulmonar; FEVE: fração de ejeção do ventrículo esquerdo.

Independentemente da estratégia de controle de ritmo ou de frequência cardíaca, o paciente deve ser avaliado para início de anticoagulação. Em longo prazo, a anticoagulação da FAPO era discutida individualmente; entretanto, em metanálise com cirurgias cardíacas e não cardíacas, foi observado risco 37% maior de AVC, e o risco de AVC foi maior entre os pacientes submetidos à cirurgia não cardíaca. Dessa maneira, a anticoagulação em longo prazo em pacientes com FAPO e com fatores de risco (CHA_2_DS_2_VASc) é recomendada para prevenir os eventos tromboembólicos, considerando o benefício clínico líquido, o risco de sangramento conforme a cirurgia realizada e a preferência do paciente ( Figura 11 ).

O Quadro 32 apresenta a recomendação para pacientes com FAPO.

**Quadro 32:** Recomendação para pacientes com FAPO

Recomendação	Classe de recomendação	Nível de evidência
A manutenção da anticoagulação em longo prazo deve ser considerada em pacientes com fibrilação atrial detectada após cirurgia não cardíaca, quando analisado risco de acidente vascular cerebral, conforme CHA_2_DS_2_VASc e risco de sangramento, de acordo com a cirurgia realizada.	IIa	B

### 6.3. Insuficiência Cardíaca Aguda

Com o envelhecimento da população mundial, a prevalência de IC está aumentando.^89^ Pacientes mais idosos e com múltiplas comorbidades são submetidos a operações não cardíacas, e a ocorrência de IC aguda como complicação pós-operatória (ICAp) de cirurgias não cardíacas provavelmente também irá aumentar.^93^ Não existem muitos estudos que avaliaram especificamente a ocorrência de ICAp. A incidência de ICAp em estudos retrospectivos ou estudos avaliando ICAp como parte do desfecho combinado de eventos CV maiores variou entre 1 e 3,8%.^406 - 412^

Em um estudo retrospectivo utilizando uma base de dados americana, 4,9% dos pacientes submetidos a operações não cardíacas apresentaram ICA durante a hospitalização (não especificado se pré ou pós-operatório), e a mortalidade intra-hospitalar desses pacientes foi superior à de pacientes sem ICA (5 vs. 0,8%, p < 0,001). A maioria dos casos ocorreu em cirurgias ortopédicas e vasculares.^93^

Em um estudo recente de coorte prospectiva envolvendo 11.162 operações não cardíacas, a incidência de ICAp foi de 2,5%. Os fatores de risco independentemente correlacionados com a ocorrência de ICAp foram idade, IC crônica, diabetes melito, FA, anemia, doença pulmonar obstrutiva crônica, DAC, doença arterial periférica, injúria miocárdica crônica e cirurgia de urgência e emergência. Cerca de 50% dos pacientes com ICAp não tinham história de IC conhecida no pré-operatório. A mortalidade desses pacientes com ICAp foi de 44% em 1 ano, comparado a 11% em pacientes sem ICAp (p < 0,001). Além disso, os pacientes com ICAp apresentaram uma taxa de re-hospitalização por ICA de 15%.^412^

Portanto, a ICAp é uma complicação perioperatória relacionada a altas taxas de mortalidade, que vem sendo negligenciada.

O diagnóstico da ICAp é dificultado porque os pacientes estão restritos ao leito, são idosos e geralmente não referem sintomas. Portanto, é necessário estar atento aos pacientes idosos, com fatores de risco submetidos a operações ortopédicas ou vasculares para a presença de ICAp. O diagnóstico é clínico e auxiliado pela dosagem dos peptídeos natriuréticos. A realização de um ecocardiograma está recomendada para avaliação da função sistólica biventricular e exclusão de doença valvar significativa.

O tratamento da ICAp deve seguir as recomendações das diretrizes atuais para o tratamento da ICA fora do contexto perioperatório.^413^ Cabe ressaltar que é recomendada a otimização do tratamento clínico medicamentoso antes da alta hospitalar para evitar as readmissões por ICA, assim como um retorno ambulatorial precoce para reavaliação clínica e da terapia medicamentosa.

O Quadro 33 apresenta as recomendações para pacientes com ICAp.

**Quadro 33:** Recomendações para pacientes com insuficiência cardíaca aguda pós-operatória

Recomendação	Classe de recomendação	Nível de evidência
Dosagem dos peptídeos natriuréticos e realização de ecocardiograma na internação	I	C
Tratamento de acordo com as diretrizes atuais e otimização do tratamento medicamentoso e da volemia antes da alta	I	C
Retorno ambulatorial precoce para reavaliação clínica e da terapia medicamentosa	I	C

### 6.4. Tromboembolismo Venoso

O tratamento de pacientes com diagnóstico de TEV no contexto perioperatório segue os princípios de tratamento em pacientes gerais. Deve-se atentar especialmente ao risco de sangramento associado aos procedimentos cirúrgicos. Nesse contexto, a equipe cirúrgica deve sempre ser consultada antes do início de terapia antitrombótica.

Na fase aguda do TEV, deve-se preferir o uso de heparina de baixo peso molecular (HBPM) ou fondaparinux à heparina não fracionada (HNF). Caso haja contraindicação a essas drogas ou risco elevado de sangramento, a HNF é o tratamento de escolha.^414 - 417^ Para os pacientes instáveis hemodinamicamente, a trombólise sistêmica é o tratamento de escolha. Entretanto, essa terapia frequentemente estará contraindicada no pós-operatório.^418 - 421^ Nesses casos, pode-se optar pela embolectomia pulmonar ou tratamento percutâneo por cateter.^422 - 425^

Para os pacientes com indicação de anticoagulação oral, o uso dos DOACs está consolidado como primeira escolha quando comparados aos antagonistas de vitamina K.^426 - 431^ Isso se deve aos menores índices de sangramento com o uso e à maior conveniência para os pacientes e profissionais de saúde (dose fixa, menores interações medicamentosas e com alimentos e ausência de necessidade de exames de sangue seriados para assegurar uma faixa terapêutica específica). Na presença de contraindicação absoluta à anticoagulação, o filtro de veia cava pode ser considerado.^432 - 434^

A duração da terapia anticoagulante é recomendada por um período mínimo de 3 meses.^435 , 436^ A extensão do tratamento normalmente reduz a recorrência de eventos tromboembólicos, mas aumenta a chance de sangramentos, conforme demostrado em vários estudos.^437^ Há vários critérios que devem ser avaliados antes da decisão de prolongar o tratamento, mas fogem do objetivo desta atualização.^438^

O Quadro 34 apresenta as recomendações para pacientes com TEV pós-operatório.

**Quadro 34:** Recomendações para pacientes com tromboembolismo venoso pós-operatório

Recomendação	Classe de recomendação	Nível de evidência
Pacientes instáveis com TEP		
Terapia trombolítica em pacientes instáveis com TEP na ausência de contraindicações	I	B
Embolectomia pulmonar em pacientes instáveis com contraindicação à terapia trombolítica ou quando a terapia trombolítica falha	I	C
Em pacientes com instabilidade hemodinâmica, deve-se optar por anticoagulação parenteral com HNF em detrimento de HBPM ou fondaparinux	I	C
Tratamento percutâneo por cateter em pacientes instáveis com contraindicação à terapia trombolítica, quando a terapia trombolítica falha ou com alto risco cirúrgico	IIa	C
**Anticoagulação/pacientes estáveis**		
Em pacientes com TEV e indicação de anticoagulação parenteral, deve-se preferir HBPM ou fondaparinux à HNF	I	A
Em pacientes com TEV e indicação de anticoagulação parenteral com contraindicação à HBPM ou fondaparinux, deve-se iniciar HNF	I	A
Em pacientes com TEV e indicação de anticoagulante oral, deve-se optar pelos DOACs em comparação à varfarina	I	A
Em pacientes com TEV e indicação de anticoagulante oral com contraindicação aos DOACs, deve-se indicar varfarina	I	A
O filtro de veia cava pode ser considerado em pacientes com contraindicação absoluta à anticoagulação	IIa	C
Pacientes com TEP devem receber tratamento com anticoagulante por um período de pelo menos 3 meses	I	A

1 TEV: tromboembolismo venoso; TEP: tromboembolismo pulmonar; HNF: heparina não fracionada; HBPM: heparina de baixo peso molecular; DOACs: anticoagulantes orais diretos.
