# Supplementary material for: Diretriz de Avaliação Cardiovascular Perioperatória da Sociedade Brasileira de Cardiologia – 2024
Source: Arq Bras Cardiol. 2024 Oct 9;121(9):e20240590. [Article in Portuguese] doi: 10.36660/abc.20240590 (PMC12094288; doi:10.36660/abc.20240590)
Supplement: Translation

# Translation: Guideline for Perioperative Cardiovascular Evaluation of the Brazilian Society of Cardiology – 2024

**DOI:** 10.36660/abc.20240590i

**Authors:** Danielle Menosi Gualandro, Luciana Savoy Fornari, Bruno Caramelli, Alexandre Antonio Cunha Abizaid, Brenno Rizerio Gomes, Caio de Assis Moura Tavares, Caio Julio Cesar dos Santos Fernandes, Carisi Anne Polanczyk, Carlos Jardim, Carolina Leticia Zilli Vieira, Claudio Pinho, Daniela Calderaro, Dirk Schreen, Fabiana Goulart Marcondes-Braga, Fábio de Souza, Francisco Akira Malta Cardozo, Flavio Tarasoutchi, Gabriel Assis Lopes Carmo, Gabriel Kanhouche, José Jayme Galvão de Lima, Luciana Dornfeld Bichuette, Luciana Sacilotto, Luciano Ferreira Drager, Luciano Janussi Vacanti, Luis Henrique Wolff Gowdak, Marcelo Luiz Campos Vieira, Marcelo Luiz Floriano Melo Martins, Márcio Silva Miguel Lima, Marcos Pita Lottenberg, Márlon Juliano Romero Aliberti, Mauricio Felippi de Sá Marchi, Milena Ribeiro Paixão, Mucio Tavares de Oliveira, Pai Ching Yu, Patricia Ramos Cury, Pedro Silvio Farsky, Ranna Santos Pessoa, Rinaldo Focaccia Siciliano, Tarso Augusto Duenhas Accorsi, Vinícius Machado Correia, Wilson Mathias

**Affiliations:** 1 São Paulo SP Brazil Instituto do Coração (InCor) do Hospital das Clínicas da Faculdade de Medicina da Universidade de São Paulo (HCFMUSP), São Paulo, SP – Brazil; 2 Basel Switzerland University Hospital Basel, Basel – Switzerland; 3 São Paulo SP Brazil Fundação Zerbini, São Paulo, SP – Brazil; 4 Natal RN Brazil Hospital Rio Grande, Natal, RN – Brazil; 5 São Paulo SP Brazil Hospital Israelita Albert Einstein, São Paulo, SP – Brazil; 6 Porto Alegre RS Brazil Hospital de Clínicas da Universidade Federal do Rio Grande do Sul (UFRS), Porto Alegre, RS – Brazil; 7 São Paulo SP Brazil Hospital das Clínicas da Faculdade de Medicina da Universidade de São Paulo (HCFMUSP), São Paulo, SP – Brazil; 8 Massachusetts USA Harvard University, Massachusetts – USA; 9 Campinas SP Brazil Pontifícia Universidade Católica de Campinas (PUC-Campinas), Campinas, SP – Brazil; 10 Campinas SP Brazil Clinica Pinho, Campinas, SP – Brazil; 11 Fortaleza CE Brazil Hospital São Carlos, Rede D’Or, Fortaleza, CE – Brazil; 12 Fortaleza CE Brazil Hospital Universitário Walter Cantidio da Universidade Federal do Ceará (UFC), Fortaleza, CE – Brazil; 13 Fortaleza CE Brazil Instituto de Medicina Nuclear, Fortaleza, CE – Brazil; 14 Rio de Janeiro RJ Brazil Escola de Medicina e Cirurgia da Universidade Federal do Estado do Rio de Janeiro (UNIRIO), Rio de Janeiro, RJ – Brazil; 15 Belo Horizonte MG Brazil Universidade Federal de Minas Gerais, Belo Horizonte, MG – Brazil; 16 Belo Horizonte MG Brazil Hospital Evangélico de Belo Horizonte, Belo Horizonte, MG – Brazil; 17 Belo Horizonte MG Brazil Hospital Orizonti, Belo Horizonte, MG – Brazil; 18 Brasília DF Brazil Hospital de Base do Distrito Federal, Brasília, DF – Brazil; 19 Brasília DF Brazil Centro Universitário Euroamericano, Brasília, DF – Brazil; 20 Teresina PI Brazil Hospital São Marcos, Teresina, PI – Brazil; 21 Salvador BA Brazil Universidade Federal da Bahia, Salvador, BA – Brazil; 22 São Paulo SP Brazil Instituto Dante Pazzanese de Cardiologia, São Paulo, SP – Brazil

**Table:** 

Guideline for Perioperative Cardiovascular Evaluation of the Brazilian Society of Cardiology – 2024	
The report below lists declarations of interest as reported to the SBC by the experts during the period of the development of these statement, 2023-2024.	
Expert	Type of relationship with industry
Alexandre Antonio Cunha Abizaid	Nothing to be declared
Brenno Rizerio Gomes	Financial declaration A - Economically relevant payments of any kind made to (i) you, (ii) your spouse/partner or any other person living with you, (iii) any legal person in which any of these is either a direct or indirect controlling owner, business partner, shareholder or participant; any payments received for lectures, lessons, training instruction, compensation, fees paid for participation in advisory boards, investigative boards or other committees, etc. From the brazilian or international pharmaceutical, orthosis, prosthesis, equipment and implants industry: – Astrazeneca: Forxiga; Novartis: Entresto; Bayer: Firialta. B - Research funding under your direct/personal responsibility (directed to the department or institution) from the Brazilian or international pharmaceutical, orthosis, prosthesis, equipment and implants industry: – B. Braun: Occlutech.
Bruno Caramelli	Nothing to be declared
Caio de Assis Moura Tavares	Financial declaration A - Economically relevant payments of any kind made to (i) you, (ii) your spouse/partner or any other person living with you, (iii) any legal person in which any of these is either a direct or indirect controlling owner, business partner, shareholder or participant; any payments received for lectures, lessons, training instruction, compensation, fees paid for participation in advisory boards, investigative boards or other committees, etc. From the brazilian or international pharmaceutical, orthosis, prosthesis, equipment and implants industry: – Novo Nordisk.
Caio Julio Cesar dos Santos Fernandes	Financial declaration A - Economically relevant payments of any kind made to (i) you, (ii) your spouse/partner or any other person living with you, (iii) any legal person in which any of these is either a direct or indirect controlling owner, business partner, shareholder or participant; any payments received for lectures, lessons, training instruction, compensation, fees paid for participation in advisory boards, investigative boards or other committees, etc. From the brazilian or international pharmaceutical, orthosis, prosthesis, equipment and implants industry: – Janssen: Uptravi; Bayer: Riociguate; MSD: Sotatercept. B - Research funding under your direct/personal responsibility (directed to the department or institution) from the Brazilian or international pharmaceutical, orthosis, prosthesis, equipment and implants industry: – Janssen: Uptravii. Other relationships Funding of continuing medical education activities, including travel, accommodation and registration in conferences and courses, from the brazilian or international pharmaceutical, orthosis, prosthesis, equipment and implants industry: – Janssen: Uptravii.
Carisi Anne Polanczyk	Nothing to be declared
Carlos Jardim	Nothing to be declared
Carolina Leticia Zilli Vieira	Nothing to be declared
Claudio Pinho	Nothing to be declared
Daniela Calderaro	Financial declaration A - Economically relevant payments of any kind made to (i) you, (ii) your spouse/partner or any other person living with you, (iii) any legal person in which any of these is either a direct or indirect controlling owner, business partner, shareholder or participant; any payments received for lectures, lessons, training instruction, compensation, fees paid for participation in advisory boards, investigative boards or other committees, etc. From the brazilian or international pharmaceutical, orthosis, prosthesis, equipment and implants industry: – Bayer: atrial fibrillation, VTE, diabetic nephropathy. Other relationships Funding of continuing medical education activities, including travel, accommodation and registration in conferences and courses, from the brazilian or international pharmaceutical, orthosis, prosthesis, equipment and implants industry: – Bayer: diabetic nephropathy.
Danielle Menosi Gualandro	Financial declaration A - Economically relevant payments of any kind made to (i) you, (ii) your spouse/partner or any other person living with you, (iii) any legal person in which any of these is either a direct or indirect controlling owner, business partner, shareholder or participant; any payments received for lectures, lessons, training instruction, compensation, fees paid for participation in advisory boards, investigative boards or other committees, etc. From the brazilian or international pharmaceutical, orthosis, prosthesis, equipment and implants industry: – Roche.
Dirk Schreen	Nothing to be declared
Fabiana Goulart Marcondes-Braga	Financial declaration A - Economically relevant payments of any kind made to (i) you, (ii) your spouse/partner or any other person living with you, (iii) any legal person in which any of these is either a direct or indirect controlling owner, business partner, shareholder or participant; any payments received for lectures, lessons, training instruction, compensation, fees paid for participation in advisory boards, investigative boards or other committees, etc. From the brazilian or international pharmaceutical, orthosis, prosthesis, equipment and implants industry: – Lectures and Advisory Committee: AstraZeneca; Boehringher-Lilly; Novartis; Bayer. B - Research funding under your direct/personal responsibility (directed to the department or institution) from the Brazilian or international pharmaceutical, orthosis, prosthesis, equipment and implants industry: – MSD: Victor Trial
Fábio de Souza	Financial declaration A - Economically relevant payments of any kind made to (i) you, (ii) your spouse/partner or any other person living with you, (iii) any legal person in which any of these is either a direct or indirect controlling owner, business partner, shareholder or participant; any payments received for lectures, lessons, training instruction, compensation, fees paid for participation in advisory boards, investigative boards or other committees, etc. From the brazilian or international pharmaceutical, orthosis, prosthesis, equipment and implants industry: – Pfizer: Amiloidose. Other relationships Funding of continuing medical education activities, including travel, accommodation and registration in conferences and courses, from the brazilian or international pharmaceutical, orthosis, prosthesis, equipment and implants industry: – Pfizer: amyloidosis; AstraZeneca: amyloidosis.
Flávio Tarasoutchi	Other relationships Funding of continuing medical education activities, including travel, accommodation and registration in conferences and courses, from the brazilian or international pharmaceutical, orthosis, prosthesis, equipment and implants industry: – Edwads.
Francisco Akira Malta Cardozo	Financial declaration A - Economically relevant payments of any kind made to (i) you, (ii) your spouse/partner or any other person living with you, (iii) any legal person in which any of these is either a direct or indirect controlling owner, business partner, shareholder or participant; any payments received for lectures, lessons, training instruction, compensation, fees paid for participation in advisory boards, investigative boards or other committees, etc. From the brazilian or international pharmaceutical, orthosis, prosthesis, equipment and implants industry: – Bayer: Xarelto; Daiichi Sankyo: Lixiana; Novo Nordisk: Rybelsius. Other relationships Funding of continuing medical education activities, including travel, accommodation and registration in conferences and courses, from the brazilian or international pharmaceutical, orthosis, prosthesis, equipment and implants industry: – Daiichi Sankyo.
Gabriel Assis Lopes do Carmo	Nothing to be declared
Gabriel Kanhouche	Nothing to be declared
José Jayme Galvão de Lima	Nothing to be declared
Luciana Dornfeld Bichuette	Nothing to be declared
Luciana Sacilotto	Nothing to be declared
Luciana Savoy Fornari	Nothing to be declared
Luciano Ferreira Drager	Nothing to be declared
Luciano Janussi Vacanti	Nothing to be declared
Luis Henrique Wolff Gowdak	Financial declaration A - Economically relevant payments of any kind made to (i) you, (ii) your spouse/partner or any other person living with you, (iii) any legal person in which any of these is either a direct or indirect controlling owner, business partner, shareholder or participant; any payments received for lectures, lessons, training instruction, compensation, fees paid for participation in advisory boards, investigative boards or other committees, etc. From the brazilian or international pharmaceutical, orthosis, prosthesis, equipment and implants industry: – Servier: síndrome coronariana crônica; Novartis: hipercolesterolemia. B - Research funding under your direct/personal responsibility (directed to the department or institution) from the Brazilian or international pharmaceutical, orthosis, prosthesis, equipment and implants industry: – Servier: chronic coronary syndrome. Other relationships Funding of continuing medical education activities, including travel, accommodation and registration in conferences and courses, from the brazilian or international pharmaceutical, orthosis, prosthesis, equipment and implants industry: – Servier: chronic coronary syndrome.
Marcelo Luiz Campos Vieira	Nothing to be declared
Marcelo Luiz Floriano Melo Martins	Nothing to be declared
Márcio Silva Miguel Lima	Nothing to be declared
Marcos Pita Lottenberg	Nothing to be declared
Márlon Juliano Romero Aliberti	Nothing to be declared
Mauricio Felippi de Sá Marchi	Nothing to be declared
Milena Ribeiro Paixão	Nothing to be declared
Mucio Tavares de Oliveira Junior	Financial declaration B - Research funding under your direct/personal responsibility (directed to the department or institution) from the Brazilian or international pharmaceutical, orthosis, prosthesis, equipment and implants industry: – Sanofi; Pasteur.
Pai Ching Yu	Nothing to be declared
Patricia Ramos Cury	Nothing to be declared
Pedro Silvio Farsky	Financial declaration A - Economically relevant payments of any kind made to (i) you, (ii) your spouse/partner or any other person living with you, (iii) any legal person in which any of these is either a direct or indirect controlling owner, business partner, shareholder or participant; any payments received for lectures, lessons, training instruction, compensation, fees paid for participation in advisory boards, investigative boards or other committees, etc. From the brazilian or international pharmaceutical, orthosis, prosthesis, equipment and implants industry: – Novo Nordisk: semaglutide; Lilly: empagliflozin and tirzepatide; AstraZeneca: dapagliflozin; GSK: shingrix vaccine. Other relationships Funding of continuing medical education activities, including travel, accommodation and registration in conferences and courses, from the brazilian or international pharmaceutical, orthosis, prosthesis, equipment and implants industry: – Novo Nordisk: semaglutide.
Ranna Santos Pessoa	Nothing to be declared
Rinaldo Focaccia Siciliano	Nothing to be declared
Tarso Augusto Duenhas Accorsi	Financial declaration A - Economically relevant payments of any kind made to (i) you, (ii) your spouse/partner or any other person living with you, (iii) any legal person in which any of these is either a direct or indirect controlling owner, business partner, shareholder or participant; any payments received for lectures, lessons, training instruction, compensation, fees paid for participation in advisory boards, investigative boards or other committees, etc. From the brazilian or international pharmaceutical, orthosis, prosthesis, equipment and implants industry: – Abbott: MitraClip; Boston: Watchman; Edwards: surgery and TAVI; Novartis: Sybrava. Other relationships Funding of continuing medical education activities, including travel, accommodation and registration in conferences and courses, from the brazilian or international pharmaceutical, orthosis, prosthesis, equipment and implants industry: – Novo Nordisk: Brazilian SBC Congress; EMS: SBC Departments Congress.
Vinícius Machado Correia	Financial declaration A - Economically relevant payments of any kind made to (i) you, (ii) your spouse/partner or any other person living with you, (iii) any legal person in which any of these is either a direct or indirect controlling owner, business partner, shareholder or participant; any payments received for lectures, lessons, training instruction, compensation, fees paid for participation in advisory boards, investigative boards or other committees, etc. From the brazilian or international pharmaceutical, orthosis, prosthesis, equipment and implants industry: – Novo Nordisk: lecture on pharmacological treatment of obesity.
Wilson Mathias Junior	Nothing to be declared

**Table of Contents**

**1. Definition of the Problem** 8

1.1. Objective of the Guideline 8

1.2. Methodology and Levels of Evidence 8

1.3. Particularities of the Perioperative Period 8

1.4. Creation of the Perioperative Risk Team 8

**2. General and Additional Preoperative Assessment** 11

2.1. Preoperative Risk Stratification 11

**2.1.1. Severe/unstable CV Conditions in the Perioperative Period** 11

**2.1.2. Estimation of Intrinsic Risk Related to Type of Surgery** 11

**2.1.3. Functional Capacity Assessment** 11

**2.1.4. Tools for Estimating Perioperative CV Risk** 12

**2.1.5. Frailty Assessment** 13

***2.1.5.1. Frailty Assessment Before Noncardiac Surgery*** 14

***2.1.5.2. Impact of Frailty Assessment on Noncardiac Surgery*** 14

2.2. Electrocardiography 15

2.3. Echocardiography 16

2.4. Noninvasive Tests for Myocardial Ischemia 17

**2.4.1. Exercise Electrocardiogram** 17

**2.4.2. Myocardial Perfusion Imaging** 17

**2.4.3. Dobutamine Stress Echocardiography** 18

**2.4.4. Summary of Recommendations for Noninvasive Functional**

**Imaging for Myocardial Ischaemia** 18

2.5. Coronary Cineangiography 18

2.6. Coronary Computed Tomography Angiography 18

**3. Specific Diseases and Procedures in the Perioperative**

**Period** 19

3.1. Heart Failure 19

3.2. Arterial Hypertension 20

**3.2.1. Preoperative BP Management** 20

**3.2.2. Intraoperative BP Management** 21

**3.2.3. Postoperative BP Management** 21

**3.2.4. Perioperative Management of Antihypertensives** 21

***3.2.4.1. Renin-Angiotensin-Aldosterone System Inhibitors*** 21

***3.2.4.2. Calcium Channel Blockers*** 22

***3.2.4.3. Diuretics*** 22

***3.2.4.4. Central Sympatholytic Drugs*** 22

**3.2.5. Final Considerations** 22

3.3. Patient Management in Low-Risk Procedures 22

**3.3.1. Dental Procedures** 22

***3.3.1.1. Antithrombotic agents*** 22

***3.3.1.2. Cardiac Implantable Electronic Devices*** 23

***3.3.1.3. Local Anesthetics*** 23

**3.3.2. Dermatological Surgery** 23

**3.3.3. Endoscopic Procedures** 24

***3.3.3.1. Management of Antiplatelet Therapy in Endoscopic***

***Procedures*** 24

***3.3.3.2. Management of Anticoagulants in Endoscopic Procedures*** 25

**3.3.4. Ophthalmic Procedures** 25

3.4. Valvular Heart Disease 26

**3.4.1. Aortic Valve Stenosis** 26

**3.4.2. Mitral Valve Stenosis** 27

**3.4.3. Aortic and Mitral Regurgitation** 27

**3.4.4. Prosthetic Heart Valves** 27

3.5. Solid Organ Transplantation 27

**3.5.1 Liver** 27

***3.5.1.1. Cirrhotic Cardiomyopathy*** 28

***3.5.1.2. Alcoholic Cardiomyopathy*** 28

***3.5.1.3. Portopulmonary Hypertension*** 28

***3.5.1.4. Hepatopulmonary Syndrome*** 29

***3.5.1.5. Coronary Artery Disease*** 30

***3.5.1.5.1. When to Investigate CAD?*** 30

***3.5.1.5.2. Which Test to Request for CAD Investigation?*** 30

***3.5.1.5.3. When and How to Intervene in CAD?*** 30

**3.5.2. Kidney** 30

***3.5.2.1. CAD Risk Stratification*** 32

**4. Measures for Reducing CV Surgical Risk** 33

4.1. Perioperative Pharmacological Therapy 33

**4.1.1. Beta-Blockers** 33

**4.1.2. Statins** 33

**4.1.3. Anti-Platelet Medications** 34

***4.1.3.1. Monotherapy with ASA*** 34

***4.1.3.2. Monotherapy with Antiplatelet Medications Other than ASA*** 35

***4.1.3.3. Dual Antiplatelet Therapy*** 35

4.2. Myocardial Revascularization 36

4.3. Perioperative Anticoagulation Management 36

**4.3.1. Warfarin** 37

***4.3.1.1. Patients with Moderate Thromboembolic Risk*** 37

***4.3.1.2. Urgent or Emergency Procedures*** 37

**4.3.2. Direct Oral Anticoagulants** 38

***4.3.2.1. Dabigatran*** 39

***4.3.2.2. Rivaroxaban*** 39

***4.3.2.3. Apixaban*** 39

***4.3.2.4. Edoxaban*** 40

***4.3.2.5. Evaluation of the Anticoagulant Effect of DOACs*** 40

***4.3.2.6. Interruption of DOACs Before Elective Surgery*** 40

4.4. Infective Endocarditis Prophylaxis 41

**4.4.1. Dental Procedures** 41

**4.4.2. Respiratory Tract Procedures** 43

**4.4.3. Gastrointestinal and Genitourinary Procedures** 43

**4.4.4. Dermatological or Musculoskeletal Procedures** 43

**4.4.5. Body Piercing and Tattooing** 43

**5. Perioperative Biomarkers** 44

5.1. Natriuretic Peptides 44

5.2. Cardiac Troponins and Surveillance of CV Complications 44

**6. Diagnosis and Treatment of Perioperative CV**

**Complications** 44

6.1. Perioperative Acute Myocardial Infarction/Injury (PMI) 44

6.2. Acute Atrial Fibrillation/Flutter 48

6.3. Acute Heart Failure 49

6.4. Venous Thromboembolism 50

**References** 51

## 1. Definition of the Problem

### 1.1. Objective of the Guideline

The rapid advancement of medical knowledge in recent years prompted the creation of guidelines to filter the best evidence and organize it for practical use in daily medical practice. The need for updates varies by field and reflects the quantity and quality of medical research on a given subject. Interest in perioperative care for noncardiac surgery is relatively recent, starting in 1996 after the publication of Mangano et al.’s study, which observed a reduction in cardiovascular (CV) complications in patients who received intravenous atenolol in the immediate preoperative period.^1^In Brazil, publications began emerging in 2003, with the first Guideline for Perioperative Cardiovascular Evaluation being published in 2007 and updated in 2011 and 2017.^2^This is the fourth update in 15 years, highlighting the topic’s importance in both research and daily professional practice.

Although the percentage of perioperative complications is small, their occurrence in Brazil is significantly higher than in other countries. Additionally, most procedures are low risk, but considering the annual volume of over 8 million surgical interventions in Brazil, there is a substantial number of patients at high risk of complications who require greater care.

The main objectives of the Guideline for Perioperative Cardiovascular Evaluation of the Brazilian Society of Cardiology – 2024 are:

To provide and standardize updated knowledge on CV risk stratification.

To establish adequate methods for risk stratification and diagnosis of complications, not only to improve efficiency but also to reduce costs associated with unnecessary tests.

To offer updated knowledge on the relationship between different comorbidities and CV diseases (CVDs) that may interact in the perioperative setting, such as the interaction and adequate discontinuation of medications or the safe timeframe for performing surgical procedures after CV interventions.

To offer recommendations on the optimal location for performing interventions in high-risk patients or on contraindications to procedures when the risk of complications outweighs the potential benefits.

### 1.2. Methodology and Levels of Evidence

The methodology and levels of evidence adopted by this Guideline are the same as those adopted by the SBC and referred to below:

Classes (grades) of recommendation:

✓ Class I – Conditions for which there is conclusive evidence or a consensus that the procedure is safe and useful/effective.
✓ Class II – Conditions for which there is conflicting evidence and/or a divergence of opinion about the safety and usefulness/efficacy of a procedure.
✓ Class IIA – Evidence or opinion in favor of the procedure. The majority agrees.
✓ Class IIB – Safety and usefulness are less well established, and there is no predominance of opinions in favor of the method.
✓ Class III – Conditions for which there is evidence and/or general agreement that a procedure is not useful/effective and, in some cases, may be harmful.

Levels of evidence:

✓ Level A – Data obtained from several large, randomized studies showing concurring results and/or a robust meta-analysis of randomized controlled trials.
✓ Level B – Data obtained from a less robust meta-analysis, a single randomized study, or from nonrandomized (observational) studies.
✓ Level C – Data obtained from consensual expert opinions.

All scientific evidence used in this Guideline comes from indexed journals with qualified editorial boards and peer reviewers.

### 1.3. Particularities of the Perioperative Period

In patients with heart disease, the perioperative period presents many peculiarities, such as the use of anticoagulation and antithrombotic therapy, which sometimes should not be interrupted and could increase the risk of intraoperative bleeding. Importantly, CV risk stratification tools, such as the ATP-III, Framingham, and CHADS-VASc scores, which are familiar to cardiologists, establish risk profiles over years and are not suitable for predicting short-term perioperative risk for noncardiac surgery (usually less than 30 days). For these reasons, the Guideline provides relevant information specifically for assessing the risk of CV events, prevention methods, and recommendations to make the perioperative period of noncardiac procedures safer for patients with heart disease. A summary of the new recommendations included in this Guideline is presented in Table 1 .

**Table 1:** New recommendations included in the Guideline for Perioperative Cardiovascular Evaluation

Recommendation		Class
General and additional preoperative assessment: Preoperative risk stratification		
**Functional capacity assessment**		
The functional capacity of patients scheduled for intermediate- or high-risk surgery should be determined during medical history (based on the ability to climb two flights of stairs).		**I**
**Frailty assessment**		
Frailty should be routinely assessed in elderly patients scheduled for intermediate- or high-risk surgery.		**IIa**
Frailty should be objectively measured using specific instruments.		**IIa**
**Electrocardiography**		
Patients undergoing surgery with intermediate or high intrinsic risk of cardiovascular complications.		**I**
Patients at intermediate or high risk for perioperative cardiovascular events as estimated by algorithms.		**I**
**Noninvasive tests for myocardial ischemia**		
Exercise electrocardiogram		
	An exercise electrocardiogram should be requested in intermediate- and high-risk patients with reduced functional capacity scheduled for intermediate- or high-risk elective noncardiac surgery in whom functional testing could potentially alter management.	**IIb**
Myocardial perfusion imaging/stress echocardiography		
	Imaging stress testing in intermediate- and high-risk patients with reduced functional capacity scheduled for intermediate- or high-risk elective noncardiac surgery in whom functional testing could potentially alter management.	**IIa**
	Imaging stress testing in asymptomatic patients with reduced functional capacity and a previous diagnosis or high probability of coronary artery disease.	**IIb**
**Specific diseases and procedures in the perioperative period**		
**Arterial hypertension**		
Episodes of hypotension should be avoided throughout the perioperative period.		**I**
Patients with suspected secondary hypertension should be investigated before surgery, except in emergency/urgent cases.		**I**
Chronic therapy with renin-angiotensin-aldosterone system antagonists may be maintained in the perioperative period; discontinuation is allowed in selected cases.		**I**
Chronic therapy with calcium channel blockers may be maintained in the perioperative period; discontinuation is allowed in selected cases.		**I**
Chronic therapy with diuretics may be maintained in the perioperative period; discontinuation is allowed in selected cases.		**I**
Chronic therapy with clonidine should be maintained in the perioperative period.		**I**
**Patient management in low-risk procedures**		
Dental procedures		
	Dental evaluation and treatment are important in the perioperative period.	**IIa**
	Use of antimicrobial mouthwash before and after dental procedures and before intermediate- and high-risk noncardiac surgery.	**IIa**
	The use of 1-4 anesthetic cartridges with vasoconstrictor is safe for dental treatment in patients with heart disease.	**IIa**
	Antiplatelet and anticoagulant therapy should be maintained during most dental treatments, including extraction of up to 2 teeth, restorations, prosthetics, endodontics, cleaning, and implants.	**IIa**
**Valvular heart disease**		
Asymptomatic patients with significant mitral stenosis and other risk factors (pulmonary artery systolic pressure [PASP] ≥ 50 mm Hg at rest or PASP ≥ 60 mm Hg on exertion) should undergo correction of mitral stenosis before noncardiac surgery.		**I**
**Solid organ transplantation**		
Liver		
	In patients with an echocardiogram showing a pulmonary artery systolic pressure (PASP) > 50 mm Hg, right heart catheterization with measurement of pulmonary artery pressure must be requested.	**I**
	In patients with an echocardiogram showing a PASP > 40 mm Hg, especially if other signs of pulmonary hypertension (PH) are present, right heart catheterization with measurement of pulmonary artery pressure should be considered.	**IIa**
	In asymptomatic patients with coronary artery disease (CAD) without segmental dysfunction on echocardiography and with 2 or more risk factors for CAD * , DSE should be preferably requested.	**IIa**
	In patients with symptoms suggestive of CAD, the pretest probability of CAD should be calculated, and additional tests should be requested according to specific guidelines.	**IIa**
	Coronary cineangiography should be performed in patients with a high pretest probability of CAD, significant angina refractory to clinical treatment, new left ventricular dysfunction, or high-risk findings on noninvasive tests, despite hemorrhagic complications being more common and alterations such as elevated creatinine potentially contributing to increased morbidity in patients with cirrhosis.	**IIa**
	Coronary artery bypass graft before transplantation should be reserved only for patients in whom the risk of death from CAD exceeds the risk of death from liver disease, and this decision should be discussed with a multidisciplinary team.	**IIa**
	Patients with a mPAP ≥ 35 mm Hg on right heart catheterization should be referred to a PH specialist.	**IIb**
Kidney		
	All kidney transplant candidates should be evaluated for the presence and severity of cardiovascular disease based on clinical history, physical examination, and routine tests.	**I**
	Measurement of high-sensitivity troponin is recommended before and 24 and 48 hours after kidney transplantation to detect perioperative infarction/injury.	**I**
	Diagnostic decision-making and the definition of a therapeutic strategy should be discussed by a Heart-Kidney Team, including a clinical cardiologist, interventional cardiologist, cardiovascular surgeon, nephrologist, and/or kidney transplant specialist.	**I**
	Stable patients with obstructive CAD should be clinically reassessed for disease progression every 12 months; patients without significant obstructive CAD should be reassessed every 36 months to detect *de novo* CAD.	**IIa**
**Measures for reducing cardiovascular surgical risk**		
**Perioperative pharmacological therapy**		
Beta-blockers		
	Chronic therapy (> 7 days) with beta-blockers should be maintained perioperatively.	**I**
Statins		
	Patients undergoing nonvascular surgery with clinical indications for statin use due to associated diseases (coronary artery disease, cerebrovascular disease, peripheral arterial disease, diabetes), regardless of the perioperative setting.	**IIa**
Dual antiplatelet therapy		
	For early interruption of dual antiplatelet therapy (DAPT) before the minimum duration time, noncardiac surgery should be performed in centers with multidisciplinary care and hemodynamic monitoring.	**I**
	A platelet aggregation test should be used to reduce the discontinuation time of P2Y12 inhibitors before noncardiac surgery.	**IIb**
	In cases with very high thrombotic risk (less than 1 month since percutaneous coronary intervention and DAPT interruption), bridging therapy with tirofiban should be used.	**IIb**
	Routine platelet aggregation testing should be performed to assess the discontinuation of ASA or P2Y12 inhibitors before noncardiac surgery.	**III**
**Myocardial revascularization**		
Recommendations for the interval between myocardial revascularization and noncardiac surgery in patients undergoing elective percutaneous coronary interventions:		
	– ≥ 6 months	**I**
	– Between 3 and 6 months	**IIa**
	– Between 30 days and 3 months	**IIb**
	– < 30 days	**III**
Recommendations for the interval between myocardial revascularization and noncardiac surgery in patients undergoing percutaneous coronary interventions due to acute coronary syndromes:		
	– ≥ 12 months	**I**
	– Between 6 and 12 months	**IIa**
	– Between 30 days and 6 months	**IIb**
	– < 30 days	**III**
**Perioperative biomarkers**		
**Natriuretic peptides**		
Patients older than 65 years or patients aged 45-64 years with established cardiovascular disease or risk factors * undergoing noncardiac surgery.		**I**
**Cardiac troponins and surveillanceof CV complications**		
High-risk patients according to algorithms undergoing intermediate- or high-risk noncardiac surgery should stay in the ICU for 48 hours after surgery.		**I**
Intermediate-risk patients according to algorithms undergoing intermediate- or high-risk noncardiac surgery should stay in the ICU for 48 hours after surgery.	**IIa**
**Diagnosis and treatment of perioperative CV complications**		
**Perioperative acute myocardial infarction/injury (PMI)**		
PMI diagnosis should be made in the presence of an absolute delta ≥ the 99th percentile of the upper reference limit of the troponin assay between the preoperative value and the value on postoperative day 1 or 2, or between two postoperative concentrations if the preoperative value is missing.		**I**
Diagnosis of AMI after postoperative day 2 should be based on the universal definition of MI, and treatment should be based on current guidelines.		**I**
In patients with perioperative AMI or PMI due to ischemia, all secondary causes of ischemia (anemia, tachycardia, hypotension, hypertension) should be treated, the risk of bleeding should be determined, and multidisciplinary discussion with the surgeon should be conducted.		**I**
**Acute atrial fibrillation/flutter**		
Long-term anticoagulation should be considered in patients with AF detected after noncardiac surgery, when stroke risk is assessed according to CHA2DS2VASc score and bleeding risk according to the surgery performed.		**IIa**
**Venous thromboembolism**		
In hemodynamically unstable patients, parenteral anticoagulation with unfractionated heparin (UFH) is preferred over low-molecular-weight heparin (LMWH) or fondaparinux.		**I**
In patients with venous thromboembolism (VTE) indicated for parenteral anticoagulation, LMWH or fondaparinux is preferred over UFH.		**I**

* Diabetes, hypertension, coronary artery disease, obesity and atrial fibrillation.

### 1.4. Creation of the Perioperative Risk Team

This Guideline focuses mainly, but not exclusively, on cardiologists, given that the most serious complications involve the CV system, such as ischemic heart disease, pulmonary embolism (PE), and acute heart failure (HF). These complications are most associated with mortality, increased length of hospital stay, and higher costs, not only due to additional procedures but also due to the request for diagnostic tests. Patients entering operating rooms today are older and consequently have more comorbidities, requiring the involvement not only of the surgeon but also the anesthesiologist, cardiologist, and other specialists in their treatment. The follow-up of high-risk patients should be interdisciplinary, including adequate planning to determine the best time for the intervention and the risk/benefit ratio, as well as to optimize the treatment of underlying diseases and their complications.

Cardiologists are already familiar with the Heart Team, typically consisting of the cardiac surgeon, the interventional cardiologist, and the clinical cardiologist, who usually work in the preoperative period to decide the best approach for a given case. The perioperative care of patients undergoing noncardiac surgery, which includes both the entire intraoperative and postoperative periods, involves a greater number of specialists from different fields. The Guideline for Perioperative Cardiovascular Evaluation of the Brazilian Society of Cardiology – 2024 innovates by proposing the creation of a Perioperative Risk Team (PRT). The purpose of the PRT, indicated for more severe cases, is to gather all relevant patient information, such as previous diseases and treatments, prognosis, type of proposed surgery, and current cardiac status. With harmony and teamwork among various specialists, it will be possible to offer the best joint decision for the patient and their family. Since the most serious complications involve the CV system, the cardiologist should plan, when necessary, the formation of the PRT for severe and specific cases and establish its dynamics. Tools such as video platforms, for recording and storing interdisciplinary meetings, can be very useful to improve outcomes and provide guarantees for both health care professionals and the patient.

## 2. General and Additional Preoperative Assessment

### 2.1. Preoperative Risk Stratification

#### 2.1.1. Severe/Unstable CV Conditions in the Perioperative Period

For elective procedures, the first step is to determine the patient’s baseline clinical condition. There are clinical circumstances in which the spontaneous risk of complications is very high, regardless of the surgical procedure to be performed. Identifying these circumstances is crucial because treating such conditions should take priority over elective procedures, which should be postponed whenever possible and reconsidered only after the patient has stabilized ( Table 2 ).

**Table 2:** Severe/unstable cardiovascular conditions in the perioperative period

Acute coronary syndrome
Unstable thoracic aortic disease
Acute pulmonary edema
Cardiogenic shock
NYHA class III/IV heart failure *
CCS class III/IV angina *
Symptomatic severe aortic/mitral stenosis
Severe bradyarrhythmias or tachyarrhythmias (complete AV block, VT)
Atrial fibrillation with high ventricular response (HR > 120 bpm)
Uncontrolled hypertension (BP > 180 x 110 mm Hg)
Symptomatic pulmonary hypertension

* Patients with these conditions who are stable and receiving optimized treatment should have the risk/benefit ratio of the surgery assessed due to the risk of complications. AV: atrioventricular; BP: blood pressure; CCS: Canadian Cardiovascular Society; HR: heart rate; NYHA: New York Heart Association; VT: ventricular tachycardia.

#### 2.1.2. Estimation of Intrinsic Risk Related to the Type of Surgery

The intrinsic risk of surgery is determined by the type and duration of the procedure, without considering the patient’s clinical characteristics. It is defined as the probability of CV events occurring perioperatively, regardless of the clinical variables of the patients. This risk is related to the duration of surgery, hemodynamic stress, and loss of blood and fluids. Patients with stable clinical conditions, who do not have high-risk CV conditions, may undergo low-intrinsic risk procedures without the need for additional evaluation. Despite the difficulty in determining the specific risk of a surgical procedure, as each procedure occurs under different circumstances, the European Society of Cardiology proposed a CV risk classification that considers the risk of CV death, acute myocardial infarction (AMI), and stroke within 30 days ( Table 3 ).

**Table 3:** Cardiovascular risk classification according to type of surgery

Low risk (< 1%)	Intermediate risk (1–5%)	High risk (> 5%)
Breast	Carotid asymptomatic	Aortic and *major* vascular surgery
Dental	Carotid endarterectomy (symptomatic)	Open lower limb revascularization for acute limb ischemia or amputation
Thyroid	Peripheral arterial angioplasty	Carotid angioplasty (symptomatic)
Eye	Endovascular aortic aneurysm	Adrenalectomy
Gynecological ( *minor* )	Head and neck surgery	Pancreatic surgery
Orthopedic ( *minor* , eg, meniscectomy)	Intraperitoneal (eg, splenectomy, hiatal hernia repair, cholecystectomy)	Liver resection, bile duct surgery
Reconstructive	Intrathoracic ( *nonmajor* )	Esophagectomy
Superficial surgery	Neurological or orthopedic ( *major* , eg, hip and spine surgery)	Pneumonectomy (VATS or open surgery)
Urological ( *minor* , eg, transurethral resection of the prostate)	Renal transplant	Pulmonary transplant
VATS ( *minor* )	Urological or gynecological ( *major* )	Liver transplant
		Total cystectomy
		Repair of perforated bowel

Adapted from Halvorsen et al.^3^VATS: video-assisted thoracic surgery.

Additionally, the urgency of the surgical intervention should always be considered. Emergency and urgent operations are associated with a higher incidence of CV complications. In situations where the prognosis of the underlying disease necessitating surgery demands an **emergency** intervention, the role of the cardiologist should be limited to suggesting surveillance measures (including the location for postoperative care) and interventions to reduce intra and postoperative risk. It is not recommended to order any additional tests that could delay the proposed surgery. For **urgent** procedures, there is sufficient time to optimize CV therapy or perform additional tests, such as transthoracic echocardiography (TTE), when indicated. However, functional tests for the assessment of myocardial ischemia should not be performed, as their results would not alter patient management since the proposed surgery cannot be postponed for coronary treatment. Furthermore, there are **time-sensitive procedures** , which are not urgencies, but whose delay could worsen the prognosis of the underlying disease. A common example is cancer surgery, whose delay can worsen the prognosis. In these cases, we recommend multidisciplinary discussion and the selection of the best individualized strategy.

#### 2.1.3. Functional Capacity Assessment

Patients with reduced functional capacity (less than four metabolic equivalents [METs] or inability to climb two flights of stairs) are more likely to develop perioperative complications.^4^Functional capacity can be objectively assessed via exercise electrocardiogram (ECG), which is not always feasible or desirable, or clinical history. A recent study demonstrated that patients who reported being able to climb two flights of stairs during the preoperative evaluation had a lower rate of postoperative CV events.^5^

Additionally, the estimate of functional capacity combined with the Revised Cardiac Risk Index (RCRI) showed greater accuracy in predicting postoperative events compared with the RCRI alone.^5^In addition to the greater likelihood of poor perioperative outcomes, patients with reduced functional capacity may have their symptoms underestimated due to their limitations. Therefore, this may be considered when deciding whether to order additional tests such as functional tests for myocardial ischemia.

Chart 1 presents recommendations for preoperative assessment of functional capacity.

**Chart 1:** Recommendations for preoperative assessment of functional capacity

Recommendation	Grade of recommendation	Level of evidence
The functional capacity of patients scheduled for intermediate- or high-risk surgery should be determined during medical history (based on the ability to climb two flights of stairs).	I	B

#### 2.1.4. Tools for Estimating Perioperative CV Risk

Subjective assessments of perioperative CV risk are valuable, but objective risk estimation allows for the rational use of additional risk stratification tools and perioperative CV care. Furthermore, the calculation of CV risk better supports multidisciplinary discussions aimed at minimizing the patient’s overall risk.

This Guideline does not advocate for the adoption of a specific algorithm for perioperative CV risk stratification but recommends calculating the risk using one of the indices available in the literature after excluding severe/unstable cardiac conditions ( Table 2 ).

Among the indices for estimating perioperative CV risk, the RCRI, or Lee score,^6^and the more recently published American University of Beirut-HAS2 (AUB-HAS2) index, stand out for their practicality and accuracy in distinguishing different risk classifications.^7^Both models integrate clinical characteristics and the type of proposed surgery, without different weightings among the variables for each index ( Tables 4 and 5 ). Increasing rates of CV complications were observed according to the number of variables in the pivotal cohorts of the RCRI and AUB-HAS2.

**Table 4:** Elements of the RCRI^6^and AUB-HAS2^7^

RCRI	AUB-HAS2
History of coronary artery disease *	History of heart disease **
History of heart failure	Symptoms of heart disease: angina or dyspnea
History of cerebrovascular disease	Age ≥ 75 years
Creatinine > 2.0 mg/dL	Hemoglocbin < 12 g/dL
Intraperitoneal, intrathoracic, or suprainguinal vascular surgery	Arterial vascular surgery
Diabetes on insulin	Emergency surgery

* In the RCRI, the criteria for coronary artery disease are a history of myocardial infarction, positive functional test, presence of angina, use of nitrate, or Q wave on the electrocardiogram.

** In the AUB-HAS2, the criteria for heart disease are a history of myocardial infarction, myocardial revascularization, heart failure, atrial fibrillation, or moderate-to-severe valvular heart disease on the echocardiogram. AUB-HAS2: American University of Beirut-HAS2; RCRI: Revised Cardiac Risk Index.

**Table 5:** Risk classification according to the number of risk variables in the derivation and validation cohorts of the RCRI^6^and AUB-HAS2^7^

RCRI	AUB-HAS2
None	None
One	One
Two	Two
Three to six	Three
	Four to six


RCRI: Revised Cardiac Risk Index; AUB-HAS2: American University of Beirut-HAS2.

In the Brazilian population, the superiority of one algorithm over the other has not yet been established. It is worth noting that although both indices estimate severe CV outcomes, the outcomes differ. The RCRI, already validated in a Brazilian population,^8^estimates the risk of myocardial infarction (MI), acute pulmonary edema, third-degree atrioventricular block, and cardiorespiratory arrest within 30 days after surgery.^6^The AUB-HAS2, on the other hand, has not yet been validated in a Brazilian population and estimates the risk of MI, stroke, and death, also within 30 days of surgery.^7^

In the older version of the RCRI used in the previous edition of this Guideline,^2^the risk of postoperative CV events was 0.4% for Class I (no risk variables), 0.9% for Class II (1 variable), 7% for Class III (2 variables), and 11% for Class IV (3 or more variables). Other algorithms that estimate the risk of perioperative complications also included numerical percentage values that represent the rates observed in reference studies. More recent studies, however, suggest that these risk estimates are outdated and are currently higher to the increased severity of patients undergoing surgery. As published in the latest European guideline on perioperative assessment,^3^there was a 4% risk for Class I, 6% for Class II, 10% for Class III, and 15% for Class IV.

Considering the significant variation in these figures by country and population, we opted for a semiquantitative classification, establishing low risk (Class I and II), intermediate risk (Class III), and high risk (Class IV) in preoperative risk assessment reports without specifying absolute values.

Among the limitations of these indices is the potential loss of accuracy in patients with reduced functional capacity. These patients have a worse perioperative prognosis^9^and may be asymptomatic simply because they do not reach the threshold to trigger symptoms. Therefore, even if a patient with reduced functional capacity receives a low or intermediate CV risk estimate from the indices, this evaluation can be complemented by tests for coronary artery disease (CAD) and HF before high-risk operations, especially when the patient has CV risk factors (see item 2.4, functional tests). Another issue is the loss of index accuracy for specific operations, such as the RCRI, which loses the ability to discriminate between risk classifications and underestimates events in patients undergoing vascular operations, particularly abdominal aortic aneurysm repair.^6 , 10 - 12^Specifically for vascular surgery, this Guideline recommends using the Vascular Study Group of New England Cardiac Risk Index (VSG-CRI) ( Tables 6 and 7 ).^11^Compared with the RCRI, the VSG-CRI showed better accuracy in predicting MI, clinically relevant arrhythmia, and HF in the postoperative period of vascular surgery.^11^The AUB-HAS2 also maintains good accuracy in distinguishing between four classes of progressive risk in the perioperative period of vascular surgery, with higher absolute rates of perioperative complications than those observed for all operations combined.^13 , 14^

**Table 6:** Risk factors and respective scores in the Vascular Study Group of New England Cardiac Risk Index (VSG-CRI)^11^

VSG-CRI risk factors		SCORE
**Age**		
	≥ 80 years	4
	70-79 years	3
	60-69 years	2
Coronary artery disease		2
Heart failure		2
Chronic obstructive pulmonary disease		2
Creatinine > 1.8 mg/dL		2
Smoking		1
Diabetes on insulin		1
Chronic therapy with beta-blockers		1
Previous myocardial revascularization		-1

**Table 7:** Risk classification according to the Vascular Study Group of New England Cardiac Risk Index (VSG-CRI)^11^

VSG-CRI score	Risk classification
0-3	Low
4	Low
5	Intermediate
6	Intermediate
7	High
≥ 8	High

Other risk indices validated for perioperative CV assessment include the American College of Physicians Risk Index^15 , 16^and the Multicenter Perioperative Evaluation Study (EMAPO) Index,^8^the latter developed and validated in Brazil. For estimating overall risk not solely related to CV morbidity and mortality outcomes, the American College of Surgeons (ACS) NSQIP® Surgical Risk Calculator (www.riskcalculator.facs.org) can be used.^17^This tool was developed from a database of over 1 million operations performed in the United States and considers, in addition to the specific type of surgery, 21 clinical variables, providing risk estimates for 8 different outcomes. The main limitation of this tool is that it is not quick or easily applicable, requiring a calculator and including some subjectively determined variables.

The choice of perioperative CV risk index should consider the evaluator’s experience and the vascular or nonvascular nature of the surgical procedure. It is worth noting that the risk of venous thromboembolism (VTE), which is highly prevalent and preventable in the perioperative period, is not covered by the indices discussed in this section and requires a dedicated approach.^2^

The flowchart for perioperative assessment is presented in Central Illustration.

#### 2.1.5. Frailty Assessment

Frailty syndrome is an age-related, multidimensional clinical entity defined as a reduction in physiological reserve across several physiological systems, leading to a state of increased vulnerability to stressors such as acute illnesses and surgery. Frailty is associated with an increased risk of mortality and several other adverse outcomes, such as hospitalization, reduced functional capacity, and poor quality of life.^18^Over the past decades, there has been growing global attention to the assessment of frailty in different clinical settings, reflecting the phenomenon of an aging population worldwide.^19 , 20^In Brazil, the prevalence of frailty is 9% among people aged ≥ 50 years living in the community and 16% among those aged ≥ 65 years. These rates are similar to those in developed countries and higher in health care settings such as clinics and hospitals.^21^Despite the lack of consensus on a gold standard method for defining frailty, the Physical Frailty Phenotype and the Frailty Index are considered the most valid measures for identifying this syndrome.^22^Although associated with multimorbidity, aging, and limitations in basic and instrumental activities of daily living, frailty syndrome is considered a distinct entity separate from these other factors.^22 - 24^The severity of frailty ranges from robust and prefrail to truly frail patients.

Although CVD can evidently cause frailty as a result of hospitalization for acute events, immobility, and physical limitation in individuals with established disease (eg, hospitalization for acute coronary syndrome, physical limitation due to HF symptoms),^25^there is biological plausibility for common underlying pathophysiological processes in both conditions, indicating a bidirectional relationship.^26^Frailty is associated with a series of physiological and functional changes, including chronic inflammation, endothelial dysfunction, autonomic dysfunction, activation of the renin-angiotensin-aldosterone system, and oxidative stress, which are risk factors for CVD.^27^Indeed, in a cohort of patients without CVD,^28^the presence of frailty was a risk factor for the incidence of CV events over a 6-year follow-up period. Other studies have also observed an independent association between frailty and adverse CV outcomes in several CV conditions.^29^

##### 2.1.5.1. Frailty Assessment Before Noncardiac Surgery

Despite the lack of consensus on a single instrument for screening frailty in older adults, both at the population level and in specific situations such as the perioperative assessment for noncardiac surgery, it is recommended that frailty be objectively assessed using previously validated instruments. Table 8 presents some examples of instruments that can be used to assess frailty. It is noteworthy that there are other instruments available beyond those mentioned in Table 8 . The recommendation to use instruments is based on the fact that the subjective assessment of physicians, especially those not specialized in geriatrics, can be influenced by confounding factors such as advanced age, the presence of multiple diseases, low weight, and the use of walking aids, which can lead to an erroneous assumption of frailty when it is not actually the case.^30 - 32^Frailty assessment with validated instruments also provides more detailed information about the patient’s status rather than simply categorizing them as frail or nonfrail, which can be simplistic and superficial.

**Table 8:** Instruments available for frailty assessment

Instrument	Type of assessment	Components	Definition of frailty	Link or app to access the instrument
Physical Frailty Phenotype^31^	Self-reported information and physical performance tests to assess 5 phenotypic criteria	Low activity Slowness Weight loss Weakness Exhaustion	Frail: ≥ 3 Prefrail: 1-2 Robust: 0	https://www.johnshopkitnssolutions.com/solution/frailty/
Clinical Frailty Scale (CFS)^32^	Overall health and functional capacity	9-Item scale (1 – very fit to 9 – terminally ill)	Frail: ≥5 Prefrail: 4 Robust: 1-3	https://www.acutefrailtynetwork.org.uk/Clinical-Frailty-Scale/Clinical-Frailty-Scale-App
Essential Frailty Toolset (EFT)^33^	Combination of physical and cognitive performance with complementary tests	Chair rise Cognition Serum albumin Hemoglobin	Frail: ≥3 Prefrail: 1-2 Robust: 0	Frailty Tool: App available for iOS and Android
Frailty Index of deficit accumulation^34^	Multidimensional due to the deficit-accumulation approach	Assessment of at least 40 items that should: – Be associated with health – Encompass multiple systems – Have a prevalence > 1% in the population of interest – Increase in prevalence with advancing age	The score ranges from 0 (no deficit present) to 1 (all deficits present), which indicates the proportion of changes found between the items evaluated In general, a score > 0.25 is used to define the presence of frailty	https://www.bidmc.org/research/research-by-department/medicine/gerontology/calculator
FRAIL Scale^35^	Concise, self-reported questionnaire including 5 health components	Fatigue Resistance Ambulation Illness Weight loss	Frail: ≥3 Prefrail: 1-2 Robust: 0	https://www.sciencedirect.com/science/article/pii/S1279770723014987?via=ihub-cesec100 https://www.ncbi.nlm.nih.gov/pmc/articles/PMC4515112/#APP1

Frailty can also be assessed and classified based on different health domains: physical, cognitive, psychosocial, and nutritional. Depending on the tool used to define frailty, one or more domains are assessed, allowing for the characterization of patients into distinct clinical phenotypes.^22^

##### 2.1.5.2. Impact of Frailty Assessment on Noncardiac Surgery

Frailty is a predictor of several adverse perioperative outcomes across multiple surgical specialties, including both elective and emergency procedures. Evidence indicates that frail patients are at higher risk for postoperative clinical complications such as infections, delirium, acute renal failure, cardiac arrhythmias, and MI, as well as issues related to wound healing, such as dehiscence and hernias. Consequently, frail patients tend to have prolonged lengths of hospital stay. These postoperative complications negatively impact the functional recovery and quality of life of frail patients undergoing surgery, often leading to a greater need for transfer to long-term care facilities and increased short- and long-term mortality compared with nonfrail patients.^33 - 43^Despite this, the impact of incorporating frailty assessment into the prognostic performance of traditional risk scores (RCRI, VSG-CRI, AUB-HAS 2, etc.) remains uncertain due to the limited number of studies on this topic.^44 , 45^The impact of this combination using other statistical methods still needs to be investigated to determine its significance and clinical utility.^46^

Despite existing limitations, perioperative frailty assessment is understood to (1) assist in the decision-making process by providing a more comprehensive assessment of the risks associated with the surgical procedure, thereby improving communication between the patient, family, and the several professionals involved in perioperative care (anesthesiologist, surgeon, clinician, cardiologist, geriatrician); and (2) identify a subgroup of patients at higher risk of complications, in whom early interventions can be implemented (eg, physical and nutritional rehabilitation; measures for prevention and early identification of delirium).

Chart 2 presents recommendations for perioperative frailty assessment in noncardiac surgery.

**Chart 2:** Recommendations for perioperative frailty assessment in noncardiac surgery

Recommendation	Grade of recommendation	Level of evidence
Frailty should be routinely assessed in elderly patients scheduled for intermediate- or high-risk surgery.	IIa	B
Frailty should be objectively measured using specific instruments.	IIa	C

### 2.2. Electrocardiography

Electrocardiography (ECG) can detect arrhythmias, conduction disorders, myocardial ischemia or prior AMI, ventricular overload, and changes due to electrolyte disturbances or effects of medications. Additionally, a baseline ECG is important for comparative evaluation in the perioperative period for patients at high risk of CV events.

However, routine application of a test with limited specificity can lead to false-positive results in asymptomatic patients. ECG changes often cause concern among the surgical and anesthetic teams and can sometimes lead to unnecessary cancellation of the operation.^47^It is estimated that approximately 50% of individuals over 40 years of age will present some ECG abnormality.^48^The presence of abnormalities tends to increase with age and the presence of comorbidities, but has a low predictive power for complications.^49 - 51^

In a retrospective study of over 23,000 patients, those with abnormal ECG findings had a greater incidence of CV death within 30 days compared with those with normal ECG results.^52^This finding was corroborated by two subsequent prospective studies which found similar results, with preoperative ECG abnormalities being predictors of perioperative CV events.^53 , 54^In another retrospective study, a corrected QT interval between 480 ms and 519 ms was an independent predictor of mortality after noncardiac surgery.^55^However, in the group of patients undergoing low-to-moderate risk surgery, the preoperative ECG provided limited prognostic information. Therefore, the prognostic interpretation of the ECG depends on the patient’s clinical history as well as their CV risk.

Thus, the main role of the preoperative ECG is to provide a baseline tracing for comparison in case of a suspected postoperative CV event. The indication for preoperative ECG should be judicious, considering the patient’s clinical history, type of surgery, and pre-existing conditions ( Figure 1 ).

**Figure 1:** Indications for additional preoperative cardiac tests. *Only indicated for patients scheduled for intermediate- or high-risk surgery. **No evaluation in the past year (or 6 months in cases of valvular heart disease). ACS: acute coronary syndromes; CVD: cardiovascular disease; HF: heart failure.

Chart 3 presents recommendations for performing ECG.

**Chart 3:** Recommendations for performing electrocardiography

Recommendation	Grade of recommendation	Level of evidence
History and/or abnormalities on physical examination suggestive of cardiovascular disease.	I	C
Patients undergoing surgery with intermediate or high intrinsic risk of cardiovascular complications.	I	C
Patients at intermediate or high risk for perioperative cardiovascular events as estimated by algorithms.	I	B
Diabetes.	I	C
Obesity.	IIa	C
Age > 40 years.	IIa	C

### 2.3. Echocardiography

Echocardiography is a low-cost, noninvasive diagnostic method with extensive application in various fields of cardiology. It provides a comprehensive CV morphofunctional assessment, which is crucial for preoperative diagnostic and therapeutic decisions. In terms of morphology and anatomy, basic data assessed in routine echocardiography include cavity size, ventricular mass, structural assessment of valves and major vessels, and systolic and diastolic function. Hemodynamic evaluation is derived from mathematical calculations using data obtained from Doppler ultrasound, and color flow mapping accurately assesses intracardiac and transvalvular flow dynamics. Morphofunctional cardiac changes can be closely correlated with an increased perioperative risk.

In addition to conventional TTE, other modalities can be performed. These include transesophageal echocardiography, a semi-invasive technique that allows detailed anatomical assessment, intracavitary thrombus investigation, and aortic evaluation, among others; myocardial strain imaging for a more detailed evaluation of ventricular contractile function; the use of contrast agents (microbubbles) for myocardial perfusion assessment; and three-dimensional echocardiography, which allows for a more accurate volumetric evaluation and determination of left ventricular ejection fraction (LVEF).

However, it is important to note that preoperative resting echocardiography should not be performed routinely in the setting of noncardiac surgery. It should only be requested when, after an initial clinical evaluation, there is suspicion of heart disease. This initial evaluation should involve thorough history-taking and physical examination, and possibly common clinical tests such as laboratory tests, ECG, and chest radiography.^56 , 57^

TTE is the primary diagnostic method in patients with suspected or known HF, and its multiple modalities help estimate surgical risk.^58 - 60^However, it should be used judiciously, as there is no evidence that TTE is associated with increased survival or shorter lengths of hospital stay. On the contrary, several studies suggest that more widespread use increases length of hospital stay without clinical benefit.^61^Finally, in patients with known or suspected valvular heart disease (VHD), prosthetic heart valves, and intracardiac devices, TTE or transesophageal echocardiography should be used to help determine perioperative risk, as well as to guide prophylaxis for infective endocarditis (IE) ( Figure 1 ).

Chart 4 presents recommendations for performing preoperative echocardiography.

**Chart 4:** Recommendations for performing preoperative echocardiography

Recommendation	Grade of recommendation	Level of evidence
Patients with known heart failure or symptoms suggestive of heart failure scheduled for intermediate- or high-risk surgery, without evaluation in the past year or with clinical worsening.	I	B
Patients with suspected or known moderate-to-significant valvular heart disease scheduled for intermediate- or high-risk surgery, without evaluation in the past 6 to 12 months or with clinical worsening.	I	C
Patients scheduled for liver transplantation.	I	B
Symptomatic patients with a prosthetic heart valve scheduled for intermediate- or high-risk surgery.	I	C
Patients with a prosthetic heart valve scheduled for intermediate- or high-risk surgery without evaluation in the past year.	IIa	C
Asymptomatic patients scheduled for high-risk surgery (see Table 3 ).	IIb	C
Routine use in asymptomatic patients without clinical suspicion of heart failure or moderate-to-severe valvular heart disease scheduled for intermediate- or low-risk surgery.	III	C

### 2.4. Noninvasive Tests for Myocardial Ischemia

#### 2.4.1. Exercise Electrocardiogram

Exercise ECG is a safe, useful, and effective tool in the investigation of myocardial ischemia,^62^with its primary mechanism being the provocation of a mismatch in blood supply and demand. Thus, it is reasonable to think that the detection of abnormalities on exercise ECG could be reproducible during the perioperative period and its varying levels of stress. However, there is no robust evidence that using this strategy results in reduced perioperative mortality.^63^

Considering that the goal of risk stratification is to reduce perioperative risk, it would not be logical to perform exercise ECG on individuals already stratified as low risk by the recommended algorithms. In populations with a low prevalence of CAD, exercise ECG would not add value to perioperative clinical stratification, given that the lower the prevalence of CAD, the lower the positive predictive value of the test. In fact, exercise ECG could even delay the surgery due to the need for other more specific tests to differentiate true from false-positive results.^64 , 65^

Even in high-risk individuals, such as in the preoperative period of vascular surgery, the positive predictive value, sensitivity, and specificity of exercise ECG are low (10%, 74%, and 69%, respectively), although the negative predictive value is high (98%).^66^Conversely, in a cohort study, preoperative exercise ECG for ischemia in high-risk patients with 3 or more clinical risk factors was associated with shorter lengths of hospital stay and lower hospital and 1-year mortality rates.^64^Therefore, among asymptomatic individuals with a higher prevalence of the disease, exercise ECG could be requested only when the result would influence the prognosis and, consequently, the preoperative approach, either to guide more intensive clinical therapy or a myocardial revascularization procedure. In this setting, low exercise tolerance (below 4 METs) and the onset of ischemic response at a low workload are associated with a higher number of perioperative CV events.^9^

Chart 5 presents recommendations for preoperative exercise ECG.

**Chart 5:** Recommendations for preoperative exercise ECG testing

Recommendation	Grade of recommendation	Level of evidence
A exercise ECG test should be requested in intermediate- and high-risk patients with reduced functional capacity scheduled for intermediate- or high-risk elective noncardiac surgery in whom functional testing could potentially alter management.	IIb	C
Patients scheduled for low-risk surgery.	III	C
Patients at low risk of complications scheduled for low- or intermediate-risk surgery.	III	C

#### 2.4.2. Myocardial Perfusion Imaging (MPI)

Despite greater availability and lower cost, exercise ECG has limitations, particularly in patients with a high pretest probability of CAD, in those with baseline ECG abnormalities, and in those unable to perform physical activity on a treadmill.^62^

Thus, the use of non-invasive functional imaging for myocardial ischaemia presents higher accuracy in assessing the risk of perioperative CV events. This approach can be useful for further risk stratification in patients with reduced functional capacity, established CAD, or a high pretest probability of CAD without a prior diagnosis of chronic coronary syndrome.

In the perioperative period, there is a strong correlation between the degree of CAD and the severity of perfusion abnormalities, meaning that functional imaging tests functional testing has a greater predictive capacity in patients with significant CAD.^67^A meta-analysis evaluating 1,179 patients undergoing vascular surgery revealed that large perfusion defects (> 20%) were associated with a higher rate of perioperative events, with an area under the curve of 0.78 (95%CI 0.65-0.89). However, it should be noted that even in a high-risk population (established vascular disease), only 23% of individuals presented with extensive ischemia.^68^

In patients with good functional capacity (≥ 4 METs), preoperative functional imaging tests in addition to the RCRI added only moderate value (AUC 0.77 vs AUC 0.70).^69^Another consideration when requesting functional imaging tests is that recent studies (outside the perioperative context) demonstrated the benefit of clinical treatment over invasive strategies even in patients with moderate-to-severe ischemia.^70^

Finally, despite its usefulness in the diagnosis of CAD and CV risk stratification, preoperative functional imaging tests should be requested only when necessary. This is because it may lead to a propensity for coronary revascularization before noncardiac surgery, which has been shown in randomized clinical trials to not alter long-term outcomes, and may even delay noncardiac surgery for the minimum time required for dual antiplatelet therapy (DAPT).^71^Even in patients with high ischemic burden on functional tests listed for kidney transplant, invasive approaches such as revascularization were not superior to optimized clinical treatment.^72^

#### 2.4.3. Dobutamine Stress Echocardiography

Dobutamine stress echocardiography (DSE) is accurate and safe in the identification of patients with CAD and plays an important role as a predictor of CV events.^73 , 74^Stress can be induced via physical exercise, on a treadmill or cycle ergometer (bicycle), or by using medications, specifically dobutamine and dipyridamole, combined with atropine if there are no contraindications. Dobutamine and exercise have similar diagnostic accuracy, which is superior to that of dipyridamole.^75^

Pharmacological stress echocardiography with dobutamine or dipyridamole is recognized for its significant diagnostic and prognostic value. Segmental contraction abnormalities involving a large extent of the left ventricle, appearing at early stages of drug infusion, not only confirm the diagnosis of CAD but also confer a high risk of short-term ischemic complications, significantly increasing perioperative CV risk. Conversely, the absence of segmental contraction abnormalities during stress is well correlated with the absence of severe coronary obstruction. Additionally, the appearance of such abnormalities in a small myocardial area at the late peak stress stage portends a better prognosis and lower risk of CV events. If DSE does not show residual ischemia in a patient with a prior infarction, the prognosis is good, and the likelihood of perioperative reinfarction, death, and acute pulmonary edema during noncardiac surgery is low.^66^The use of DSE in the assessment of perioperative risk is well documented, with a positive predictive value ranging from 25% to 55% and a negative predictive value ranging from 93% to 100% for CV events in patients undergoing noncardiac surgery.^66 , 76^The results are typically used to guide preoperative clinical management, especially the decision to perform coronary angiography with angioplasty or myocardial revascularization surgery before or after elective surgery. A meta-analysis of 15 studies comparing MPI and DSE in preoperative CV risk stratification demonstrated that the prognostic value of abnormalities in both imaging methods for perioperative ischemic events is similar.^77^

Finally, the use of microbubble contrast agents improves the visualization of left ventricular endocardial borders, increasing the sensitivity of DSE for detecting segmental contraction abnormalities. Furthermore, real-time assessment of myocardial perfusion may also be performed. Perfusion deficits detected in an ischemic wall are correlated with a worse prognosis, higher likelihood of short-term events and, consequently, increased perioperative CV risk.

#### 2.4.4. Summary of Recommendations for Noninvasive Functional Imaging for Myocardial Ischaemia ( Figure 1 )

Chart 6 presents recommendations for preoperative myocardial perfusion imaging or stress echocardiography.

**Chart 6:** Recommendations for preoperative myocardial perfusion imaging or stress echocardiography

Recommendation	Class of recommendation	Level of evidence
Intermediate- and high-risk patients with reduced functional capacity scheduled for intermediate- or high-risk elective noncardiac surgery in whom functional testing could potentially alter management.	IIa	B
Asymptomatic patients with reduced functional capacity and a previous diagnosis or high probability of coronary artery disease.	IIb	B
Routine in low-risk patients and/or patients scheduled for low-intrinsic-risk procedures.	III	C

### 2.5. Coronary Cineangiography

Invasive coronary assessment with coronary cineangiography is not routinely recommended before noncardiac surgery and should not replace noninvasive tests for myocardial ischemia, when indicated. Performing coronary cineangiography unnecessarily may delay surgical planning and has not shown evidence of increased patient survival or reduced risk of perioperative MI, even in patients undergoing procedures with a high risk of CV complications.^71 , 78^

The indications for invasive coronary assessment are similar to those outside the preoperative setting, such as patients with acute coronary syndrome or extensive ischemia on functional testing ( Figure 1 ).^79^A small, randomized study evaluated the effect of routine preoperative coronary cineangiography on carotid endarterectomy. Despite fewer ischemic events in patients undergoing preoperative angioplasty, there was no difference in mortality. Moreover, surgery was performed on average 4 days after the procedure, which is neither recommended nor safe.^80 , 81^

Time-sensitive procedures, such as cancer surgery, should not be postponed for invasive assessment in asymptomatic patients. This recommendation is based on studies that failed to show any reduction in postoperative CV complications in asymptomatic patients undergoing preoperative coronary revascularization.^82^

Chart 7 presents recommendations for preoperative coronary cineangiography.

**Chart 7:** Recommendations for preoperative coronary cineangiography

Recommendation	Class of recommendation	Level of evidence
Patients with high-risk acute coronary syndromes.	I	A
Patients with extensive ischemia on functional testing.	I	B
Stable patients undergoing low-risk surgery.	III	C

### 2.6. Coronary Computed Tomography Angiography

Coronary computed tomography angiography (CCTA) has high sensitivity for anatomical detection of coronary stenoses, including multivessel and left main CAD.^83^However, the benefits of CCTA before noncardiac surgery remain uncertain.

The benefit of coronary anatomy assessment via CCTA was investigated by Li et al., who assessed 841 elderly patients undergoing high-risk noncardiac surgery without known or suspected CAD. Single-, two-, and three-vessel disease was found in 103 (12.2%), 45 (5.4%) and 16 (1.9%) patients, respectively. An Agatston score above 195 was independently associated with a higher risk of CAD. Significant CAD was found in 19.5% of patients. In multivariate analysis, the degree of stenosis was as an independent factor for the cancellation of scheduled surgery. The authors considered CCTA useful for ruling out or confirming significant CAD.^84^

The use of CCTA in addition to the RCRI was investigated by Ahn et al.,^85^who evaluated retrospective studies including patients undergoing intermediate-risk procedures. The presence of lesions > 50% increased the incidence of major adverse cardiac events (MACE), reaching up to 29.7% in multi-vessel CAD vs 4.3% in its absence. The authors concluded that CCTA showed additive value to RCRI in risk reclassification. In a prospective study of 955 patients, Sheth et al. demonstrated that preoperative CCTA could improve estimation of risk for patients who will experience perioperative CV death or MI. However, risk reclassification using CCTA compared with the RCRI occurred in 22% of patients and could inappropriately overestimate the risk of complications by up to 5 times.^86^

The extent and severity of CAD on CCTA concerning MACE incidence was evaluated in a meta-analysis of 11 studies.^87^It was observed that the severity and extent of CAD were associated with increased MACE risk (no CAD 2%, nonobstructive 4.1%, obstructive single-vessel 7.1%, obstructive multivessel 23.1%). Multi-vessel CAD presented the highest risk (odds ratio [OR] 8.9). Increased calcium scores were also associated with a higher perioperative MACE risk (calcium score ≥ 100, OR 5.1, ≥ 1,000 OR 10.4, both p < 0.001).

The PANDA trial directly compared the prognostic accuracy of CCTA vs DSE.^88^The study included 215 patients with more than 1 risk factor for perioperative CV events who underwent both CCTA and DSE. DSE had an OR of 6.1 for CV events; the presence of significant CAD on CCTA had an OR of 18.8; and an elevated calcium score had an OR of 4.2. The authors concluded that CCTA might have higher prognostic value than DSE before noncardiac surgery, but the sample size of the study was too small for a definitive conclusion.

Due to the lack of more studies and evidence demonstrating event reduction with the use of CCTA, and primarily due to the concern of generating excessive "preventive" angioplasties, CCTA is not yet routinely recommended for risk stratification before noncardiac surgery.

## 3. Specific Diseases and Procedures in the Perioperative Period

### 3.1. Heart Failure

Heart failure (HF) affects approximately 1% to 2% of the general population in developed countries, approximately 5.7 million patients in the United States, and over 10% of the population older than 70 years.^89 , 90^Circulatory system diseases are the leading cause of death in Brazil, accounting for approximately 29% of deaths in the country. Ischemic heart diseases and HF are responsible for approximately 39% of deaths due to circulatory system diseases.^91^

HF is a well-known risk factor for perioperative CV events. Data from a large registry of noncardiac operations, which included over 150,000 procedures, revealed that the presence of HF was associated with a 63% increase in the risk of perioperative mortality and a 51% increase in the risk of 30-day all-cause readmission compared with the group with CAD without HF.^92^

A recent analysis with data from a U.S. database including 21,560,996 noncardiac surgical procedures from 2012 to 2014 revealed the presence of perioperative HF in 4.9% of cases, which was associated with a higher in-hospital mortality rate (4.8% vs. 0.8%), conferring a 2.2 times increased risk of perioperative mortality. The risk was higher among patients with acute HF than those with chronic HF, and the risk was greater in patients with decompensated chronic HF compared with those with chronic HF alone (7.8% vs. 3.8%, p < 0.001).^93^

In another retrospective cohort study involving 609,735 patients undergoing noncardiac surgery, the 90-day mortality rate was higher among patients with symptomatic HF (10.1%), followed by patients with asymptomatic HF (4.9%). In patients without HF, the mortality rate was 1.2%. Thus, the presence of HF, whether symptomatic or not, increases the risk of early death.^94^

Reduced LVEF is considered a strong predictor of CV events in patients undergoing vascular surgery. However, most studies analyze LVEF by dichotomizing it as greater or less than 40%. A study involving 174 patients with HF revealed that only severely reduced LVEF (< 30%) was an independent predictor of mortality. The presence of moderately (30-40%) or mildly (40-50%) reduced LVEF, or HF with preserved LVEF (> 50%), were not independent predictors of 30-day mortality.^95^Despite the predictive power of LVEF for CV events, routine echocardiography for all patients scheduled for noncardiac surgery is not indicated. A Canadian cohort study involving more than 250,000 patients (15% with preoperative echocardiography, n = 40,084) revealed that preoperative echocardiography is not associated with improved survival or reduced length of hospital stay after major noncardiac surgery.^61^

Preoperative elevation of natriuretic peptide levels is associated with a worse prognosis in the perioperative period, as it is associated with worsening ventricular function and a higher rate of CV events.^96 , 97^Measuring these biomarkers can assist in the risk stratification of patients with HF.

Perioperative clinical management of patients with HF should involve special attention to volemia. Both hypovolemia, which can intensify hypotension, and hypervolemia, which can cause pulmonary edema and systemic venous congestion, should be avoided. The Austrian IMPROVE study aims to determine if the use of an inodilator, levosimendan, may benefit postoperative outcomes by evaluating the impact of the medication on natriuretic peptide levels. Its results may provide a new perioperative management approach for patients with HF undergoing noncardiac surgery.^98^

The flowchart for preoperative evaluation of patients with known or suspected HF in is shown in Figure 2 .

**Figure 2:** Preoperative approach for patients with known or suspected heart failure. HF: heart failure; NYHA: New York Heart Association. #In patients with an echocardiogram from the past year and without new symptoms, it is not necessary to repeat the echocardiogram. *Optimized treatment of HF according to current guidelines (maximum tolerated dose of angiotensin-converting enzyme inhibitors/angiotensin receptor blockers/sacubitril/valsartan, beta-blockers, spironolactone, and sodium-glucose cotransporter type 2 [SGLT2] inhibitors for HF with reduced ejection fraction and adequate control of blood volume and blood pressure, spironolactone and SGLT2 inhibitors for patients with HF and preserved ejection fraction), evaluation and correction of myocardial ischemia, and correction of valvular heart disease in significant cases.

Chart 8 presents recommendations for the perioperative management of patients with HF.

**Chart 8:** Recommendations for the perioperative management of patients with heart failure

Recommendation	Grade of recommendation	Level of evidence
Elective surgery in patients with decompensated heart failure (NYHA class III/IV) should be postponed until the patient’s clinical condition has stabilized.	I	C
Elective surgery in patients with newly diagnosed heart failure, whose treatment has not yet been optimized, should be postponed for at least 3 months to allow adequate adjustment of medication doses.	I	C
All chronic medications should be maintained in the perioperative period and reintroduced as soon as possible postoperatively. If oral administration is not possible, consider the placement of a nasoenteric tube or intravenous administration.	I	C
The use of beta-blockers should be maintained in the perioperative period; however, initiating high doses in patients not previously on beta-blockers is not recommended unless there is sufficient time for dose adjustment before surgery.	I	C

NYHA: New York Heart Association.

### 3.2. Arterial Hypertension

Systemic hypertension is a highly prevalent clinical condition, both in the general population and in patients undergoing surgery, being responsible for approximately 50% of deaths from CAD and stroke.^99^Although the importance of strict blood pressure (BP) control in the prevention of long-term CV events is well established, its perioperative management remains controversial.

#### 3.2.1. Preoperative BP Management

A previous diagnosis of hypertension is an independent predictor of mortality in patients undergoing noncardiac surgery.^100^However, there is no solid evidence linking BP measurement at the time of hospital admission with perioperative CV complications.^101^This suggests that the perioperative risk attributed to hypertension is primarily due to its impact on target organ damage.^102^

Regarding hypotension, the literature provides more concrete data. A prospective cohort study involving 251,567 patients demonstrated that systolic BP levels < 119 mm Hg and diastolic levels < 63 mm Hg are associated with increased 30-day postoperative mortality.^103^Therefore, BP management should also consider this probable J-curve phenomenon, in which low BP levels also represent a risk.

Regarding hypertension, it is recommended that high-risk elective operations be postponed only if systolic BP is ≥ 180 mm Hg and/or diastolic BP is ≥ 110 mm Hg. It is important to note that this threshold is based on older observational data^104^and was defined before the current understanding of the risks associated with perioperative hypotension. Thus, the defined BP target should be maintained, but care must be taken to avoid hypotension.

Finally, patients with suspected secondary hypertension should be investigated before surgery. Although there is no robust evidence of increased perioperative risk in patients with secondary hypertension, those with undiagnosed pheochromocytoma are known to have very high surgical mortality rates.^105^

#### 3.2.2. Intraoperative BP Management

When the introduction of antihypertensives is required intraoperatively, the medication should ideally be easily titratable, have a rapid onset of action, few side effects, and low cost. Several classes are available, including beta-blockers (esmolol and labetalol), calcium channel blockers (nicardipine), and nitrates (sodium nitroprusside and nitroglycerin).

Hypertensive patients are also more susceptible to hypotension, often due to intravascular volume depletion.^106^Excessive concern about strict BP control in the preoperative period can lead to hypotension both during and after surgery, which is particularly associated with cardiac, renal, and cerebral injury, as well as increased mortality.^107^

In this setting, the INPRESS study (Effect of Individualized vs Standard Blood Pressure Management Strategies on Postoperative Organ Dysfunction Among High-Risk Patients Undergoing Major Surgery) compared an individualized strategy aimed at achieving a systolic BP within 10% of the reference value vs. a standard strategy where patients received vasoconstrictors only if systolic BP was < 80 mm Hg or 40% of the reference value. The individualized strategy reduced the incidence of systemic inflammatory response syndrome and organ dysfunction 7 days after surgery.^108^A retrospective analysis involving patients undergoing elective noncardiac surgery underscored the deleterious effects of perioperative hypotension.^109^

#### 3.2.3. Postoperative BP Management

A previous diagnosis of hypertension is the main risk factor for postoperative hypertension. However, other factors such as pain, hypercapnia, and agitation after anesthesia also contribute. Intravenous antihypertensive therapy should be considered in patients with sustained systolic BP ≥ 180 mm Hg and/or diastolic BP ≥ 110 mm Hg, as these levels increase the risk of bleeding, especially in cardiac, vascular, and endoscopic procedures, such as transurethral resection of the prostate. Hypotension should also be properly treated and prevented by maintaining an adequate volume status.

Chart 9 shows recommendations for perioperative BP management.

**Chart 9:** Recommendations for perioperative blood pressure management

Recommendation	Grade of recommendation	Level of evidence
Elective high-risk surgery should be postponed if systolic blood pressure is ≥ 180 mm Hg and/or diastolic blood pressure is ≥ 110 mm Hg.	I	C
Optimization of volemia (avoid dehydration) should be conducted throughout the perioperative period.	I	C
Episodes of hypotension should be avoided throughout the entire perioperative period.	I	B
Patients with suspected secondary hypertension should be investigated before surgery, except in emergency/urgent cases.	I	C
Antihypertensive therapy, preferably the one the patient used before surgery, should be restarted postoperatively as soon as possible.	I	C

#### 3.2.4. Perioperative Management of Antihypertensives

##### 3.2.4.1. Renin-Angiotensin-Aldosterone System Inhibitors

The use of renin-angiotensin-aldosterone system (RAAS) inhibitors in the perioperative period is highly controversial due to conflicting study results. Large retrospective studies comparing the discontinuation vs. continuation of angiotensin-converting enzyme inhibitors (ACEIs) and angiotensin II receptor blockers (ARBs) perioperatively have not shown differences between groups in terms of hypotension incidence, need for fluid resuscitation, and 30-day mortality.^110^Furthermore, discontinuing these medications on the day of surgery did not significantly increase the incidence of preoperative hypertension or result in higher surgery cancellation rates.^111^

However, Roshanov et al. observed a decrease in the intraoperative risk of hypotension and a reduction in the composite outcome of death, MI, and stroke with the discontinuation of these medications.^112^Despite the potential for increased hypotension with the maintenance of ACEIs/ARBs, there was no observed increase in acute kidney injury.^113^It is also noteworthy that if ARBs were discontinued and not reintroduced within 2 days after surgery, mortality rates increased.^114^

Thus, current evidence does not provide a definitive answer on whether to maintain or discontinue ACEIs/ARBs in the perioperative period, requiring individualized decision-making. The ongoing randomized trial STOP-or-NOT may provide additional information on this topic.^115^For this, factors such as the type of surgery, patient BP variability, and bleeding risk should be considered. Importantly, if these medications are discontinued, they should ideally be reintroduced postoperatively as soon as clinically feasible.

##### 3.2.4.2. Calcium Channel Blockers

In patients undergoing noncardiac surgery, no significant differences in BP levels were observed with the use of calcium channel blockers compared with other antihypertensive medications, and their use was not associated with increased occurrence of adverse events.^116^Therefore, while the formal recommendation is to continue chronic therapy with calcium channel blockers perioperatively, this decision should be individualized, considering the risks of hypotension and the lack of deleterious effects from their discontinuation.

##### 3.2.4.3. Diuretics

There is no robust evidence regarding the use of diuretics in the perioperative period. Their maintenance, including administration on the day of surgery, has been shown to be safe and not associated with increased risk of hypotension, need for fluid resuscitation, or use of vasopressor agents, nor with increased risk of postoperative CV events.^117^

##### 3.2.4.4. Central Sympatholytic Drugs

Early studies on the use of clonidine in the perioperative period of noncardiac surgery showed a reduction in myocardial ischemia.^118^However, larger, more recent studies have failed to confirm this association, instead linking its use to increased incidence of nonfatal cardiac arrest, particularly due to asystole or pulseless electrical activity. The use of clonidine is also associated with a significant increase in bradycardia and hypotension, with hypotension being an independent predictor of MI.^119^Thus, clonidine may be used in cases of uncontrolled hypertension with insufficient time for effective management, but it should not be introduced to reduce CV events. Discontinuation in chronic users is not recommended due to the potential for rebound hypertension.

#### 3.2.5. Final Considerations

The perioperative management of hypertension is highly challenging. While it is crucial to avoid extremely high BP levels (systolic BP > 180 mm Hg and diastolic BP > 110 mm Hg), it is equally important to prevent hypotension, which has been increasingly recognized in recent studies as a predictor of poor prognosis and increased risk of mortality. Therefore, aside from beta-blockers and central sympatholytic drugs, whose discontinuation may be harmful, the perioperative management of antihypertensives should be individualized to avoid significant BP increase, variability, and particularly hypotension. Perioperative clinical evaluation should include BP monitoring and vigilance for symptoms of hypotension such as dizziness, fainting, drowsiness, signs of low cardiac output, renal function deterioration, low urine output, and delirium.

Chart 10 shows recommendations for the perioperative management of antihypertensives.

**Chart 10:** Recommendations for the perioperative management of antihypertensives

Recommendation	Grade of recommendation	Level of evidence
Chronic therapy with renin-angiotensin-aldosterone system antagonists may be maintained in the perioperative period; discontinuation is allowed in selected cases.	I	C
Chronic therapy with calcium channel blockers may be maintained in the perioperative period; discontinuation is allowed in selected cases.	I	C
Chronic therapy with diuretics may be maintained in the perioperative period; discontinuation is allowed in selected cases.	I	C
Chronic therapy with clonidine should be maintained in the perioperative period.	I	B

### 3.3. Patient Management in Low-Risk Procedures

In this Guideline, "low-risk procedures" refers exclusively to the risk of bleeding and CV complications. We are aware that there are low risk surgical procedures in all medical specialties, but specific surgical considerations are beyond the scope of this document.

#### 3.3.1. Dental Procedures

Maintaining adequate oral health is crucial for reducing the risk of systemic complications, especially in patients with CVD, leading to better glycemic control, reduced risk of bacteremia, and possibly better BP control.^120 - 122^Oral diseases, such as periodontal diseases and endodontic infections, may pose a postoperative risk of bacteremia and sepsis in patients requiring intubation and in transplant recipients^123^and should be treated before surgical procedures.^124^

Mouth rinsing with 15 mL of 0.2% chlorhexidine (most effective) or 5% povidone-iodine for 30 to 60 seconds is recommended before dental procedures and before intermediate- and high-risk procedures to reduce the risk of sepsis.^125 , 126^

##### 3.3.1.1. Antithrombotic Agents

When patients on antithrombotic therapy, including anticoagulants and antiplatelet medications, undergo dental surgical treatment, a decision must be made regarding the continuation of anticoagulation therapy, balancing the risk of hemorrhagic complications against the risk of embolic complications. Decades of studies involving thousands of patients on anticoagulation therapy undergoing dental procedures have shown that hemorrhagic complications requiring more than local hemostatic measures are rare and never fatal. However, some embolic complications have been fatal or debilitating in patients in whom anticoagulation therapy was interrupted due to a dental procedure.^127^

Most dental procedures have a low risk of bleeding, with a bleeding rate of up to 3.63%.^128^There is strong evidence for older medications (e.g., warfarin, antiplatelet medications) and limited evidence for newer oral anticoagulants that, for most patients, the occurrence of significant uncontrollable bleeding is very low. For these reasons, this Guideline does not recommend altering or interrupting anticoagulation or antiplatelet therapy before dental procedures.^129 , 130^

In general, dental procedures can be safely performed in patients on warfarin with an international normalized ratio (INR) < 3.5, provided local measures to reduce bleeding are employed. For patients with an INR above this range or those expected to have a higher risk of bleeding, the best strategy should be discussed between the dentist and the physician who prescribed anticoagulant therapy.^131^

Bleeding management in dental practice can include lysine analogs (tranexamic acid 250mg, 15 to 25 mg/kg, ie, 2 tablets of 250 mg, 2-3 times a day) and local application of hemostatic sponges and cyanoacrylates, among other methods.

##### 3.3.1.2. Cardiac Implantable Electronic Devices

In addition to the risk of infection, the functioning of cardiac implantable electronic devices (CIEDs) may be impaired by the action of dental equipment during some procedures, particularly battery-operated composite curing light, ultrasonic scalers, and cleaning systems, when the distance between them and the CIED is less than 23 cm.^132^In these cases, alternative dental equipment should be used.

Conversely, other dental electronic devices such as electric toothbrushes, electric scalpels, electric pulp testers, high- and low-speed handpieces, apex locators, and amalgamators have not shown interference.^132 , 133^

##### 3.3.1.3. Local Anesthetics

Excessive use of local anesthetics combined with vasoconstrictors can increase heart rate (HR), BP, and subsequent myocardial oxygen demand. However, recent studies confirm that the use of 1-4 anesthetic cartridges with vasoconstrictor (lidocaine with epinephrine 1:800,00, 1:100,000, and 1:200,000) is relatively safe for patients with controlled CVD and hypertension.^134 , 135^

Chart 11 presents a summary of recommendations for dental procedures.

**Chart 11:** Summary of recommendations for dental procedures

Recommendation	Grade of recommendation	Level of evidence
Dental evaluation and treatment are important in the perioperative period.	IIa	B
Use of antimicrobial mouthwash before and after dental procedures and before intermediate- and high-risk noncardiac surgery.	IIa	B
The use of 1-4 anesthetic cartridges with vasoconstrictor is safe for dental treatment in patients with heart disease.	IIa	C
Antiplatelet and anticoagulant therapy should be maintained during most dental treatments, including extraction of up to 2 teeth, restorations, prosthetics, endodontics, cleaning, and implants.	IIa	B

#### 3.3.2. Dermatological Surgery

Dermatological surgical procedures have a low risk of both CV events and bleeding. Data from the literature suggest that approximately 50% of patients undergoing dermatological surgery are on antiplatelet or anticoagulant therapy.^136 , 137^In these cases, the surgical team and anesthesiologist should be informed about current medications and necessary precautions, including more careful and prolonged hemostasis, as the risk associated with discontinuing antithrombotic therapy usually outweighs the risk of bleeding inherent to the procedure.

In patients on acetylsalicylic acid (ASA) for secondary prevention of CV events, discontinuation is not recommended before any dermatological surgical procedure.^138 , 139^

In patients on clopidogrel monotherapy, the risk of bleeding during surgery is increased, and some studies recommend discontinuing the medication at least 6 days before the procedure.^140 , 141^However, no studies have demonstrated the safety of discontinuation in relation to CV events. Although there is a tendency to extrapolate evidence from ASA monotherapy to clopidogrel, there is insufficient evidence to support maintenance or discontinuation. Since bleeding in dermatological surgery is typically minimal and controllable, we recommend maintaining clopidogrel.

In patients on DAPT due to a coronary stent who are out of the critical thrombosis period, the recommendation is to discontinue the second antiplatelet medication,^142 , 143^respecting the intervals described in this Guideline (see section on antiplatelet medications, item 4.1.3).^144^

In patients on warfarin, it is recommended not to discontinue the medication, adjusting the INR to ≤ 3.5 to minimize the risk of bleeding. However, some studies have not shown a correlation between INR level and increased risk of bleeding in patients on warfarin.^142 , 145 - 148^

In patients on direct oral anticoagulants (DOACs), despite limited evidence, it is recommended to maintain these medications during most dermatological procedures^142 , 149 , 150^and schedule the surgery a few hours before the next dose to avoid peak serum concentration.

Chart 12 shows recommendations for patients scheduled for dermatological surgery.

**Chart 12:** Recommendations for patients scheduled for dermatological surgery

Recommendation	Grade of recommendation	Level of evidence
Acetylsalicylic acid (ASA) should be maintained for secondary prevention of cardiovascular events in patients undergoing any dermatological surgical intervention.	I	B
In patients on dual antiplatelet therapy due to a stent who are out of the critical thrombosis period, ASA should be maintained, and the second antiplatelet medication should be discontinued.	I	C
Clopidogrel (monotherapy) may be maintained for secondary prevention of cardiovascular events in patients undergoing dermatological surgical interventions.	IIa	C
Warfarin should be maintained in patients undergoing dermatological surgery, with an INR ≤ 3.5.	IIa	C
DOACs should be maintained in patients undergoing dermatological surgery, and the surgery should be scheduled a few hours before the next dose.	IIa	C

#### 3.3.3. Endoscopic Procedures

Endoscopic procedures are considered low risk for the occurrence of CV events.^151^Cancelling endoscopic procedures for CV intervention is usually unnecessary, except for severe CV conditions, as outlined in the section on perioperative assessment algorithms of this Guideline (item 2.1.1). Additionally, most CV medications do not need to be discontinued and can be taken with a minimal amount of water. The most crucial decision is whether to maintain or discontinue antithrombotic medications in patients on antithrombotic therapy, as their maintenance during an endoscopic procedure may lead to bleeding, whereas their interruption poses a risk of thromboembolic events. The risk of bleeding in endoscopic procedures is variable, which should always be considered when determining the best strategy to be used. The risk varies according to the type of procedure and is particularly related to the presence of therapeutic interventions and biopsies. Table 9 describes the risk of bleeding associated with common endoscopic procedures in clinical practice.^152^The risk of thromboembolic events from discontinuing antithrombotic therapy varies according to the therapy’s indication and the patient’s condition.

**Table 9:** Risk of bleeding according to endoscopic procedure

High-risk procedures	Low-risk procedures
Polypectomy	Diagnostic (EGD, colonoscopy, flexible sigmoidoscopy), including mucosal biopsy
Biliary or pancreatic sphincterotomy	ERCP with stent placement or papillary balloon dilation without sphincterotomy
Therapeutic balloon-assisted enteroscopy	Push enteroscopy and diagnostic balloon-assisted enteroscopy
Gastrostomy or percutaneous endoscopic jejunostomy	Capsule endoscopy
Endoscopic ultrasound with fine-needle biopsy	Endoscopic ultrasound without fine-needle biopsy
Cystogastrostomy	Enteral stent deployment
Esophageal dilation	Barrett’s ablation
Mucosectomy and submucosal dissection	Argon plasma coagulation
Tumor ablation	

Adapted from Acosta et al.^152^EGD: esophagogastroduodenoscopy; ERCP: endoscopic retrograde cholangiopancreatography.

##### 3.3.3.1. Management of Antiplatelet Therapy in Endoscopic Procedures

In endoscopic procedures with a low risk of bleeding, antiplatelet therapy can be maintained, whether as monotherapy (regardless of the medication) or DAPT.^152 - 157^In procedures with a high risk of bleeding, several considerations should be made. Patients on DAPT due to recent stent placement or acute coronary syndrome (see itens 4.1 and 4.2) are at a higher risk of suffering CV events from discontinuation of antiplatelet therapy.^144^Thus, elective endoscopic procedures with a high risk of bleeding should be postponed whenever possible until this high-risk period has passed. However, for procedures that must be performed during this period, the most accepted strategy is maintaining ASA and discontinuing the second antiplatelet medication,^155 , 158^although evidence for this strategy is limited. ASA monotherapy for secondary prevention of CV events may be maintained in the perioperative period of endoscopic procedures, including high-risk ones, as most evidence shows a low risk of significant bleeding in these circumstances.^156 , 157 , 159 - 169^Some studies have shown increased bleeding in procedures such as endoscopic submucosal dissection in patients with gastric neoplasia^170^and mucosectomy for colonic tumors larger than 20 mm.^171^Such cases should be assessed individually, considering the risk of thrombotic events from ASA discontinuation.^155^The maintenance of clopidogrel monotherapy during percutaneous endoscopic gastrostomy may be considered as there is some evidence supporting its use in this setting.^160^Conversely, evidence on the use of prasugrel and ticagrelor during endoscopic procedures with a high risk of bleeding is scarce. If antiplatelet therapy is to be discontinued, the interval between discontinuation and the endoscopic procedure should follow the recommendations in the section on antiplatelet management of this Guideline.^144^Antiplatelet therapy can be resumed after the procedure once hemostasis has been achieved, and a loading dose may be considered in patients at high risk of CV events.^158^

##### 3.3.3.2. Management of Anticoagulants in Endoscopic Procedures

Anticoagulation therapy with warfarin may be maintained during endoscopic procedures with a low risk of bleeding^152 , 153 , 155 - 157 , 161 , 169^but should be discontinued in high-risk ones.^156 , 162 , 168^There is no current evidence on the use of DOACs in this setting. It is suggested to maintain DOACs during procedures with a low risk of bleeding but discontinue them in high-risk ones.^152 , 156 , 157 , 169^The intervals between discontinuation and resumption of DOACs and warfarin (including bridging therapy in high-thromboembolic-risk patients) should follow the recommendations in the section on perioperative anticoagulation management of this Guideline (item 4.3). Regarding the use of DOACs, if possible, the endoscopic procedure should be performed before the next dose to avoid peak concentration, which occurs in the first 2 hours after administration.^157 , 169^

Chart 13 presents recommendations for patients scheduled for endoscopic procedures.

**Chart 13:** Recommendations for patients scheduled for endoscopic procedures

Recommendation	Level of Recommendation	Level of evidence
Antiplatelet (monotherapy or dual antiplatelet therapy [DAPT]) and anticoagulation therapy with warfarin should be maintained during endoscopic procedures with a low risk of bleeding.	I	B
Acetylsalicylic acid monotherapy for secondary prevention of cardiovascular events should be maintained in the perioperative period of endoscopic procedures, including most high-bleeding-risk procedures.	I	B
Anticoagulation therapy with warfarin or direct oral anticoagulants should be discontinued for endoscopic procedures with a high risk of bleeding.	I	B
Ideally, patients on DAPT after coronary angioplasty should not undergo endoscopic procedures with a high risk of bleeding during the duration of treatment.	I	B
In patients who need to undergo endoscopic procedures with a high risk of bleeding before the anticipated end of DAPT after coronary angioplasty, ASA should be maintained, and the second antiplatelet medication should be discontinued.	IIa	C
Anticoagulation therapy with DOACs may be maintained for endoscopic procedures with a low risk of bleeding.	IIa	C

#### 3.3.4. Ophthalmic Procedures

Ophthalmic surgery is relatively common among the older population. CV comorbidities requiring antithrombotic therapy, and its perioperative management are topics of intense debate among ophthalmologists and cardiologists. In Brazil, the fear of hemorrhagic events, including periorbital hematomas, leads to the indiscriminate discontinuation of ASA and warfarin in 82.7% of patients undergoing glaucoma surgery.^172^However, the available evidence, although limited, suggests that this fear is not justified. The rate of bleeding events reported in observational studies is low and without major consequences, particularly in cataract surgery using conventional anesthesia techniques.^173 - 177^Certain types of ophthalmic surgery, however, have a higher risk of bleeding events, such as trabeculectomy for glaucoma^178 , 179^and vitrectomy for retinal diseases.^180 , 181^Nonetheless, the available evidence does not demonstrate an increased risk of significant hemorrhagic events in these procedures with the use of ASA.^179 , 182 - 184^In these cases, management should be individualized, but it is generally recommended to maintain ASA perioperatively.^184 , 185^Patients on DAPT due to recent stent placement or acute coronary syndrome (see itens 4.1 and 4.2) are at higher risk of CV events with the interruption of antiplatelet therapy. Thus, ophthalmic surgical procedures, whenever possible, should be postponed until this high-risk period has passed. For procedures that need to be performed during this period, the approach depends on the risk of hemorrhagic events associated with the intervention. For low-risk interventions (intravitreal injections, cataract surgery, and peribulbar anesthesia), ASA and P2Y12 receptor inhibitors should be maintained. However, for high-risk interventions, such as vitrectomy and trabeculectomy, the most accepted recommendation is to maintain ASA and suspend the second antiplatelet medication, following the intervals described in the specific section of this Guideline (item 4.2),^144^although evidence for this strategy is limited. Similar to patients on ASA monotherapy, published evidence supports maintaining clopidogrel monotherapy in the perioperative period of cataract surgery.^176 , 177^Evidence for glaucoma and retinal surgery is even scarcer, thus it is recommended to discontinue clopidogrel 5 days before the procedure.

In patients on warfarin, the available evidence supports its maintenance during procedures with a low risk of hemorrhagic events, such as cataract surgery, provided the INR is within the therapeutic range.^173 , 174 , 184 , 186 , 187^A meta-analysis of observational studies with patients undergoing cataract surgery while on warfarin found a bleeding incidence of approximately 10%. The bleedings were mostly self-limiting and subconjunctival, and no patients experienced compromised visual acuity related to a bleeding event.^175^For glaucoma and retinal surgery, warfarin should be discontinued, and perioperative management should follow the strategy described in the section on perioperative anticoagulation management of this Guideline (item 4.3), according to the individual thrombotic risk of each patient. To date, evidence on the risk of bleeding in patients on DOACs undergoing ophthalmic surgery has only been well established for cataract surgery. The risk of bleeding associated with warfarin in cataract surgery has been well studied, and most guidelines recommend maintaining warfarin if the INR is within the therapeutic range. DOACs present a lower risk of bleeding than warfarin.^188^A systematic review and meta-analysis revealed that patients on DOACs have a 22% relative risk reduction in spontaneous intraocular bleeding compared with warfarin.^189^Two small studies compared the discontinuation and maintenance of DOACs during cataract surgery and found no increase in complications when maintaining DOAC therapy.^186 , 190^Therefore, most major centers tend to maintain the use of DOACs during cataract surgery, primarily due to the absence of evidence suggesting otherwise.^184 , 187^In high-risk procedures, there is no evidence regarding the safety of DOAC use. They are typically interrupted 24 hours before surgery.

Recommendations for the management of patients with coronary stents and prosthetic heart valves should be individualized, considering the risk of both thrombotic and hemorrhagic events. For patients recommended to continue anticoagulant and/or antiplatelet therapy, the surgeon should be informed of the need to ensure adequate hemostasis. A suggestion that can be considered and discussed with the anesthesiologist—who makes the final decision—is to use a specific type of anesthesia less associated with hemorrhagic complications.^177^As for antiplatelets, if they are interrupted, they should be resumed postoperatively as soon as possible. It is also recommended that procedures with a higher risk of CV events be performed in hospitals equipped for urgent hemodynamic intervention (coronary angioplasty) if needed.

Chart 14 presents recommendations for patients scheduled for ophthalmic surgery.

**Chart 14:** Recommendations for patients scheduled for ophthalmic surgery

Recommendation	Grade of recommendation	Level of evidence
For patients recommended to continue anticoagulation and/or antiplatelet therapy, the ophthalmologist should be informed of the need to maintain adequate hemostasis.	I	B
Acetylsalicylic acid (ASA) for secondary cardiovascular prevention should be maintained in the perioperative period of ophthalmic surgery.	I	B
Clopidogrel monotherapy should be interrupted in the perioperative period of glaucoma and vitrectomy surgery.	I	C
Warfarin should be interrupted in the perioperative period of vitrectomy and trabeculectomy.	I	B
Warfarin should be maintained in patients undergoing cataract surgery, provided the INR is within the therapeutic range.	I	B
Direct oral anticoagulants may be maintained in patients undergoing cataract surgery.	I	B
Clopidogrel monotherapy for secondary cardiovascular prevention should be maintained in the perioperative period of cataract surgery.	IIa	B
Dual antiplatelet therapy due to recent stent placement (see item 4.2) or acute coronary syndrome within the past year should be maintained in the perioperative period of low-risk interventions (intravitreal injections, cataract surgery, and peribulbar anesthesia).	IIa	B
In patients on dual antiplatelet therapy due to recent stent placement (see item 4.2) or acute coronary syndrome within the past year undergoing high-risk interventions (vitrectomy or trabeculectomy), ASA should be maintained and P2Y12 receptor inhibitors should be interrupted in the perioperative period.	IIa	C

### 3.4. Valvular Heart Disease

Patients with valvular heart disease (VHD) who are candidates for noncardiac surgery have a higher risk of perioperative CV complications.^191^This risk is related to the type and anatomical severity of the disease, the presence of symptoms, and the type of surgery.^6^The main CV complications that may occur in this setting include pulmonary congestion, acute pulmonary edema, cardiogenic shock, MI, tachyarrhythmias, embolic events, bleeding, and IE.^192^

Upon suspicion of VHD following a detailed clinical history and physical examination, a TTE should be performed. The TTE aims to assess the anatomical severity of the disease, ventricular function, cardiac remodeling, and to estimate pulmonary artery systolic pressure (PASP). If uncertainty remains, other diagnostic methods may be used, such as transesophageal echocardiography, computed tomography, magnetic resonance imaging, and cardiac catheterization.

Symptomatic patients with significant VHD present high morbidity and mortality and should undergo valvular intervention,^193 , 194^which should be performed before elective procedures. Conversely, emergency noncardiac procedures should be performed following specific recommendations for each VHD to minimize the chances of CV decompensation.

Heart valve stenosis carries a higher perioperative risk compared with valve regurgitation. Therefore, extra care should be taken with patients with aortic and/or mitral stenosis.^3 , 195^If there is more than one valvular lesion and/or a combination of stenosis and regurgitation, the approach should be based on the most significant lesion.

#### 3.4.1. Aortic Valve Stenosis

Aortic valve stenosis is a common type of VHD in elderly patients, affecting approximately 2% to 4% of adults over 75 years,^196^and this number is expected to increase in the coming years. Significant aortic stenosis is a well-established risk factor for perioperative mortality, HF, and MI in noncardiac surgery.^193^

Asymptomatic patients with significant aortic stenosis can safely undergo low-risk noncardiac surgery as long as volume overload is avoided.^191 , 193^In these cases, if there is doubt as to whether the patient is asymptomatic, a stress test should be performed. The Heart Team, including the surgeon and anesthesiologist, plays an important role in decision-making.

In symptomatic patients with significant aortic stenosis or those scheduled for moderate-to-high risk surgery, aortic valve intervention should be performed before noncardiac surgery.^197 - 199^Aortic valve replacement is associated with lower in-hospital and 30-day mortality in individuals undergoing intermediate- or high-risk surgery.^198 , 199^Additionally, a study by Mizuno et al.^197^with 218 patients demonstrated that those with aortic stenosis undergoing high-risk noncardiac surgery experienced faster progression of aortic stenosis compared with those who did not undergo surgical intervention. The choice between surgery or transcatheter aortic valve implantation (TAVI) should be based on the latest guidelines on VHD. When noncardiac surgery needs to be performed promptly, TAVI should be considered due to the quicker recovery associated with this procedure.^5^In cases where TAVI and heart valve surgery are unfeasible, balloon valvuloplasty is an alternative, although the risks of this procedure should be weighed and restenosis will occur later.^193 , 197 , 200^

If noncardiac surgery is urgent or time-sensitive, such as in the case of femur fracture, invasive hemodynamic monitoring during anesthesia is necessary, avoiding changes in volume status, especially due to the high risk of hypervolemia. In these cases, postoperative care in an intensive care unit (ICU) with continuous hemodynamic and ECG monitoring, as well as serial cardiac troponin (cTn) measurements, is recommended. In time-sensitive procedures, balloon valvuloplasty and TAVI may be considered depending on the anatomical severity of the disease and the availability of these resources.

#### 3.4.2. Mitral Valve Stenosis

Patients with mild-to-moderate mitral valve stenosis can undergo noncardiac surgery safely, provided measures are taken to prevent tachycardia and volume overload before and after the procedure.

Patients with significant mitral stenosis, even in the absence of symptoms, have a higher risk of CV complications. In cases for which surgical or percutaneous correction is indicated, patients should undergo the procedure before any elective noncardiac surgery.^201^In asymptomatic patients with significant mitral stenosis without an indication for interventional treatment, mitral valve intervention should be considered before any high-risk procedure.

In emergency noncardiac surgery, invasive hemodynamic monitoring and prevention of tachycardia and hypervolemia are recommended. Increased HR, especially if atrial fibrillation (AF) develops, can lead to congestion and pulmonary edema. Thus, the use of beta-blockers and/or diuretics can be implemented in the perioperative period.

#### 3.4.3. Aortic and Mitral Regurgitation

Primary valve regurgitation is associated with increased CV risk during noncardiac surgery, but it is better tolerated than valve stenosis.^202 , 203^Mild-to-moderate aortic and mitral regurgitation do not increase the risk of CV complications during noncardiac surgery. In the case of significant valvular dysfunction, provided there are no symptoms and ejection fraction is preserved, noncardiac operations can also be performed, with caution to avoid volume overload.

Conversely, patients with significant primary aortic or mitral regurgitation with an indication for heart valve replacement have an increased risk of CV complications and should undergo surgical correction before noncardiac surgery.^191^In symptomatic patients with moderate-to-severe secondary mitral regurgitation, it is important to assess, in conjunction with the Heart Team, whether the patient is a candidate for surgery or transcatheter edge-to-edge repair before the elective noncardiac surgery.^193^

If the noncardiac procedure is urgent or an emergency, it should be performed after optimization of pharmacological treatment and hemodynamic stabilization, preferably using vasodilators and diuretics, with postoperative care in an ICU.

#### 3.4.4. Prosthetic Heart Valves

Patients with normally functioning prosthetic heart valves and no left ventricular dysfunction can undergo noncardiac surgery without additional risk.

Chart 15 presents recommendations for patients with VHD.

**Chart 15:** Recommendations for patients with valvular heart disease

Recommendation	Grade of recommendation	Level of evidence
Echocardiography should be performed in patients with known or suspected moderate-to-significant valvular heart disease scheduled for intermediate- or high-risk surgery, without evaluation in the past 6 to 12 months or with clinical worsening.	I	C
Patients with valvular heart disease requiring interventional treatment should undergo cardiac treatment first, followed by the proposed noncardiac surgery.	I	B
Asymptomatic patients with significant aortic stenosis scheduled for intermediate- or high-risk noncardiac surgery should undergo correction of aortic stenosis before noncardiac surgery.	I	B
Asymptomatic patients with significant mitral stenosis and other risk factors (pulmonary artery systolic pressure [PASP] ≥ 50 mm Hg at rest or PASP ≥ 60 mm Hg on exertion) should undergo correction of mitral stenosis before noncardiac surgery.	I	C
Asymptomatic patients with significant valve regurgitation may undergo elective noncardiac surgery.	I	C

### 3.5. Solid Organ Transplantation

#### 3.5.1. Liver

Considered the treatment of choice for many end-stage liver diseases, liver transplantation has become a routine procedure in several centers. Advances in anesthetic approaches, increased surgical experience, and improved postoperative care have allowed practices that, just over a decade ago, would have been exceptions. Consequently, many groups have expanded transplant indications to increasingly older populations and more complex cases for which transplant was previously contraindicated.^204^Additionally, indications for nonalcoholic steatohepatitis, typically associated with obesity, diabetes, hypertension, and CVDs, have become more frequent, being leading causes of liver transplantation in some centers.^205^

Studies show that up to 26% of patients undergoing liver transplantation have at least one critical coronary artery stenosis.^206^Within 1 year after the transplant, 15.2% of patients have at least one CV event, and 2.8% die from it.^207^Thus, CVD is the third most common cause of death following liver transplantation, after infections and graft rejection or dysfunction. The most common CV events are arrhythmias, pulmonary edema, ventricular dysfunction, sudden death and AMI.^206^For these reasons, pretransplant CV evaluation is crucial to determine the correct management of these patients both preoperatively and postoperatively.

In addition to CAD-related events, other comorbidities commonly found in patients with liver disease can increase morbidity and mortality after liver transplantation. These include alcoholic cardiomyopathy, cirrhotic cardiomyopathy, pulmonary hypertension (PH), and hepatopulmonary syndrome (HPS).

Table 10 ^208^summarizes the main cardiopulmonary comorbidities of liver transplant candidates, clinical findings, diagnosis, and expected outcomes after the transplant.

**Table 10:** Prevalence of cardiopulmonary comorbidities in liver transplant candidates

	Prevalence	Expected outcome after liver transplantation
Coronary artery disease	2.5%-27%	Progression, expected worsening of risk factors
Cirrhotic cardiomyopathy	Unknown, but probably high	Reversal after liver transplantation
Valvular heart disease	Unknown, but probably low	Progression irrespective of liver transplantation
Chronic obstructive pulmonary disease	18%	Progression irrespective of liver transplantation
Hepatic hydrothorax	5%-12%	Resolution after liver transplantation
Hepatopulmonary syndrome	20%	Resolution after liver transplantation in most cases
Pulmonary hypertension	4%	Unchanged or slowly progressive

Adapted from Martinez-Palli et al.^208^

##### 3.5.1.1. Cirrhotic Cardiomyopathy

Cirrhotic cardiomyopathy is characterized by the following triad in patients with cirrhosis^209 , 210^: systolic dysfunction (primarily due to a deficit in stress-induced contractile response, with a resting LVEF < 55%), diastolic dysfunction, and electrophysiological abnormalities (especially prolonged QT intervals, chronotropic incompetence, and bradycardia, as well as ventricular repolarization abnormalities). Left atrial enlargement, increased myocardial mass, and elevated type B natriuretic peptide (BNP), N-terminal pro BNP (NT-proBNP), and cTnI levels are also commonly found.

Despite these findings increasing morbidity and mortality in liver transplant candidates, no specific treatment for these abnormalities has shown benefit.

##### 3.5.1.2. Alcoholic Cardiomyopathy

Alcoholic cardiomyopathy affects 21% to 32% of patients with dilated cardiomyopathy in some centers.^211 , 212^Considering that alcoholic cirrhosis is among the leading causes of liver disease, the concomitant occurrence of cirrhosis with dilated cardiomyopathy is relatively common.

##### 3.5.1.3. Portopulmonary Hypertension

The hyperdynamic state of patients with portal hypertension can lead to vasoconstriction and pulmonary vascular remodeling, resulting in PH. This condition affects 5% to 10% of transplant candidates and is not related to the etiology or severity of portal hypertension, even in the absence of liver cirrhosis.^213 , 214^Portopulmonary hypertension (POPH) is a subset of group 1 PH due to its pathological and hemodynamic similarities with other causes of precapillary PH. POPH can be classified, based on mean pulmonary artery pressure (mPAP), as mild (> 25 and < 35 mm Hg), moderate (> 35 and < 45 mm Hg), or severe (> 45 mm Hg).^213^Clinical status and medical tests present unique features.^215 , 216^

As the symptoms of POPH are nonspecific and its diagnosis directly impacts liver transplant eligibility, screening with TTE is recommended for all candidates. A PASP > 50 mm Hg has a high diagnostic accuracy for POPH in liver transplant candidates (97% sensitivity and 77% specificity for moderate-to-severe POPH).^217^In addition, echocardiography is useful to assess right ventricular function and rule out left ventricular dysfunction and/or VHD that could contribute to postcapillary PH.^216^The International Liver Transplantation Society guidelines suggest right heart catheterization for definitive POPH if estimated PASP on TTE is > 50 mm Hg. However, new evidence indicates increased diagnostic sensitivity with an estimated PASP cutoff > 40 mm Hg. Depending on service availability, a PASP between 40 and 45 mm Hg on echocardiography may indicate the need for cardiac catheterization, especially if there are other signs of PH ( Figure 3 ).^216 , 217^

**Figure 3:** Evaluation of pulmonary artery pressure in liver transplant candidates. mPAP: mean pulmonary arterial pressure; PASP: pulmonary artery systolic pressure; PH: pulmonary hypertension; TTE: transthoracic echocardiogram. Adapted from Cartin-Ceba and Krowka.^214^

The diagnosis of POPH involves ruling out other causes of PH and the presence of the following hemodynamic criteria confirmed by right heart catheterization^216^:

• mPAP > 25 mm Hg.
• Pulmonary vascular resistance (PVR) > 3 Wood units (or 240 dyn.s.cm-5).
• Pulmonary capillary wedge pressure (PCWP) < 15 mm Hg. If PCWP > 15 mm Hg, likely due to hypervolemia (common in patients with cirrhosis), a transpulmonary pressure gradient (mPAP–PCWP) > 12 mm Hg suggests the presence of POPH.

Importantly, approximately 20% of patients with cirrhosis have a moderate increase in pulmonary pressure on echocardiography, but only a small fraction of these actually have POPH.^218^

Management of POPH depends on the presence of hemodynamic criteria assessed by right heart catheterization ( Figure 3 ).^214^In patients with a mPAP between 35 mm Hg and 50 mm Hg and increased PVR (> 240), liver transplantation cannot be performed unless there is a reduction in mPAP and PVR after pharmacological treatment of PH. The medications used in the treatment of PH have vasodilatory, antiplatelet, and antiproliferative effects, which can reduce pulmonary artery pressure.^214^The main available medication classes are:

• Phosphodiesterase inhibitors (eg, sildenafil).
• Endothelin receptor antagonists (eg, bosentan, ambrisentan, macitentan).
• Soluble guanylate cyclase stimulators (eg, riociguat).
• Prostanoids (eg, epoprostenol, iloprost, treprostinil).

However, an equivalence in morbidity and mortality between patients with HP treated with these medications and those without a diagnosis of PH has not yet been demonstrated. Further studies are needed in this area.

Perioperative mortality in patients with severe POPH is close to 100%, making it a contraindication for isolated liver transplantation. In select centers, combined lung-liver transplantation should be considered.^219^

##### 3.5.1.4. Hepatopulmonary Syndrome

Although sometimes mistaken for POPH, HPS presents different characteristics, such as hypoxia in the presence of liver disease that worsens in the upright position, with evidence of intrapulmonary vasodilation. Hypoxia occurs due to vasodilation in the pulmonary vascular bed, induced mainly by the accumulation of nitric oxide, leading to intrapulmonary arteriovenous shunting.^220^

HPS is diagnosed by the presence of the following triad^214^:

• Portal hypertension, chronic liver disease, or congenital portosystemic shunts.
• Hypoxemia (alveolar–arterial gradient > 15 mm Hg or > 20 mm Hg if age > 64 years).
• Intrapulmonary vasodilation, leading to shunting (visualization of bubbles in the left heart after 3 cardiac cycles on contrast echocardiography with microbubbles).

HPS affects 5% to 32% of transplant candidates and is not related to the etiology or severity of liver disease.^221 , 222^

The typical clinical presentation includes signs of chronic liver disease associated with cyanosis, nail clubbing, dyspnea when standing upright (platypnea), and hypoxia when also standing upright (orthodeoxia). Hypoxemia is a prominent finding in this syndrome.^214^

Screening is conducted with pulse oximetry. An oxygen saturation < 96% has a sensitivity of 100% and specificity of 88% for detecting PaO_2_< 60 mm Hg.^214^Therefore, arterial blood gas analysis is recommended in these patients. If arterial blood gas analysis shows an elevated alveolar–arterial gradient (≥ 15 mm Hg or > 20 mm Hg if age > 64 years) and a PaO_2_< 80 mm Hg, a transthoracic echocardiogram with microbubbles should be performed.^214^If there is an intracardiac shunt (eg, atrial septal defect, patent foramen ovale), the bubbles pass to the left heart 1 or 2 cardiac cycles after the visualization of bubbles in the right atrium. If these bubbles appear in the left atrium after 3 cardiac cycles, the presence of a pulmonary shunt is confirmed (the bubbles can pass through the dilated pulmonary vessels in HPS). It is important to note that echocardiography with microbubble contrast agents is sensitive but not specific for HPS, as many patients with cirrhosis have a positive test (intrapulmonary shunts) without meeting the criteria for HPS due to the absence of hypoxemia. A more specific test for HPS is Tc-99m macroaggregated albumin scintigraphy.^214^

The management of HPS consists of oxygen support, although it does not show sustained benefits in improving dyspnea and quality of life for these patients. Several pharmacological therapies have been tested, but none have shown proven benefits.^214^

Unlike PH, the treatment of choice for HPS is liver transplantation, although nondefinitive data correlate the degree of hypoxia with perioperative mortality.^223^

##### 3.5.1.5. Coronary Artery Disease

Risk factors for CAD are as prevalent or even more prevalent in patients with cirrhosis than in the general population. In patients with advanced liver disease, the prevalence of diabetes and CAD is equal to or greater than in the general population. Particularly in patients with diabetes, the prevalence of CAD ranges from 2.5% to 27%.^224 , 225^Conversely, the extent of the disease (degree of stenosis) does not appear to correlate with a worse prognosis.^226^A comparison between liver and kidney transplant candidates revealed that the latter have a higher frequency of diabetes, hypertension, and CAD.^227^

###### 3.5.1.5.1. When to Investigate CAD?

Indications for noninvasive tests to assess CV risk in liver transplant candidates vary among medical societies: some recommend it for all liver transplant candidates, while others suggest CV risk stratification before requesting a test.^228^An interesting strategy for asymptomatic patients with CAD awaiting liver transplantation is to evaluate the presence of CV risk factors (male sex, hypertension, diabetes, dyslipidemia, past or current smoking, and a family history of early CAD).^219^If the patient does not have any risk factor or has 1 risk factor, the negative predictive value for obstructive CAD is high, so there is no need to request a noninvasive test. However, if the patient has 2 or more risk factors, noninvasive testing is indicated.^225^

For symptomatic patients, usual CAD evaluation should be performed as recommended in specific guidelines, stratifying the patient according to symptoms (eg, angina and dyspnea) and risk factors (eg, sex, age).^228 , 226^After stratification, the pretest probability of CAD is determined. If the probability is low/intermediate (< 15%), noninvasive tests (anatomic or functional) may be performed, or the investigation may be interrupted if the probability of CAD is very low. If the probability is high (> 15%), preference should be given to cardiac catheterization. In general, the preference for invasive approaches is given to patients with significant angina refractory to medical treatment, left ventricular dysfunction, or high-risk findings in noninvasive tests.^228^

###### 3.5.1.5.2. Which Test to Request for CAD Investigation?

Once the patient’s risk has been determined, the best complementary method for CAD investigation can be chosen. Due to the characteristics of liver transplant candidates, this investigation lacks formal standardization. Many patients have mobility limitations, ascites, and neuropathy due to the chronic nature of the disease, preventing, for example, the performance of exercise ECG. Furthermore, approximately 50% of patients with cirrhosis have prolonged QT intervals, which can hinder test evaluation.^216^Additionally, terminal liver disease is characterized by vasodilation and tachycardia, impairing the response induced by vasodilators (dipyridamole and adenosine) in myocardial scintigraphy. Beta-adrenergic receptors respond poorly to sympathetic stimuli, leading to inconclusive responses on DSE.^215 , 217 , 218^Comparisons between the use of dobutamine vs. vasodilators as pharmacological stress agents in liver transplant candidates favor dobutamine, particularly DSE.^218 , 219^The choice of which test to use should also respect the regional characteristics of each center.

CCTA has also been investigated in this setting and, when normal or showing nonobstructive coronary lesions, achieved a negative predictive value of 95% for MACE and 100% for clinical coronary events. A calcium score > 400 has high predictive power for early CV events in these patients.^226^Care should be taken with CCTA and cardiac catheterization due to the use of contrast agents. Terminal liver disease is usually accompanied by renal dysfunction, which should be considered before indicating any of these tests.

###### 3.5.1.5.3. When and How to Intervene in CAD?

The decision to treat CAD should be made when the lack of intervention could lead to excessive risk during and after noncardiac surgery. However, the best treatment option has not been established and should be individualized for each patient.

The indication for percutaneous procedures with stent placement (current consensus is that these should be drug-eluting stents) should consider the time-sensitive nature of liver transplant surgery, opting for the choice that has the least impact on waiting list time.

Surgical revascularization should, when possible, be postponed until after the transplant due to the high risk of hemorrhagic events or worsening liver condition associated with the surgery. Coronary artery bypass graft surgery (CABG) before transplantation should be reserved only for patients in whom the risk of death from CAD exceeds the risk of death from liver disease. CABG can be performed after liver transplantation with reasonable safety and low complication rates.^229 , 230^

Chart 16 presents recommendations for patients awaiting liver transplantation.

**Chart 16:** Recommendations for patients awaiting liver transplant

Recommendation	Grade of recommendation	Level of evidence
An electrocardiogram and chest radiograph must be requested for all patients.	I	C
An echocardiogram must be requested for all patients.	I	B
In patients with an echocardiogram showing a pulmonary artery systolic pressure (PASP) > 50 mm Hg, right heart catheterization with measurement of pulmonary artery pressure must be requested.	I	C
In patients with an echocardiogram showing a PASP > 40 mm Hg, especially if other signs of pulmonary hypertension (PH) are present, right heart catheterization with measurement of pulmonary artery pressure should be considered.	IIa	C
In asymptomatic patients with coronary artery disease (CAD) without segmental dysfunction on echocardiography and with 2 or more risk factors for CAD * , DSE should be preferably requested.	IIa	B
In symptomatic patients with CAD, the pretest probability of CAD should be calculated, and additional tests should be requested according to specific guidelines.	IIa	C
Coronary cineangiography should be performed in patients with a high pretest probability of CAD, significant angina refractory to clinical treatment, new left ventricular dysfunction, or high-risk findings on noninvasive tests, despite hemorrhagic complications being more common and alterations such as elevated creatinine potentially contributing to increased morbidity in patients with cirrhosis.	IIa	C
In patients scheduled for percutaneous coronary intervention with stent placement (when necessary prior to transplantation due to CAD severity), the possibility of the patient dying from liver disease while awaiting the antiplatelet period and the real benefit of the intervention in minimizing perioperative risks should be considered.	IIa	C
Coronary artery bypass graft before transplantation should be reserved only for patients in whom the risk of death from CAD exceeds the risk of death from liver disease, and this decision should be discussed with a multidisciplinary team.	IIa	C
Pulmonary vasodilators may be used to try to reduce PH in patients with a mean pulmonary artery pressure (mPAP) between 35 and 45 mm Hg and increased systemic vascular resistance (> 240 dynes).	IIb	B
Patients with a mPAP ≥ 35 mm Hg on right heart catheterization should be referred to a PH specialist.^213^	IIb	C
Liver transplantation in patients with severe PH in centers that do not offer aggressive therapies to reduce PAP or the possibility of combined lung-kidney transplantation.	III	B

* Male sex, hypertension, diabetes, dyslipidemia, past or current smoking, and family history of early CAD.

#### 3.5.2. Kidney

Patients with stage 5 chronic kidney disease (CKD) (glomerular filtration rate < 15 mL/min/1.73 m^2^) undergoing dialysis or not constitute one of the highest CV risk groups, with CV mortality rates 5 to 100 times higher than those in the general population for the same age group. Although kidney transplant recipients have a lower rate of CV complications compared with patients maintained on dialysis, it is still higher than in the general population. In fact, CVD is the leading cause of post-transplant death, especially due to CAD.^231^The post-transplant period presents a high risk of MI, particularly during the first year.^232^In a cohort of 600 patients, Gill et al. reported a significant increase in the incidence of CV events in the first year after transplantation (39.6/100 patient-years, 95%CI 20.6-76.1) compared with the pre-transplant period.^233^In the first 30 days after kidney transplantation, approximately half of deaths result from MI.^234^Similarly, in the long-term follow-up, chronic ischemic heart disease accounts for over a third of deaths in patients with functioning grafts.^234^

Thus, the preoperative evaluation of kidney transplant candidates aims not only to reduce the short-term CV risk related to the procedure but also to reduce late CV events. During the evaluation of kidney transplant candidates, identifying the presence and extent of CAD is of fundamental importance as it allows the medical team to more accurately establish the risk/benefit of transplantation, the potential need for preoperative coronary intervention, the use of perioperative cardioprotective measures, and the control of risk factors postoperatively.^235 , 236^

The purpose of this section is to provide cardiologists with the most appropriate means of establishing CV risk in a very special population, almost always excluded from surgical risk stratification studies. The main goal is to identify, among kidney transplant candidates, those most likely of being diagnosed with CAD. Thus, the recommendations included in this section should only be applied to asymptomatic patients or those with atypical symptoms; for those individuals with clinical evidence and/or diagnostic findings suggestive of CAD, complementary investigation and treatment should follow the recommendations proposed for the general population contained in specific sections of this Guideline.

Identifying CAD is a significant challenge in kidney transplant candidates. Significant obstructive CAD, defined as stenosis ≥ 70% in major epicardial vessels (or ≥ 50% in the left main coronary artery), is described in kidney transplant candidates, being observed in up to 50% of individuals depending on the inclusion criteria for angiography.^237 , 238^However, in the presence of advanced CAD, these patients are commonly asymptomatic or present with atypical symptoms. Noninvasive methods for detecting MI, such as exercise ECG, MPI, or pharmacological stress echocardiography—all routinely used in the general population—, show lower sensitivity and specificity in patients with CKD than in individuals with normal renal function, leading to a great number of false-negative results.^239 - 241^More recently, the ISCHEMIA-CKD study showed in a population of patients with stage 4 and 5 CKD that myocardial revascularization based on severity of ischemia was not superior to optimized clinical treatment in the occurrence of composite CV outcomes. It is worth noting that the study did not specifically target patients on dialysis, as only 53.4% of included patients were on renal replacement therapy. Nonetheless, there was no significant interaction between the treatment effect (intervention vs. conservative treatment) for the primary outcome in the dialysis vs. nondialysis subgroups.^242^

The indiscriminate use of coronary cineangiography is not justified as it is an invasive method, not free of complications, costly, and results in more than 50% of normal results or without significant obstructive lesions. Conversely, observational studies suggest that patients with significant CAD have a high risk of events and CV death.^243^Advances in CV imaging may lead to the replacement of invasive evaluation with noninvasive tests to document not only the presence and extent of coronary atherosclerosis but also its functional significance.^244 , 245^

To date, evidence does not support the indication of intervention based on the finding of obstructive CAD during pre-kidney transplant evaluation.^72 , 246 - 248^In a retrospective study involving 406 patients undergoing invasive coronary angiography before kidney transplantation, the occurrence of CV events (CV death, acute coronary syndrome, or the need for post-transplant revascularization) was similar between patients with obstructive CAD and revascularization (23%) and those with CAD and no revascularization (26%) during a follow-up period of 5 years.^249^

Thus, the investigation of the presence and extent of obstructive CAD in kidney transplant candidates should be just one step in the overall risk assessment of this population, to identify individuals most likely to benefit from revascularization and who should therefore be referred for angiography. Such a strategy should reduce the number of unnecessary invasive tests, optimizing available economic resources,^250^and lead to a more favorable relationship between the inherent risk of intervention (greater in these patients than in the general population) and long-term prognostic benefit.

##### 3.5.2.1. CAD Risk Stratification

There is no consensus regarding the best strategy for investigating and treating CAD in kidney transplant candidates.^251^This topic is controversial, with some suggesting the complete elimination of screening for occult CAD in asymptomatic individuals.^252^This is primarily due to the lack of prospective studies in this population.^253^However, it is known that the clinical parameters most strongly associated with ischemic heart disease after kidney transplantation are age > 50 years, diabetes, and previous evidence of CVD (clinical history and/or test findings). The prevalence of significant CAD (stenosis ≥ 70%) increases with the number of risk factors present. These three risk factors have served as the basis for formulating algorithms for investigating CAD in patients with CKD.^254^Other factors considered predictors of CV events in kidney transplant candidates include systemic hypertension, ventricular dysfunction, left ventricular hypertrophy, smoking, dyslipidemia, and time on dialysis > 1 year. In general, traditional risk factors have less impact on renal patients, and risk scores such as the Framingham score underestimate the actual risk of events in these patients.^255^

Based on the results of mostly observational studies, we proposed a model for CV risk stratification in asymptomatic patients with CKD being screened for kidney transplantation, according to the presence or absence of the three cited risk factors ( Figure 4 ).^256^If there is any delay between the initial stratification and the transplant, we suggest a period of 3 years for restratification, provided the patient remains stable and without new symptoms or CV events.

**Figure 4:** Flowchart of perioperative assessment of kidney transplant candidates. CVD: cardiovascular disease; MPI: myocardial perfusion imaging; PO: postoperative.

High-sensitivity cTn (hs-cTn) levels can be used as an additional predictor of perioperative CV events in kidney transplantation. We recommend measuring hs-cTn levels in all patients before the procedure and serially at 24 and 48 hours after the transplant.^257^An absolute increase in hs-cTn concentration on postoperative days 1 or 2 compared with preoperative values is indicative of perioperative infarction/injury. In the absence of baseline values, a significant increase in hs-cTn concentration on postoperative day 1 (eg, > 5 times the upper reference limit [URL]) or a significant change in these concentrations between days 1 and 2 (absolute rise or fall above the URL vs day 1) also indicates the occurrence of perioperativeinfarction/injury.

Finally, we recommend that decision-making regarding diagnostic investigation in asymptomatic kidney transplant candidates and in the face of potential obstructive CAD findings be made by a multidisciplinary team (Heart-Kidney Team).^258^

Chart 17 presents recommendations for the perioperative risk assessment of kidney transplant candidates.

**Chart 17:** Recommendations for the perioperative risk assessment of kidney transplant candidates

Recommendation	Grade of recommendation	Level of evidence
All kidney transplant candidates should be evaluated for the presence and severity of cardiovascular disease based on clinical history, physical examination, and routine tests.	I	A
Patients without major risk factors * are considered at low cardiovascular risk and can be cleared for kidney transplantation without the need for additional investigation.	I	C
Measurement of high-sensitivity troponin is recommended before and 24 and 48 hours after kidney transplantation to detect perioperative infarction/injury.	I	B
Diagnostic decision-making and the definition of a therapeutic strategy should be discussed by a Heart-Kidney Team, including a clinical cardiologist, interventional cardiologist, cardiovascular surgeon, nephrologist, and/or kidney transplant specialist.	I	C
Patients with only 1 major risk factor * are considered at intermediate cardiovascular risk and should undergo functional imaging for myocardial ischaemia If there is evidence of stress-induced myocardial ischemia, proceed with invasive investigation via coronary angiography; if there is no evidence of stress-induced ischemia or other findings suggestive of coronary artery disease (CAD) (eg, reduced LVEF), the patient can be cleared for kidney transplantation.	IIa	C
Patients with at least 2 major risk factors * are considered at high cardiovascular risk and should be directly referred for coronary angiography before kidney transplantation.	IIa	C
Stable patients with obstructive CAD should be clinically reassessed for disease progression every 12 months; patients without significant obstructive CAD should be reassessed every 36 months to detect de novo CAD.	IIa	C
Asymptomatic patients with obstructive CAD should not be routinely referred for myocardial revascularization only because they might undergo kidney transplantation ("prophylactic intervention"), unless there is an unequivocal prognostic impact of the intervention.	III	A

* Age > 50 years, diabetes, and previous evidence of cardiovascular disease.

## 4. Measures for Reducing CV Surgical Risk

### 4.1. Perioperative Pharmacological Therapy

#### 4.1.1. Beta-Blockers

Although early studies suggested the benefits of beta-blockers in reducing perioperative CV events, this topic remains somewhat controversial, and the best approach seems to be individualized treatment.^259^Indeed, the benefit of beta-blockers was shown to be directly associated with individual CV risk, with benefits observed in high-risk patients, whereas no such benefit was seen in low-risk ones.^260^In this context, the POISE study (Effects of Extended-Release Metoprolol Succinate in Patients Undergoing Non-Cardiac Surgery) evaluated the use of beta-blockers in high-risk patients or those with established atherosclerotic disease and demonstrated a reduction in AMI, but with an increase in the incidence of stroke and overall mortality, which was likely associated with increased rates of bradycardia and hypotension.^261^The major criticism of the study was that metoprolol was introduced 2 to 4 hours before the surgical procedure, potentially reaching a dose of 400 mg per day, which is rarely used in clinical practice and very close to the maximum recommended dose. This means there was insufficient time for more appropriate titration of the medication. However, when started at least a week in advance, beta-blockers demonstrated a reduction in long-term mortality in patients undergoing vascular surgery.^262^

Therefore, in general, we suggest that beta-blockers should be maintained in chronic users. Additionally, in patients who are not already on beta-blockers, they should not be introduced in the week (7 days) preceding the procedure.

Chart 18 shows recommendations for the perioperative management of beta-blockers.

**Chart 18:** Recommendations for the perioperative management of beta-blockers

Recommendations	Grade of recommendation	Level of evidence
Chronic therapy (> 7 days) with beta-blockers should be maintained perioperatively.	I	B
The introduction of beta-blockers within 7 days before surgery is not recommended in patients not already on beta-blockers.	III	B

#### 4.1.2. Statins

Statins, in addition to reducing cholesterol levels, have pleiotropic effects, which reduce inflammation and stabilize atherosclerotic plaques. The use of statins for the prevention of CV events following vascular surgery is well-established, based on prospective, randomized, and placebo-controlled studies. In 2004, a randomized study involving 100 patients demonstrated that the use of 20 mg of atorvastatin was associated with a significant reduction in major CV events (death, AMI, stroke, unstable angina) in the perioperative period and at 6 months of follow-up, regardless of baseline cholesterol levels.^263^In 2009, the use of fluvastatin slow-release (xl 80 mg) in 250 patients undergoing vascular surgery was shown to reduce the occurrence of postoperative MI and the combined outcome of AMI and cardiac arrest at 30 days compared with placebo (247 patients).^264^These data were confirmed in a meta-analysis involving 23,536 patients, in which the perioperative use of statins in vascular surgery reduced overall mortality and the rates of AMI and stroke.^265^Atorvastatin 20 mg in patients undergoing vascular surgery should preferably be introduced 2 weeks before the procedure and maintained for 30 days. After this period, the dose should be adjusted according to each patient’s LDL target.

Conversely, evidence on the use of statins for preventing CV complications in nonvascular surgery is more heterogeneous. Lindenauer et al.^266^evaluated 780,591 patients undergoing noncardiac surgery (92% nonvascular) in a retrospective cohort study, of whom 77,082 (9.9%) received statins. Patients who received statins had lower in-hospital mortality. Another retrospective case-control study, including 989 patients who died within 30 days postoperatively and 1,879 controls, demonstrated that statin use was also associated with reduced mortality (OR 0.4, 95%CI 0.24-0.68).^267^In a retrospective cohort study including 752 patients undergoing nonvascular surgery, a reduction in the composite outcome of nonfatal MI, AF, and 30-day mortality was seen in patients using statins.^268^In an analysis of patients included in the VISION study, Berwanger et al. evaluated 2,842 patients who received statins and 4,492 controls and compared the occurrence of all-cause mortality, postoperative cardiac troponin elevation myocardial injury after noncardiac surgery (MINS) (ie, without evidence of a nonischemic etiology), or stroke at 30 days after surgery using propensity score matching. Statin use was associated with lower risk of the composite outcome (relative risk [RR] 0.83; IC95% 0.73-0.95; p = 0.007). Statins were also associated with a lower risk of all-cause mortality (RR 0.58, 95%CI 0.40-0.83, p = 0.003), CV mortality (RR 0.42, 95%CI 0.23-0.76, p = 0.004), and MINS (RR 0.86; 95%CI 0.73-0.98; p = 0.02). There was no reduction in non-CV mortality, MI, or stroke. It is noteworthy that, despite the propensity score matching analysis, patients in the statin group more frequently had CAD, diabetes, peripheral vascular disease, and were more likely to use ASA and ACE inhibitors/ARBs compared with patients not treated with statins. Therefore, despite having more risk factors, patients in the statin group had fewer CV events.^269^In a recent retrospective study involving 180,000 patients, statin use on the day of surgery or postoperative day 1 was associated with lower 30-day mortality.^270^However, the randomized LOAD study, in which 648 patients were randomized to a loading dose of atorvastatin 80 mg before surgery (or placebo) followed by a dose of 40 mg (or placebo) for 7 days, showed no difference in the combined outcome of all-cause mortality, nonfatal MI, and stroke at 30 days (hazard ratio 0.87, 95%CI 0.60-1.26, p = 0.46).^271^It should be noted that the study lacked statistical power to draw definitive conclusions. Conversely, in a meta-analysis of randomized controlled trials, Putzu et al. demonstrated that perioperative statin use was associated with a lower occurrence of perioperative MI, without reducing mortality.^272^Thus, due to conflicting results, routine statin prescription to reduce complications in patients undergoing nonvascular surgery is not recommended. However, patients with an indication for statin use due to comorbidities (CAD, diabetes, peripheral vascular disease) regardless of the perioperative context may benefit from statin initiation in the perioperative period of nonvascular surgery.

Statins are often discontinued postoperatively. The main reasons for discontinuing statins are postoperative ileus and the impossibility of administering oral medications, hemodynamic instability, concerns about side effects, and lack of awareness of the importance of maintaining statin use. The perioperative discontinuation of statins in chronic users is an independent predictor of CV events after vascular surgery.^273 , 274^Perioperative statin use is safe. Although patients on statins have higher baseline creatine phosphokinase levels, elevations exceeding 5 times the reference value or rhabdomyolysis are rare.^273^Therefore, in patients already using statins, the medication should be maintained throughout the perioperative period.

Chart 19 presents the recommendations for the perioperative use of statins.

**Chart 19:** Recommendations for the perioperative use of statins

Recommendation	Grade of recommendation	Level of evidence
Patients scheduled for vascular surgery.	I	A
Maintenance of statins in patients already using statins.	I	B
Patients undergoing nonvascular surgery with clinical indications for statin use due to associated diseases (coronary artery disease, cerebrovascular disease, peripheral arterial disease, diabetes), regardless of the perioperative setting.	IIa	C

#### 4.1.3. Antiplatelet Medications

Surgical procedures performed while the patient is on antiplatelet therapy are associated with an increased risk of bleeding^139 , 275^; however, discontinuing antiplatelets can lead to a rebound effect^276^and an increase in the occurrence of atherothrombotic events.^139 , 277^

##### 4.1.3.1. Monotherapy with ASA

Regarding the use of ASA, several studies, such as the POISE-2,^275^which evaluated 10,010 patients (70% in primary prevention), support the discontinuation of ASA for primary prevention 7 days before surgery and its nonreintroduction postoperatively.

Conversely, patients on ASA for secondary prevention should continue its use throughout the perioperative period to avoid rebound effects and increased risk of atherothrombotic events.^139 , 278 , 279^The only exceptions include neurosurgery, in which any minimal bleeding can be catastrophic, and transurethral resection of the prostate,^139^which lacks direct hemostasis is are associated with a higher bleeding risk. In these cases, ASA should be discontinued 7 days before surgery. However, for patients undergoing transurethral resection of the prostate with the use of greenlight lasers, ASA may be maintained.^280 , 281^Additionally, there is no recommendation for the routine discontinuation of ASA for transrectal biopsy, a common urology procedure.^280^

There is no recommendation to introduce ASA before noncardiac surgery. If patients with established vascular disease were not previously on antiplatelet therapy, this Guideline recommends, by expert consensus, that this therapy should be planned at hospital discharge, because no studies currently support starting ASA before surgery.

##### 4.1.3.2. Monotherapy with Antiplatelet Medications Other than ASA

Although ASA remains the most prescribed antiplatelet medication for monotherapy, many patients use a substitute due to allergy, gastrointestinal intolerance, a history of stroke, or according to new coronary syndrome guidelines.^282^For patients on clopidogrel monotherapy, the risk of bleeding during surgery is increased.^140^However, there are no studies demonstrating the safety of its discontinuation in relation to CV events. Therefore, the evidence on ASA maintenance or discontinuation cannot be extrapolated to clopidogrel.

The experts involved in the writing of this Guideline recommend maintaining clopidogrel during the perioperative period of noncardiac surgery with a low risk of bleeding, such as dermatological interventions. However, for procedures with a moderate-to-high-risk of bleeding, clopidogrel should be discontinued 5 days before the procedure.

For patients on antiplatelet monotherapy with ticagrelor, it should be stopped 5 days before the surgery. In the case of prasugrel, it should be discontinued 7 days before surgery. For patients on ticagrelor and prasugrel undergoing noncardiac surgery with a low risk of bleeding, such as dermatological interventions, there is insufficient scientific evidence. It is recommended that each case be individually assessed by a multidisciplinary team.

Chart 20 presents recommendations for the perioperative management of antiplatelet monotherapy.

**Chart 20:** Recommendations for the perioperative management of antiplatelet monotherapy

Recommendation	Grade of recommendation	Level of evidence
Acetylsalicylic acid (ASA) for primary prevention should be discontinued 7 days before noncardiac surgery.	I	A
ASA for secondary prevention should be maintained at 100 mg daily throughout the perioperative period, except in patients undergoing neurosurgery or procedures with prohibitive bleeding risk.	I	A
Clopidogrel monotherapy may be maintained in patients undergoing procedures with a low risk of bleeding but should be discontinued 5 days before procedures with a moderate- or high-risk of bleeding.	IIb	C
Ticagrelor monotherapy should be discontinued 5 days before surgery.	IIb	C
Prasugrel monotherapy should be discontinued 7 days before surgery.	IIb	C

##### 4.1.3.3. Dual Antiplatelet Therapy

For updated recommendations on the management of DAPT, refer to the specific guideline "Focus on Managing Patients with Percutaneous Coronary Intervention – 2022." A summary of the recommendations is presented below.^144^

Chart 21 presents recommendations for the perioperative management of DAPT.

**Chart 21:** Recommendations for the perioperative management of dual antiplatelet therapy

Recommendation	Grade of recommendation	Level of evidence
Acetylsalicylic acid should be maintained at 100 mg daily throughout the perioperative period, except in patients undergoing neurosurgery or procedures with prohibitive bleeding risk.	I	A
Clopidogrel and ticagrelor should be discontinued 5 days before noncardiac surgery.	I	B
Prasugrel should be discontinued 7 days before noncardiac surgery.	I	B
For early interruption of dual antiplatelet therapy (DAPT) before the minimum duration time, noncardiac surgery should be performed in centers with multidisciplinary care and hemodynamic monitoring.	I	C
Operations with a low risk of bleeding may be performed during DAPT if the interval since angioplasty is less than 3 months.	IIa	C
A platelet aggregation test should be used to reduce the discontinuation time of P2Y12 inhibitors before noncardiac surgery.	IIb	B
In cases with very high thrombotic risk (less than 1 month since percutaneous coronary intervention and DAPT interruption), bridging therapy with tirofiban should be used.	IIb	B
Bridging therapy with low-molecular-weight heparin.	III	B
Routine platelet aggregation testing should be performed to assess the discontinuation of ASA or P2Y12 inhibitors before noncardiac surgery.	III	C

### 4.2. Myocardial Revascularization

Recent evidence has failed to demonstrate the beneficial role of prophylactic myocardial revascularization in patients with stable CAD in the preoperative period of noncardiac surgery.^71 , 283^This is due to advancements in pharmacological therapy and, consequently, perioperative medication management, which make the potential benefit of prophylactic myocardial revascularization increasingly restricted. Therefore, the indications for myocardial revascularization before noncardiac surgery are the same as outside the perioperative period, aiming to improve the long-term prognosis and not just to reduce perioperative ischemic events.

For cases with unequivocal indications for myocardial revascularization in patients scheduled for noncardiac surgery, factors such as clinical stability, prognosis of the underlying condition that prompted the surgical indication, and potential risk of bleeding from the procedure should be considered in decision-making. Regarding the interval between myocardial revascularization and noncardiac surgery, no study has specifically investigated this topic. Therefore, the optimal interval refers to the necessary recovery time after cardiac surgery (approximately 30 days), with no minimum established period, requiring individual assessment considering the patient’s general clinical status and the urgency of the noncardiac surgery.

Conversely, the interval between myocardial revascularization and noncardiac surgery is crucial in cases of percutaneous coronary intervention (PCI).

The latest evidence on the safe interval between myocardial revascularization and noncardiac surgery is available in the specific guideline "Focus on Managing Patients with Percutaneous Coronary Intervention – 2022." Below is a summary of the recommendations. The classes of recommendation in this Guideline reflect available evidence, common sense, and clinical experience, particularly regarding the urgency of noncardiac surgery. For this reason, shorter intervals were attributed a weaker class of recommendation but are justifiable in individual cases of urgent noncardiac surgery.^144^

Chart 22 presents recommendations for the interval between myocardial revascularization and noncardiac surgery in patients undergoing elective percutaneous coronary interventions.

**Chart 22:** Recommendations for the interval between myocardial revascularization and noncardiac surgery in patients undergoing elective percutaneous coronary interventions

Recommendation	Grade of recommendation	Level of evidence
≥ 6 months	I	A
Between 3 and 6 months	IIa	B
Between 30 days and 3 months	IIb	B
< 30 days	III	B

Chart 23 presents recommendations for the interval between myocardial revascularization and noncardiac surgery in patients undergoing percutaneous coronary interventions due to acute coronary syndromes.

**Chart 23:** Recommendations for the interval between myocardial revascularization and noncardiac surgery in patients undergoing percutaneous coronary interventions due to acute coronary syndromes

Recommendation	Grade of recommendation	Level of evidence
≥ 12 months	I	A
Between 6 and 12 months	IIa	B
Between 30 days and 6 months	IIb	B
< 30 days	III	B

### 4.3. Perioperative Anticoagulation Management

Anticoagulation is increasingly common in clinical practice for patients with AF, VTE prevention or treatment, and in patients with mechanical heart valves. It is estimated that a quarter of these patients will require temporary interruption of anticoagulation for a scheduled surgical intervention within 2 years after starting therapy.^284^The main challenge of perioperative anticoagulation management is that interruption of anticoagulation therapy temporarily increases thromboembolic risk, while its maintenance during invasive procedures can increase the risk of bleeding events, both of which increase the risk of mortality.^285 - 288^When assessing perioperative thromboembolic risk, it is essential to recognize different risk situations. One involves patients receiving anticoagulation therapy for VTE prevention, while the other includes patients on anticoagulation therapy due to mechanical heart valves and/or AF to prevent arterial thromboembolism. Table 11 provides a proposed risk stratification for these patients, considering high-risk individuals as those with an annual thromboembolism risk of > 10%, moderate risk 5%-10%, and low risk < 5%.^289 - 291^Patients with mechanical heart valves are generally considered at high thromboembolic risk.

**Table 11:** Risk stratification for thromboembolism

Risk classification	AF	VTE
High *	CHADS_2_score of 5 or 6 Recent stroke or TIA (< 3 months) Rheumatic heart disease	Recent VTE (< 3 months) Severe thrombophilia †
Moderate	CHADS_2_score of 3 or 4	VTE 3-12 months ago Mild thrombophilia ‡ New VTE Active cancer
Low	CHADS_2_score of 0 to 2 (no previous stroke or previous TIA)	VTE > 12 months without other risk factors

CHADS_2_score: heart failure = 1 point; hypertension = 1 point; age > 75 years = 1 point; diabetes = 1 point; stroke/TIA = 2 points.

* High-risk patients may also include those with stroke or TIA > 3 months before planned surgery with a CHADS_2_score < 5, those who developed thromboembolism during temporary anticoagulation interruption, or those undergoing operations with a high risk of stroke or other type of thromboembolism (eg, heart valve replacement, carotid endarterectomy, major vascular surgery).

† Severe thrombophilia: deficiency of protein C, S, antithrombin or presence of antiphospholipid antibodies.

‡ Mild thrombophilia: heterozygous mutation of factor V Leiden or the prothrombin gene. AF: atrial fibrillation; TIA: transient ischemic attack; VTE: venous thromboembolism.

In addition to evaluating thromboembolic risk, the bleeding risk associated with certain surgical procedures while patients are on antithrombotic medications should also be considered. Table 12 describes the risk of bleeding associated with different surgical procedures.^286^In general, procedures are divided into those with a high risk of major bleeding within 2 to 4 days (2%-4%) and those with a low risk (0%-2%). Major bleeding is typically defined as intracranial bleeding or bleeding leading to death, requiring reoperation, causing a hemoglobin drop ≥ 2 g/dL, or necessitating transfusion of ≥ 2 units of red blood cells.^292^

**Table 12:** Risk of bleeding according to surgical procedure

High risk (risk of major bleeding within 2 days 2%-4%)	Abdominal aortic aneurysm repair Any major surgery (> 45 minutes) Bilateral knee replacement surgery Endoscopic ultrasound-guided fine needle aspiration Renal biopsy Laminectomy Urology, head and neck, abdominal, neurosurgery, breast cancer surgery Polypectomy, esophageal varices, biliary sphincterotomy, pneumatic dilation Transurethral resection of the prostate
Low risk (risk of major bleeding within 2 days 0%-2%)	Ventral hernia repair Abdominal hysterectomy Axillary lymph node dissection Bronchoscopy with or without biopsy Carpal tunnel release surgery Ophthalmic surgery Central venous catheter removal Cholecystectomy Skin, bladder, prostate, breast, thyroid, lymph node biopsies Dilation and curettage Gastrointestinal endoscopy with or without biopsy, enteroscopy, biliary or pancreatic stent without sphincterotomy Hemorrhoid surgery Hydrocelectomy Knee or hip replacement, hand, shoulder, foot surgery, arthroscopy Noncoronary angiography Dental extractions and other minor dental procedures

Furthermore, clinical conditions inherent to each patient can increase the risk of bleeding. Scores such as the HAS-BLED can quantify bleeding risk based on clinical characteristics of patients on anticoagulant therapy,^293^with a HAS-BLED score ≥ 3 being associated with a higher risk.

Some procedures have a minimal risk of bleeding and can be performed without interruption of anticoagulant therapy, such as pacemaker/implantable cardioverter-defibrillator insertion, coronary angiography, minor dental procedures (extraction of up to 2 teeth, restorations, prostheses, endodontics, cleaning, and implants), cataract surgery, minor dermatologic procedures, arthrocentesis, and intra-articular injection.^294^

#### 4.3.1. Warfarin^289 - 291 , 295^

Warfarin is a vitamin K antagonist whose anticoagulant effect takes days to wear off (half-life of 36 to 42 hours) and a similar time to be re-established after surgery. The flowchart for perioperative management of patients on warfarin is shown in Figure 5 .

**Figure 5:** Perioperative management of patients anticoagulated with warfarin. *See item 3.3. **Severe thrombophilia: deficiency of protein C, S, antithrombin or presence of antiphospholipid antibodies. **Mild thrombophilia: heterozygous mutation of factor V Leiden or the prothrombin gene. AF: atrial fibrillation; INR: international normalized ratio; TIA: transient ischemic attack; VTE: venous thromboembolism.

High-risk thromboembolism patients may require bridging therapy with parenteral anticoagulants such as unfractionated heparin (UFH) or low-molecular-weight heparin (LMWH). These anticoagulants have a faster onset of action and a shorter half-life, allowing for the anticoagulant to be interrupted as close as possible to the surgery, minimizing thromboembolic risk. As warfarin metabolism can be influenced by factors such as patient age, renal function, and drug interactions, it is suggested to measure the INR the day before surgery to ensure it is < 1.5, and if not, reverse it with oral vitamin K (1-2 mg) and reassess the INR the next day.

The decision whether to perform bridging therapy in patients on warfarin depends on a joint analysis of thromboembolic risk ( Table 11 ), bleeding risk ( Table 12 ), and the patient himself/herself ( Figure 5 ). The goal of heparin bridging is to minimize the time the patient is not on anticoagulation, thus reducing thromboembolic risk. However, observational studies and meta-analyses have shown that heparin bridging may increase bleeding.^296 , 297^The BRIDGE study, which randomized 1,884 patients to receive bridging therapy with heparin or placebo, found that bridging did not significantly reduce thrombotic events but increased major bleeding. Notably, the mean CHADS_2_score of the study population was 2.3, indicating a low thrombotic risk. This could mean that outcomes might differ for patients with a higher thrombotic risk (CHADS_2_score > 4).^298^Therefore, heparin bridging should not be indiscriminately administered to all anticoagulated patients.

##### 4.3.1.1. Patients with Moderate Thromboembolic Risk

There is a lack of evidence on the best approach for patients with moderate thromboembolic risk regarding bridging therapy. Thus, the decision should be based on individual patient and surgery characteristics, with bridging therapy indicated only in specific cases at the discretion of the attending physician.

##### 4.3.1.2. Urgent or Emergency Procedures

The therapeutic measures used to reverse oral anticoagulation with warfarin will depend on how quickly the prothrombin time (PT) measured by the INR needs to be corrected. In operations that can wait 18-24 hours, stopping warfarin and using intravenous vitamin K (2.5-5 mg) typically reduces the INR if it is within the therapeutic range.^289 - 291^

For rapid INR reduction, clotting factor deficiencies need to be treated with fresh frozen plasma (FFP) and prothrombin complex concentrate (PCC). Resolution No. 10 of January 23, 2004, of the Brazilian National Health Surveillance Agency (Agência Nacional de Vigilângia Sanitária, ANVISA) states that PCC is the treatment of choice for bleeding caused by coumarin anticoagulants and for rapid reversal of coumarin-associated complications. However, as PCC is not widely available in Brazilian hospitals, FFP is an acceptable alternative.^299^The recommended dose of FFP is 15 mL/kg, avoiding volume overload,^300^while there is no standardized dose for PCC. Table 13 outlines the doses used in some English services. However, regardless of the method used to replenish vitamin K-dependent coagulation factors, it is essential to administer vitamin K1 (2.5-5.0 mg orally or slowly intravenously) to maintain normal perioperative prothrombin levels.^289 - 291^

**Table 13:** Prothrombin complex concentrate doses for anticoagulation reversal according to INR values

INR	Dose based on factor IX
2.0–3.9	25 U/kg
4.0–5.9	35 U/kg
≥ 6.0	50 U/kg

INR: international normalized ratio.

Chart 24 presents recommendations for patients anticoagulated with warfarin undergoing urgent/emergency surgery.

**Chart 24:** Recommendations for patients anticoagulated with warfarin undergoing urgent/emergency surgery

Recommendation	Grade of recommendation	Level of evidence
Discontinuation of anticoagulation therapy, administration of intravenous vitamin K, and treatment of clotting factor deficiencies with prothrombin complex concentrate or fresh frozen plasma, depending on availability.	I	C

Chart 25 presents recommendations for patients anticoagulated with warfarin.

**Chart 25:** Recommendations for patients anticoagulated with warfarin

Recommendations for patients with high thromboembolic risk and patients with a mechanical heart valve	Grade of recommendation	Level of evidence
Warfarin should be discontinued 5 days before surgery until INR < 1.5.	I	C
Bridging therapy with full-dose unfractionated heparin (UFH) or low-molecular-weight heparin (LMWH) when INR < 2.	IIa	C
UFH should be discontinued 4-6 hours before surgery and LMWH 24 hours before surgery.	IIa	C
Postoperatively, full-dose UFH or LMWH and warfarin should be resumed at least 24 hours after surgery and heparin should be discontinued only when the INR is within the therapeutic range.	IIa	C
In patients undergoing surgery with high risk of hemorrhagic events, LMWH should be resumed 48-72 hours after surgery.	IIa	C
**Recommendations for patients with low thromboembolic risk**	**Grade of recommendation**	**Level of evidence**
Bridging therapy should not be performed (warfarin should be discontinued 5 days before surgery, and wait for INR < 1.5 before surgery).	IIa	C
Preoperative prophylactic UFH or LMWH may be used when indicated.	IIa	C
Postoperative prophylactic UFH or LMWH may be used when indicated and warfarin should be resumed 12-24 hours after surgery.	IIa	C

#### 4.3.2. Direct Oral Anticoagulants

Since 2010, the development of anticoagulants that act directly on the coagulation cascade, known as DOACs, has provided a promising alternative to vitamin K antagonists, which were the only available anticoagulants until then.^301^DOACs offer easier dosing regimens, pre-established doses, greater pharmacokinetic stability, and fewer drug and food interactions, without the need for serial PT/INR monitoring. These medications have been approved for use in the prevention of stroke in patients with nonvalvular AF, treatment of VTE (deep vein thrombosis/PE), prevention of recurrent VTE, and prevention of VTE in major orthopedic surgery.

The term "nonvalvular FA" has spawned confusion regarding which patients benefit from the use of DOACs. All pivotal trials of DOACs excluded patients with mechanical mitral valves or moderate-to-severe mitral stenosis, generating doubts regarding their use in other types of VHD. Currently, with new trials available, DOACs are authorized for anticoagulation in patients with the following valvular conditions: aortic regurgitation, mitral regurgitation, aortic stenosis, and mild mitral stenosis. Patients with bioprosthetic heart valves may also use DOACs instead of warfarin. In patients undergoing TAVI, DOACs may be used alone when anticoagulation is indicated. If anticoagulation is not indicated, it is not recommended to replace antiplatelets with DOACs.

In patients with moderate-to-severe mitral stenosis and those with a mechanical heart valve, current evidence contraindicates the use of DOACs. Regarding moderate-to-severe mitral stenosis, the INVICTUS study demonstrated that warfarin is superior to rivaroxaban in patients with rheumatic disease (reducing CV events and mortality), remaining the medication of choice in this population.^302^Regarding the use of DOACs in patients with mechanical aortic valves, the PROACT-Xa trial was prematurely terminated due to an increase in thromboembolic events with the use of apixaban.

##### 4.3.2.1. Dabigatran^295 , 303 - 306^

Dabigatran is an anticoagulant medication that acts as a reversible, direct thrombin inhibitor, preventing the conversion of fibrinogen to fibrin (factor IIa). It has a rapid onset of action, reaching peak activity between 30 and 120 minutes after administration, and a half-life of 12-17 hours. Excretion is predominantly renal (80%). Due to its rapid onset of action and shorter half-life, there is no need for bridging therapy with this medication. One concern associated with dabigatran use was the lack of specific antidotes until recently, when the available options were limited to PCC and hemodialysis, which had limited effect. The first antidote for reversal of direct thrombin inhibitors (dabigatran), idarucizumab, was approved by the U.S. Food and Drug Administration (FDA) in October 2015. Idarucizumab is a monoclonal antibody fragment that binds both free and thrombin-bound dabigatran with higher affinity than the binding affinity of dabigatran for factor II, completely reversing the anticoagulant effect of dabigatran. The recommended dose is 5 g administered intravenously as two bolus infusions of 2.5 g/50 mL 15 minutes apart.^307^

##### 4.3.2.2. Rivaroxaban^286 , 295 , 303 , 304 , 306^

Rivaroxaban is a factor Xa inhibitor that blocks the conversion of prothrombin to thrombin. It also has a rapid onset of action, reaching peak activity between 2 and 4 hours after administration, and a short half-life (5-9 hours in young individuals and 11-13 hours in older patients). Rivaroxaban is eliminated by both hepatic metabolism and renal excretion (66%). Due to its rapid onset of action and short half-life, bridging therapy is also not necessary with this medication. Previously, only PCC was available for attempting to reverse the effects of rivaroxaban, as no specific antidotes were available. Currently, andexanet alfa is a specific antidote that binds factor Xa inhibitors in the active site, rapidly reversing the anticoagulant effect of apixaban and rivaroxaban within minutes after administration. Andexanet alfa can be administered as an 800 mg IV bolus followed by an 8 mg/min continuous infusion for 120 minutes (high dose) or as a 400 mg IV bolus followed by a 4 mg/min continuous infusion for 120 minutes (low dose).^308^

##### 4.3.2.3. Apixaban^286 , 295 , 304 , 306^

Apixaban is another factor Xa inhibitor that prevents the conversion of prothrombin to thrombin. It has a rapid onset of action, reaching peak activity 3 hours after administration, and a short half-life (8-15 hours). Apixaban is eliminated by hepatic metabolism and renal (27%) and fecal excretion. Due to its rapid onset of action and short half-life, bridging therapy is also not necessary with this medication. Andexanet alfa is currently the specific antidote for factor Xa inhibitors, rapidly reversing the anticoagulant effect of apixaban and rivaroxaban within minutes after administration.^308^

##### 4.3.2.4. Edoxaban^309^

Edoxaban is another factor Xa inhibitor. It has a rapid onset of action, reaching peak activity between 1 and 2 hours after administration, and a short half-life (10-14 hours). Edoxaban is eliminated by renal (50%) and biliary/intestinal (50%) excretion. Due to its rapid onset of action and short half-life, bridging therapy is also not necessary with this medication. Despite being the most recently approved DOAC, andexanet alfa is also available as an antidote for this edoxaban.^310^

##### 4.3.2.5. Evaluation of the Anticoagulant Effect of DOACs

Although there is no universal test to accurately determine the anticoagulant effect of DOACs, conventional qualitative tests can be useful for this purpose and may be used in urgent situations. For dabigatran, thrombin time and activated partial thromboplastin time are used, and if elevated, it indicates the medication is still active. For factor Xa inhibitors, prothrombin activity and PT are used, but the INR should not be used.

##### 4.3.2.6. Interruption of DOACs Before Elective Surgery

Previous guidelines have relied on the pharmacokinetics of DOACs to estimate the optimal time to interrupt them before elective surgery, evaluating data on half-life, renal clearance, and patient renal function. However, in 2019, the PAUSE study^311^was published, which included 3,007 patients with AF using dabigatran, rivaroxaban, or apixaban. The study followed a standardized protocol for DOAC interruption and resumption based on DOAC pharmacokinetic properties, patient renal function, and procedure-associated bleeding risk, without using routine coagulation tests or heparin bridging. This strategy was associated with low rates of bleeding and thromboembolism, and these results can be extrapolated to clinical practice ( Figure 6 ). Several guidelines have incorporated the PAUSE study protocol into their recommendations for DOAC interruption before surgical procedures.^312 , 313^For patients undergoing surgical procedures with a low bleeding risk, DOAC can be suspended 1 day before surgery, and for those undergoing procedures with a high bleeding risk, DOAC should be discontinued 2 days before surgery. High-bleeding-risk procedures include any operation requiring neuraxial or epidural anesthesia, neurosurgery, major thoracic surgery (lobectomy, pneumonectomy, esophagectomy), major vascular surgery (aorta, lower limb revascularization, carotid endarterectomy), major abdominal and pelvic surgery (resection of hepatobiliary cancer, pancreatic cancer or pseudocyst, gastric and colorectal cancer; bower resection; resection of kidney, bladder, endometrial, or ovarian cancer; radical prostatectomy), major orthopedic surgery (hip arthroplasty or hip fracture, knee arthroplasty, metatarsal osteotomy), and other major cancer or reconstructive surgery (head and neck cancer).

**Figure 6:** CrCl: creatinine clearance; DOAC: direct oral anticoagulant. The black squares indicate the days on which the patient should not receive DOAC doses. High bleeding risk: any operation requiring neuraxial or epidural anesthesia, neurosurgery, major thoracic surgery (lobectomy, pneumonectomy, esophagectomy), major vascular surgery (aorta, lower limb revascularization, carotid endarterectomy), major abdominal and pelvic surgery (resection of hepatobiliary cancer, pancreatic cancer or pseudocyst, gastric and colorectal cancer; bower resection; resection of kidney, bladder, endometrial, or ovarian cancer; radical prostatectomy), major orthopedic surgery (hip arthroplasty or hip fracture, knee arthroplasty, metatarsal osteotomy), and other major cancer or reconstructive surgery (head and neck cancer).

Specifically for dabigatran, due to its high rate of renal excretion, if creatinine clearance is less than 50 mL/min, the medication should be interrupted 2 days before low-bleeding-risk operations and 4 days before high-bleeding-risk operations. In cases of regional anesthesia with an epidural catheter, wait at least 6 hours after catheter removal before the next DOAC dose.

Regarding edoxaban, as it is the most recently developed DOAC, studies evaluating its management in the perioperative period are scarce. The most accepted approach is to interrupt edoxaban 24 hours before low-bleeding-risk operations and 48 to 72 hours before high-bleeding-risk operations.

According to several guidelines,^312^prophylactic doses of LMWH should be started 12 hours after surgery (if adequate hemostasis) in patients/procedures with a high risk of VTE. The prophylactic doses should be stopped concomitantly with the reintroduction of oral anticoagulation.t

Chart 26 presents recommendations for DOAC management.

**Chart 26:** Recommendations for direct oral anticoagulant management

Recommendations for patients on chronic therapy with dabigatran	Grade of recommendation	Level of evidence
In patients with normal renal function undergoing low-bleeding-risk surgery, dabigatran should be interrupted 24 hours before surgery and reintroduced 24 hours after surgery.	I	B
In patients with normal renal function undergoing high-bleeding-risk surgery, dabigatran should be interrupted 48 hours before surgery and reintroduced 48 hours after surgery.	I	B
In patients with renal dysfunction (creatinine clearance < 50 mL/min) undergoing low-bleeding-risk surgery, dabigatran should be interrupted 48 hours before surgery and reintroduced 24 hours after surgery.	I	B
In patients with renal dysfunction (creatinine clearance < 50 mL/min) undergoing high-bleeding-risk surgery, dabigatran should be suspended 96 hours before surgery and reintroduced 48 hours after surgery.	I	B
In cases of regional anesthesia with an epidural catheter, wait at least 6 hours after catheter removal before starting the first dose of dabigatran.	I	B
**Recommendations for patients on chronic therapy with rivaroxaban**	**Grade of recommendation**	**Level of evidence**
In patients with normal renal function, rivaroxaban should be interrupted 24 hours before surgery and reintroduced 24 hours after surgery.	I	B
In cases of severe renal dysfunction (creatinine clearance 15 to 30 mL/min) or high-bleeding-risk surgery, rivaroxaban should be interrupted at least 48 hours before the procedure and reintroduced 48 hours after the procedure.	I	B
In cases of regional anesthesia with an epidural catheter, wait at least 6 hours after catheter removal before the next dose of rivaroxaban.	I	B
**Recommendations for patients on chronic therapy with apixaban**	**Grade of recommendation**	**Level of evidence**
In patients with normal renal function, apixaban should be interrupted 24 hours before surgery and reintroduced 24 hours after surgery.	I	B
In cases of moderate renal dysfunction (creatinine clearance 15 to 50 mL/min) or high-bleeding-risk surgery, apixaban should be interrupted 48 hours before surgery and reintroduced 48 hours after surgery.	I	B
In cases of regional anesthesia with an epidural catheter, wait at least 6 hours after catheter removal before the next dose of apixaban.	I	B

### 4.4. Infective Endocarditis (IE) Prophylaxis

IE has high morbidity and mortality.^314 , 315^Strategies to reduce its incidence are predominantly based on experimental models, observational studies, and expert opinions and show some divergences among the main guidelines.^316 - 318^

The primary determinant of IE is structural heart disease, particularly VHDs.^315^In Brazil, the main cause of chronic VHD is rheumatic fever, whose prevalence and mortality have been increasing annually, predominantly affecting young adults.^319^Endothelial injury caused by structural lesions promotes subendothelial exposure, leading to platelet activation followed by fibrin deposition. Circulating microorganisms adhere to damaged or inflamed endothelium and proliferate, culminating in IE. In the case of highly virulent microorganisms such as *Staphylococcus aureus* , intravenous drug use, and intravascular devices, IE can occur in individuals with structurally normal hearts.^320^

Because IE is most caused by bacterial infection, several studies have investigated the risk of bacteremia related to routine activities and invasive procedures. In low- and middle-income countries, such as Brazil, IE is mostly caused by *Streptococcus spp* ., particularly the viridans group streptococci found in the oral cavity. In high-income countries, there is a greater incidence of health-care-associated IE caused by *Staphylococcus aureus* .^315^We describe below the prophylaxis recommendations for each type of procedure based on the latest scientific evidence, focusing on the particularities of the Brazilian population.

#### 4.4.1. Dental Procedures

Since the 1930s, several studies have correlated dental extraction and endodontic and/or periodontal manipulation with the presence of transient bacteremia.^321 - 324^After experimental animal models confirmed the reduction of bacteremia after dental extraction with the use of prophylactic antibiotics,^325 , 326^prophylaxis before dental procedures has been recommended for individuals with structural heart disease.

The positive impact of prophylaxis and the risks associated with antibiotic use have been evaluated in recent years. Observational studies have estimated the prevalence of IE related to dental procedures to be 2.7% to 13%.^327 - 329^Routine activities such as chewing, tooth brushing, and flossing have also been correlated with transient bacteremia.^325 , 330 - 333^Additionally, the use of antibiotics carries potential risks of adverse events, such as anaphylaxis, and frequent use increases the likelihood of antimicrobial resistance.^333 , 334^

Given the lack of strong scientific evidence and the potential risks of prescribing antibiotics vs. the high morbidity and mortality of IE, recommendations for prophylaxis before dental procedures have been revised. Currently, prophylaxis is recommended for individuals at high risk of IE, with some differences between guidelines.

In 2008, the UK National Institute for Health and Clinical Excellence (NICE) started not recommending prophylaxis against IE for individuals undergoing dental procedures.^318^An observational study from England showed an increase in the incidence of IE after the restriction of prophylaxis from 2008 onwards, although it is not possible to establish a direct correlation with NICE recommendations.^335 , 336^In 2016, the recommendation was updated to "prophylaxis is not routinely recommended," allowing the use of antibiotics on an individualized basis and specifically mentioning patients at high risk of IE.^337^In 2020, another observational study found an increase in the incidence of IE in England, but did not detect any change in trends directly following the update of prophylaxis recommendations in 2008, either overall or in cases associated with oral streptococci.^338^

The American Heart Association (AHA) and the European Society of Cardiology (ESC) have recommended prophylaxis for individuals at high risk of IE since 2007^339^and 2009, respectively.^317^The population considered at high risk of developing IE-related complications is described in Table 14 .^317 , 339^A study showed an increase in the incidence of streptococcus IE in the United States compared with other etiologies following the update of prophylaxis recommendations in 2007, although there was no increase in the rates of hospitalization and valve surgery for IE.^340^Other observational studies did not find an increase in the total number of IE cases or IE caused by microorganisms related to the oral cavity in the USA after the 2007 update of prophylaxis recommendations.^335 , 341 , 342^

**Table 14:** Patients indicated for infective endocarditis (IE) prophylaxis^316 - 318^

Brazilian Society of Cardiology	
**Individuals at high risk of developing IE:**	
	Acquired valvular heart disease with stenosis or regurgitation
	Prosthetic heart valve
	Acquired valvular heart disease repaired with prosthetic material
	Previous IE
	Hypertrophic cardiomyopathy
	Uncorrected congenital heart disease
	Congenital heart disease repaired with prosthetic material (first 6 months)
	Corrected congenital heart disease with residual lesions
	Valvular heart disease in heart transplant recipients
**Not indicated for prophylaxis:** patients with cardiac implantable electronic devices (pacemaker, cardioverter-defibrillator), repaired septal defect, vascular stents, inferior vena cava filter, central nervous system shunts, fistulas, or venous catheters.	
**American Heart Association (AHA)**	
**Particularities:** prophylaxis is indicated for individuals at high risk of adverse outcomes from infective endocarditis. Does not include acquired valvular heart disease with stenosis or regurgitation.	
**European Society of Cardiology (ESC)**	
**Particularities:** does not include acquired valvular heart disease with stenosis or regurgitation, valvular disease in heart transplant recipients, and congenital heart disease.	
**National Institute for Health and Clinical Excellence (NICE)**	
**Particularities:** does not include acquired valvular disease corrected with prosthetic material and valvular heart disease in heart transplant recipients. Prophylaxis is recommended for congenital heart disease, except isolated atrial septal defect, fully repaired ventricular septal defect, and fully repaired patent ductus arteriosus.	

Given the low access to dental care in Brazil, potentially leading to high rates of poor dental hygiene and thus a higher risk of bacteremia related to invasive dental procedures,^343^coupled with the rarity of severe adverse events with antibiotic use and the lack of prophylaxis studies in our country, this Guideline recommends prophylaxis for patients with native VHD.

Therefore, IE prophylaxis is recommended for all patients with significant VHD ( Table 14 ) undergoing dental procedures involving gingival and periodontal manipulation or mucosal incision ( Table 15 ). The antibiotic should be administered in a single dose 30 to 60 minutes before the procedure ( Table 16 ). If the antibiotic is not used before the procedure, it can be administered up to 2 hours after the procedure.

**Table 15:** Infective endocarditis prophylaxis in dental procedures

Prophylaxis indicated	Patients undergoing procedures involving gingival and periodontal manipulation and/or mucosal incision
**Prophylaxis not indicated**	Local anesthesia in noninfected tissue
	Dental radiography
	Placement, adjustment, or removal of dental appliances
	Natural loss of deciduous teeth
	Bleeding from oral mucosa or lip trauma

**Table 16:** Prophylactic regimens to be administered 1 hour before dental procedures

Route of administration		Antibiotics	Dose (adults)	Dose (children)
Oral		Amoxicillin	2 g	50 mg/kg
	Allergy to penicillin	Azithromycin or clarithromycin	500 mg	15 mg/kg
		Cephalexin *	2 g	50 mg/kg
		Doxycycline	100 mg	2,2 mg/kg < 45 kg, 100 mg > 45 kg
		Clindamycin	600 mg	20 mg/kg
Parenteral (IV or IM)		Ampicilin	2 g	50 mg/kg
	Allergy to penicillin	Cefazolin or ceftriaxone *	1 g	50 mg/kg
		Clindamycin	600 mg	20 mg/kg

* Avoid cephalosporins in cases of anaphylaxis, angioedema, or urticaria with penicillin due to the risk of cross-reactivity.

IE more commonly results from bacteremia due to daily routine activities than dental procedures. There is no doubt that maintaining good oral health is the best strategy for preventing IE. In individuals with periodontal and endodontic disease, the incidence and magnitude of bacteremia due to daily activities and surgical procedures are higher compared with individuals with healthy teeth.^343^Therefore, we recommend daily dental care and biannual evaluation by a dentist.

Chart 27 presents recommendations for prophylaxis before dental procedures.

**Chart 27:** Recommendations for prophylaxis before dental procedures

Recommendation	Grade of recommendation	Level of evidence
Patients with risk factors for infective endocarditis according to the Brazilian Society of Cardiology ( Table 14 ) undergoing procedures involving gingival and periodontal manipulation and/or mucosal incision.	I	B

#### 4.4.2. Respiratory Tract Procedures

Patients undergoing procedures that involve incision or biopsy of the respiratory mucosa, such as otorhinolaryngological surgery, should receive an antibiotic regimen similar to that used before dental procedures with a high risk of bacteremia. Antibiotic prophylaxis is not recommended for bronchoscopy, laryngoscopy, and orotracheal intubation. Patients undergoing treatment for an established infection, such as drainage of an abscess, should receive an antibiotic regimen that contains an antistaphylococcal medication.^317^

#### 4.4.3. Gastrointestinal and Genitourinary Procedures

Despite limited evidence, we recommend prophylaxis before gastrointestinal and genitourinary procedures involving mucosal incision or biopsy in patients at high risk of IE. These include cesarean section and vaginal delivery, which have the potential for bacteremia, although there are no specific studies on the efficacy of this conduct.^344^

For upper gastrointestinal endoscopy and colonoscopy, we recommend prescribing prophylaxis whenever there is a high possibility of mucosal manipulation (eg, biopsies, resections). If unexpected mucosal manipulation occurs without prior prophylaxis administration, antibiotics can be administered up to 2 hours after the procedure. Prophylaxis is not indicated for other endoscopic exams, such as transesophageal echocardiography and endoscopic retrograde cholangiopancreatography.

The recommended antibiotic prophylactic regimen for gastrointestinal and genitourinary procedures is described in Table 17 .

**Table 17:** Prophylactic regimens to be administered 30 minutes before gastrointestinal and genitourinary procedures

Antibiotic		Dose (adults)	Dose (children)
	Intravenous ampicillin * + intravenous gentamicin	2 g 1.5 mg/kg	50 mg/kg 1.5 mg/kg
Allergy to penicillin:			
	Intravenous vancomycin + intravenous gentamicin	1 g 1.5 mg/kg	20 mg/kg 1.5 mg/kg

* Administer an additional dose of intravenous ampicillin 1 g 6 hours after the procedure, or alternatively, oral amoxicillin 1 g.

Chart 28 presents recommendations for prophylaxis before gastrointestinal and genitourinary procedures.

**Chart 28:** Recommendations for prophylaxis before gastrointestinal and genitourinary procedures

Recommendation	Grade of recommendation	Level of evidence
Patients with risk factors for infective endocarditis according to the Brazilian Society of Cardiology ( Table 14 ).	IIa	C

#### 4.4.4. Dermatological or Musculoskeletal Procedures

Patients undergoing treatment for an established infection, such as drainage of an abscess, should receive an antibiotic regimen that contains an agent active against staphylococci and streptococci.^317^

#### 4.4.5. Body Piercing and Tattooing

There are case reports of IE after piercing and tattooing, particularly tongue piercing.^345 , 346^Therefore, patients should be warned about the risks associated with piercing and tattooing, and these procedures should be discouraged.^317^

## 5. Perioperative Biomarkers

### 5.1. Natriuretic Peptides

Natriuretic peptides are released into the bloodstream by the myocardium in response to several physiological stimuli, such as myocardial stress and ischemia, among others. Several studies have demonstrated that elevated preoperative BNP levels are strong predictors of perioperative CV complications.^347 , 348^

In 2012, Biccard et al. conducted a prospective observational study involving 788 patients undergoing noncardiac surgery to investigate the clinical utility of preoperative BNP measurement compared with other biomarkers, such as cardiac troponins (cTn). Elevated preoperative levels of both cTn and BNP were independent predictors of CV events.^349^

A meta-analysis of 15 prospective observational studies including 4,856 patients found that elevated preoperative levels of BNP or NTproBNP were associated with a significantly increased risk (20 times higher) of MACE, CV mortality, and all-cause mortality (10 times higher) in the perioperative period (< 43 days post-surgery).^350^However, the data from these studies did not establish a specific cutoff point for BNP levels nor did they determine whether these biomarkers add prognostic value to existing risk indices.^351^

A multicenter prospective observational study including 979 patients assessed the incremental value of hs-cTnT for risk prediction prior to noncardiac surgery in comparison with the RCRI score. The study also investigated the role of natriuretic peptides as risk predictors in noncardiac surgery. Both hs-cTnT and NTproBNP levels were higher among individuals who died compared to those who survived. The authors suggested that NTproBNP levels greater than 300 pg/mL confer a higher risk of mortality (4.8% vs 1.4%, p = 0.002).^352^

In 2014, a meta-analysis of 18 prospective observational studies assessed individual data from 2,179 patients undergoing noncardiac surgery and revealed that pre and postoperative BNP and NTproBNP levels enhance risk stratification for death or nonfatal MI within 30 days after the procedure. Preoperative BNP levels > 92 pg/mL or NTproBNP > 300 pg/mL increased the risk of death or nonfatal MI by 3.4 times (OR 3.4, 95%CI 2.6-4.5), while postoperative BNP levels > 400 pg/mL and NTproBNP > 900 pg/mL increased the risk by 2.7 and 1.8 times, respectively. Both pre and postoperative natriuretic peptide levels were independent predictors of death and nonfatal MI within 30 days. The meta-analysis also indicated that BNP levels < 30 pg/mL have a negative predictive value for perioperative CV events.

Additional postoperative natriuretic peptide measurement can improve risk stratification for CV events at 30 and 180 days after noncardiac surgery compared with preoperative measurement alone.^353^More recently, a subanalysis of the VISION (Vascular Events in Noncardiac Surgery Patients Cohort Evaluation) study involving 10,402 patients over 45 years of age undergoing noncardiac surgery (vascular and nonvascular) confirmed previous findings that natriuretic peptides are good predictors of vascular death or MI in the postoperative period of noncardiac surgery.^354^NTproBNP values between 100-200 pg/mL increased the risk of primary events by 2.3 times, values between 200-1500 pg/mL by 3.6 times, and values greater than 1,500 pg/mL by 5.5 times. The authors also showed that preoperative NTproBNP improves CV risk prediction in addition to the RCRI.^355^

Given the evidence presented, a slight modification in recommendations is proposed as described below.

Chart 29 presents recommendations for preoperative BNP/NTproBNP measurement for risk prediction of perioperative CV events.

**Chart 29:** Recommendations for preoperative BNP/NTproBNP measurement for risk prediction of perioperative cardiovascular events

Recommendation	Grade of recommendation	Level of evidence
Patients older than 65 years or patients aged 45-64 years with established cardiovascular disease or risk factors * undergoing noncardiac surgery.	I	B

* Diabetes, hypertension, coronary artery disease, obesity, atrial fibrillation (risk factors for heart failure with preserved ejection fraction).

### 5.2. Cardiac Troponins and Surveillance CV Complications

Despite the several algorithms and tools available for adequate risk stratification in patients undergoing noncardiac surgery, the accuracy in predicting adverse events remains suboptimal.^356^Among the different tools that can assist in the prediction of CV events and postoperative mortality, the use of biomarkers has gained prominence due to the scientific evidence accumulated over recent decades, particularly the use of natriuretic peptides (BNP/NT-proBNP) and hs-cTnT/I.

Several studies have consolidated the role of hs-cTn in the optimization of preoperative risk stratification in noncardiac surgery. In a study with 979 patients undergoing noncardiac surgery, a preoperative hs-cTnT measurement above the 99th percentile was related to an increased risk for the combined outcome of mortality, MI, recovered cardiac arrest, and acute HF (RR 2.6, 95%CI 1.3-5.3). Hs-cTnT was also superior to the RCRI in predicting in-hospital mortality (AUC 0.809 vs 0.658, p = 0.006).^352^A meta-analysis of 7 studies with over 4,000 patients revealed an increase in combined CV events (RR 2.9, 95%CI 1.9-4.4), short-term mortality (RR 5.4, 95%CI 3.21-9.06), and long-term mortality (RR 2.9, 95%CI 1.8-4.6) in patients with elevated hs-cTnT before noncardiac surgery.^357^

Hs-cTnI assays have also been evaluated in the perioperative setting. In the BASEL-PMI and Tropovasc studies, hs-cTnT and hs-cTnI levels were measured in 1,022 patients undergoing noncardiac surgery. Both hs-cTnI and hs-cTnT showed good accuracy in predicting combined CV events within 30 days after noncardiac and nonvascular surgery (AUC hs-cTnI 0.77, 95%CI 0.71-0.83; and AUC hs-cTnT 0.79, 95%CI 0.73-0.85). In patients undergoing vascular surgery, hs-cTnI performed better than hs-cTnT (AUC hs-cTnI 0.67, 95%CI 0.59-0.75; and AUC hs-cTnT 0.59, 95%CI 0.51-0.67).^358^

Finally, in hospitalized patients scheduled for surgery, several comorbidities and acute and chronic conditions can affect cTn levels (eg, anemia, sepsis, renal failure, HF).^359 , 360^Different cohorts have observed that hs-cTn levels, particularly hs-cTnT, may be above the 99th percentile of the URL in up to 50% of patients undergoing noncardiac surgery.^352 , 358 , 361 - 365^

Therefore, although elevated preoperative cTn levels are a risk factor for postoperative CV events, there is no specific evidence or recommendations for additional investigations or specific measures to reduce risk in this population. Elevated values should be interpreted as chronic myocardial injury (defined by the Fourth Universal Definition of MI as at least one cTn value above the 99th percentile of the URL without dynamic changes).^360^Abnormally high values in relation to known comorbidities should be investigated before elective operations. In other cases, the value should be considered the patient’s baseline and used for the correct interpretation of subsequent measurements, facilitating the correct diagnosis of acute perioperative myocardial infarction/injury (PMI) and differentiating acute from chronic biomarker elevations.^361^

As PMI is often asymptomatic, measuring of cTn levels on postoperative days 1 and 2 should be performed to diagnose PMI (see item 6.1). There is still no definitive consensus on which patients benefit the most cost-effectively from cTn screening. Indiscriminate cTn screening ensures no PMI is missed but results in many normal results. Conversely, cTn screening only on very high-risk patients increases the identification of PMI but misses the diagnosis in many patients.

In the VISION study, cTn levels were measured in all patients over 45 years of age undergoing surgery with regional or general anesthesia, who were hospitalized for at least 1 day.^365^In the BASEL-PMI study, patients over 65 years of age or over 45 with known atherosclerotic disease (coronary, cerebrovascular, or peripheral) were screened.^361^Recent data show that the relative percentage of individuals with myocardial injury after non-cardiac surgery (MINS) was higher among those classified as high risk by the RCRI (7.9%, 14.2%, 25.3%, and 38% in classes I, II, III, and IV, respectively). However, in absolute numbers, 30% of all MINS cases were in class I patients (ie, with a RCRI score of 0).^366^Conversely, there is no evidence to recommend routine cTn measurements in patients without risk factors or RCRI class I undergoing low-complexity procedures.^367^However, perioperative cTn screening in patients over 65 years of age or with a history of atherosclerotic disease has been shown to be cost-effective and has received support from some authors.^361 , 368 , 369^

The 2022 ESC Guidelines recommend cTn screening for patients with CV risk factors (age ≥ 65 years, dyslipidemia, hypertension, diabetes, smoking, family history), known CVD, or CVD symptoms.^3^Considering the Brazilian context and the available evidence, this Guideline recommends systematic surveillance with ECG and cTn measurement before surgery and daily for 48 hours after surgery in patients with an intermediate- or high-risk estimate according to preoperative evaluation algorithms. Of note, approximately 80% to 90% of cTn changes, alone or not, occurred up to postoperative day 2,^361 , 365^making it reasonable to measure cTn levels for 48 hours after surgery.

It is worth noting that with the availability of hs-cTn and new evidence, systematic ECG monitoring and automated ST-segment monitoring stopped being used due to significant variability in sensitivity (between 55% and 100%) and specificity (between 37% and 85%) for the detection of intra and postoperative ischemia, as its effectiveness depends on the technique used and the baseline characteristics of the study population (pretest probability of CAD).^370 - 372^

Chart 30 presents recommendations for perioperative surveillance.

**Chart 30:** Recommendations for perioperative surveillance

Recommendation	Grade of recommendation	Level of evidence
Measurement of cardiac troponin before surgery and on postoperative days 1 and 2 in patients at intermediate or high risk of complications according to algorithms, undergoing intermediate-or high-risk noncardiac surgery.	I	B
Electrocardiography before surgery and on postoperative days 1 and 2 in patients at intermediate or high risk of complications according to algorithms, undergoing intermediate-or high-risk noncardiac surgery.	I	C
High-risk patients according to algorithms undergoing intermediate-or high-risk noncardiac surgery should stay in the ICU for 48 hours after surgery.	I	C
Intermediate-risk patients according to algorithms undergoing intermediate- or high-risk noncardiac surgery should stay in the ICU for 48 hours after surgery.	IIa	C
Measurement of troponin levels in low-risk patients or those undergoing low-risk surgery.	III	B

## 6. Diagnosis and Treatment of Perioperative CV Complications

### 6.1. Perioperative Acute myocardial Infarction/Injury (PMI)

AMI is the most feared perioperative CV complication, occurring in 0.3% to 3% of low-risk patients with no history of CAD and in almost 33% of high-risk patients with a history of CAD.^373^In recent observational studies, AMI incidence ranged from 0.4% to 0.9%, although it may be underdiagnosed without adequate monitoring.^374 , 375^Although mortality rates have decreased in recent decades, hospital readmission or death within 30 days due to AMI still occurs in 1 in 3 patients,^375 - 377^which is likely to be related to the presence of comorbidities, diagnostic difficulties, and limitations in using antithrombotic and antiplatelet therapies in patients with perioperative AMI.

Most patients with perioperative AMI are asymptomatic because some of the events occur during the intraoperative period and the administration of potent analgesia postoperatively.^356 , 361 , 365 , 378 , 379^Additionally, when present, chest pain is often attributed to more obvious causes such as incisional or postural pain. Other perioperative manifestations, such as dyspnea and nausea, may be explained by alternative causes (atelectasis and medication effects), meaning that the hypothesis of perioperative AMI is often not considered by the medical team. Furthermore, only 25% of patients present ischemic changes on the ECG,^361 , 380^and these changes need to be differentiated from other causes of ECG alterations, such as electrolyte disturbances, hypothermia, medication effects, or incorrect lead placement. Therefore, cTn screening is crucial for the diagnosis of AMI.

In recent years, postoperative cTn elevation has been associated with increased 30-day and 1-year mortality, regardless of the presence of other criteria from the universal definition of AMI ( Figure 7 ). Considering all the particularities of perioperative AMI, we have chosen to use the term "perioperative acute myocardial infarction/injury (PMI)" for events occurring within the first 2 days after surgery during the phase of cTn screening.^361^

**Figure 7:** Fourth universal definition of acute myocardial infarction.

PMI is defined as the occurrence of an acute increase in cTn, defined as an absolute delta above or equal to the 99th percentile of the URL of the cTn assay between the preoperative value and the value on postoperative day 1 or 2, or between two postoperative concentrations if the preoperative value is missing ( Figure 8 ).^361 , 378^In the BASEL-PMI study, in which cTn screening was performed in patients over 65 years or over 45 years with atherosclerotic disease (coronary, cerebral, or peripheral) undergoing noncardiac surgery, the incidence of PMI using hs-cTnT (delta ≥ 14 ng/L) was 16%. In alignment with findings from previous studies, only 18% of patients were symptomatic. Patients with PMI had significantly higher mortality than patients without PMI at 30 days (9% vs 1%, p < 0.001, adjusted hazard ratio 2.7; 95%CI 1.5-4.8) and 1 year (23% vs 9%, p < 0.001, adjusted hazard ratio 1.6; 95%CI 1.2-2.2).^361^These findings were later validated for hs-cTnI as well.^378^

**Figure 8:** PMI diagnosis. URL: 99th percentile upper reference limit value of the troponin assay – URL hs-cTnT (Roche) 14 ng/L, URL hs-cTnI (Abbott) 26 ng/L, URL hs-cTnI (Siemens) 40 ng/L. ECG: electrocardiogram; hs-cTn: high-sensitivity cardiac troponin; PMI: perioperative myocardial infarction/injury.

Hs-cTnI is produced by several manufacturers, and each has its own 99th percentile URL. The definition of PMI has been validated for Abbott (delta ≥ 26 ng/L) and Siemens (delta ≥ 40ng/L) hs-cTnI assays, but may be extrapolated to other assays.^378^Although there was no difference in mortality between patients with PMI meeting one of the criteria from the universal definition of AMI or not, patients with PMI meeting any additional criteria from the AMI definition ( Figure 7 ) had a higher incidence of MACE (including AMI, acute HF, arrhythmias, and CV death) than patients with PMI and increased cTn alone.^378^

Another condition specific to the perioperative period is MINS (myocardial injury after non-cardiac surgery), which is defined as a postoperative cTnT value ≥ 65 ng/L or a cTnT value between 20 and 65 ng/L AND an absolute delta ≥ 5 ng/L between pre and postoperative values. MINS also refers to cTn elevation caused by ischemia, excluding other causes such as sepsis, PE, tachyarrhythmias, and HF.^365^The VISION study included patients over 45 years undergoing noncardiac surgery and found an incidence of MINS of 18%, with 93% of patients with MINS being asymptomatic. MINS occurrence was associated with a significant increase in mortality (adjusted hazard ratio 3.20; 95%CI 2.4-4.3). Furthermore, higher absolute postoperative cTn values, as well as greater acute deltas, were associated with increased mortality.^365^It should be noted that the definition of MINS considers only the absolute postoperative cTn value and does not differentiate acute from chronic myocardial injury.

Traditionally, the etiology of perioperative AMI was considered a combination of type 1 AMI (plaque rupture due to increased platelet aggregation, intraplaque inflammation, decreased fibrinolysis, or increased catecholamine) and type 2 AMI (oxygen supply/demand mismatch due to anemia, tachycardia, hypoxemia, or hypotension).^356 , 373^Pathologic and *in vivo* studies have shown that approximately 50% of perioperative AMIs are type 1.^379 , 381 - 383^However, with the development of hs-cTn tests, increases in cTn can be detected in several other conditions in addition to AMI, such as sepsis, HF, renal failure, and even after strenuous exercise.^359 , 384^Therefore, the etiology of PMI is much broader, as shown in Figure 9 ,^385^and the prognosis of PMI also directly depends on its etiology. Patients with PMI due to extracardiac causes (eg, sepsis), HF, and tachyarrhythmias have the highest mortality.^386^

**Figure 9:** PMI and MINS etiology. PMI: perioperative acute myocardial infarction/injury, AMI: acute myocardial infarction, MINS: myocardial injury after noncardiac surgery; hs-cTnT: high-sensitivity troponin T.

ECG is mandatory in patients with PMI, and an echocardiogram should be considered to assess biventricular systolic function, VHD, and wall motion abnormalities.

Treatment of PMI fundamentally depends on its etiology. The adequate approach for a patient with PMI is shown in Figure 10 . It is worth noting that in the presence of type 1 AMI, the choice of antiplatelet medication to be added to ASA depends on the bleeding risk, which should be discussed with the surgeon. In high-bleeding-risk patients, clopidogrel should be preferred. In cases of non-ST-elevation MI, the second antiplatelet medication should be administered during or after coronary cineangiography. Additionally, secondary causes of ischemia (tachycardia, hypotension, hypertension, anemia, pain) should always be treated.^373^Although evidence for PMI treatment is scarce, retrospective studies suggest that optimized clinical management of CV risk factors (dyslipidemia, hypertension, diabetes, smoking cessation) is associated with improved prognosis and should therefore be performed in all patients.^387^

**Figure 10:** Flowchart for the management of patients with perioperative acute myocardial infarction/injury. *Clopidogrel or ticagrelor depending on bleeding risk;^#^Timing depending on bleeding risk after discussion by the multidisciplinary team;^&^If not performed preoperatively, perform on an outpatient basis or before discharge, as applicable;^$^On an outpatient basis or before discharge, as applicable. AMI: acute myocardial infarction; ASA: acetylsalicylic acid; CAD: coronary artery disease; CCA: coronary cineangiography; CT: computed tomography; DAPT: dual antiplatelet therapy; ECG: electrocardiogram; Hb: hemoglobin; LMWH: low-molecular-weight heparin; PMI: perioperative acute myocardial infarction/injury; UFH: unfractionated heparin.

A recent study showed that risk factors for MACE in patients with PMI likely due cardiac coronary causes include the presence of angina or dyspnea, an absolute increase in cTn 2 to 4 times the 99th percentile of the URL, emergency surgery, and high-risk surgery. Surprisingly, the presence of perioperative bleeding was a protective factor, probably because no other events occurred after the cause of ischemia was corrected.^386^

There is currently no evidence to determine the best time for performing noninvasive functional imaging for myocardial ischaemia tests in patients with PMI. However, considering that the risk of CV events and mortality after PMI is highest in the first 120 days after surgery, it is reasonable to perform the functional stress test before this period.

Regarding MINS treatment, the randomized MANAGE study investigated the use of dabigatran 110 mg twice daily in patients with MINS and demonstrated a reduction in the combined outcome of vascular mortality, nonfatal AMI, ischemic stroke, arterial thrombosis, amputation, and VTE.^388^

Although less frequent, perioperative AMI can occur after postoperative day 2, following the cTn screening period. In these cases, diagnosis should be made using the universal definition of MI criteria ( Figure 7 ).^360^Treatment is the same as for AMI outside the perioperative setting, respecting the particularities previously described (consider bleeding risk and multidisciplinary discussion with the surgeon).^389^

Chart 31 presents recommendations for the diagnosis and treatment of perioperative AMI.

**Chart 31:** Recommendations for the diagnosis and treatment of perioperative AMI

Recommendation	Grade of recommendation	Level of evidence
PMI diagnosis should be made in the presence of an absolute delta ≥ the 99th percentile of the upper reference limit of the troponin assay between the preoperative value and the value on postoperative day 1 or 2, or between two postoperative concentrations if the preoperative value is missing.	I	B
Clinical evaluation, ECG, and hemoglobin measurement in patients with PMI.	I	B
Determination of PMI etiology using the management flowchart.	I	B
Treatment of PMI cause according to specific guidelines.	I	C
Diagnosis of AMI after postoperative day 2 should be based on the universal definition of MI, and treatment should be based on current guidelines.	I	C
In patients with perioperative AMI or PMI due to ischemia, all secondary causes of ischemia (anemia, tachycardia, hypotension, hypertension) should be treated, the risk of bleeding should be determined, and multidisciplinary discussion with the surgeon should be conducted.	I	C

### 6.2. Acute Atrial Fibrillation/Flutter

The diagnosis of AF/flutter requires assessment of heart rhythm, preferably with a standard 12-lead ECG to avoid artifacts and misdiagnoses. When using a single lead (including hospital monitors), AF is defined as an arrhythmia lasting ≥ 30 seconds.^390^In noncardiac surgery, a meta-analysis showed a 10%^391^incidence of postoperative AF (POAF), with a higher incidence in thoracic surgery.^392^Although often self-limiting and asymptomatic, POAF is associated with a higher risk of late recurrence and cardioembolic events.^393^

POAF is defined as new-onset AF during or after the first hours of surgery, with a peak incidence between postoperative days 2 and 4.^394^The pathogenesis of POAF results from the interaction between inflammation, pre-existing triggers, structural disease, and perioperative insults.^395^Its occurrence after noncardiac surgery is associated with a 3-fold increase in the risk of stroke, a 5-fold increase in the risk of MI, and a 3-fold increase in mortality.^396^Preventive measures against AF include adequate pre and postoperative regulation of hydroelectrolytic balance (normovolemia, monitoring, and replenishment of magnesium and potassium) and maintenance of current medications when appropriate in the hemodynamic setting.^397^

Several medications have been studied to reduce the incidence of AF and its deleterious effects. Preventive antiarrhythmic therapy with amiodarone or intravenous magnesium can be individually discussed in patients scheduled for thoracic surgery. The use of oral magnesium (total of 3.2 g over 3 days and 1.6 g on the day of surgery) in patients undergoing myocardial revascularization (POMAF-CS single-center, randomized controlled study) reduced the incidence of POAF compared with placebo.^398^In a retrospective study by Tisdale et al., the use of intravenous amiodarone during anesthetic induction (43.75 mg/hour for 96 hours) in patients undergoing esophagectomy reduced the rate of perioperative AF but had no impact on length of hospital stay. Additionally, amiodarone resulted in hypotension, bradycardia, and prolonged corrected QT intervals, raising questions about whether it should be used routinely.^399^Riber et al., in a randomized, double-blind, placebo-controlled study of patients undergoing pneumonectomy, demonstrated that administration of intravenous amiodarone 300 mg postoperatively, followed by 1200 mg orally per day for 5 days in hemodynamically stable patients, reduced the rate of perioperative AF (9% vs 32% in the control group).^392^Khalil et al. compared three groups in the immediate postoperative period: group 1 received amiodarone (5 mg/kg bolus, followed by 15 mg/kg per day for 48 hours); group 2 received intravenous magnesium (80 mg/kg bolus, followed by 8 mg/kg/h for 48 hours); and the control group was derived from a retrospective analysis of patients undergoing lung resection. They found an AF rate of 10% in group 1, 12.5% in group 2, and 20.5% in the control group.^400^Other nonantiarrhythmic medications have also been evaluated. Statins showed a potential role in preventing POAF in cardiac surgery, particularly myocardial revascularization.^401 , 402^However, a later meta-analysis focusing on the postoperative period of noncardiac surgery did not observe any effect of statin use on POAF prevention.^272^Colchicine is currently under investigation (Colchicine For The Prevention Of Perioperative Atrial Fibrillation In Patients Undergoing Thoracic Surgery [COP-AF], NCT03310125).^403^

The management of POAF in patients with conditions that decrease the likelihood of obtaining rhythm control involves initially correction of volemia and electrolyte imbalance, reduction of pain and postoperative inflammation, and HR control (< 100 to 110 bpm).^404^In patients with preserved ejection fraction, calcium channel blockers and beta-blockers are preferred, while in patients with reduced ejection fraction, digoxin and specific beta-blockers are preferred. Digoxin may be less effective in hyperadrenergic states during surgery. When initiating treatment with a new medication, the dose should be slowly titrated to avoid hypotension, which is known to be deleterious in the postoperative period. In patients with rapid ventricular response and difficult chronotropic control, rhythm control (that is, conversion of AF) is the preferred choice, either by electrical or pharmacological cardioversion with monitoring of arrhythmia onset and the potential need for transesophageal echocardiography, as per the Brazilian AF guidelines.^405^

Regardless of the rhythm or HR control strategy, patients should be evaluated for anticoagulation initiation. The use of long-term anticoagulation for POAF was previously discussed individually for each patient. However, a meta-analysis of cardiac and noncardiac surgery observed a 37% higher risk of stroke, which was higher among patients undergoing noncardiac surgery. Therefore, long-term anticoagulation in patients with POAF and risk factors (CHA2DS2VASc) is recommended to prevent thromboembolic events, considering the net clinical benefit, bleeding risk based on the surgery performed, and patient preference ( Figure 11 ).

**Figure 11:** Flowchart for the treatment of postoperative atrial fibrillation. *Dosages according to AF Guideline and clinical judgment. AF: atrial fibrillation; POAF: postoperative atrial fibrillation; HR: heart rate; ECV: electrical cardioversion; PE: pulmonary embolism.

Chart 32 presents recommendations for patients with POAF.

**Chart 32:** Recommendations for patients with POAF

Recommendation	Grade of recommendation	Level of evidence
Long-term anticoagulation should be considered in patients with AF detected after noncardiac surgery, when stroke risk is assessed according to CHA2DS2VASc and bleeding risk according to the surgery performed.	IIa	B

### 6.3. Acute Heart Failure

With the aging of the world’s population, the prevalence of HF is increasing.^89^Older patients with multiple comorbidities are increasingly undergoing noncardiac surgery, which means that the occurrence of postoperative acute HF as a complication of noncardiac surgery is also likely to increase.^93^Few studies have specifically evaluated the occurrence of postoperative acute HF. Its incidence in retrospective studies or studies evaluating postoperative acute HF as part of a combined endpoint of MACE ranges from 1% to 3.8%.^406 - 412^

A retrospective study with data from a U.S. database found that 4.9% of patients undergoing noncardiac surgery experienced HF during hospitalization (not specified if pre or postoperative), with an in-hospital mortality rate higher than that of patients without HF (5% vs 0.8%, p < 0.001). Most cases occurred in orthopedic and vascular operations.^93^

A recent prospective cohort study involving 11,162 noncardiac operations found an incidence of postoperative acute HF of 2.5%. Independent risk factors for postoperative acute HF include age, chronic HF, diabetes, AF, anemia, chronic obstructive pulmonary disease, CAD, peripheral artery disease, chronic myocardial injury, and emergency surgery. Approximately 50% of patients with postoperative acute HF had no known preoperative history of HF. The 1-year mortality rate for these patients was 44%, compared with 11% for those without postoperative acute HF (p < 0.001). Additionally, patients with postoperative acute HF had a rate of rehospitalization for HF of 15%.^412^

Therefore, postoperative acute HF is a perioperative complication associated with high mortality rates that is often overlooked.

Diagnosing postoperative acute HF is challenging because patients are bedridden, older, and often asymptomatic. Thus, in older patients with risk factors undergoing orthopedic or vascular operations, attention should be given to signs of postoperative acute HF. Diagnosis is clinical and supported by natriuretic peptide levels. An echocardiogram is recommended to assess biventricular systolic function and exclude significant VHD.

Treatment of postoperative acute HF should follow current guidelines for HF management outside the perioperative period.^413^Medical therapy should be optimized before hospital discharge to prevent rehospitalizations for HF, and early outpatient follow-up should be scheduled for clinical reassessment and medication adjustment.

Chart 33 presents recommendations for patients with postoperative acute HF.

**Chart 33:** Recommendations for patients with postoperative acute heart failure

Recommendation	Grade of recommendation	Level of evidence
Natriuretic peptide measurement and echocardiogram during hospitalization.	I	C
Treatment according to current guidelines and optimization of medication and volemia before discharge.	I	C
Early outpatient follow-up for clinical reassessment and medication adjustment.	I	C

### 6.4. Venous Thromboembolism

The treatment of patients with perioperative venous thromboembolism (VTE) follows the principles applied to general patients. Special attention should be given to the bleeding risk associated with surgical procedures. In this setting, the surgical team should always be consulted before initiating antithrombotic therapy.

In the acute phase of VTE, LMWH or fondaparinux is preferred over UFH. If these medications are contraindicated or there is a high bleeding risk, UFH is the treatment of choice.^414 - 417^For hemodynamically unstable patients, systemic thrombolysis is the treatment of choice, although often contraindicated postoperatively.^418 - 421^In such cases, pulmonary embolectomy or catheter-directed therapy should be considered.^422 - 425^

For patients with an indication for oral anticoagulation, DOACs are preferred over vitamin K antagonists^426 - 431^due to lower bleeding rates and greater convenience for patients and health care professionals (fixed doses, fewer drug and food interactions, and no need for serial blood tests to ensure a specific therapeutic range). If there is an absolute contraindication to anticoagulation, a vena cava filter may be considered.^432 - 434^

Anticoagulant therapy is recommended for a minimum duration of 3 months.^435 , 436^Prolonging treatment generally reduces the recurrence of thromboembolic events but increases the risk of bleeding.^437^Several criteria should be evaluated before deciding to prolong treatment, which is beyond the scope of this Guideline.^438^

Chart 34 presents recommendations for patients with postoperative VTE.

**Chart 34:** Recommendations for patients with postoperative venous thromboembolism

Recommendation	Grade of recommendation	Level of evidence
Unstable patients with pulmonary embolism (PE)		
Thrombolytic therapy in unstable patients with PE if there are no contraindications.	I	B
Pulmonary embolectomy in unstable patients with contraindications for thrombolytic therapy or when thrombolytic therapy fails.	I	C
In hemodynamically unstable patients, parenteral anticoagulation with unfractionated heparin (UFH) is preferred over low-molecular-weight heparin (LMWH) or fondaparinux.	I	C
Catheter-directed therapy in unstable patients with contraindications for thrombolytic therapy when thrombolytic therapy fails, or with high surgical risk.	IIa	C
**Anticoagulation in stable patients**		
In patients with venous thromboembolism (VTE) indicated for parenteral anticoagulation, LMWH or fondaparinux is preferred over UFH.	I	A
In patients with VTE requiring parenteral anticoagulation who have contraindications for LMWH or fondaparinux, UFH should be used.	I	A
In patients with VTE indicated for oral anticoagulation, DOACs are preferred over warfarin.	I	A
In patients with VTE requiring oral anticoagulation who have contraindications for DOACs, warfarin should be used.	I	A
A vena cava filter may be considered in patients with an absolute contraindication to anticoagulation.	IIa	C
Patients with PE should receive anticoagulation therapy for at least 3 months.	I	A
